# Human tear film protein sampling using soft contact lenses

**DOI:** 10.1186/s12014-024-09475-8

**Published:** 2024-03-13

**Authors:** Robert K. Roden, Nathan Zuniga, Joshua C. Wright, David H. Parkinson, Fangfang Jiang, Leena M. Patil, Rebecca S. Burlett, Alyssa A. Nitz, Joshua J. Rogers, Jarett T. Pittman, Kenneth L. Virgin, P. Christine Ackroyd, Samuel H. Payne, John C. Price, Kenneth A. Christensen

**Affiliations:** 1 Department of Chemistry & Biochemistry, Brigham Young University, Provo, UT 84602 USA; 2 College of Optometry, Rocky Mountain University of Health Professions, Provo, UT 84606 USA; 3 Department of Biology, Brigham Young University, Provo, UT 84602 USA

**Keywords:** Tear sampling, Soft contact lenses, Mass spectrometry, Proteomics, Etafilcon A, Basal tears

## Abstract

**Background:**

Human tear protein biomarkers are useful for detecting ocular and systemic diseases. Unfortunately, existing tear film sampling methods (Schirmer strip; SS and microcapillary tube; MCT) have significant drawbacks, such as pain, risk of injury, sampling difficulty, and proteomic disparities between methods. Here, we present an alternative tear protein sampling method using soft contact lenses (SCLs).

**Results:**

We optimized the SCL protein sampling in vitro and performed in vivo studies in 6 subjects. Using Etafilcon A SCLs and 4M guanidine-HCl for protein removal, we sampled an average of 60 ± 31 µg of protein per eye. We also performed objective and subjective assessments of all sampling methods. Signs of irritation post-sampling were observed with SS but not with MCT and SCLs. Proteomic analysis by mass spectrometry (MS) revealed that all sampling methods resulted in the detection of abundant tear proteins. However, smaller subsets of unique and shared proteins were identified, particularly for SS and MCT. Additionally, there was no significant intrasubject variation between MCT and SCL sampling.

**Conclusions:**

These experiments demonstrate that SCLs are an accessible tear-sampling method with the potential to surpass current methods in sampling basal tears.

**Graphical Abstract:**

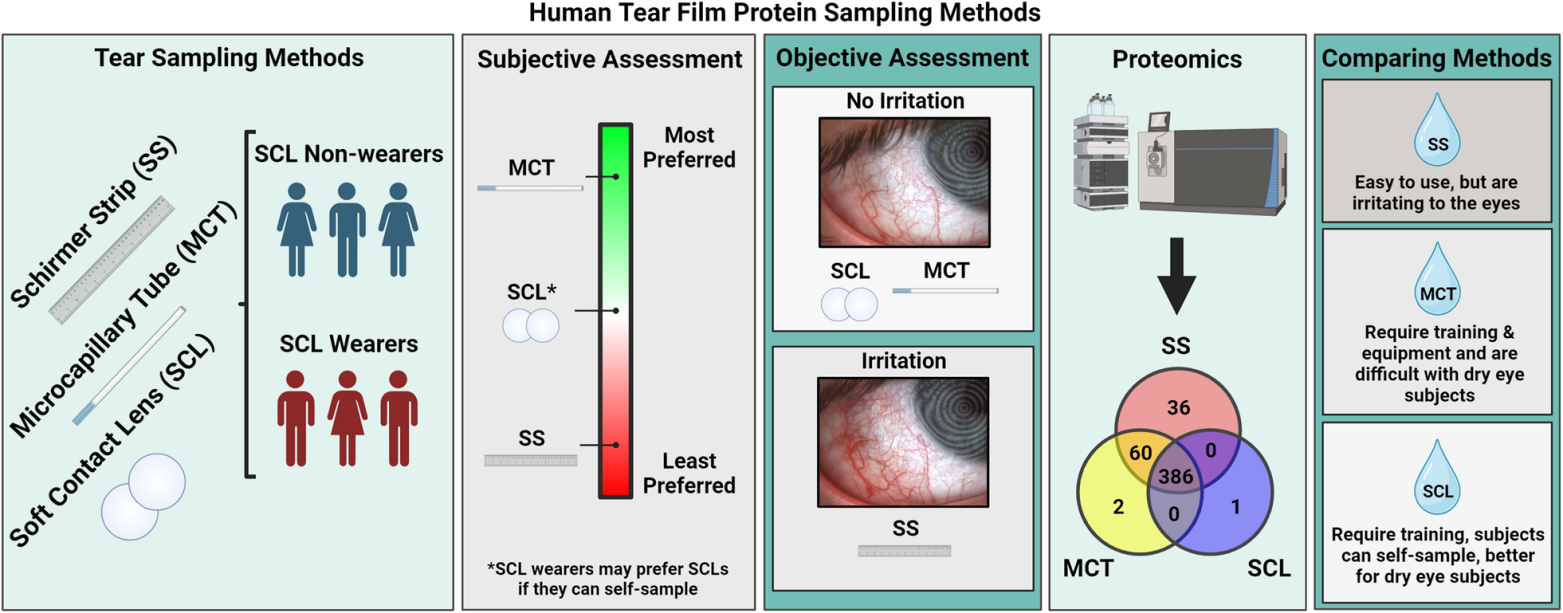

**Supplementary Information:**

The online version contains supplementary material available at 10.1186/s12014-024-09475-8.

## Introduction

Human tear film is an attractive biospecimen, given its accessibility and potential for use in diagnostic screenings [1]. In recent years, human tear protein biomarkers have been identified for various diseases, including glaucoma, multiple sclerosis, Parkinson’s disease, Alzheimer’s disease, and various forms of cancer [2, 3]. Mass spectrometric identification of relevant disease biomarkers could lead to clinical diagnostics directly from tears [3, 4]. However, a considerable obstacle to tear analysis is sampling. The most common tear sampling methods are cellulose filter Schirmer strips (SS) and microcapillary tubes (MCT) [5]. Each method has considerable drawbacks, including pain, irritation, conjunctival epithelium damage, corneal injury risk, difficulty capturing tears, and/or low sample volume [6, 7]. Furthermore, the tear sampling method impacts downstream analysis because of proteomic differences between methods [8, 9]. Thus, improved tear sampling methods are needed.

Soft contact lenses (SCLs) are FDA-approved hydrogels for vision correction. During contact lens wear, SCLs capture and concentrate proteins in tear film by adsorption and absorption [10, 11]. We hypothesized that if such proteins could be sampled and analyzed, SCLs would provide an alternative tear sampling method. As SCLs are designed for interaction with the ocular surface and optimized for comfort, we further hypothesized that SCLs would have advantages over current tear sampling methods. Here, we present an objective and subjective analysis of SCLs as a tear protein sampling method and compare these results to SS and MCT sampling. These data demonstrate that SCLs likely sample basal tears and yield comparable protein levels to SS and MCT. Our method has been tested using tear proteomics but may also be helpful for other applications.

## Materials and methods

### SCL protein quantification

Senofilcon A (Oasys, Acuvue), Nesofilcon A (Biotrue ONEday, Bausch & Lomb), Balafilcon A (Purevision 2, Bausch & Lomb), and Etafilcon A (1-Day Moist, Acuvue) lenses with a spherical equivalent (SE) power between − 0.50D and − 1.50D were tested in triplicate. Lenses were soaked for 1 h in a simulated human tear protein mixture (HTPM) (human albumin, human lactoferrin, and human lysozyme, 2/2/0.1, w/w/w in Milli-Q H_2_O) to a final concentration of 9 mg/mL. Lenses were removed from the protein solution with forceps and were lightly touched to a Kimwipe to wick away excess fluid. Lenses were then placed in a microcentrifuge tube with 400 µL phosphate buffered saline (PBS, Genesee Scientific), 4M guanidine (GoldBio), or 10% HPLC grade acetone (Fischer Chemical) and sonicated for 10 min to desorb proteins from the lens. The total protein in the solution was measured using the Pierce BCA Protein Assay kit (ThermoFisher Scientific) referencing HTPM in 4M guanidine as the standard.

### SCL total protein capture time course

Etafilcon A lenses were fully submerged and soaked in 400 µL HTPM for 5 min, 1 h, 4 h, and 16 h. After incubation, excess fluid was wicked using a Kimwipe, protein was removed with 4M guanidine and sonication, and total protein was measured as previously described.

### SCL total protein capture by dioptric power

− 8.00, − 0.75, and + 2.00 spherical Etafilcon A lenses were removed from their blister packs with forceps and touched to a Kimwipe. Individual lenses were transferred to microcentrifuge tubes and submerged in 400 µL HTPM for 1 h. After incubation, excess fluid was wicked using a Kimwipe, then captured protein was removed with 4M guanidine and sonication, and total protein was measured as previously described.

### Human subject enrollment

Human subjects research was performed in accordance with the Declaration of Helsinki; approval was granted by the Internal Review Board at Brigham Young University (IRB2022-166). Samples were collected at Alpine Vision Center (AVC) (Saratoga Springs, UT). Subjects were educated on the study’s purposes, risks, and benefits. Informed consent was obtained before subject enrollment, and the privacy rights of human subjects were observed. Enrollment was based on predetermined inclusion and exclusion criteria. Subjects 18 years or younger and pregnant women were excluded from the study. Only subjects with a tear meniscus height > 0.3 mm were allowed to participate.

Three female and three male subjects between the ages of 21 and 29 were recruited. Of these subjects, two males and one female reported having previously used SCLs. At the beginning of the study, subjects were briefly instructed on SS, MCT, and SCL tear sampling methods. Each subject then had photos taken of their eyes, answered a pre-sampling questionnaire, donated tears by SS, MCT, or SCL, repeated anterior segment photos, answered a post-sampling questionnaire, and waited 45 min before repeating the cycle until all 3 sampling methods were performed. An optometrist collected all samples; subjects could not sample their own tears.

### Bulbar conjunctival injection (BCI) assessment

Photographs of the inferior temporal bulbar conjunctiva were taken using a Keratograph 5M (Oculus) before and after each sampling method. Photos were printed, and each subject’s pre- and post-sampling images were randomly placed side by side. Photos blinded for the sampling method were shown to three optometrists who determined which photo in each pair had greater BCI. Photo pairs with greater BCI before tear sampling were counted as “− 1,” and those with greater BCI after tear sampling were counted as “1”. All photo pairs were scored.

### Subjective tear sampling method assessment

Subjects were given a brief overview of all sampling methods at the beginning of the study. Before donating each tear sample, subjects were asked to quantify their anxiety about the tear sampling method on a scale of 0 (no anxiety) to 10 (extreme anxiety). After sampling, the subjects were given a questionnaire assessing their anxiety about repeating the method, discomfort, and difficulty, each on a scale of 0 (none) to 10 (extreme). Subjects were also asked if they would be willing to repeat the sampling method if it could provide useful information about their eye health. They were asked to explain if the subject was unwilling to repeat the experiment. Finally, subjects were asked to rate their overall tear sampling experience from 0 (terrible) to 10 (excellent).

After the study, subjects were asked to preferentially rank the tear sampling methods from 2 (most preferred) to 0 (least preferred) and explain their answer. Subjects answered questionnaires individually and privately.

### Tear sampling

SS: Wearing nitrile powder-free exam gloves (NIGHT ANGEL, Adenna), the optometrist inserted the tip of a SS (Gulden Ophthalmics) between the lid and the globe of the inferior temporal portion of the subject’s eye. After 5 min, the optometrist donned a new set of gloves, removed the SS, and placed it in a microcentrifuge tube.

MCT: Subjects were seated at an SL-D2 slit lamp (Topcon) and instructed to look in superior nasal gaze. A 5 µL Microcaps MCT (Drummond) was gently positioned into the inferior temporal tear prism until tears were drawn up the tube. The optometrist attempted to minimize contact with the ocular surface. Tears were then pushed out of the MCT into a microcentrifuge tube using a rubber bulb.

SCL: Using new gloves for each lens, the optometrist placed Etafilcon A SCLs (− 0.50 DS) on each eye. After 5 min, the optometrist donned a fresh set of gloves, removed the lenses, and placed them in microcentrifuge tubes.

All samples were cold-chain transported on ice and stored at – 80 ℃. All sampling was done without anesthesia. Subjects could open or close their eyes during SS and SCL sampling. Tear samples were collected from both eyes for all methods, though only tears sampled from the right eye were analyzed in our study.

### MS sample preparation

After thawing all samples, SSs were cut into 2 mm squares with clean scissors. 400 µL 4M guanidine in MS-grade water was added to each SS, MCT, and SCL sample. All samples were sonicated at room temperature for 10 min and then incubated at 100 ℃ for 5 min. Total protein was then measured as described previously with standards prepared in 4M guanidine. All tear samples were normalized to 20 µg per sample.

Next, 250 mM tris (2-carboxyethyl) phosphine hydrochloride (TCEP, ThermoFisher Scientific) was added to each sample at volumes sufficient to make a 5 mM final concentration. 250 mM 2-chloroacetamide (CAA, Acros Organics) was added to make a final concentration of 15 mM. Samples were then placed on a heat block at 100 ℃ for 5 min. The entire reaction solution was transferred to a 30 kD Nanosep filter (Pall, Port Washington, NY), centrifuged at 14,000 × *g* for 10 min, and washed with 300 µL 25 mM triethylamine bicarbonate (TEAB, pH 8.5, ThermoFisher Scientific). 100 µL TEAB was mixed with 1 µg/µL trypsin (ThermoFisher Scientific) in the volume above the filter; the samples were pulsed in the centrifuge for 2 s and then placed on a shaker in a 37 ℃ incubator for 14 h. Samples were then centrifuged at 14,000 × *g* for 30 min before adding 100 µL 25 mM TEAB pH 8.5 and repeating centrifugation for 30 min. The filtrate was then transferred to vials and labeled using the TMT10-plex Isobaric Label Reagent Set (ThermoFisher Scientific). TMT labeling was performed according to the manufacturer’s protocol, except 8 µL of TMT label was added per sample (35 µg total protein each). Two TMT 10-plexes were created, each consisting of 9 individual tear samples and 1 pooled sample of equal amounts of the 9 protein aliquots.

### Mass spectrometry

Tear peptides were resuspended in OrbiA (3% ACN, 0.1% FA, 96.9% Optima-LC/MS grade H2O, [all chemicals from Fisher Chemical]) to a concentration of 1 µg/µL. Samples were analyzed with online nanoflow (300 nL/min) liquid chromatography tandem mass spectrometry (LC–MS/MS) using an Ultimate 3000-RSLC nano HPLC system (ThermoFisher Scientific) coupled to an Orbitrap Fusion Lumos MS (ThermoFisher Scientific). 6 µL of each sample was separated with a 75 µm inner diameter (360 µm outer diameter), 25 cm in length microcapillary column packed with 2 µm C18 beads heated to 35 ℃ and ionized using an electrospray emitter tip (10 µm). HPLC solvent A (OrbiA) and solvent B (OrbiB, 80% ACN, 0.1% FA, 19.9% MS grade H2O). Each sample ran for 266 min with the following gradient at 300 nL/min: 0–2 min, 5% B; 2–231 min, 5 to 32% B; 231–244 min, 32 to 42% B; 244–256 min, 42 to 99% B; 256–266 min, 99% B; the separation gradient was followed with a seesaw wash: 266–269 min, 99 to 2% B; 269–271 min, 2% B; 271–273 min, 2 to 100% B; 273–276 min, 100% B; 276–279 min, 100 to 2% B; 279–281 min, 2 to 100% B; 281–284 min, 100% B; 284–286 min, 100–0% B; 286–288 min, 0% B.

The Orbitrap Fusion Lumos MS was operated in data-dependent mode with a 3 s cycle time to acquire CID MS/MS scans. MS1 data was acquired by orbitrap with a resolution of 120,000. The following filters were used to select MS2 scans: precursor range, 400–1400 m/z; monoisotopic peak determination, peptide; intensity threshold, 5.0 × 103; theoretic precursor fit threshold, 70% with a 0.5 m/z fit window; charge states, 2–6; dynamic exclusions, precursor exclusion after 1 time for 60 s. Selected precursors were activated (normalized) HCD with a fixed 35% collision energy, and MS2 data was detected with the ion trap at a scan rate of 125,000 Da/sec set to 50 ms max injection time. As part of the TMT 10-plex workflow, selected precursors with an exclusion window of 2 m/z were selected for MS3 fragmentation. Synchronous precursor selection was set to 10, and precursors were activated with 65% (normalized) HCD. Orbitrap scans with a range of 110–500 were collected at a resolution of 50,000 with a max injection time of 105 ms.

### Data analysis

Statistical analysis of in vitro SCL testing, subject questionnaire data, and inter-method BCI comparisons were performed using one-way ANOVA. The absolute value of the average BCI score was used to calculate statistical significance between sampling methods. One-sample t-tests were used to calculate the BCI score significance for each method.

MS data was analyzed using Peaks software (Bioinformatics Solutions Inc.). Spectrum filter settings were set to a false discovery rate (FDR) of 1% (− 10logP ≥ 26.4) and quality ≥ 8.7. Protein filter settings were significance ≥ 0, fold change ≥ 1, and at least one unique peptide. All spectra with intensity < 1 × 10^2^ were excluded. Only proteins identified in ≥ 3 subjects were counted between TMT 10-plexes for the qualitative analysis. Only proteins identified in all subjects within each TMT-10plex were counted in our quantitative analysis. Within each TMT 10-plex, samples were normalized to the average signal intensity of the pooled sample, log2 transformed, and normalized to the slope.

Inter-method comparisons were made by assessing data normality using the Shapiro–Wilk test (Additional file 1: Figure S1) and then performing paired, two-tailed t-tests for each protein. FDR was accounted for by adjusting p-values with the Benjamini–Hochberg equation (FDR = 0.25). Adjusted p-values < 0.05 were considered significant.

Groups of proteins unique to each sampling method and shared between methods were analyzed separately for functional enrichment relative to the combined list of all identified proteins using the STRING database [12]. The common Repository of Adventitious Proteins (cRAP) database was used to screen all identified tear proteins for contaminants (accessed 8/21/23).

## Results

To develop a method for SCL tear sampling, we began by selecting SCL candidates. Senofilcon A, Nesofilcon A, Balafilcon A, and Etafilcon A were chosen to represent a broad spectrum of chemical compositions and characteristics (Additional file 1: Figure S2).

**Fig. 1:**
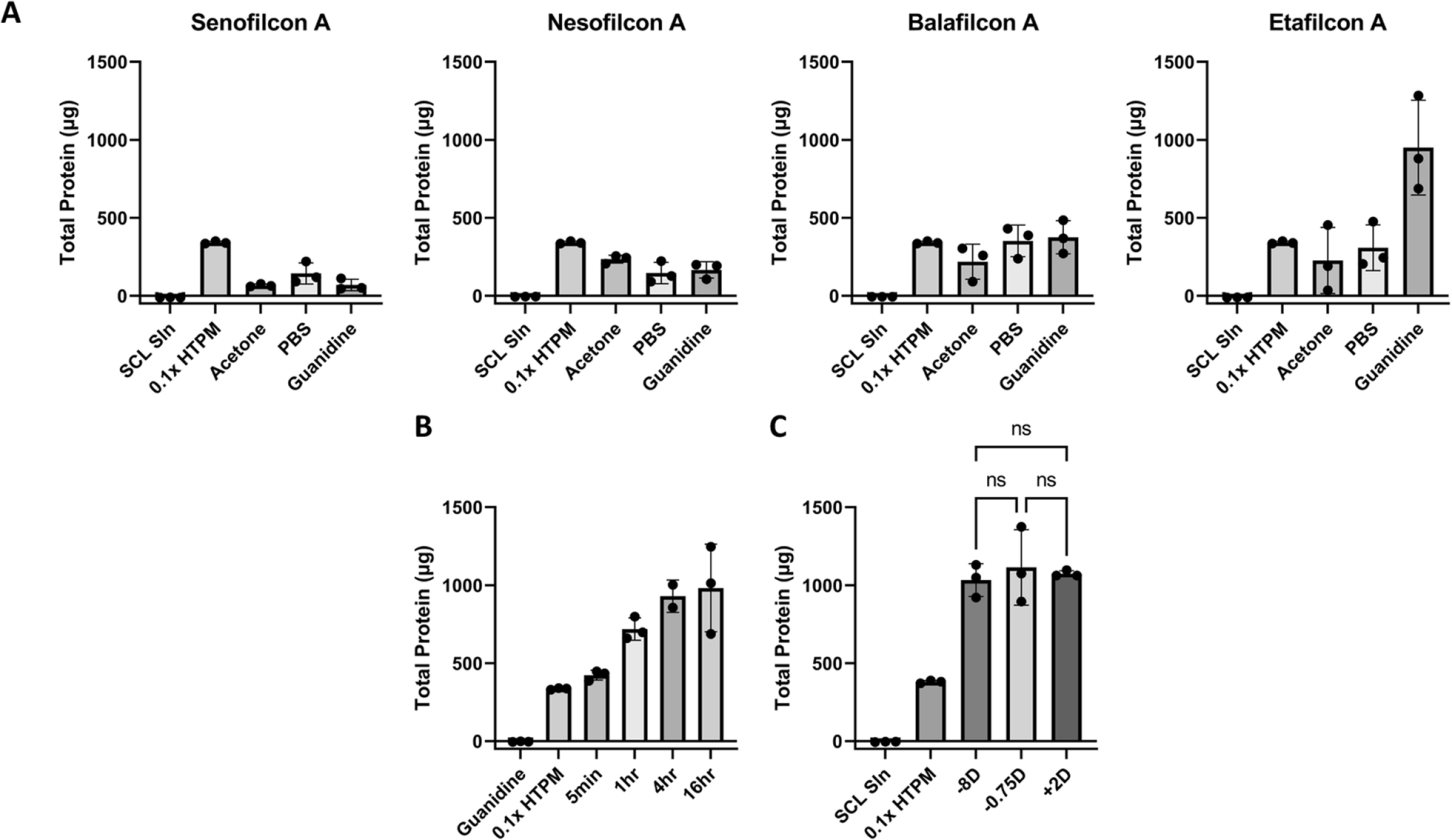
SCL protein sampling in vitro. **A** Total protein of Nesofilcon A, Balafilcon A, Etafilcon A, and Senofilcon A lenses after soaking for 1 h in HTPM, then sonicated for 10 min in PBS, 10% acetone, and 4M guanidine, **B** Total protein from Etafilcon A lenses soaked in HTPM for 5 min, 1 h, 4 h, and 16 h, **C** Total protein by SCL power (− 0.75DS, − 8.00DS, and + 2.00DS) after a 1 h incubation in HTPM. Error bars represent standard deviation, n = 3 for all experiments. *NS*  not significant

We first compared the ability of the selected SCLs to capture human tear proteins in vitro. Each SCL (n = 3) was exposed to a simulated human tear protein mixture (HTPM) that contained human albumin, lactoferrin, and lysozyme at a physiological total protein concentration. After protein capture, three protein removal methods were tested: a saline solution (1 × PBS), a chaotropic agent (4M guanidine), and an organic solvent (10% acetone) (Fig. 1A). The etafilcon A SCL and guanidine combination provided the highest protein yield, with a ~ threefold increase in recovered protein compared to other SCL/chemical combinations.

To estimate how long SCLs should be in contact with tears on the eye, we assessed the impact of SCL protein capture time in vitro by soaking SCLs in HTPM for 5 min, 1 h, 4 h, and 16 h, followed by a 10-min incubation in 4M guanidine (Fig. 1B). A 5-min SCL soak in HTPM captured an average of 450 ± 37 μg of protein. Protein capture increased with time and yielded an average of 1.07 ± 0.28 mg at 16 h. However, since several hundred μg is ample for most MS proteomics experiments, we concluded that a 5 min on-eye sampling was sufficient.

Given that SCL dioptric power is related to lens thickness, and thicker lenses could potentially absorb more protein, we assessed whether SCL dioptric power affected total protein capture. As shown in Fig. 1C, differences in dioptric power showed no statistically significant difference in collected protein yield. We conclude that yields of captured protein are largely insensitive to SCL dioptric power.

**Fig. 2:**
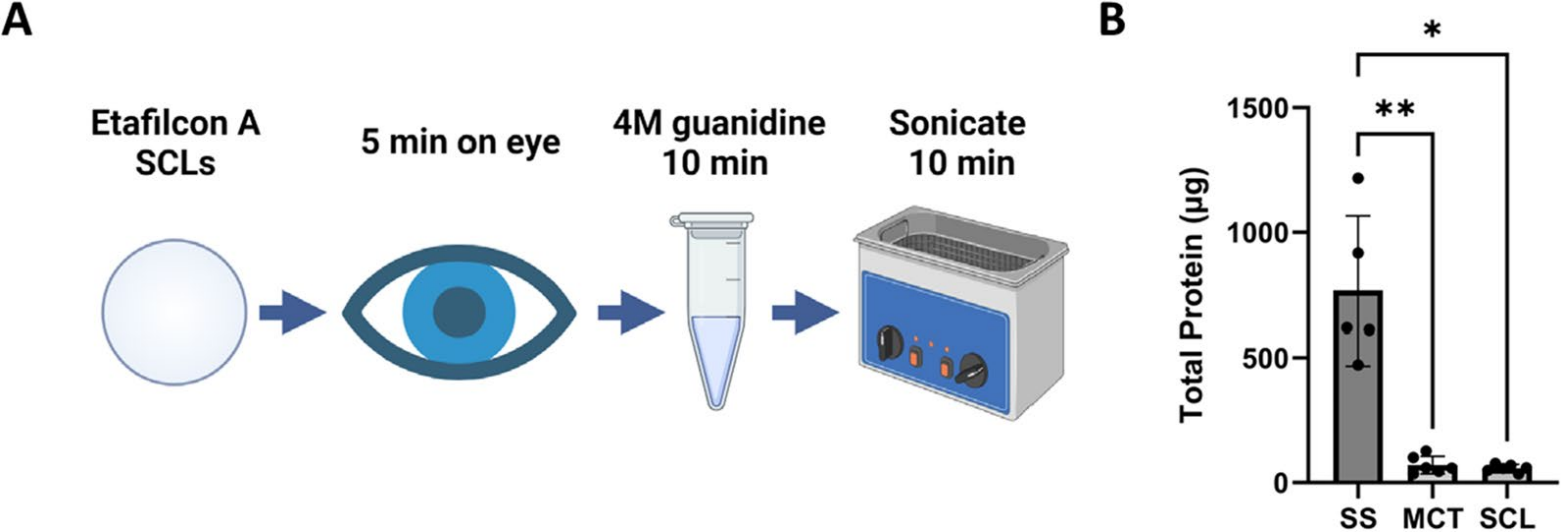
SCL human tear sampling. **A** Method overview, **B** Total protein from human subjects for 1 eye using SS, MCT, and SCL sampling. Error bars represent standard deviation, n = 6. * = p-value ≤ 0.05, ** = p-value ≤ 0.01

After analyzing the data from our in vitro experiments, we created a SCL protein sampling method that would be used to analyze human tear film (Fig. 2A). Etafilcon A lenses are placed on the subject’s eye for 5 min. The lens is removed, placed in 400 µL 4M guanidine, and sonicated for 10 min. This solution is then ready for downstream analysis. We analyzed the SCL elution using MS as described in Materials and Methods.

We tested our SCL tear film sampling method on 6 human subjects: 3 male and 3 female subjects between the ages of 21 and 29. Each subject donated tears by SS, MCT, and SCL, yielding averages of 260 ± 60, 40 ± 28, and 60 ± 31 µg total tear protein, respectively (Fig. 2B). As expected, SS yielded the largest amount of protein. The SCL and MCT methods gave similar results. We attribute the difference in total protein between SS and MCT/SCL primarily to sampling volume since SS methods have a significantly larger tear sampling capacity. For SCL sampling, differences between in vitro and in vivo sampling may reflect differential protein capture in HTPM versus individual subject tear proteins or partial mechanical protein removal due to wiping of the SCLs by the eyelids.

**Fig. 3:**
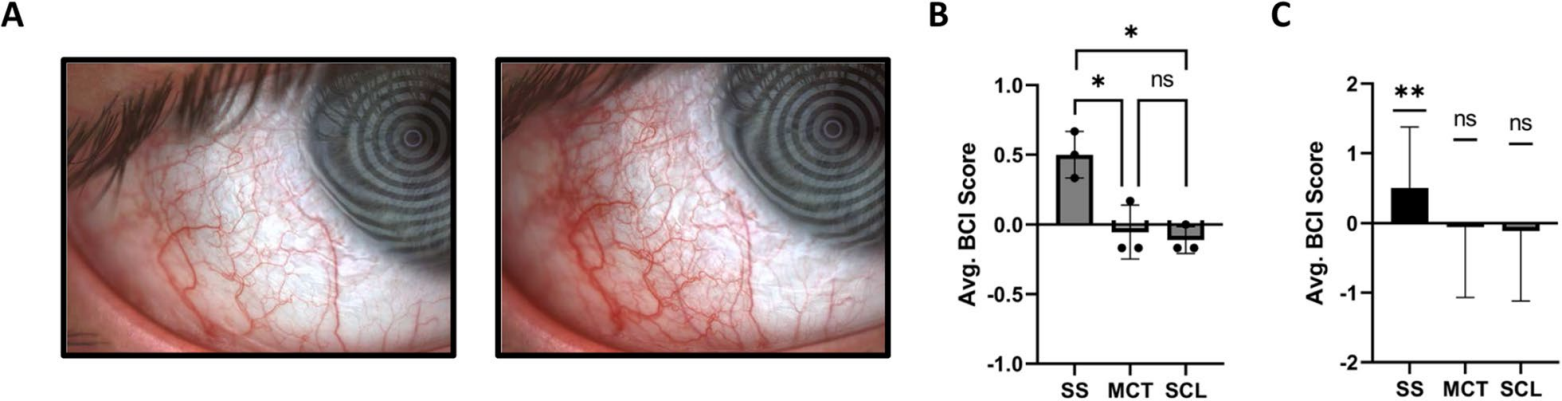
Objective bulbar conjunctival injection (BCI). **A** Photos demonstrating pre- (left) and post- (right) SS sampling, **B** Scoring of all pre- and post-sampling photos for all subjects and methods by three optometrists in a blinded pairwise comparison. BCI Score: Photo pairs scored with pre-sampling photos having greater BCI were counted as “-1”. Post-sampling photos scored with greater BCI were counted as “1”, **C** Average BCI score for individual sampling methods analyzed by one-sample t-tests. Error bars represent standard deviation, n = 12. * = p-value ≤ 0.05, ** = p-value ≤ 0.01

Ocular irritation could be a potential source for a greater sample volume in SS tear sampling. Importantly, eye irritation from tear sampling can alter the proteomic profile and increase subject reluctance for future studies [13, 14]. Hence, we assessed conjunctivitis, a known sign of ocular irritation [15]. Objective indications of conjunctivitis were determined for each subject by comparing bulbar conjunctival injection (BCI) before and after each tear film sampling method (Fig. 3A). Three optometrists performed a blinded, pairwise comparison of BCI from pre- and post-sampling photos. As shown in Fig. 3B, ocular surface irritation was significantly higher post-SS sampling compared to MCT or SCL. We conclude that differences in pre- and post-sampling BCI for SS are significant and demonstrate irritation from sampling. In contrast, differences for MCTs or SCLs are indistinguishable and thus do not induce ocular irritation, as observed by BCI (Fig. 3C).

**Fig. 4:**
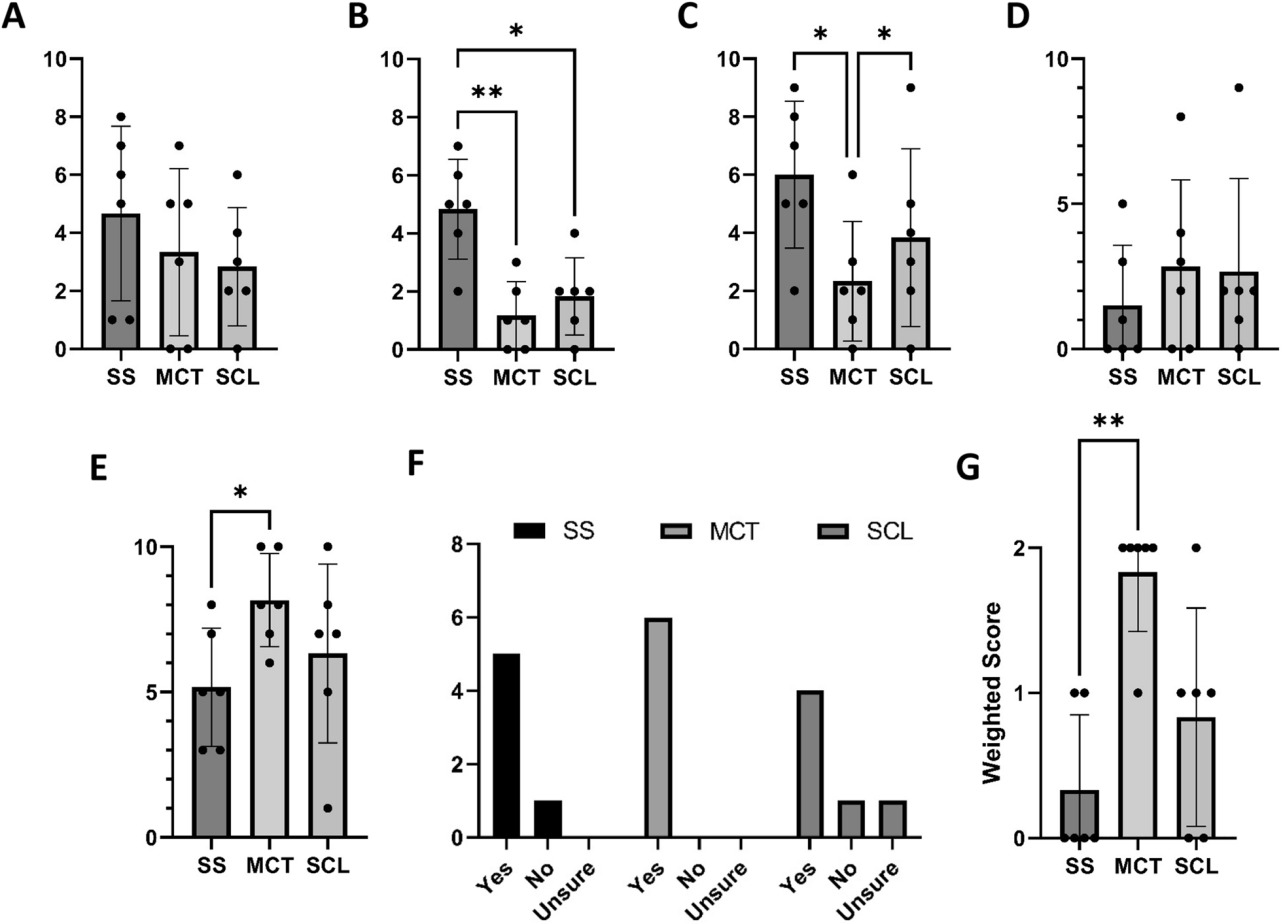
Subject tear sampling questionnaire responses. **A** Anxiety pre-sampling, **B** Anxiety post-sampling, **C** Discomfort during sampling, **D** Difficulty of sampling, **E** Willingness to repeat the sampling method if it could provide useful health information, **F** Overall experience, **G** Rank order of preferred tear sampling method. All questionnaires were graded on a scale of 0 = none, to 10 = extreme, except for a willingness to repeat (graded “yes,” “no,” or “unsure”) and overall experience (graded 0 = terrible, to 10 = excellent). Error bars represent standard deviation, n = 6. * = p-value ≤ 0.05, ** = p-value ≤ 0.01

Subject cooperation is required for effective sampling. Hence, we sought to understand the subject’s perspective on each sampling method. In our small study, subject questionnaire responses revealed anxiety about all methods (Fig. 4A). Post sampling, subjects viewed MCT and SCL with less anxiety than SS (Fig. 4B). On average, subjects reported the lowest level of discomfort with MCT (Fig. 4C). The subject’s assessment of tear sampling difficulty was also queried. Responses were varied; there was no statistically significant difference between methods (Fig. 4D). Finally, subjects were asked to rate their overall experience with each method numerically. Subjects reported the best experience with MCT and the worst with SS (Fig. 4E).

Tear film biomarker discovery or diagnostic screening requires subject/patient acceptance of tear sample collection. Most subjects responded positively when asked if they would be willing to repeat each tear sampling method if it could provide useful eye health information. One subject reported that they would refuse SS due to discomfort, 1 subject (who had not previously worn contact lenses) reported that they would refuse SCL due to perceived sampling difficulty, and 1 subject who reported “unsure” further explained that they would prefer the SCL method if they could perform SCL insertion and removal themselves (Fig. 4F). Most subjects reported the best experience with MCT and were willing to repeat any method if it could provide useful ocular health information.

After the study, subjects were asked to compare tear sampling methods and rank them by preference. Subjects ranked MCT as the most preferred, followed by SCL, with SS as the least preferred method (Fig. 4G). Subjects further commented that they perceived MCT as the fastest, least invasive, most comfortable, and easiest method. Two of the three subjects who were SCL wearers commented that they would choose SCL over MCT if they could insert and remove the SCLs themselves.

**Fig. 5:**
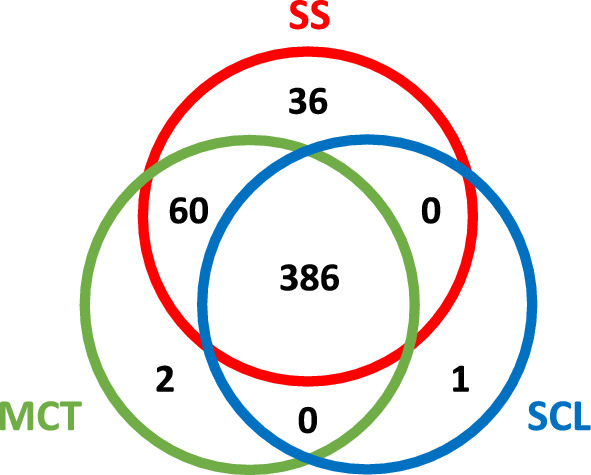
Total protein identifications by MS for tear sampling methods. SS = red, MCT = green, SCL = blue

Next, we assessed the identifiable proteins present in tear samples for each sampling method. Using MS proteomics, we identified 482, 448, and 387 total proteins out of the SS, MCT, and SCL subject samples, respectively (Fig. 5, Additional file 1: Table S1, Additional file 1: Figure S3 and S4)﻿. The majority (386 proteins) were shared between all methods. Furthermore, 36 proteins were unique to SSs, while MCTs and SCLs had only 2 and 1 unique proteins, respectively. Additionally, 60 proteins were shared between SS and MCT with no other shared proteins between sampling methods.

Intra-subject analysis reveals that none of our subjects had statistically significant quantitative changes between MCTs and SCLs, half had significant changes between SSs and SCLs, and most subjects had significant changes between SSs and MCTs (Table 1). Functional enrichment analysis of unique and shared proteins between all methods was analyzed using the STRING database. Our queries did not yield statistically significant results for groups of unique or shared protein species found across the different tear sampling methods.

**Table 1:** Intrasubject tear protein variation between sampling methods

Subject	p-value		
	SS/MCT	SS/SCL	MCT/SCL
1	NS	NS	NS
2	0.010	0.021	NS
3	0.007	NS	NS
4	0.005	0.012	NS
5	0.011	NS	NS
6	0.003	0.025	NS

NS = not significant

We assessed identified proteins for previously proposed reflex tear proteins and found putative markers in all our subjects for all tear sampling methods (Additional file 1: Table S2). Our proteomics data showed protein changes were consistently significant in both TMT 10-plexes only when comparing MCT and SCL sampled tears. Specifically, zymogen granule protein 16 homolog B (ZG16B) and lactotransferrin (TRFL) were increased in MCT sampled tears, and lysozyme C (LYSC) was increased with SCL sampled tears.

## Discussion

All tear sampling methods involve foreign bodies touching the ocular surface. Notably, foreign bodies often cause irritation, risk of injury, and the potential confounding effects of reflex tearing [23, 24]. Abrasive cellulose SSs or sharp-edged glass/plastic MCTs can irritate the eye and induce reflex tearing [25]. SS are so irritating that topical anesthesia is commonly used with SS in dry eye testing to reduce reflex epiphora [26]. However, anesthesia is a confounding variable and not always an appropriate solution to eye irritation. MCT sampling takes significant dexterity to perform without irritating the ocular surface and should be performed by a specialist with access to a slit lamp [25]. We recommend that MCT sampling not be performed by people with hand tremors or poor depth perception to protect subjects from injury. Furthermore, subjects who cannot sit still due to age or disease are also advised against MCT sampling for safety reasons. Thus, improved tear sampling methods are needed.

Given that SCLs are designed for optimal comfort and FDA approved for safety, we developed a tear sampling method using SCLs and assessed its potential for tear protein analysis. Not surprisingly, SCLs show significantly less ocular surface irritation than SS since SCLs are designed for the comfort and safety of the eye. Like SCLs, our BCI data shows MCT irritates the eye less than SS. Objectively, neither SCL nor MCT sampling show clinical signs of irritation. However, it is important to note that for MCT and SCL sampling, irritation, reflex tearing, and subject preference may depend upon the skill of the person carrying out the sample collection rather than the method itself.

We also assessed subjective experiences between sampling methods and observed that MCT was the most preferred tear sampling method. However, this data conflicts with previous reports, including a recent study in which MCT was ranked the least comfortable of four methods, including SS [16, 27]. A possible explanation for this discrepancy was that in our study, an optometrist trained in MCT sampling collected tears using a slit lamp. Not all researchers use slit lamp microscopy for MCT sampling; its absence can affect the subject’s experience and increase the risk of injury and discomfort. It is also important to note that MCT samples are more difficult to collect from low tear volume dry eye disease (DED) subjects, a condition which increases linearly with age [28]. In this study, all subjects were young and had a healthy tear volume before sampling [29]. Hence, while subjects here responded favorably to MCT sampling, an older subject population, sampling without a slit lamp, and/or less practiced hands carrying out the sample collection could yield a different result.

Depending on the subject demographic and study design, each tear sampling method may have advantages over other methods. For example, SCLs may be preferred for researchers or subjects with previous SCL experience. To our knowledge, SCL sampling is also the only known method where both researcher sampling and subject self-sampling are reasonable options. Notably, the possibility for self-sampling creates the potential for at-home tear sampling. This approach allows subjects to insert the SCLs in the morning before their eye exam to collect more protein throughout the day. This strategy could make SCLs the tear sampling method of choice for low abundance biomarkers. In-office tear sampling at routine eye exams is also a feasible option.

To analyze proteins collected by the different sampling methods, we used MS proteomics, the method typically applied for tear biomarker identification [9]. Our proteomic analysis confirmed that all tear sampling methods capture a common set of proteins representing most of the proteins sampled by each method. Consistent with previous reports, SS sampling returned higher protein identifications than MCT [8, 9]. To rule out the possibility that this difference simply reflects the observed larger sample volumes and thus higher total protein, our MS analysis normalized for total protein concentration between methods. Notably, even when corrected for differences in protein yield, the MS data still showed a significant subset of proteins unique to SS. Previous studies comparing SS and MCT proteomics show similar subsets unique to SS, including a label-free experiment reported by Nättinen et al. where 850 proteins were identified, 80 were unique to SS, 9 were unique to MCT, and 761 were shared [8, 30]. In their study, the total sampled protein was not normalized between methods (Avg MCT: 19.7 µg, Avg SS: 199 µg).

It has been proposed that SS sample reflex tears and MCT sample basal tears [8, 16]. However, the characterization of different tear types has not been well-established on a proteomic level. Significantly, variables and nuances in the tear sampling process can affect the tear type, regardless of the sampling method. For example, an anxious subject may flinch during MCT sampling, injure or irritate the eye, and elicit reflex tearing. Another subject more comfortable with eye touch may sit still and provide better basal tear samples. Thus, different tear types may be obtained despite using the same tear sampling method.

Our ocular BCI findings support previous reports that SS irritates the ocular surface [7] and reportedly stimulates reflex tearing [25, 31]. Our data further suggests that unique SS proteins may be linked to irritation and ocular surface damage, a hypothesis made previously [32]. However, we also observed a set of 60 proteins shared by SS and MCT that were not detected by SCL sampling. While SCLs may not have captured this subset of proteins, it is possible that biochemical processes associated with reflex tearing might be stimulated with MCT sampling, even without visible signs of irritation. It is also important to consider that given the number and type of variables involved in tear sampling, basal, and reflex tears may be considered as ends of a spectrum rather than distinct qualitative classifications. Further studies are needed to investigate these differences.

Since few studies have assessed proteomic differences between reflex and basal tears, classifying tears based on proteomic differences is difficult [13, 14, 25]. Nevertheless, we evaluated our respective tear sampling method proteomes for proteins previously reported to be associated with reflex tearing. As there is currently no reference proteome for reflex tears, we relied mainly on the recent report by Perumal et al. [14].

In their report, the authors suggest that ZG16B may be associated with neural stimulation of the lacrimal gland and exocytosis of secretory granules in reflex tearing [14]. Thus, the increases of ZG16B in MCT sampling when compared to SCLs suggests that MCTs may induce some unique aspect of irritation and subsequent reflex tearing. However, it is important to note that correlations of previously reported reflex tear markers to our study are indeterminate given small sample sizes and confounding variables. For example, In the Perumal et al. study, basal and reflex tears were collected using MCTs, and reflex tearing was stimulated using onion vapors. As reflex tearing has many different triggers [33], proteomic differences may exist depending on how reflex tearing is stimulated. Given these confounding variables, more research is needed to assess reflex tear stimulation subtypes and validate their associated proteomes. 

As expected, we observed elevated levels of lysozyme C (LYSC) in SCL sampling since ionic SCLs (such as etafilcon A) preferentially adsorb LYSC [17, 18]. However, lactoferrin (TRFL) was significantly reduced with SCL sampling. Differences in TRFL are possibly due to material specific interactions [19], though more research and larger sample sizes may be needed to understand these differences. Since TRFL accounts for over 25% of total tear proteins [20], a reduction in TRFL should reduce ion suppression in MS; a potential benefit of SCL sampling [21, 22]. Thus, our studies show SCLs sample basal tears and could aid in the discovery of low abundant biomarkers by reducing levels of highly abundant proteins.

Importantly, our intrasubject tear sampling method comparisons showed statistically significant proteomic differences when comparing SS to MCT or SCL for at least half of our subjects. There was no significant difference between MCT and SCL sampling for any of our subjects. Since SS and MCT are reported to sample reflex and basal tears [8, 16, 34] respectively, these proteomic findings support the idea that SCLs sample basal tears. Overall, our combined clinical and proteomic data support SCL sampling as an alternative to MCT for basal tear sampling. At the same time, unique protein species in SS sampling may indicate biochemical changes associated with reflex tearing. While contaminants such as keratins can be introduced during sample prep, they are also an important component of meibum [35] and are expected to be present in tears, given the proximity of host ocular adnexal skin. Thus, keratins should be considered a natural component of tears.

We hypothesize that SCLs may be advantageous for tear sampling for several other reasons. SCLs capture and concentrate tear proteins on the eye over time, not just at the moment of initial foreign body contact. Thus, hypothetically, wearing contacts for longer periods allows basal tear production to return and SCLs to capture basal tear proteins. Additionally, SCL sampling is particularly advantageous for dry eye disease subjects with low tear volumes since SCL sampling is largely volume independent. Finally, because patients can sample their own tears with SCLs, some patients may jerk or flinch less in the tear sampling process if they are in control of the situation, particularly if they are SCL wearers and are comfortable inserting and removing lenses on their own.

This study has several important limitations. First, our pilot study had a small sample size. Second, the researcher sampling tears had practiced MCT sampling in preparation for this study, which may have affected both proteomic and subject questionnaire results. Third, all subjects were under 30 years of age. Since tear volume decreases linearly with age, older subjects should be included in sampling assessments in future and more extensive studies. Fourth, not all sampling methodologies were included in this study. Methods such as the “flush” method and weck-cel sponges were not assessed [36]. Finally, adding saline may also facilitate ease of sampling for all methods [37], though studies are needed to understand these effects.

Our study highlights important considerations when selecting a tear sampling method. The desired tear type, effects on the subject, and available resources in both personnel and equipment are all significant factors. SS are easy to use but irritate the eye and likely induce reflex tearing. When performed by a dexterous researcher with access to a slit lamp, subject cooperation with MCT can be high. However, MCT may be inappropriate for DED subjects. SCLs are an easily implemented tear sampling method appropriate for DED subjects that do not require additional equipment. We conclude that SCLs are an accessible tear sampling method that may provide more pure basal tear samples relative to current methods.

### Supplementary Information

**Additional file1.  **Supplementary figures, tables, and methods. **Fig. S1.** Distribution of p-values obtained from the Shapiro-Wilk normality test for each protein, **Fig. S2.** Specifications of SCLs used, **Fig. S3.** Total protein identification comparison, **Fig. S4.** Volcano plots of protein quantification between tear sampling methods, **Table S1.** Proteins identified by MS for SS, MCT, and SCL Sampling, **Table S2.** Quantitative Changes in Selected Reflex Tear Proteins Between Sampling Methods, **Table S3.** Schirmer Strip Wet Length by Subject, **Table S4.** Supplemental Mass Spectrometry Data, **Table S5.** Mass Spectrometry Settings & Details, **Table S6.** TMT10-plex Label Assignments, **Table S7.** Contaminants Identified In Subject Tears Using the Common Repository of Adventitious Proteins (cRAP) database, **Table S8.** Median Protein Values for Individual Proteins for Individual 10-plexes, and Supplementary Methods S1.

## Data Availability

The datasets supporting the conclusions of this article are available in the Mendeley Data repository, https://data.mendeley.com/datasets/f4vggkmcdn/draft?a=82975819-b7d7-486a-9db2-76919a7a7cfb
