# Supplementary material for: Human tear film protein sampling using soft contact lenses
Source: Clin Proteomics. 2024 Mar 13;21:23. doi: 10.1186/s12014-024-09475-8 (PMC10936081; doi:10.1186/s12014-024-09475-8)
Supplement: Supplementary file 1 — Additional file1. Supplementary figures, tables, and methods. Fig. S1. Distribution of p-values obtained from the Shapiro-Wilk normality test for each protein, Fig. S2. Specifications of SCLs used, Fig. S3. Total protein identification comparison, Fig. S4. Volcano plots of protein quantification between tear sampling methods, Table S1. Proteins identified by MS for SS, MCT, and SCL Sampling, Table S2. Quantitative Changes in Selected Reflex Tear Proteins Between Sampling Methods, Table S3. Schirmer Strip Wet Length by Subject, Table S4. Supplemental Mass Spectrometry Data, Table S5. Mass Spectrometry Settings & Details, Table S6. TMT10-plex Label Assignments, Table S7. Contaminants Identified In Subject Tears Using the Common Repository of Adventitious Proteins (cRAP) database, Table S8. Median Protein Values for Individual Proteins for Individual 10-plexes, and Supplementary Methods S1. [file 12014_2024_9475_MOESM1_ESM.docx]

**Human Tear Film Protein Sampling Using Soft Contact Lenses**

Robert K. Roden ^a, b^, robkroden@gmail.com

Nathan Zuniga ^a^, natezu93@gmail.com

Joshua C. Wright ^a^, wright.c.josh@gmail.com

David H. Parkinson ^a^, davidhparkinson@gmail.com

Fangfang Jiang ^a^, fangfangjiang123@gmail.com

Leena M. Patil ^a^, leena2411@gmail.com

Rebecca S. Burlett ^a^, burlett.rebecca@gmail.com

Alyssa A. Nitz ^c^, alyssa@nitz.org

Joshua J. Rogers ^a^, joshuarogersbyu@gmail.com

Jarett T. Pittman ^a^, jarettpittman5@gmail.com

Kenneth L. Virgin ^a^, kenlvirgin@gmail.com

P. Christine Ackroyd ^a^, canoowidow@yahoo.com

Samuel H. Payne ^c^, sam_payne@byu.edu

John C. Price ^a^, jcprice@chem.byu.edu

Kenneth A. Christensen ^a^, kenc@chem.byu.edu

^a^ Department of Chemistry & Biochemistry, Brigham Young University, Provo, UT, 84602, USA

^b^ College of Optometry, Rocky Mountain University of Health Professions, Provo, UT, 84606, USA

^c^ Department of Biology, Brigham Young University, Provo, UT, 84602, USA

**Supplementary Information**

**Fig. S1**





**Distribution of p-values obtained from the Shapiro-Wilk normality test for each protein.** Before paired tests were performed on the transformed abundance data, normality of the data within each paired test was assessed using the Shapiro-Wilk test for normality. The red line demarcates a p-value > 0.05, indicating that the data were normally distributed. Since 61.3% of the datasets were normally distributed, paired t-test were performed for each protein.

**Fig. S2**


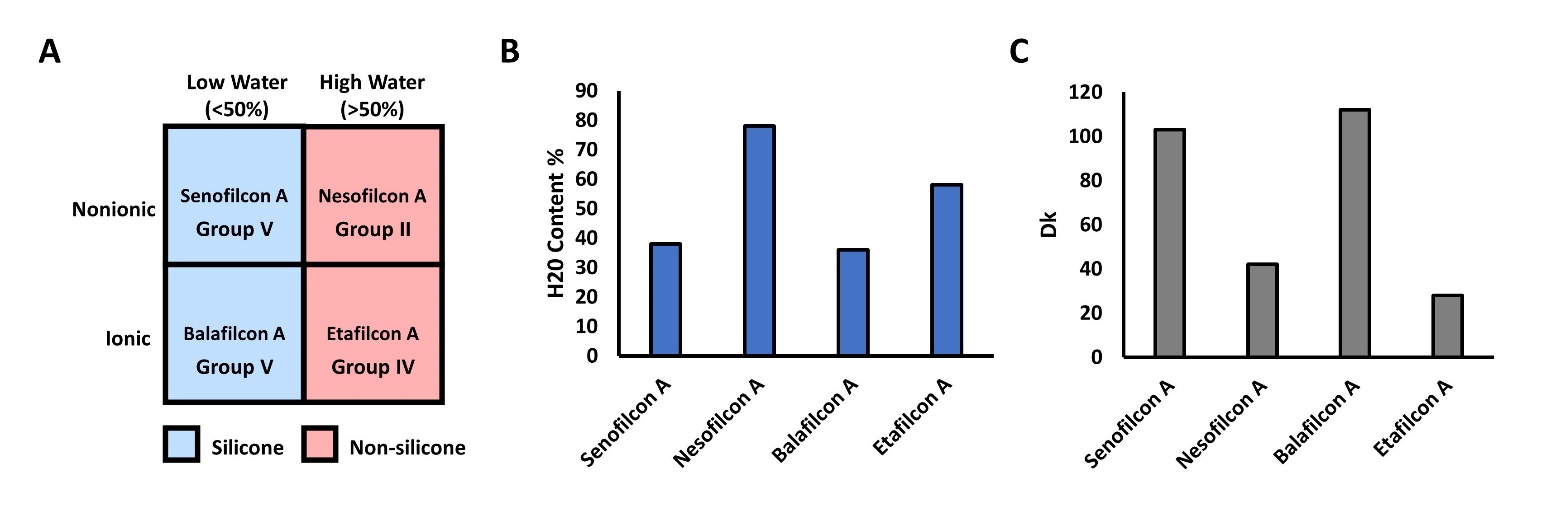


**Specifications of SCLs used.** A) Hydrogel polymers by FDA grouping, B) Water content by polymer, C) Oxygen transmissibility (Dk) by polymer. Data is shown as reported by contact lens manufacturers

**Fig. S3**

**
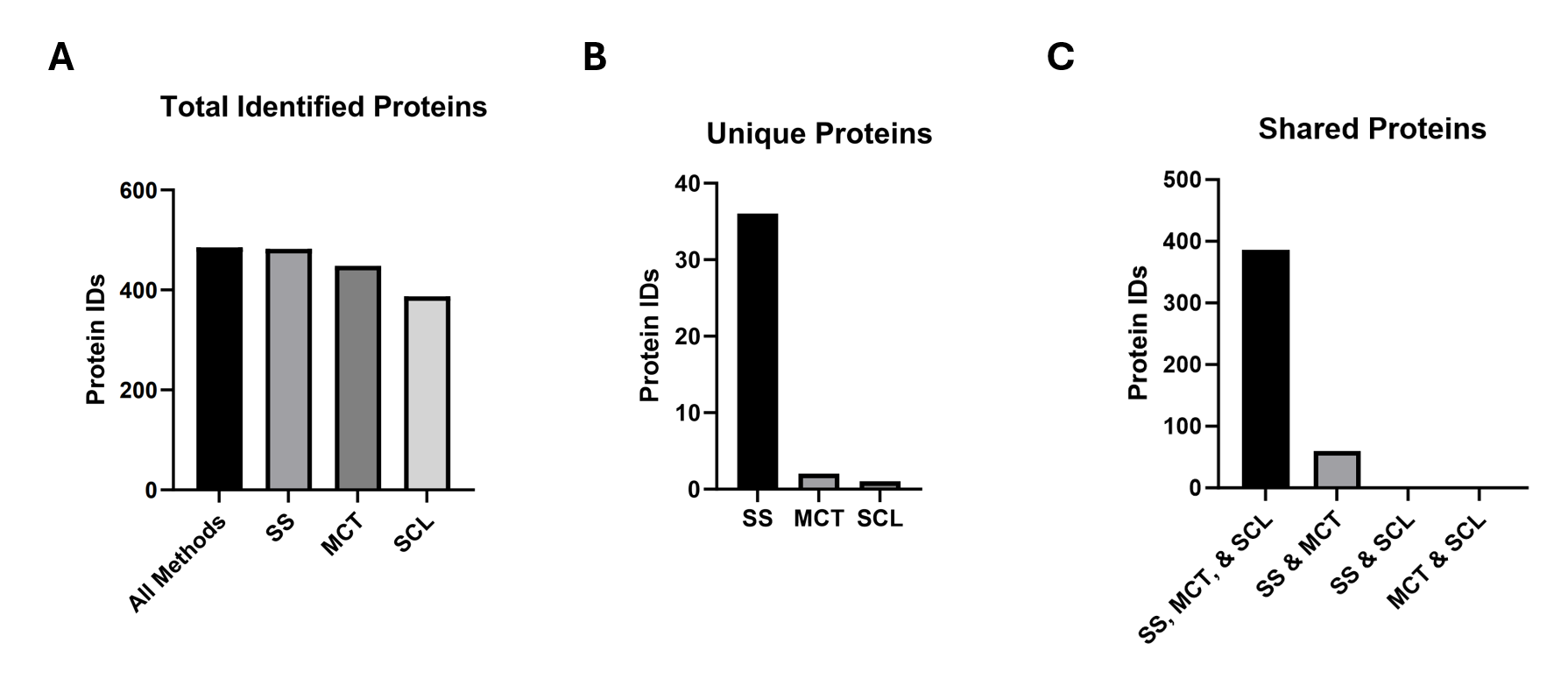
**

**Total protein identification comparison.** A. Total identified proteins between all methods, B. Unique proteins to each method, C. Shared proteins between each method.

**Fig. S4**


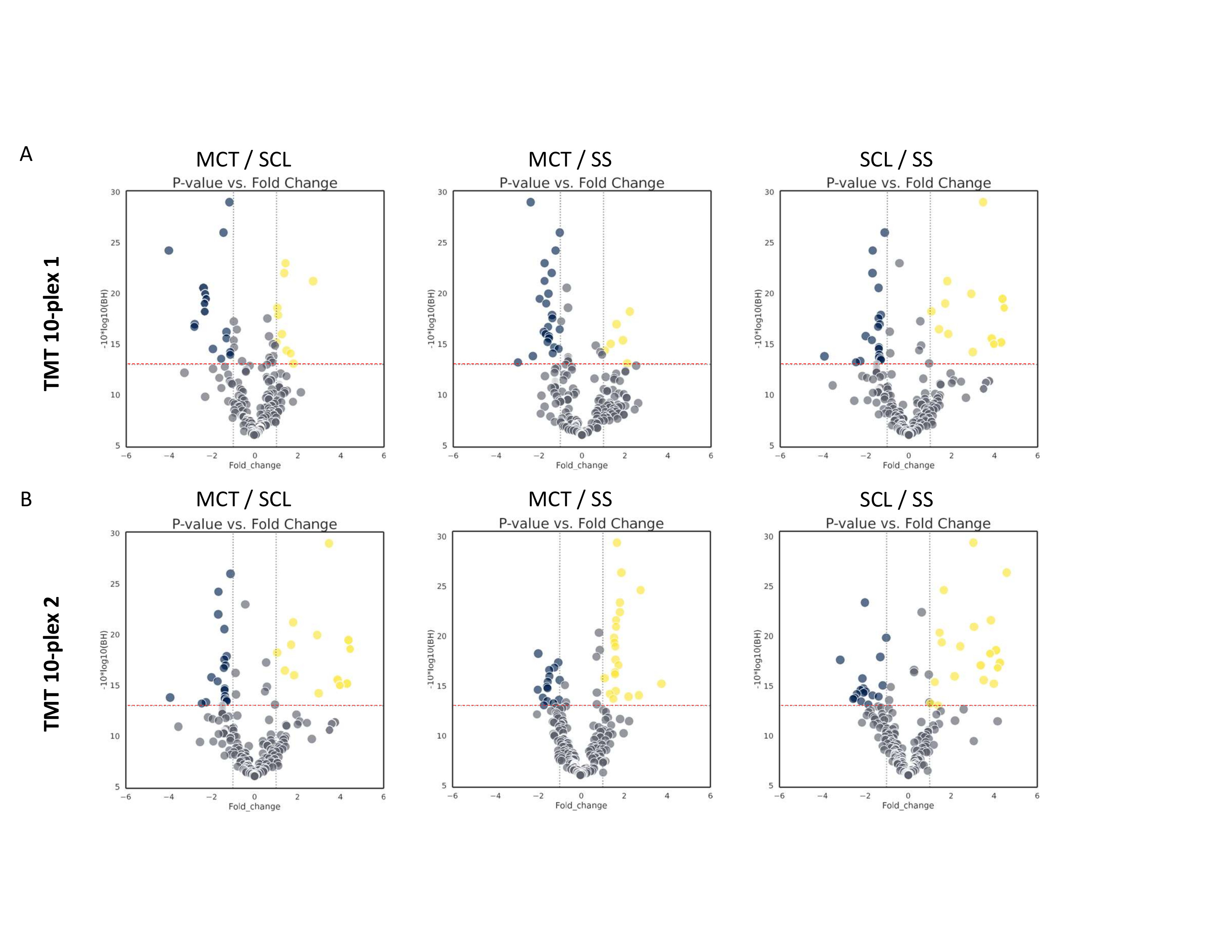


**Volcano plots of protein quantification between tear sampling methods.** Tear sampling methods were compared using log2 transformed protein abundance data in paired t-tests. The horizontal red dotted line represents an adjusted p-value of 0.05.

**Table S1.** Proteins identified by MS for SS, MCT, and SCL Sampling

| Total Identified Proteins | | | | | | | | | | | | | | | | | | | | | |
| --- | --- | --- | --- | --- | --- | --- | --- | --- | --- | --- | --- | --- | --- | --- | --- | --- | --- | --- | --- | --- | --- |
| All Methods | | | | | | SS | | | | | | MCT | | | | | | | SCL | | |
| Total IDs | | | 485 | | | Total IDs | | | 482 | | | Total IDs | | | | | 448 | | Total IDs | | 387 |
| Uniprot | | | Protein ID | | | Uniprot | | | Protein ID | | | Uniprot | | | | | Protein ID | | Uniprot | | Protein ID |
| P02788 | | | TRFL | | | P02788 | | | TRFL | | | P02788 | | | | | TRFL | | P02788 | | TRFL |
| P02768 | | | ALBU | | | P02768 | | | ALBU | | | P02768 | | | | | ALBU | | P02768 | | ALBU |
| P31025 | | | LCN1 | | | P31025 | | | LCN1 | | | P31025 | | | | | LCN1 | | P31025 | | LCN1 |
| P98160 | | | PGBM | | | P98160 | | | PGBM | | | P98160 | | | | | PGBM | | P98160 | | PGBM |
| P01833 | | | PIGR | | | P01833 | | | PIGR | | | P01833 | | | | | PIGR | | P01833 | | PIGR |
| P01024 | | | CO3 | | | P01024 | | | CO3 | | | P01024 | | | | | CO3 | | P01024 | | CO3 |
| P01876 | | | *IGHA1 | | | P01876 | | | *IGHA1 | | | P01876 | | | | | *IGHA1 | | P01876 | | *IGHA1 |
| P61626 | | | LYSC | | | P61626 | | | LYSC | | | P61626 | | | | | LYSC | | P61626 | | LYSC |
| P25311 | | | ZA2G | | | P25311 | | | ZA2G | | | P25311 | | | | | ZA2G | | P25311 | | ZA2G |
| P0DOX7 | | | IGK | | | P0DOX7 | | | IGK | | | P0DOX7 | | | | | IGK | | P0DOX7 | | IGK |
| P04264 | | | K2C1 | | | P04264 | | | K2C1 | | | P04264 | | | | | K2C1 | | P04264 | | K2C1 |
| Q5VSP4 | | | LC1L1 | | | Q5VSP4 | | | LC1L1 | | | Q5VSP4 | | | | | LC1L1 | | P02787 | | TRFE |
| P02787 | | | TRFE | | | P02787 | | | TRFE | | | P02787 | | | | | TRFE | | P14618 | | KPYM |
| P14618 | | | KPYM | | | P14618 | | | KPYM | | | P14618 | | | | | KPYM | | P06733 | | ENOA |
| P0DOX2 | | | *IGA2 | | | P0DOX2 | | | *IGA2 | | | P0DOX2 | | | | | *IGA2 | | P19013 | | K2C4 |
| P06733 | | | ENOA | | | P06733 | | | ENOA | | | P06733 | | | | | ENOA | | P01036 | | CYTS |
| P19013 | | | K2C4 | | | P19013 | | | K2C4 | | | P19013 | | | | | K2C4 | | P21980 | | TGM2 |
| P01036 | | | CYTS | | | P01036 | | | CYTS | | | P01036 | | | | | CYTS | | Q9UGM3 | | DMBT1 |
| P21980 | | | TGM2 | | | P21980 | | | TGM2 | | | P21980 | | | | | TGM2 | | P01037 | | CYTN |
| Q9UGM3 | | | DMBT1 | | | Q9UGM3 | | | DMBT1 | | | Q9UGM3 | | | | | DMBT1 | | P06396 | | GELS |
| P01037 | | | CYTN | | | P01037 | | | CYTN | | | P01037 | | | | | CYTN | | P13647 | | K2C5 |
| P06396 | | | GELS | | | P06396 | | | GELS | | | P06396 | | | | | GELS | | P10909 | | CLUS |
| P13647 | | | K2C5 | | | P13647 | | | K2C5 | | | P13647 | | | | | K2C5 | | P13645 | | K1C10 |
| P10909 | | | CLUS | | | P10909 | | | CLUS | | | P10909 | | | | | CLUS | | P98088 | | MUC5A |
| P13645 | | | K1C10 | | | P13645 | | | K1C10 | | | P13645 | | | | | K1C10 | | Q13421 | | MSLN |
| P98088 | | | MUC5A | | | P98088 | | | MUC5A | | | P98088 | | | | | MUC5A | | P01009 | | A1AT |
| Q13421 | | | MSLN | | | Q13421 | | | MSLN | | | Q13421 | | | | | MSLN | | P60709 | | ACTB |
| P01009 | | | A1AT | | | P01009 | | | A1AT | | | P01009 | | | | | A1AT | | P04083 | | ANXA1 |
| P60709 | | | ACTB | | | P60709 | | | ACTB | | | P60709 | | | | | ACTB | | P02647 | | APOA1 |
| P04083 | | | ANXA1 | | | P04083 | | | ANXA1 | | | P04083 | | | | | ANXA1 | | P02538 | | K2C6A |
| P02647 | | | APOA1 | | | P02647 | | | APOA1 | | | P02647 | | | | | APOA1 | | P35527 | | K1C9 |
| P02538 | | | K2C6A | | | P02538 | | | K2C6A | | | P02538 | | | | | K2C6A | | P08727 | | K1C19 |
| P35527 | | | K1C9 | | | P35527 | | | K1C9 | | | P35527 | | | | | K1C9 | | P00738 | | HPT |
| P08727 | | | K1C19 | | | P08727 | | | K1C19 | | | P08727 | | | | | K1C19 | | P07355 | | ANXA2 |
| P00738 | | | HPT | | | P00738 | | | HPT | | | P00738 | | | | | HPT | | P00352 | | AL1A1 |
| P07355 | | | ANXA2 | | | P07355 | | | ANXA2 | | | P07355 | | | | | ANXA2 | | Q16378 | | PROL4 |
| P00352 | | | AL1A1 | | | P00352 | | | AL1A1 | | | P00352 | | | | | AL1A1 | | P35908 | | K22E |
| Q16378 | | | PROL4 | | | Q16378 | | | PROL4 | | | Q16378 | | | | | PROL4 | | P0DOX5 | | *IGG1 |
| P35908 | | | K22E | | | P35908 | | | K22E | | | P35908 | | | | | K22E | | P01871 | | *IGHM |
| P0DOX5 | | | *IGG1 | | | P0DOX5 | | | *IGG1 | | | P0DOX5 | | | | | *IGG1 | | P00450 | | CERU |
| P01871 | | | *IGHM | | | P01871 | | | *IGHM | | | P01871 | | | | | *IGHM | | Q9GZZ8 | | LACRT |
| P00450 | | | CERU | | | P00450 | | | CERU | | | P00450 | | | | | CERU | | P0DMV8 | | HS71A |
| Q9GZZ8 | | | LACRT | | | Q9GZZ8 | | | LACRT | | | Q9GZZ8 | | | | | LACRT | | P0DMV9 | | HS71B |
| P0DMV8 | | | HS71A | | | P0DMV8 | | | HS71A | | | P0DMV8 | | | | | HS71A | | P13646 | | K1C13 |
| P0DMV9 | | | HS71B | | | P0DMV9 | | | HS71B | | | P0DMV9 | | | | | HS71B | | P06702 | | S10A9 |
| P13646 | | | K1C13 | | | P13646 | | | K1C13 | | | P13646 | | | | | K1C13 | | P30740 | | ILEU |
| P09228 | | | CYTT | | | P09228 | | | CYTT | | | P09228 | | | | | CYTT | | P60174 | | TPIS |
| P06702 | | | S10A9 | | | P06702 | | | S10A9 | | | P06702 | | | | | S10A9 | | P08729 | | K2C7 |
| P30740 | | | ILEU | | | P30740 | | | ILEU | | | P30740 | | | | | ILEU | | P12273 | | PIP |
| P60174 | | | TPIS | | | P60174 | | | TPIS | | | P60174 | | | | | TPIS | | P04259 | | K2C6B |
| P08729 | | | K2C7 | | | P08729 | | | K2C7 | | | P08729 | | | | | K2C7 | | P68104 | | EF1A1 |
| P12273 | | | PIP | | | P12273 | | | PIP | | | P12273 | | | | | PIP | | P80303 | | NUCB2 |
| P04259 | | | K2C6B | | | P04259 | | | K2C6B | | | P04259 | | | | | K2C6B | | P04406 | | G3P |
| P01861 | | | *IGHG4 | | | P01861 | | | *IGHG4 | | | P01861 | | | | | *IGHG4 | | P00558 | | PGK1 |
| P01859 | | | *IGHG2 | | | P01859 | | | *IGHG2 | | | P01859 | | | | | *IGHG2 | | B9A064 | | *IGLL5 |
| P68104 | | | EF1A1 | | | P68104 | | | EF1A1 | | | P68104 | | | | | EF1A1 | | O75556 | | SG2A1 |
| P80303 | | | NUCB2 | | | P80303 | | | NUCB2 | | | P80303 | | | | | NUCB2 | | P04792 | | HSPB1 |
| P04406 | | | G3P | | | P04406 | | | G3P | | | P04406 | | | | | G3P | | P01023 | | A2MG |
| P00558 | | | PGK1 | | | P00558 | | | PGK1 | | | P00558 | | | | | PGK1 | | P02533 | | K1C14 |
| B9A064 | | | *IGLL5 | | | B9A064 | | | *IGLL5 | | | B9A064 | | | | | *IGLL5 | | P09211 | | GSTP1 |
| O75556 | | | SG2A1 | | | O75556 | | | SG2A1 | | | O75556 | | | | | SG2A1 | | P30044 | | PRDX5 |
| P04792 | | | HSPB1 | | | P04792 | | | HSPB1 | | | P04792 | | | | | HSPB1 | | P30086 | | PEBP1 |
| P01023 | | | A2MG | | | P01023 | | | A2MG | | | P01023 | | | | | A2MG | | P04075 | | ALDOA |
| P02533 | | | K1C14 | | | P02533 | | | K1C14 | | | P02533 | | | | | K1C14 | | P05787 | | K2C8 |
| P09211 | | | GSTP1 | | | P09211 | | | GSTP1 | | | P09211 | | | | | GSTP1 | | P02675 | | FIBB |
| P30044 | | | PRDX5 | | | P30044 | | | PRDX5 | | | P30044 | | | | | PRDX5 | | P02790 | | HEMO |
| P30086 | | | PEBP1 | | | P30086 | | | PEBP1 | | | P30086 | | | | | PEBP1 | | P02679 | | FIBG |
| P04075 | | | ALDOA | | | P04075 | | | ALDOA | | | P04075 | | | | | ALDOA | | Q08380 | | LG3BP |
| P05787 | | | K2C8 | | | P05787 | | | K2C8 | | | P05787 | | | | | K2C8 | | P11142 | | HSP7C |
| P02675 | | | FIBB | | | P02675 | | | FIBB | | | P02675 | | | | | FIBB | | P02774 | | VTDB |
| P02790 | | | HEMO | | | P02790 | | | HEMO | | | P02790 | | | | | HEMO | | O95968 | | SG1D1 |
| P02679 | | | FIBG | | | P02679 | | | FIBG | | | P02679 | | | | | FIBG | | P07900 | | HS90A |
| Q08380 | | | LG3BP | | | Q08380 | | | LG3BP | | | Q08380 | | | | | LG3BP | | P07858 | | CATB |
| P11142 | | | HSP7C | | | P11142 | | | HSP7C | | | P11142 | | | | | HSP7C | | Q06830 | | PRDX1 |
| P02774 | | | VTDB | | | P02774 | | | VTDB | | | P02774 | | | | | VTDB | | Q8N474 | | SFRP1 |
| O95968 | | | SG1D1 | | | O95968 | | | SG1D1 | | | O95968 | | | | | SG1D1 | | P07602 | | SAP |
| P07900 | | | HS90A | | | P07900 | | | HS90A | | | P07900 | | | | | HS90A | | P05109 | | S10A8 |
| P07858 | | | CATB | | | P07858 | | | CATB | | | P07858 | | | | | CATB | | Q13228 | | SBP1 |
| Q06830 | | | PRDX1 | | | Q06830 | | | PRDX1 | | | Q06830 | | | | | PRDX1 | | Q7Z406 | | MYH14 |
| Q8N474 | | | SFRP1 | | | Q8N474 | | | SFRP1 | | | Q8N474 | | | | | SFRP1 | | P31946 | | 1433B |
| P07602 | | | SAP | | | P07602 | | | SAP | | | P07602 | | | | | SAP | | P15311 | | EZRI |
| P05109 | | | S10A8 | | | P05109 | | | S10A8 | | | P05109 | | | | | S10A8 | | P80188 | | NGAL |
| Q13228 | | | SBP1 | | | Q13228 | | | SBP1 | | | Q13228 | | | | | SBP1 | | P01034 | | CYTC |
| Q7Z406 | | | MYH14 | | | Q7Z406 | | | MYH14 | | | Q7Z406 | | | | | MYH14 | | P63104 | | 1433Z |
| P31946 | | | 1433B | | | P31946 | | | 1433B | | | P31946 | | | | | 1433B | | Q9BQE3 | | TBA1C |
| P15311 | | | EZRI | | | P15311 | | | EZRI | | | P15311 | | | | | EZRI | | Q71U36 | | TBA1A |
| P80188 | | | NGAL | | | P80188 | | | NGAL | | | P80188 | | | | | NGAL | | P68363 | | TBA1B |
| P01034 | | | CYTC | | | P01034 | | | CYTC | | | P01034 | | | | | CYTC | | P16403 | | H12 |
| P63104 | | | 1433Z | | | P63104 | | | 1433Z | | | P63104 | | | | | 1433Z | | Q14515 | | SPRL1 |
| Q9BQE3 | | | TBA1C | | | Q9BQE3 | | | TBA1C | | | Q9BQE3 | | | | | TBA1C | | P00966 | | ASSY |
| Q71U36 | | | TBA1A | | | Q71U36 | | | TBA1A | | | Q71U36 | | | | | TBA1A | | Q99935 | | PROL1 |
| P68363 | | | TBA1B | | | P68363 | | | TBA1B | | | P68363 | | | | | TBA1B | | P37802 | | TAGL2 |
| P16403 | | | H12 | | | P16403 | | | H12 | | | P16403 | | | | | H12 | | Q96DA0 | | ZG16B |
| Q14515 | | | SPRL1 | | | Q14515 | | | SPRL1 | | | Q14515 | | | | | SPRL1 | | P30041 | | PRDX6 |
| P00966 | | | ASSY | | | P00966 | | | ASSY | | | P00966 | | | | | ASSY | | P11021 | | BIP |
| Q99935 | | | PROL1 | | | Q99935 | | | PROL1 | | | Q99935 | | | | | PROL1 | | P23528 | | COF1 |
| P08238 | | | HS90B | | | P08238 | | | HS90B | | | P37802 | | | | | TAGL2 | | P22079 | | PERL |
| P37802 | | | TAGL2 | | | P37802 | | | TAGL2 | | | Q96DA0 | | | | | ZG16B | | P01011 | | AACT |
| Q96DA0 | | | ZG16B | | | Q96DA0 | | | ZG16B | | | P30041 | | | | | PRDX6 | | P06727 | | APOA4 |
| P30041 | | | PRDX6 | | | P30041 | | | PRDX6 | | | P11021 | | | | | BIP | | P62937 | | PPIA |
| P11021 | | | BIP | | | P11021 | | | BIP | | | P23528 | | | | | COF1 | | P02763 | | A1AG1 |
| P23528 | | | COF1 | | | P23528 | | | COF1 | | | P22079 | | | | | PERL | | O43852 | | CALU |
| P22079 | | | PERL | | | P22079 | | | PERL | | | P01011 | | | | | AACT | | Q6MZM9 | | PRR27 |
| P01011 | | | AACT | | | P01011 | | | AACT | | | P06727 | | | | | APOA4 | | P02652 | | APOA2 |
| P06727 | | | APOA4 | | | P06727 | | | APOA4 | | | P62937 | | | | | PPIA | | Q96KP4 | | CNDP2 |
| P62937 | | | PPIA | | | P62937 | | | PPIA | | | P02763 | | | | | A1AG1 | | P30838 | | AL3A1 |
| P02763 | | | A1AG1 | | | P02763 | | | A1AG1 | | | O43852 | | | | | CALU | | P02671 | | FIBA |
| O43852 | | | CALU | | | O43852 | | | CALU | | | Q6MZM9 | | | | | PRR27 | | P14555 | | PA2GA |
| Q6MZM9 | | | PRR27 | | | Q6MZM9 | | | PRR27 | | | P02652 | | | | | APOA2 | | P32119 | | PRDX2 |
| P02652 | | | APOA2 | | | P02652 | | | APOA2 | | | P68032 | | | | | ACTC | | P07737 | | PROF1 |
| P68032 | | | ACTC | | | P68032 | | | ACTC | | | P62736 | | | | | ACTA | | P20061 | | TCO1 |
| P62736 | | | ACTA | | | P62736 | | | ACTA | | | P63267 | | | | | ACTH | | Q01518 | | CAP1 |
| P63267 | | | ACTH | | | P63267 | | | ACTH | | | P68133 | | | | | ACTS | | P01780 | | HV307 |
| P68133 | | | ACTS | | | P68133 | | | ACTS | | | Q96KP4 | | | | | CNDP2 | | P01591 | | *IGJ |
| Q96KP4 | | | CNDP2 | | | Q96KP4 | | | CNDP2 | | | P30838 | | | | | AL3A1 | | P03973 | | SLPI |
| P30838 | | | AL3A1 | | | P30838 | | | AL3A1 | | | P02671 | | | | | FIBA | | P01619 | | KV320 |
| P02671 | | | FIBA | | | P02671 | | | FIBA | | | P14555 | | | | | PA2GA | | P30085 | | KCY |
| P14555 | | | PA2GA | | | P14555 | | | PA2GA | | | P32119 | | | | | PRDX2 | | P61769 | | B2MG |
| P32119 | | | PRDX2 | | | P32119 | | | PRDX2 | | | P07737 | | | | | PROF1 | | P60660 | | MYL6 |
| P07737 | | | PROF1 | | | P07737 | | | PROF1 | | | P20061 | | | | | TCO1 | | Q99880 | | H2B1L |
| P20061 | | | TCO1 | | | P20061 | | | TCO1 | | | Q01518 | | | | | CAP1 | | Q99879 | | H2B1M |
| Q01518 | | | CAP1 | | | Q01518 | | | CAP1 | | | P01780 | | | | | HV307 | | Q93079 | | H2B1H |
| P01780 | | | HV307 | | | P01780 | | | HV307 | | | P01591 | | | | | *IGJ | | Q5QNW6 | | H2B2F |
| P01591 | | | *IGJ | | | P01591 | | | *IGJ | | | P03973 | | | | | SLPI | | Q99877 | | H2B1N |
| P03973 | | | SLPI | | | P03973 | | | SLPI | | | P01619 | | | | | KV320 | | O60814 | | H2B1K |
| P35579 | | | MYH9 | | | P35579 | | | MYH9 | | | P0DP24 | | | | | CALM2 | | P62807 | | H2B1C |
| P01619 | | | KV320 | | | P01619 | | | KV320 | | | P0DP23 | | | | | CALM1 | | P58876 | | H2B1D |
| P0DP24 | | | CALM2 | | | P0DP24 | | | CALM2 | | | P0DP25 | | | | | CALM3 | | P57053 | | H2BFS |
| P0DP23 | | | CALM1 | | | P0DP23 | | | CALM1 | | | P30085 | | | | | KCY | | P63241 | | IF5A1 |
| P0DP25 | | | CALM3 | | | P0DP25 | | | CALM3 | | | P61769 | | | | | B2MG | | P18669 | | PGAM1 |
| P30085 | | | KCY | | | P30085 | | | KCY | | | P60660 | | | | | MYL6 | | Q9BRK5 | | CAB45 |
| P61769 | | | B2MG | | | P61769 | | | B2MG | | | Q99880 | | | | | H2B1L | | P31949 | | S10AB |
| P60660 | | | MYL6 | | | P60660 | | | MYL6 | | | Q99879 | | | | | H2B1M | | O75874 | | IDHC |
| Q99880 | | | H2B1L | | | Q99880 | | | H2B1L | | | Q93079 | | | | | H2B1H | | P68871 | | HBB |
| Q99879 | | | H2B1M | | | Q99879 | | | H2B1M | | | Q5QNW6 | | | | | H2B2F | | P33778 | | H2B1B |
| Q93079 | | | H2B1H | | | Q93079 | | | H2B1H | | | Q99877 | | | | | H2B1N | | P06899 | | H2B1J |
| Q5QNW6 | | | H2B2F | | | Q5QNW6 | | | H2B2F | | | O60814 | | | | | H2B1K | | P23527 | | H2B1O |
| Q99877 | | | H2B1N | | | Q99877 | | | H2B1N | | | P62807 | | | | | H2B1C | | Q16778 | | H2B2E |
| O60814 | | | H2B1K | | | O60814 | | | H2B1K | | | P58876 | | | | | H2B1D | | Q8N257 | | H2B3B |
| P62807 | | | H2B1C | | | P62807 | | | H2B1C | | | P57053 | | | | | H2BFS | | P07384 | | CAN1 |
| P58876 | | | H2B1D | | | P58876 | | | H2B1D | | | P63241 | | | | | IF5A1 | | Q02818 | | NUCB1 |
| P57053 | | | H2BFS | | | P57053 | | | H2BFS | | | P18669 | | | | | PGAM1 | | Q99497 | | PARK7 |
| P63241 | | | IF5A1 | | | P63241 | | | IF5A1 | | | Q9BRK5 | | | | | CAB45 | | Q8NBJ4 | | GOLM1 |
| P18669 | | | PGAM1 | | | P18669 | | | PGAM1 | | | P31949 | | | | | S10AB | | Q13217 | | DNJC3 |
| Q9BRK5 | | | CAB45 | | | Q9BRK5 | | | CAB45 | | | O75874 | | | | | IDHC | | P04080 | | CYTB |
| P31949 | | | S10AB | | | P31949 | | | S10AB | | | P68871 | | | | | HBB | | P07237 | | PDIA1 |
| O75874 | | | IDHC | | | O75874 | | | IDHC | | | P33778 | | | | | H2B1B | | P28799 | | GRN |
| P68871 | | | HBB | | | P68871 | | | HBB | | | P06899 | | | | | H2B1J | | P17931 | | LEG3 |
| P33778 | | | H2B1B | | | P33778 | | | H2B1B | | | P23527 | | | | | H2B1O | | P62805 | | H4 |
| P06899 | | | H2B1J | | | P06899 | | | H2B1J | | | Q16778 | | | | | H2B2E | | P36952 | | SPB5 |
| P23527 | | | H2B1O | | | P23527 | | | H2B1O | | | Q8N257 | | | | | H2B3B | | P05090 | | APOD |
| Q16778 | | | H2B2E | | | Q16778 | | | H2B2E | | | P07384 | | | | | CAN1 | | P04004 | | VTNC |
| Q8N257 | | | H2B3B | | | Q8N257 | | | H2B3B | | | Q02818 | | | | | NUCB1 | | P02766 | | TTHY |
| P07384 | | | CAN1 | | | P07384 | | | CAN1 | | | Q99497 | | | | | PARK7 | | Q14764 | | MVP |
| Q02818 | | | NUCB1 | | | Q02818 | | | NUCB1 | | | Q8NBJ4 | | | | | GOLM1 | | P01782 | | HV309 |
| Q99497 | | | PARK7 | | | Q99497 | | | PARK7 | | | Q13217 | | | | | DNJC3 | | P08571 | | CD14 |
| Q8NBJ4 | | | GOLM1 | | | Q8NBJ4 | | | GOLM1 | | | P04080 | | | | | CYTB | | P04217 | | A1BG |
| P52209 | | | 6PGD | | | P52209 | | | 6PGD | | | P07237 | | | | | PDIA1 | | P00338 | | LDHA |
| Q13217 | | | DNJC3 | | | Q13217 | | | DNJC3 | | | P28799 | | | | | GRN | | P12830 | | CADH1 |
| P04080 | | | CYTB | | | P04080 | | | CYTB | | | P17931 | | | | | LEG3 | | P50995 | | ANX11 |
| P07237 | | | PDIA1 | | | P07237 | | | PDIA1 | | | P62805 | | | | | H4 | | Q04828 | | AK1C1 |
| P28799 | | | GRN | | | P28799 | | | GRN | | | P36952 | | | | | SPB5 | | P29401 | | TKT |
| P17931 | | | LEG3 | | | P17931 | | | LEG3 | | | P62258 | | | | | 1433E | | O00299 | | CLIC1 |
| P62805 | | | H4 | | | P62805 | | | H4 | | | P05090 | | | | | APOD | | A0A0C4DH38 | | HV551 |
| P36952 | | | SPB5 | | | P36952 | | | SPB5 | | | P01008 | | | | | ANT3 | | P0CG47 | | UBB |
| P62258 | | | 1433E | | | P62258 | | | 1433E | | | P04004 | | | | | VTNC | | P0CG48 | | UBC |
| P05090 | | | APOD | | | P05090 | | | APOD | | | P02766 | | | | | TTHY | | P62987 | | RL40 |
| P01008 | | | ANT3 | | | P01008 | | | ANT3 | | | Q14764 | | | | | MVP | | P62979 | | RS27A |
| P04004 | | | VTNC | | | P04004 | | | VTNC | | | P80748 | | | | | LV321 | | P02750 | | A2GL |
| P50395 | | | GDIB | | | P50395 | | | GDIB | | | P01782 | | | | | HV309 | | P03950 | | ANGI |
| P02766 | | | TTHY | | | P02766 | | | TTHY | | | P08571 | | | | | CD14 | | P47895 | | AL1A3 |
| Q14764 | | | MVP | | | Q14764 | | | MVP | | | P04217 | | | | | A1BG | | A0A075B6K4 | | LV310 |
| P01782 | | | HV309 | | | P01782 | | | HV309 | | | P00338 | | | | | LDHA | | P01717 | | LV325 |
| P08571 | | | CD14 | | | P08571 | | | CD14 | | | P02765 | | | | | FETUA | | P16401 | | H15 |
| P04217 | | | A1BG | | | P04217 | | | A1BG | | | P12830 | | | | | CADH1 | | P01700 | | LV147 |
| P00338 | | | LDHA | | | P00338 | | | LDHA | | | P50995 | | | | | ANX11 | | P00390 | | GSHR |
| P02765 | | | FETUA | | | P02765 | | | FETUA | | | Q04828 | | | | | AK1C1 | | P55072 | | TERA |
| P12830 | | | CADH1 | | | P12830 | | | CADH1 | | | P29401 | | | | | TKT | | P05155 | | IC1 |
| P50995 | | | ANX11 | | | P50995 | | | ANX11 | | | O00299 | | | | | CLIC1 | | P01825 | | HV459 |
| P22392 | | | NDKB | | | P22392 | | | NDKB | | | P08603 | | | | | CFAH | | P0DP08 | | HVD82 |
| Q04828 | | | AK1C1 | | | Q04828 | | | AK1C1 | | | A0A0C4DH38 | | | | | HV551 | | P0DP07 | | HV431 |
| P18510 | | | IL1RA | | | P18510 | | | IL1RA | | | P0CG47 | | | | | UBB | | A0A0C4DH41 | | HV461 |
| P29401 | | | TKT | | | P29401 | | | TKT | | | P0CG48 | | | | | UBC | | P0DP06 | | HVD34 |
| O00299 | | | CLIC1 | | | O00299 | | | CLIC1 | | | P62987 | | | | | RL40 | | P01824 | | HV439 |
| P30101 | | | PDIA3 | | | P30101 | | | PDIA3 | | | P62979 | | | | | RS27A | | A0A0A0MS15 | | HV349 |
| P08603 | | | CFAH | | | P08603 | | | CFAH | | | P07108 | | | | | ACBP | | Q9HC38 | | GLOD4 |
| A0A0C4DH38 | | | HV551 | | | A0A0C4DH38 | | | HV551 | | | P02750 | | | | | A2GL | | A0A0B4J2D9 | | KVD13 |
| P0CG47 | | | UBB | | | P0CG47 | | | UBB | | | P01615 | | | | | KVD28 | | P0DP09 | | KV113 |
| P0CG48 | | | UBC | | | P0CG48 | | | UBC | | | A0A075B6P5 | | | | | KV228 | | P16152 | | CBR1 |
| P62987 | | | RL40 | | | P62987 | | | RL40 | | | P03950 | | | | | ANGI | | P26447 | | S10A4 |
| P62979 | | | RS27A | | | P62979 | | | RS27A | | | Q96S96 | | | | | PEBP4 | | P06753 | | TPM3 |
| P07108 | | | ACBP | | | P07108 | | | ACBP | | | P47895 | | | | | AL1A3 | | P01033 | | TIMP1 |
| P02750 | | | A2GL | | | P02750 | | | A2GL | | | A0A075B6K4 | | | | | LV310 | | P22626 | | ROA2 |
| P01615 | | | KVD28 | | | P01615 | | | KVD28 | | | P01717 | | | | | LV325 | | P02545 | | LMNA |
| A0A075B6P5 | | | KV228 | | | A0A075B6P5 | | | KV228 | | | P09467 | | | | | F16P1 | | P81605 | | DCD |
| Q9Y490 | | | TLN1 | | | Q9Y490 | | | TLN1 | | | P16401 | | | | | H15 | | P01624 | | KV315 |
| P03950 | | | ANGI | | | P03950 | | | ANGI | | | P00751 | | | | | CFAB | | P34096 | | RNAS4 |
| Q96S96 | | | PEBP4 | | | Q96S96 | | | PEBP4 | | | P01700 | | | | | LV147 | | P06312 | | KV401 |
| P47895 | | | AL1A3 | | | P47895 | | | AL1A3 | | | P00390 | | | | | GSHR | | P09651 | | ROA1 |
| A0A075B6K4 | | | LV310 | | | A0A075B6K4 | | | LV310 | | | P14625 | | | | | ENPL | | Q32P51 | | RA1L2 |
| P01717 | | | LV325 | | | P01717 | | | LV325 | | | P55072 | | | | | TERA | | Q92743 | | HTRA1 |
| P09467 | | | F16P1 | | | P09467 | | | F16P1 | | | Q13162 | | | | | PRDX4 | | P52565 | | GDIR1 |
| P16401 | | | H15 | | | P16401 | | | H15 | | | P55058 | | | | | PLTP | | P07339 | | CATD |
| P00751 | | | CFAB | | | P00751 | | | CFAB | | | P05155 | | | | | IC1 | | O75083 | | WDR1 |
| P01700 | | | LV147 | | | P01700 | | | LV147 | | | P01825 | | | | | HV459 | | P14550 | | AK1A1 |
| P00390 | | | GSHR | | | P00390 | | | GSHR | | | P0DP08 | | | | | HVD82 | | P09429 | | HMGB1 |
| P14625 | | | ENPL | | | P14625 | | | ENPL | | | P0DP07 | | | | | HV431 | | Q99954 | | SMR3A |
| P55072 | | | TERA | | | P55072 | | | TERA | | | A0A0C4DH41 | | | | | HV461 | | P01611 | | KVD12 |
| Q13162 | | | PRDX4 | | | Q13162 | | | PRDX4 | | | P0DP06 | | | | | HVD34 | | A0A0C4DH73 | | KV112 |
| P55058 | | | PLTP | | | P55058 | | | PLTP | | | P01824 | | | | | HV439 | | P08582 | | TRFM |
| P05155 | | | IC1 | | | P05155 | | | IC1 | | | A0A0A0MS15 | | | | | HV349 | | Q7Z5P9 | | MUC19 |
| P46940 | | | IQGA1 | | | P46940 | | | IQGA1 | | | Q9HC38 | | | | | GLOD4 | | P02511 | | CRYAB |
| P01825 | | | HV459 | | | P01825 | | | HV459 | | | A0A0B4J2D9 | | | | | KVD13 | | P21964 | | COMT |
| P0DP08 | | | HVD82 | | | P0DP08 | | | HVD82 | | | P0DP09 | | | | | KV113 | | P02814 | | SMR3B |
| P0DP07 | | | HV431 | | | P0DP07 | | | HV431 | | | O43707 | | | | | ACTN4 | | O14950 | | ML12B |
| A0A0C4DH41 | | | HV461 | | | A0A0C4DH41 | | | HV461 | | | P16152 | | | | | CBR1 | | P24844 | | MYL9 |
| P0DP06 | | | HVD34 | | | P0DP06 | | | HVD34 | | | P26447 | | | | | S10A4 | | P19105 | | ML12A |
| P01824 | | | HV439 | | | P01824 | | | HV439 | | | P06753 | | | | | TPM3 | | Q09666 | | AHNK |
| P52566 | | | GDIR2 | | | P52566 | | | GDIR2 | | | P01033 | | | | | TIMP1 | | P21926 | | CD9 |
| A0A0A0MS15 | | | HV349 | | | A0A0A0MS15 | | | HV349 | | | P40925 | | | | | MDHC | | P01859 | | *IGHG2 |
| Q9HC38 | | | GLOD4 | | | Q9HC38 | | | GLOD4 | | | P22626 | | | | | ROA2 | | P68032 | | ACTC |
| A0A0B4J2D9 | | | KVD13 | | | A0A0B4J2D9 | | | KVD13 | | | P19021 | | | | | AMD | | P62736 | | ACTA |
| P0DP09 | | | KV113 | | | P0DP09 | | | KV113 | | | P02545 | | | | | LMNA | | P63267 | | ACTH |
| O43707 | | | ACTN4 | | | O43707 | | | ACTN4 | | | P81605 | | | | | DCD | | P68133 | | ACTS |
| Q99538 | | | LGMN | | | Q99538 | | | LGMN | | | P01624 | | | | | KV315 | | P02765 | | FETUA |
| P16152 | | | CBR1 | | | P16152 | | | CBR1 | | | P34096 | | | | | RNAS4 | | P08603 | | CFAH |
| P26447 | | | S10A4 | | | P26447 | | | S10A4 | | | P06312 | | | | | KV401 | | P01593 | | KVD33 |
| P06753 | | | TPM3 | | | P06753 | | | TPM3 | | | A0A0C4DH69 | | | | | KV109 | | P01594 | | KV133 |
| P01033 | | | TIMP1 | | | P01033 | | | TIMP1 | | | P09651 | | | | | ROA1 | | P00751 | | CFAB |
| P40925 | | | MDHC | | | P40925 | | | MDHC | | | Q32P51 | | | | | RA1L2 | | P22314 | | UBA1 |
| P22626 | | | ROA2 | | | P22626 | | | ROA2 | | | Q92743 | | | | | HTRA1 | | P01714 | | LV319 |
| P19021 | | | AMD | | | P19021 | | | AMD | | | A0A075B6R9 | | | | | KVD24 | | P62277 | | RS13 |
| P02545 | | | LMNA | | | P02545 | | | LMNA | | | A0A0C4DH68 | | | | | KV224 | | P13693 | | TCTP |
| P81605 | | | DCD | | | P81605 | | | DCD | | | P52565 | | | | | GDIR1 | | P09228 | | CYTT |
| P22314 | | | UBA1 | | | P22314 | | | UBA1 | | | P01714 | | | | | LV319 | | P40121 | | CAPG |
| P01624 | | | KV315 | | | P01624 | | | KV315 | | | P07339 | | | | | CATD | | P01008 | | ANT3 |
| P34096 | | | RNAS4 | | | P34096 | | | RNAS4 | | | O75083 | | | | | WDR1 | | P22392 | | NDKB |
| P06312 | | | KV401 | | | P06312 | | | KV401 | | | P14550 | | | | | AK1A1 | | P18510 | | IL1RA |
| O00151 | | | PDLI1 | | | O00151 | | | PDLI1 | | | P17858 | | | | | PFKAL | | P30101 | | PDIA3 |
| A0A0C4DH69 | | | KV109 | | | A0A0C4DH69 | | | KV109 | | | P09429 | | | | | HMGB1 | | P07108 | | ACBP |
| P09651 | | | ROA1 | | | P09651 | | | ROA1 | | | P25815 | | | | | S100P | | P09467 | | F16P1 |
| Q32P51 | | | RA1L2 | | | Q32P51 | | | RA1L2 | | | Q99954 | | | | | SMR3A | | Q13162 | | PRDX4 |
| Q99878 | | | H2A1J | | | Q99878 | | | H2A1J | | | P40926 | | | | | MDHM | | P46940 | | IQGA1 |
| Q96KK5 | | | H2A1H | | | Q96KK5 | | | H2A1H | | | P01611 | | | | | KVD12 | | O43707 | | ACTN4 |
| Q16777 | | | H2A2C | | | Q16777 | | | H2A2C | | | A0A0C4DH73 | | | | | KV112 | | P40925 | | MDHC |
| Q9BTM1 | | | H2AJ | | | Q9BTM1 | | | H2AJ | | | P62277 | | | | | RS13 | | P19021 | | AMD |
| P04908 | | | H2A1B | | | P04908 | | | H2A1B | | | P08582 | | | | | TRFM | | Q99878 | | H2A1J |
| Q6FI13 | | | H2A2A | | | Q6FI13 | | | H2A2A | | | Q7Z5P9 | | | | | MUC19 | | Q96KK5 | | H2A1H |
| Q7L7L0 | | | H2A3 | | | Q7L7L0 | | | H2A3 | | | P02511 | | | | | CRYAB | | Q16777 | | H2A2C |
| Q93077 | | | H2A1C | | | Q93077 | | | H2A1C | | | P49788 | | | | | TIG1 | | Q9BTM1 | | H2AJ |
| P0C0S8 | | | H2A1 | | | P0C0S8 | | | H2A1 | | | A0A087WSZ0 | | | | | KVD08 | | P04908 | | H2A1B |
| P20671 | | | H2A1D | | | P20671 | | | H2A1D | | | P21964 | | | | | COMT | | Q6FI13 | | H2A2A |
| Q8IUE6 | | | H2A2B | | | Q8IUE6 | | | H2A2B | | | P25789 | | | | | PSA4 | | Q7L7L0 | | H2A3 |
| P16104 | | | H2AX | | | P16104 | | | H2AX | | | P02814 | | | | | SMR3B | | Q93077 | | H2A1C |
| Q96QV6 | | | H2A1A | | | Q96QV6 | | | H2A1A | | | P19823 | | | | | ITIH2 | | P0C0S8 | | H2A1 |
| Q92743 | | | HTRA1 | | | Q92743 | | | HTRA1 | | | O14950 | | | | | ML12B | | P20671 | | H2A1D |
| A0A075B6R9 | | | KVD24 | | | A0A075B6R9 | | | KVD24 | | | P24844 | | | | | MYL9 | | Q8IUE6 | | H2A2B |
| A0A0C4DH68 | | | KV224 | | | A0A0C4DH68 | | | KV224 | | | P19105 | | | | | ML12A | | P16104 | | H2AX |
| P52565 | | | GDIR1 | | | P52565 | | | GDIR1 | | | Q09666 | | | | | AHNK | | Q96QV6 | | H2A1A |
| P07339 | | | CATD | | | P07339 | | | CATD | | | P21926 | | | | | CD9 | | P25815 | | S100P |
| Q15181 | | | IPYR | | | Q15181 | | | IPYR | | | P07195 | | | | | LDHB | | P40926 | | MDHM |
| P01042 | | | KNG1 | | | P01042 | | | KNG1 | | | Q8TD06 | | | | | AGR3 | | P49788 | | TIG1 |
| O75083 | | | WDR1 | | | O75083 | | | WDR1 | | | P52209 | | | | | 6PGD | | P19823 | | ITIH2 |
| P14550 | | | AK1A1 | | | P14550 | | | AK1A1 | | | P22392 | | | | | NDKB | | Q5VSP4 | | LC1L1 |
| P17987 | | | TCPA | | | P17987 | | | TCPA | | | P18510 | | | | | IL1RA | | P0DOX2 | | *IGA2 |
| P17858 | | | PFKAL | | | P17858 | | | PFKAL | | | P06310 | | | | | KV230 | | P0DP24 | | CALM2 |
| P09429 | | | HMGB1 | | | P09429 | | | HMGB1 | | | A0A075B6S6 | | | | | KVD30 | | P0DP23 | | CALM1 |
| P40939 | | | ECHA | | | P40939 | | | ECHA | | | P30101 | | | | | PDIA3 | | P0DP25 | | CALM3 |
| P25815 | | | S100P | | | P25815 | | | S100P | | | Q99538 | | | | | LGMN | | P52209 | | 6PGD |
| Q99954 | | | SMR3A | | | Q99954 | | | SMR3A | | | P22314 | | | | | UBA1 | | P13489 | | RINI |
| P13797 | | | PLST | | | P13797 | | | PLST | | | O00151 | | | | | PDLI1 | | P62258 | | 1433E |
| P40926 | | | MDHM | | | P40926 | | | MDHM | | | Q15181 | | | | | IPYR | | P80748 | | LV321 |
| P01611 | | | KVD12 | | | P01611 | | | KVD12 | | | P01042 | | | | | KNG1 | | P06310 | | KV230 |
| A0A0C4DH73 | | | KV112 | | | A0A0C4DH73 | | | KV112 | | | P13693 | | | | | TCTP | | A0A075B6S6 | | KVD30 |
| P62277 | | | RS13 | | | P62277 | | | RS13 | | | P27797 | | | | | CALR | | P19971 | | TYPH |
| P13693 | | | TCTP | | | P13693 | | | TCTP | | | P35579 | | | | | MYH9 | | Q96S96 | | PEBP4 |
| P27797 | | | CALR | | | P27797 | | | CALR | | | P50395 | | | | | GDIB | | P55058 | | PLTP |
| P08582 | | | TRFM | | | P08582 | | | TRFM | | | P52566 | | | | | GDIR2 | | P31151 | | S10A7 |
| Q7Z5P9 | | | MUC19 | | | Q7Z5P9 | | | MUC19 | | | P40939 | | | | | ECHA | | A0A0C4DH32 | | HV320 |
| P02511 | | | CRYAB | | | P02511 | | | CRYAB | | | Q99933 | | | | | BAG1 | | P25705 | | ATPA |
| P49788 | | | TIG1 | | | P49788 | | | TIG1 | | | P07437 | | | | | TBB5 | | P52907 | | CAZA1 |
| A0A087WSZ0 | | | KVD08 | | | A0A087WSZ0 | | | KVD08 | | | Q04695 | | | | | K1C17 | | A0A0C4DH69 | | KV109 |
| P21964 | | | COMT | | | P21964 | | | COMT | | | P40121 | | | | | CAPG | | A0A075B6R9 | | KVD24 |
| P25789 | | | PSA4 | | | P25789 | | | PSA4 | | | P13489 | | | | | RINI | | A0A0C4DH68 | | KV224 |
| P02814 | | | SMR3B | | | P02814 | | | SMR3B | | | P27348 | | | | | 1433T | | P49189 | | AL9A1 |
| P19823 | | | ITIH2 | | | P19823 | | | ITIH2 | | | P19971 | | | | | TYPH | | Q15181 | | IPYR |
| O14950 | | | ML12B | | | O14950 | | | ML12B | | | P01593 | | | | | KVD33 | | Q8WVQ1 | | CANT1 |
| P24844 | | | MYL9 | | | P24844 | | | MYL9 | | | P01594 | | | | | KV133 | | P17858 | | PFKAL |
| P19105 | | | ML12A | | | P19105 | | | ML12A | | | Q9Y490 | | | | | TLN1 | | P08670 | | VIME |
| Q09666 | | | AHNK | | | Q09666 | | | AHNK | | | P46940 | | | | | IQGA1 | | P16070 | | CD44 |
| Q99933 | | | BAG1 | | | Q99933 | | | BAG1 | | | P31151 | | | | | S10A7 | | P0C0L4 | | CO4A |
| P21926 | | | CD9 | | | P21926 | | | CD9 | | | A0A0C4DH32 | | | | | HV320 | | P0C0L5 | | CO4B |
| P07195 | | | LDHB | | | P07195 | | | LDHB | | | P25705 | | | | | ATPA | | P13797 | | PLST |
| Q8TD06 | | | AGR3 | | | Q8TD06 | | | AGR3 | | | Q14118 | | | | | DAG1 | | Q14697 | | GANAB |
| P40121 | | | CAPG | | | P40121 | | | CAPG | | | P52907 | | | | | CAZA1 | | O00462 | | MANBA |
| P13489 | | | RINI | | | P13489 | | | RINI | | | Q99878 | | | | | H2A1J | | P08758 | | ANXA5 |
| P80748 | | | LV321 | | | P80748 | | | LV321 | | | Q96KK5 | | | | | H2A1H | | P25789 | | PSA4 |
| P06310 | | | KV230 | | | P06310 | | | KV230 | | | Q16777 | | | | | H2A2C | | P43490 | | NAMPT |
| A0A075B6S6 | | | KVD30 | | | A0A075B6S6 | | | KVD30 | | | Q9BTM1 | | | | | H2AJ | | Q8TD19 | | NEK9 |
| P01593 | | | KVD33 | | | P01593 | | | KVD33 | | | P04908 | | | | | H2A1B | | P07195 | | LDHB |
| P01594 | | | KV133 | | | P01594 | | | KV133 | | | Q6FI13 | | | | | H2A2A | | Q9Y5Z4 | | HEBP2 |
| P48643 | | | TCPE | | | P48643 | | | TCPE | | | Q7L7L0 | | | | | H2A3 | | Q9NSB4 | | KRT82 |
| Q8TCD5 | | | NT5C | | | Q8TCD5 | | | NT5C | | | Q93077 | | | | | H2A1C | | Q6NT89 | | TRNP1 |
| P01714 | | | LV319 | | | P01714 | | | LV319 | | | P0C0S8 | | | | | H2A1 | | P48634 | | PRC2A |
| P06576 | | | ATPB | | | P06576 | | | ATPB | | | P20671 | | | | | H2A1D | | Q9UL51 | | HCN2 |
| P0C0L4 | | | CO4A | | | P0C0L4 | | | CO4A | | | Q8IUE6 | | | | | H2A2B | | Q86UT5 | | NHRF4 |
| P0C0L5 | | | CO4B | | | P0C0L5 | | | CO4B | | | P16104 | | | | | H2AX | | Q8NCB2 | | CAMKV |
| Q9UQ80 | | | PA2G4 | | | Q9UQ80 | | | PA2G4 | | | Q96QV6 | | | | | H2A1A | | Q6SPF0 | | SAMD1 |
| Q15056 | | | IF4H | | | Q15056 | | | IF4H | | | P49189 | | | | | AL9A1 | | Q9NRI5 | | DISC1 |
| Q06210 | | | GFPT1 | | | Q06210 | | | GFPT1 | | | Q8WVQ1 | | | | | CANT1 | | P25774 | | CATS |
| O94808 | | | GFPT2 | | | O94808 | | | GFPT2 | | | P17987 | | | | | TCPA | | A8MYP8 | | ODF3B |
| P01860 | | | *IGHG3 | | | P01860 | | | *IGHG3 | | | P08670 | | | | | VIME | | O00716 | | E2F3 |
| P07437 | | | TBB5 | | | P07437 | | | TBB5 | | | P16070 | | | | | CD44 | | Q6ZMY3 | | SPOC1 |
| Q04695 | | | K1C17 | | | Q04695 | | | K1C17 | | | P0C0L4 | | | | | CO4A | | P0DOY3 | | *IGLC3 |
| P27348 | | | 1433T | | | P27348 | | | 1433T | | | P0C0L5 | | | | | CO4B | | P0DOY2 | | *IGLC2 |
| P19971 | | | TYPH | | | P19971 | | | TYPH | | | P13797 | | | | | PLST | | P68371 | | TBB4B |
| P31151 | | | S10A7 | | | P31151 | | | S10A7 | | | P19652 | | | | | A1AG2 | | P08779 | | K1C16 |
| A0A0C4DH32 | | | HV320 | | | A0A0C4DH32 | | | HV320 | | | Q14697 | | | | | GANAB | | A0A0B4J1V0 | | HV315 |
| P25705 | | | ATPA | | | P25705 | | | ATPA | | | O00462 | | | | | MANBA | | Q01469 | | FABP5 |
| Q14118 | | | DAG1 | | | Q14118 | | | DAG1 | | | P19827 | | | | | ITIH1 | | A0A0C4DH25 | | KVD20 |
| P52907 | | | CAZA1 | | | P52907 | | | CAZA1 | | | P08758 | | | | | ANXA5 | | Q6S8J3 | | POTEE |
| P49189 | | | AL9A1 | | | P49189 | | | AL9A1 | | | Q9BS40 | | | | | LXN | | P31947 | | 1433S |
| Q8WVQ1 | | | CANT1 | | | Q8WVQ1 | | | CANT1 | | | P09525 | | | | | ANXA4 | | Q9UBG3 | | CRNN |
| P08670 | | | VIME | | | P08670 | | | VIME | | | P40306 | | | | | PSB10 | | A0A0B4J1Y9 | | HV372 |
| P16070 | | | CD44 | | | P16070 | | | CD44 | | | P17812 | | | | | PYRG1 | | P28325 | | CYTD |
| P19652 | | | A1AG2 | | | P19652 | | | A1AG2 | | | P43490 | | | | | NAMPT | | Q9UBC9 | | SPRR3 |
| Q14697 | | | GANAB | | | Q14697 | | | GANAB | | | Q00610 | | | | | CLH1 | | A0A0A0MRZ8 | | KVD11 |
| O00462 | | | MANBA | | | O00462 | | | MANBA | | | P01718 | | | | | LV327 | | P04433 | | KV311 |
| P19827 | | | ITIH1 | | | P19827 | | | ITIH1 | | | Q8TD19 | | | | | NEK9 | | P01743 | | HV146 |
| P08758 | | | ANXA5 | | | P08758 | | | ANXA5 | | | P55795 | | | | | HNRH2 | | A0A0C4DH72 | | KV106 |
| Q9BS40 | | | LXN | | | Q9BS40 | | | LXN | | | P31943 | | | | | HNRH1 | | Q06323 | | PSME1 |
| Q9H008 | | | LHPP | | | Q9H008 | | | LHPP | | | Q9Y5Z4 | | | | | HEBP2 | | A0A075B6S9 | | KV137 |
| O95834 | | | EMAL2 | | | O95834 | | | EMAL2 | | | Q96FW1 | | | | | OTUB1 | | P0DSN7 | | KVD37 |
| P09525 | | | ANXA4 | | | P09525 | | | ANXA4 | | | O75882 | | | | | ATRN | | A0A0C4DH42 | | HV366 |
| P40306 | | | PSB10 | | | P40306 | | | PSB10 | | | P00326 | | | | | ADH1G | | Q08431 | | MFGM |
| Q01082 | | | SPTB2 | | | Q01082 | | | SPTB2 | | | P07327 | | | | | ADH1A | | P00441 | | SODC |
| P17812 | | | PYRG1 | | | P17812 | | | PYRG1 | | | Q9NSB4 | | | | | KRT82 | | Q15293 | | RCN1 |
| P43490 | | | NAMPT | | | P43490 | | | NAMPT | | | P06744 | | | | | G6PI | | A0A075B6I0 | | LV861 |
| P17655 | | | CAN2 | | | P17655 | | | CAN2 | | | Q6NT89 | | | | | TRNP1 | | P04179 | | SODM |
| Q00610 | | | CLH1 | | | Q00610 | | | CLH1 | | | P48634 | | | | | PRC2A | | P15814 | | *IGLL1 |
| P01718 | | | LV327 | | | P01718 | | | LV327 | | | Q9UL51 | | | | | HCN2 | | P19961 | | AMY2B |
| Q07020 | | | RL18 | | | Q07020 | | | RL18 | | | Q9HAP2 | | | | | MLXIP | | P0DTE7 | | AMY1B |
| Q7Z4S6 | | | KI21A | | | Q7Z4S6 | | | KI21A | | | Q96JH8 | | | | | RADIL | | P04746 | | AMYP |
| O00534 | | | VMA5A | | | O00534 | | | VMA5A | | | Q86UT5 | | | | | NHRF4 | | P04745 | | AMY1A |
| Q8TD19 | | | NEK9 | | | Q8TD19 | | | NEK9 | | | Q8NCB2 | | | | | CAMKV | | P0DTE8 | | AMY1C |
| O15144 | | | ARPC2 | | | O15144 | | | ARPC2 | | | Q6SPF0 | | | | | SAMD1 | | Q00796 | | DHSO |
| Q8IZP2 | | | ST134 | | | Q8IZP2 | | | ST134 | | | Q9NRI5 | | | | | DISC1 | | O60664 | | PLIN3 |
| P50502 | | | F10A1 | | | P50502 | | | F10A1 | | | P25774 | | | | | CATS | | P0DOX3 | | *IGD |
| Q8NFI4 | | | F10A5 | | | Q8NFI4 | | | F10A5 | | | O60437 | | | | | PEPL | | A0A075B6I9 | | LV746 |
| P55795 | | | HNRH2 | | | P55795 | | | HNRH2 | | | A8MYP8 | | | | | ODF3B | | P04211 | | LV743 |
| P31943 | | | HNRH1 | | | P31943 | | | HNRH1 | | | Q9BXJ3 | | | | | C1QT4 | | A0A075B6H8 | | KVD42 |
| P35606 | | | COPB2 | | | P35606 | | | COPB2 | | | O00716 | | | | | E2F3 | | A0A075B6J9 | | LV218 |
| Q99832 | | | TCPH | | | Q99832 | | | TCPH | | | Q6ZMY3 | | | | | SPOC1 | | A0A0B4J1U7 | | HV601 |
| P28066 | | | PSA5 | | | P28066 | | | PSA5 | | | P0DOY3 | | | | | *IGLC3 | | P37837 | | TALDO |
| P11216 | | | PYGB | | | P11216 | | | PYGB | | | P0DOY2 | | | | | *IGLC2 | | A0A0A0MT36 | | KVD21 |
| Q9Y5Z4 | | | HEBP2 | | | Q9Y5Z4 | | | HEBP2 | | | P68371 | | | | | TBB4B | | A0A0C4DH24 | | KV621 |
| Q9H6S3 | | | ES8L2 | | | Q9H6S3 | | | ES8L2 | | | A0M8Q6 | | | | | *IGLC7 | | P01701 | | LV151 |
| P52788 | | | SPSY | | | P52788 | | | SPSY | | | P08779 | | | | | K1C16 | | A0A075B6I4 | | LVX54 |
| Q96FW1 | | | OTUB1 | | | Q96FW1 | | | OTUB1 | | | A0A0B4J1V0 | | | | | HV315 | | P01721 | | LV657 |
| O75882 | | | ATRN | | | O75882 | | | ATRN | | | Q01469 | | | | | FABP5 | | Q9BUP0 | | EFHD1 |
| P00326 | | | ADH1G | | | P00326 | | | ADH1G | | | A0A0C4DH25 | | | | | KVD20 | | Q96C19 | | EFHD2 |
| P07327 | | | ADH1A | | | P07327 | | | ADH1A | | | Q6S8J3 | | | | | POTEE | | A0A075B6Q5 | | HV364 |
| Q9NSB4 | | | KRT82 | | | Q9NSB4 | | | KRT82 | | | P00747 | | | | | PLMN | | Q99574 | | NEUS |
| P06744 | | | G6PI | | | P06744 | | | G6PI | | | P31947 | | | | | 1433S | | O00391 | | QSOX1 |
| Q6NT89 | | | TRNP1 | | | Q6NT89 | | | TRNP1 | | | Q9UBG3 | | | | | CRNN | | P01703 | | LV140 |
| P25398 | | | RS12 | | | P25398 | | | RS12 | | | A0A0B4J1Y9 | | | | | HV372 | | P23526 | | SAHH |
| P48634 | | | PRC2A | | | P48634 | | | PRC2A | | | P28325 | | | | | CYTD | | O43548 | | TGM5 |
| Q9UL51 | | | HCN2 | | | Q9UL51 | | | HCN2 | | | Q9UBC9 | | | | | SPRR3 | | Q9UBZ9 | | REV1 |
| Q9HAP2 | | | MLXIP | | | Q9HAP2 | | | MLXIP | | | A0A0A0MRZ8 | | | | | KVD11 | | Q15782 | | CH3L2 |
| Q9NY33 | | | DPP3 | | | Q9NY33 | | | DPP3 | | | P04433 | | | | | KV311 | | P23284 | | PPIB |
| Q96JH8 | | | RADIL | | | Q96JH8 | | | RADIL | | | P01743 | | | | | HV146 | | Q15365 | | PCBP1 |
| P36955 | | | PEDF | | | P36955 | | | PEDF | | | A0A0C4DH72 | | | | | KV106 | | Q15366 | | PCBP2 |
| Q86UT5 | | | NHRF4 | | | Q86UT5 | | | NHRF4 | | | Q06323 | | | | | PSME1 | | P57721 | | PCBP3 |
| Q8NCB2 | | | CAMKV | | | Q8NCB2 | | | CAMKV | | | O15143 | | | | | ARC1B | | P57723 | | PCBP4 |
| Q6SPF0 | | | SAMD1 | | | Q6SPF0 | | | SAMD1 | | | A0A075B6S9 | | | | | KV137 | | Q7Z614 | | SNX20 |
| Q86YR7 | | | MF2L2 | | | Q86YR7 | | | MF2L2 | | | P0DSN7 | | | | | KVD37 | | A6NKD9 | | CC85C |
| Q9NRI5 | | | DISC1 | | | Q9NRI5 | | | DISC1 | | | A0A0C4DH42 | | | | | HV366 | | Q13315 | | ATM |
| P25774 | | | CATS | | | P25774 | | | CATS | | | A0A0C4DH34 | | | | | HV428 | | P46459 | | NSF |
| O60437 | | | PEPL | | | O60437 | | | PEPL | | | Q08431 | | | | | MFGM | | Q9UBQ7 | | GRHPR |
| A8MYP8 | | | ODF3B | | | A8MYP8 | | | ODF3B | | | P00441 | | | | | SODC | | Q9NZJ7 | | MTCH1 |
| A0A286YF58 | | | TM271 | | | A0A286YF58 | | | TM271 | | | A0A0C4DH29 | | | | | HV103 | | Q9Y6X0 | | SETBP |
| Q9BXJ3 | | | C1QT4 | | | Q9BXJ3 | | | C1QT4 | | | Q15293 | | | | | RCN1 | | P60900 | | PSA6 |
| O00716 | | | E2F3 | | | O00716 | | | E2F3 | | | P55064 | | | | | AQP5 | | Q6ZS81 | | WDFY4 |
| Q6ZMY3 | | | SPOC1 | | | Q6ZMY3 | | | SPOC1 | | | A0A075B6I0 | | | | | LV861 | | Q13608 | | PEX6 |
| P0DOY3 | | | *IGLC3 | | | P0DOY3 | | | *IGLC3 | | | P04179 | | | | | SODM | | Q9HBH5 | | RDH14 |
| P0DOY2 | | | *IGLC2 | | | P0DOY2 | | | *IGLC2 | | | P15814 | | | | | *IGLL1 | | Q9NRU3 | | CNNM1 |
| P68371 | | | TBB4B | | | P68371 | | | TBB4B | | | P19961 | | | | | AMY2B | | Q96G74 | | OTUD5 |
| A0M8Q6 | | | *IGLC7 | | | A0M8Q6 | | | *IGLC7 | | | P0DTE7 | | | | | AMY1B | |  | |  |
| P08779 | | | K1C16 | | | P08779 | | | K1C16 | | | P04746 | | | | | AMYP | |  | |  |
| A0A0B4J1V0 | | | HV315 | | | A0A0B4J1V0 | | | HV315 | | | P04745 | | | | | AMY1A | |  | |  |
| Q01469 | | | FABP5 | | | Q01469 | | | FABP5 | | | P0DTE8 | | | | | AMY1C | |  | |  |
| A0A0C4DH25 | | | KVD20 | | | A0A0C4DH25 | | | KVD20 | | | Q00796 | | | | | DHSO | |  | |  |
| Q6S8J3 | | | POTEE | | | Q6S8J3 | | | POTEE | | | O60664 | | | | | PLIN3 | |  | |  |
| P00747 | | | PLMN | | | P00747 | | | PLMN | | | P01742 | | | | | HV169 | |  | |  |
| P31947 | | | 1433S | | | P31947 | | | 1433S | | | A0A0B4J2H0 | | | | | HV69D | |  | |  |
| Q9UBG3 | | | CRNN | | | Q9UBG3 | | | CRNN | | | P0DOX3 | | | | | *IGD | |  | |  |
| A0A0B4J1Y9 | | | HV372 | | | A0A0B4J1Y9 | | | HV372 | | | A0A075B6I9 | | | | | LV746 | |  | |  |
| P28325 | | | CYTD | | | P28325 | | | CYTD | | | P04211 | | | | | LV743 | |  | |  |
| Q9UBC9 | | | SPRR3 | | | Q9UBC9 | | | SPRR3 | | | A0A075B6H8 | | | | | KVD42 | |  | |  |
| A0A0A0MRZ8 | | | KVD11 | | | A0A0A0MRZ8 | | | KVD11 | | | A0A075B6J9 | | | | | LV218 | |  | |  |
| P04433 | | | KV311 | | | P04433 | | | KV311 | | | A0A0B4J1U7 | | | | | HV601 | |  | |  |
| P01743 | | | HV146 | | | P01743 | | | HV146 | | | P37837 | | | | | TALDO | |  | |  |
| A2NJV5 | | | KV229 | | | A2NJV5 | | | KV229 | | | A0A0A0MT36 | | | | | KVD21 | |  | |  |
| A0A075B6S2 | | | KVD29 | | | A0A075B6S2 | | | KVD29 | | | A0A0C4DH24 | | | | | KV621 | |  | |  |
| A0A0C4DH72 | | | KV106 | | | A0A0C4DH72 | | | KV106 | | | P13639 | | | | | EF2 | |  | |  |
| Q06323 | | | PSME1 | | | Q06323 | | | PSME1 | | | P01701 | | | | | LV151 | |  | |  |
| O15143 | | | ARC1B | | | O15143 | | | ARC1B | | | A0A075B6I4 | | | | | LVX54 | |  | |  |
| A0A075B6S9 | | | KV137 | | | A0A075B6S9 | | | KV137 | | | P01721 | | | | | LV657 | |  | |  |
| P0DSN7 | | | KVD37 | | | P0DSN7 | | | KVD37 | | | Q9BUP0 | | | | | EFHD1 | |  | |  |
| A0A0C4DH42 | | | HV366 | | | A0A0C4DH42 | | | HV366 | | | Q96C19 | | | | | EFHD2 | |  | |  |
| A0A0C4DH34 | | | HV428 | | | A0A0C4DH34 | | | HV428 | | | A0A075B6Q5 | | | | | HV364 | |  | |  |
| Q08431 | | | MFGM | | | Q08431 | | | MFGM | | | Q99574 | | | | | NEUS | |  | |  |
| O00764 | | | PDXK | | | O00764 | | | PDXK | | | O00391 | | | | | QSOX1 | |  | |  |
| P00441 | | | SODC | | | P00441 | | | SODC | | | P01703 | | | | | LV140 | |  | |  |
| A0A0C4DH29 | | | HV103 | | | A0A0C4DH29 | | | HV103 | | | P23526 | | | | | SAHH | |  | |  |
| Q15293 | | | RCN1 | | | Q15293 | | | RCN1 | | | P25786 | | | | | PSA1 | |  | |  |
| P55064 | | | AQP5 | | | P55064 | | | AQP5 | | | O43548 | | | | | TGM5 | |  | |  |
| A0A075B6I0 | | | LV861 | | | A0A075B6I0 | | | LV861 | | | Q9Y6U3 | | | | | ADSV | |  | |  |
| P01040 | | | CYTA | | | P01040 | | | CYTA | | | Q9UBZ9 | | | | | REV1 | |  | |  |
| P04179 | | | SODM | | | P04179 | | | SODM | | | Q8WXS5 | | | | | CCG8 | |  | |  |
| P15814 | | | *IGLL1 | | | P15814 | | | *IGLL1 | | | O14990 | | | | | IPP2C | |  | |  |
| P19961 | | | AMY2B | | | P19961 | | | AMY2B | | | Q15782 | | | | | CH3L2 | |  | |  |
| P0DTE7 | | | AMY1B | | | P0DTE7 | | | AMY1B | | | P23284 | | | | | PPIB | |  | |  |
| P04746 | | | AMYP | | | P04746 | | | AMYP | | | Q7Z572 | | | | | SPT21 | |  | |  |
| P04745 | | | AMY1A | | | P04745 | | | AMY1A | | | Q15365 | | | | | PCBP1 | |  | |  |
| P0DTE8 | | | AMY1C | | | P0DTE8 | | | AMY1C | | | Q15366 | | | | | PCBP2 | |  | |  |
| Q00796 | | | DHSO | | | Q00796 | | | DHSO | | | P57721 | | | | | PCBP3 | |  | |  |
| O60664 | | | PLIN3 | | | O60664 | | | PLIN3 | | | P57723 | | | | | PCBP4 | |  | |  |
| P0DOX3 | | | *IGD | | | P0DOX3 | | | *IGD | | | Q7Z614 | | | | | SNX20 | |  | |  |
| A0A075B6I9 | | | LV746 | | | A0A075B6I9 | | | LV746 | | | A6NKD9 | | | | | CC85C | |  | |  |
| P04211 | | | LV743 | | | P04211 | | | LV743 | | | Q13315 | | | | | ATM | |  | |  |
| A0A075B6H8 | | | KVD42 | | | A0A075B6H8 | | | KVD42 | | | Q14624 | | | | | ITIH4 | |  | |  |
| A0A075B6J9 | | | LV218 | | | A0A075B6J9 | | | LV218 | | | P46459 | | | | | NSF | |  | |  |
| A0A0B4J1U7 | | | HV601 | | | A0A0B4J1U7 | | | HV601 | | | Q9UBQ7 | | | | | GRHPR | |  | |  |
| P37837 | | | TALDO | | | P37837 | | | TALDO | | | P15104 | | | | | GLNA | |  | |  |
| A0A0A0MT36 | | | KVD21 | | | A0A0A0MT36 | | | KVD21 | | | Q9NZJ7 | | | | | MTCH1 | |  | |  |
| A0A0C4DH24 | | | KV621 | | | A0A0C4DH24 | | | KV621 | | | Q86UL8 | | | | | MAGI2 | |  | |  |
| P13639 | | | EF2 | | | P13639 | | | EF2 | | | Q9Y6X0 | | | | | SETBP | |  | |  |
| P01701 | | | LV151 | | | P01701 | | | LV151 | | | Q8WUM4 | | | | | PDC6I | |  | |  |
| A0A075B6I4 | | | LVX54 | | | A0A075B6I4 | | | LVX54 | | | P60900 | | | | | PSA6 | |  | |  |
| P01721 | | | LV657 | | | P01721 | | | LV657 | | | Q6ZS81 | | | | | WDFY4 | |  | |  |
| Q9BUP0 | | | EFHD1 | | | Q9BUP0 | | | EFHD1 | | | Q96T58 | | | | | MINT | |  | |  |
| Q96C19 | | | EFHD2 | | | Q96C19 | | | EFHD2 | | | Q13608 | | | | | PEX6 | |  | |  |
| A0A075B6Q5 | | | HV364 | | | A0A075B6Q5 | | | HV364 | | | Q9HBH5 | | | | | RDH14 | |  | |  |
| Q9UKY7 | | | CDV3 | | | Q9UKY7 | | | CDV3 | | | O95969 | | | | | SG1D2 | |  | |  |
| Q99574 | | | NEUS | | | Q99574 | | | NEUS | | | Q9NRU3 | | | | | CNNM1 | |  | |  |
| O00391 | | | QSOX1 | | | O00391 | | | QSOX1 | | | Q6ZN18 | | | | | AEBP2 | |  | |  |
| P01703 | | | LV140 | | | P01703 | | | LV140 | | | P02654 | | | | | APOC1 | |  | |  |
| P23526 | | | SAHH | | | P23526 | | | SAHH | | |  | | | | |  | |  | |  |
| P25786 | | | PSA1 | | | P25786 | | | PSA1 | | |  | | | | |  | |  | |  |
| O43548 | | | TGM5 | | | O43548 | | | TGM5 | | |  | | | | |  | |  | |  |
| Q9Y6U3 | | | ADSV | | | Q9Y6U3 | | | ADSV | | |  | | | | |  | |  | |  |
| Q9UBZ9 | | | REV1 | | | Q9UBZ9 | | | REV1 | | |  | | | | |  | |  | |  |
| Q8WXS5 | | | CCG8 | | | Q8WXS5 | | | CCG8 | | |  | | | | |  | |  | |  |
| O14990 | | | IPP2C | | | O14990 | | | IPP2C | | |  | | | | |  | |  | |  |
| Q15782 | | | CH3L2 | | | Q15782 | | | CH3L2 | | |  | | | | |  | |  | |  |
| P23284 | | | PPIB | | | P23284 | | | PPIB | | |  | | | | |  | |  | |  |
| Q7Z572 | | | SPT21 | | | Q7Z572 | | | SPT21 | | |  | | | | |  | |  | |  |
| Q15365 | | | PCBP1 | | | Q15365 | | | PCBP1 | | |  | | | | |  | |  | |  |
| Q15366 | | | PCBP2 | | | Q15366 | | | PCBP2 | | |  | | | | |  | |  | |  |
| P57721 | | | PCBP3 | | | P57721 | | | PCBP3 | | |  | | | | |  | |  | |  |
| P57723 | | | PCBP4 | | | P57723 | | | PCBP4 | | |  | | | | |  | |  | |  |
| Q7Z614 | | | SNX20 | | | Q7Z614 | | | SNX20 | | |  | | | | |  | |  | |  |
| A6NKD9 | | | CC85C | | | A6NKD9 | | | CC85C | | |  | | | | |  | |  | |  |
| Q13315 | | | ATM | | | Q13315 | | | ATM | | |  | | | | |  | |  | |  |
| Q14624 | | | ITIH4 | | | Q14624 | | | ITIH4 | | |  | | | | |  | |  | |  |
| P46459 | | | NSF | | | P46459 | | | NSF | | |  | | | | |  | |  | |  |
| Q9UBQ7 | | | GRHPR | | | Q9UBQ7 | | | GRHPR | | |  | | | | |  | |  | |  |
| P15104 | | | GLNA | | | P15104 | | | GLNA | | |  | | | | |  | |  | |  |
| Q9NZJ7 | | | MTCH1 | | | Q9NZJ7 | | | MTCH1 | | |  | | | | |  | |  | |  |
| Q86UL8 | | | MAGI2 | | | Q86UL8 | | | MAGI2 | | |  | | | | |  | |  | |  |
| Q9Y6X0 | | | SETBP | | | Q9Y6X0 | | | SETBP | | |  | | | | |  | |  | |  |
| Q8WUM4 | | | PDC6I | | | Q8WUM4 | | | PDC6I | | |  | | | | |  | |  | |  |
| P60900 | | | PSA6 | | | P60900 | | | PSA6 | | |  | | | | |  | |  | |  |
| Q6ZS81 | | | WDFY4 | | | Q6ZS81 | | | WDFY4 | | |  | | | | |  | |  | |  |
| Q96T58 | | | MINT | | | Q96T58 | | | MINT | | |  | | | | |  | |  | |  |
| Q13608 | | | PEX6 | | | Q13608 | | | PEX6 | | |  | | | | |  | |  | |  |
| Q9HBH5 | | | RDH14 | | | Q9HBH5 | | | RDH14 | | |  | | | | |  | |  | |  |
| O95969 | | | SG1D2 | | | O95969 | | | SG1D2 | | |  | | | | |  | |  | |  |
| Q9NRU3 | | | CNNM1 | | | Q9NRU3 | | | CNNM1 | | |  | | | | |  | |  | |  |
| Q6ZN18 | | | AEBP2 | | | Q6ZN18 | | | AEBP2 | | |  | | | | |  | |  | |  |
| P02654 | | | APOC1 | | | P02654 | | | APOC1 | | |  | | | | |  | |  | |  |
| P01742 | | | HV169 | | |  | | |  | | |  | | | | |  | |  | |  |
| A0A0B4J2H0 | | | HV69D | | |  | | |  | | |  | | | | |  | |  | |  |
| Q96G74 | | | OTUD5 | | |  | | |  | | |  | | | | |  | |  | |  |
|  | | |  | | |  | | |  | | |  | | | | |  | |  | |  |
| * = Immunoglobulins | | | | | |  | | |  | | |  | | | | |  | |  | |  |
| Unique Proteins | | | | | | | | | | | | | | | |  |  |  |  |  |  |
| SS | | | | | MCT | | | | | | SCL | | | | |  |  |  |  |  |  |
| Total IDs | | 36 | | | Total IDs | | | 2 | | | Total IDs | | | 1 | |  |  |  |  |  |  |
| Uniprot | | Protein ID | | | Uniprot | | | Protein ID | | | Uniprot | | | Protein ID | |  |  |  |  |  |  |
| P01860 | | *IGHG3 | | | A0A0B4J2H0 | | | HV69D | | | Q96G74 | | | OTUD5 | |  |  |  |  |  |  |
| Q01082 | | SPTB2 | | | P01742 | | | HV169 | | |  | | |  | |  |  |  |  |  |  |
| P50502 | | F10A1 | | |  | | |  | | |  | | |  | |  |  |  |  |  |  |
| P08238 | | HS90B | | |  | | |  | | |  | | |  | |  |  |  |  |  |  |
| P28066 | | PSA5 | | |  | | |  | | |  | | |  | |  |  |  |  |  |  |
| Q15056 | | IF4H | | |  | | |  | | |  | | |  | |  |  |  |  |  |  |
| P11216 | | PYGB | | |  | | |  | | |  | | |  | |  |  |  |  |  |  |
| O00764 | | PDXK | | |  | | |  | | |  | | |  | |  |  |  |  |  |  |
| A0A075B6S2 | | KVD29 | | |  | | |  | | |  | | |  | |  |  |  |  |  |  |
| P17655 | | CAN2 | | |  | | |  | | |  | | |  | |  |  |  |  |  |  |
| Q99832 | | TCPH | | |  | | |  | | |  | | |  | |  |  |  |  |  |  |
| Q7Z4S6 | | KI21A | | |  | | |  | | |  | | |  | |  |  |  |  |  |  |
| O00534 | | VMA5A | | |  | | |  | | |  | | |  | |  |  |  |  |  |  |
| P36955 | | PEDF | | |  | | |  | | |  | | |  | |  |  |  |  |  |  |
| Q8NFI4 | | F10A5 | | |  | | |  | | |  | | |  | |  |  |  |  |  |  |
| A2NJV5 | | KV229 | | |  | | |  | | |  | | |  | |  |  |  |  |  |  |
| O95834 | | EMAL2 | | |  | | |  | | |  | | |  | |  |  |  |  |  |  |
| Q07020 | | RL18 | | |  | | |  | | |  | | |  | |  |  |  |  |  |  |
| A0A286YF58 | | TM271 | | |  | | |  | | |  | | |  | |  |  |  |  |  |  |
| Q86YR7 | | MF2L2 | | |  | | |  | | |  | | |  | |  |  |  |  |  |  |
| Q9H008 | | LHPP | | |  | | |  | | |  | | |  | |  |  |  |  |  |  |
| Q9H6S3 | | ES8L2 | | |  | | |  | | |  | | |  | |  |  |  |  |  |  |
| Q9NY33 | | DPP3 | | |  | | |  | | |  | | |  | |  |  |  |  |  |  |
| Q8TCD5 | | NT5C | | |  | | |  | | |  | | |  | |  |  |  |  |  |  |
| P35606 | | COPB2 | | |  | | |  | | |  | | |  | |  |  |  |  |  |  |
| P48643 | | TCPE | | |  | | |  | | |  | | |  | |  |  |  |  |  |  |
| O94808 | | GFPT2 | | |  | | |  | | |  | | |  | |  |  |  |  |  |  |
| P25398 | | RS12 | | |  | | |  | | |  | | |  | |  |  |  |  |  |  |
| O15144 | | ARPC2 | | |  | | |  | | |  | | |  | |  |  |  |  |  |  |
| P52788 | | SPSY | | |  | | |  | | |  | | |  | |  |  |  |  |  |  |
| Q9UQ80 | | PA2G4 | | |  | | |  | | |  | | |  | |  |  |  |  |  |  |
| Q06210 | | GFPT1 | | |  | | |  | | |  | | |  | |  |  |  |  |  |  |
| P01040 | | CYTA | | |  | | |  | | |  | | |  | |  |  |  |  |  |  |
| Q8IZP2 | | ST134 | | |  | | |  | | |  | | |  | |  |  |  |  |  |  |
| Q9UKY7 | | CDV3 | | |  | | |  | | |  | | |  | |  |  |  |  |  |  |
| P06576 | | ATPB | | |  | | |  | | |  | | |  | |  |  |  |  |  |  |
|  | |  | | |  | | |  | | |  | | |  | |  |  |  |  |  |  |
| * = Immunoglobulins | | | | |  | | |  | | |  | | |  | |  |  |  |  |  |  |
| Shared Proteins | | | | | | | | | | | | | | | | | | | |  |  |
| SS, MCT, & SCL | | | | SS & MCT | | | | | | SS & SCL | | | | | MCT & SCL | | | | |  |  |
| Total IDs | 386 | | | Total IDs | | | 60 | | | Total IDs | | | 0 | | Total IDs | | | 0 | |  |  |
| Uniprot | Protein ID | | | Uniprot | | | Protein ID | | | Uniprot | | | Protein ID | | Uniprot | | | Protein ID | |  |  |
| P09211 | GSTP1 | | | P01042 | | | KNG1 | | |  | | |  | |  | | |  | |  |  |
| P08758 | ANXA5 | | | P13639 | | | EF2 | | |  | | |  | |  | | |  | |  |  |
| P09651 | ROA1 | | | Q99933 | | | BAG1 | | |  | | |  | |  | | |  | |  |  |
| O60814 | H2B1K | | | P40939 | | | ECHA | | |  | | |  | |  | | |  | |  |  |
| P30101 | PDIA3 | | | A0A075B6P5 | | | KV228 | | |  | | |  | |  | | |  | |  |  |
| P02675 | FIBB | | | Q8TD06 | | | AGR3 | | |  | | |  | |  | | |  | |  |  |
| P01594 | KV133 | | | O75882 | | | ATRN | | |  | | |  | |  | | |  | |  |  |
| Q6NT89 | TRNP1 | | | P17987 | | | TCPA | | |  | | |  | |  | | |  | |  |  |
| P57053 | H2BFS | | | P06744 | | | G6PI | | |  | | |  | |  | | |  | |  |  |
| P48634 | PRC2A | | | P27348 | | | 1433T | | |  | | |  | |  | | |  | |  |  |
| P05155 | IC1 | | | P14625 | | | ENPL | | |  | | |  | |  | | |  | |  |  |
| P0CG47 | UBB | | | O95969 | | | SG1D2 | | |  | | |  | |  | | |  | |  |  |
| A0A0C4DH69 | KV109 | | | O60437 | | | PEPL | | |  | | |  | |  | | |  | |  |  |
| Q99877 | H2B1N | | | Q9HAP2 | | | MLXIP | | |  | | |  | |  | | |  | |  |  |
| A0A0A0MRZ8 | KVD11 | | | Q04695 | | | K1C17 | | |  | | |  | |  | | |  | |  |  |
| P13489 | RINI | | | Q7Z572 | | | SPT21 | | |  | | |  | |  | | |  | |  |  |
| P0DOX5 | *IGG1 | | | P52566 | | | GDIR2 | | |  | | |  | |  | | |  | |  |  |
| Q8WVQ1 | CANT1 | | | P15104 | | | GLNA | | |  | | |  | |  | | |  | |  |  |
| P19823 | ITIH2 | | | O14990 | | | IPP2C | | |  | | |  | |  | | |  | |  |  |
| P06899 | H2B1J | | | Q9BXJ3 | | | C1QT4 | | |  | | |  | |  | | |  | |  |  |
| P01859 | *IGHG2 | | | Q8WXS5 | | | CCG8 | | |  | | |  | |  | | |  | |  |  |
| P21980 | TGM2 | | | P00326 | | | ADH1G | | |  | | |  | |  | | |  | |  |  |
| Q96C19 | EFHD2 | | | Q9BS40 | | | LXN | | |  | | |  | |  | | |  | |  |  |
| Q9BQE3 | TBA1C | | | O00151 | | | PDLI1 | | |  | | |  | |  | | |  | |  |  |
| P14618 | KPYM | | | P01615 | | | KVD28 | | |  | | |  | |  | | |  | |  |  |
| P34096 | RNAS4 | | | A0A0C4DH34 | | | HV428 | | |  | | |  | |  | | |  | |  |  |
| P68104 | EF1A1 | | | Q9Y490 | | | TLN1 | | |  | | |  | |  | | |  | |  |  |
| P06753 | TPM3 | | | Q6ZN18 | | | AEBP2 | | |  | | |  | |  | | |  | |  |  |
| O14950 | ML12B | | | P09525 | | | ANXA4 | | |  | | |  | |  | | |  | |  |  |
| Q14515 | SPRL1 | | | P25786 | | | PSA1 | | |  | | |  | |  | | |  | |  |  |
| P09429 | HMGB1 | | | P07437 | | | TBB5 | | |  | | |  | |  | | |  | |  |  |
| Q86UT5 | NHRF4 | | | Q00610 | | | CLH1 | | |  | | |  | |  | | |  | |  |  |
| P04264 | K2C1 | | | P19827 | | | ITIH1 | | |  | | |  | |  | | |  | |  |  |
| P13647 | K2C5 | | | Q86UL8 | | | MAGI2 | | |  | | |  | |  | | |  | |  |  |
| P02774 | VTDB | | | P01718 | | | LV327 | | |  | | |  | |  | | |  | |  |  |
| P00450 | CERU | | | O15143 | | | ARC1B | | |  | | |  | |  | | |  | |  |  |
| Q00796 | DHSO | | | P17812 | | | PYRG1 | | |  | | |  | |  | | |  | |  |  |
| Q96S96 | PEBP4 | | | P02654 | | | APOC1 | | |  | | |  | |  | | |  | |  |  |
| P80748 | LV321 | | | P31943 | | | HNRH1 | | |  | | |  | |  | | |  | |  |  |
| P01008 | ANT3 | | | P07327 | | | ADH1A | | |  | | |  | |  | | |  | |  |  |
| P19961 | AMY2B | | | Q96T58 | | | MINT | | |  | | |  | |  | | |  | |  |  |
| P02647 | APOA1 | | | P00747 | | | PLMN | | |  | | |  | |  | | |  | |  |  |
| Q01469 | FABP5 | | | P55064 | | | AQP5 | | |  | | |  | |  | | |  | |  |  |
| P57721 | PCBP3 | | | Q14118 | | | DAG1 | | |  | | |  | |  | | |  | |  |  |
| P06310 | KV230 | | | P35579 | | | MYH9 | | |  | | |  | |  | | |  | |  |  |
| P40121 | CAPG | | | A0M8Q6 | | | *IGLC7 | | |  | | |  | |  | | |  | |  |  |
| P13797 | PLST | | | A0A087WSZ0 | | | KVD08 | | |  | | |  | |  | | |  | |  |  |
| Q96KP4 | CNDP2 | | | Q9Y6U3 | | | ADSV | | |  | | |  | |  | | |  | |  |  |
| P63267 | ACTH | | | Q14624 | | | ITIH4 | | |  | | |  | |  | | |  | |  |  |
| P04259 | K2C6B | | | P40306 | | | PSB10 | | |  | | |  | |  | | |  | |  |  |
| Q9BTM1 | H2AJ | | | A0A0C4DH29 | | | HV103 | | |  | | |  | |  | | |  | |  |  |
| P0DMV8 | HS71A | | | Q96JH8 | | | RADIL | | |  | | |  | |  | | |  | |  |  |
| P01703 | LV140 | | | P27797 | | | CALR | | |  | | |  | |  | | |  | |  |  |
| Q06323 | PSME1 | | | P01861 | | | *IGHG4 | | |  | | |  | |  | | |  | |  |  |
| P32119 | PRDX2 | | | Q8WUM4 | | | PDC6I | | |  | | |  | |  | | |  | |  |  |
| P30041 | PRDX6 | | | Q99538 | | | LGMN | | |  | | |  | |  | | |  | |  |  |
| P11142 | HSP7C | | | P19652 | | | A1AG2 | | |  | | |  | |  | | |  | |  |  |
| P12273 | PIP | | | P55795 | | | HNRH2 | | |  | | |  | |  | | |  | |  |  |
| Q13228 | SBP1 | | | Q96FW1 | | | OTUB1 | | |  | | |  | |  | | |  | |  |  |
| P63104 | 1433Z | | | P50395 | | | GDIB | | |  | | |  | |  | | |  | |  |  |
| P04746 | AMYP | | |  | | |  | | |  | | |  | |  | | |  | |  |  |
| P22626 | ROA2 | | |  | | |  | | |  | | |  | |  | | |  | |  |  |
| P01782 | HV309 | | |  | | |  | | |  | | |  | |  | | |  | |  |  |
| A0A0B4J1V0 | HV315 | | |  | | |  | | |  | | |  | |  | | |  | |  |  |
| P03950 | ANGI | | |  | | |  | | |  | | |  | |  | | |  | |  |  |
| P15311 | EZRI | | |  | | |  | | |  | | |  | |  | | |  | |  |  |
| Q15365 | PCBP1 | | |  | | |  | | |  | | |  | |  | | |  | |  |  |
| P0DP07 | HV431 | | |  | | |  | | |  | | |  | |  | | |  | |  |  |
| Q96DA0 | ZG16B | | |  | | |  | | |  | | |  | |  | | |  | |  |  |
| P40926 | MDHM | | |  | | |  | | |  | | |  | |  | | |  | |  |  |
| P01876 | *IGHA1 | | |  | | |  | | |  | | |  | |  | | |  | |  |  |
| P47895 | AL1A3 | | |  | | |  | | |  | | |  | |  | | |  | |  |  |
| P14550 | AK1A1 | | |  | | |  | | |  | | |  | |  | | |  | |  |  |
| P98088 | MUC5A | | |  | | |  | | |  | | |  | |  | | |  | |  |  |
| P0C0S8 | H2A1 | | |  | | |  | | |  | | |  | |  | | |  | |  |  |
| P01034 | CYTC | | |  | | |  | | |  | | |  | |  | | |  | |  |  |
| A0A0A0MS15 | HV349 | | |  | | |  | | |  | | |  | |  | | |  | |  |  |
| P04179 | SODM | | |  | | |  | | |  | | |  | |  | | |  | |  |  |
| P01593 | KVD33 | | |  | | |  | | |  | | |  | |  | | |  | |  |  |
| P01023 | A2MG | | |  | | |  | | |  | | |  | |  | | |  | |  |  |
| P02671 | FIBA | | |  | | |  | | |  | | |  | |  | | |  | |  |  |
| Q7L7L0 | H2A3 | | |  | | |  | | |  | | |  | |  | | |  | |  |  |
| A0A0C4DH32 | HV320 | | |  | | |  | | |  | | |  | |  | | |  | |  |  |
| A0A0C4DH25 | KVD20 | | |  | | |  | | |  | | |  | |  | | |  | |  |  |
| A0A075B6S6 | KVD30 | | |  | | |  | | |  | | |  | |  | | |  | |  |  |
| P16104 | H2AX | | |  | | |  | | |  | | |  | |  | | |  | |  |  |
| Q7Z614 | SNX20 | | |  | | |  | | |  | | |  | |  | | |  | |  |  |
| P00441 | SODC | | |  | | |  | | |  | | |  | |  | | |  | |  |  |
| O43707 | ACTN4 | | |  | | |  | | |  | | |  | |  | | |  | |  |  |
| Q8NBJ4 | GOLM1 | | |  | | |  | | |  | | |  | |  | | |  | |  |  |
| P46940 | IQGA1 | | |  | | |  | | |  | | |  | |  | | |  | |  |  |
| P01714 | LV319 | | |  | | |  | | |  | | |  | |  | | |  | |  |  |
| Q15782 | CH3L2 | | |  | | |  | | |  | | |  | |  | | |  | |  |  |
| P37837 | TALDO | | |  | | |  | | |  | | |  | |  | | |  | |  |  |
| Q09666 | AHNK | | |  | | |  | | |  | | |  | |  | | |  | |  |  |
| P62258 | 1433E | | |  | | |  | | |  | | |  | |  | | |  | |  |  |
| P01871 | *IGHM | | |  | | |  | | |  | | |  | |  | | |  | |  |  |
| P25789 | PSA4 | | |  | | |  | | |  | | |  | |  | | |  | |  |  |
| P07339 | CATD | | |  | | |  | | |  | | |  | |  | | |  | |  |  |
| Q32P51 | RA1L2 | | |  | | |  | | |  | | |  | |  | | |  | |  |  |
| P35908 | K22E | | |  | | |  | | |  | | |  | |  | | |  | |  |  |
| Q9NSB4 | KRT82 | | |  | | |  | | |  | | |  | |  | | |  | |  |  |
| P05787 | K2C8 | | |  | | |  | | |  | | |  | |  | | |  | |  |  |
| P22314 | UBA1 | | |  | | |  | | |  | | |  | |  | | |  | |  |  |
| P01780 | HV307 | | |  | | |  | | |  | | |  | |  | | |  | |  |  |
| Q93077 | H2A1C | | |  | | |  | | |  | | |  | |  | | |  | |  |  |
| P00966 | ASSY | | |  | | |  | | |  | | |  | |  | | |  | |  |  |
| A0A075B6R9 | KVD24 | | |  | | |  | | |  | | |  | |  | | |  | |  |  |
| P02766 | TTHY | | |  | | |  | | |  | | |  | |  | | |  | |  |  |
| Q7Z406 | MYH14 | | |  | | |  | | |  | | |  | |  | | |  | |  |  |
| P08603 | CFAH | | |  | | |  | | |  | | |  | |  | | |  | |  |  |
| P00558 | PGK1 | | |  | | |  | | |  | | |  | |  | | |  | |  |  |
| P62937 | PPIA | | |  | | |  | | |  | | |  | |  | | |  | |  |  |
| P01011 | AACT | | |  | | |  | | |  | | |  | |  | | |  | |  |  |
| P62736 | ACTA | | |  | | |  | | |  | | |  | |  | | |  | |  |  |
| Q9UGM3 | DMBT1 | | |  | | |  | | |  | | |  | |  | | |  | |  |  |
| P00751 | CFAB | | |  | | |  | | |  | | |  | |  | | |  | |  |  |
| P04083 | ANXA1 | | |  | | |  | | |  | | |  | |  | | |  | |  |  |
| O00391 | QSOX1 | | |  | | |  | | |  | | |  | |  | | |  | |  |  |
| P0C0L4 | CO4A | | |  | | |  | | |  | | |  | |  | | |  | |  |  |
| O00299 | CLIC1 | | |  | | |  | | |  | | |  | |  | | |  | |  |  |
| P31025 | LCN1 | | |  | | |  | | |  | | |  | |  | | |  | |  |  |
| P02511 | CRYAB | | |  | | |  | | |  | | |  | |  | | |  | |  |  |
| A0A075B6K4 | LV310 | | |  | | |  | | |  | | |  | |  | | |  | |  |  |
| Q99878 | H2A1J | | |  | | |  | | |  | | |  | |  | | |  | |  |  |
| P62807 | H2B1C | | |  | | |  | | |  | | |  | |  | | |  | |  |  |
| Q01518 | CAP1 | | |  | | |  | | |  | | |  | |  | | |  | |  |  |
| P03973 | SLPI | | |  | | |  | | |  | | |  | |  | | |  | |  |  |
| Q9HC38 | GLOD4 | | |  | | |  | | |  | | |  | |  | | |  | |  |  |
| P17931 | LEG3 | | |  | | |  | | |  | | |  | |  | | |  | |  |  |
| Q13217 | DNJC3 | | |  | | |  | | |  | | |  | |  | | |  | |  |  |
| Q9NRI5 | DISC1 | | |  | | |  | | |  | | |  | |  | | |  | |  |  |
| P19021 | AMD | | |  | | |  | | |  | | |  | |  | | |  | |  |  |
| P07737 | PROF1 | | |  | | |  | | |  | | |  | |  | | |  | |  |  |
| Q06830 | PRDX1 | | |  | | |  | | |  | | |  | |  | | |  | |  |  |
| A0A0B4J1U7 | HV601 | | |  | | |  | | |  | | |  | |  | | |  | |  |  |
| A0A0C4DH73 | KV112 | | |  | | |  | | |  | | |  | |  | | |  | |  |  |
| Q8N257 | H2B3B | | |  | | |  | | |  | | |  | |  | | |  | |  |  |
| Q8IUE6 | H2A2B | | |  | | |  | | |  | | |  | |  | | |  | |  |  |
| P23527 | H2B1O | | |  | | |  | | |  | | |  | |  | | |  | |  |  |
| P0C0L5 | CO4B | | |  | | |  | | |  | | |  | |  | | |  | |  |  |
| P21964 | COMT | | |  | | |  | | |  | | |  | |  | | |  | |  |  |
| A0A075B6H8 | KVD42 | | |  | | |  | | |  | | |  | |  | | |  | |  |  |
| P09467 | F16P1 | | |  | | |  | | |  | | |  | |  | | |  | |  |  |
| Q14764 | MVP | | |  | | |  | | |  | | |  | |  | | |  | |  |  |
| P06727 | APOA4 | | |  | | |  | | |  | | |  | |  | | |  | |  |  |
| Q71U36 | TBA1A | | |  | | |  | | |  | | |  | |  | | |  | |  |  |
| P17858 | PFKAL | | |  | | |  | | |  | | |  | |  | | |  | |  |  |
| P02750 | A2GL | | |  | | |  | | |  | | |  | |  | | |  | |  |  |
| Q9Y6X0 | SETBP | | |  | | |  | | |  | | |  | |  | | |  | |  |  |
| P29401 | TKT | | |  | | |  | | |  | | |  | |  | | |  | |  |  |
| Q6SPF0 | SAMD1 | | |  | | |  | | |  | | |  | |  | | |  | |  |  |
| P30085 | KCY | | |  | | |  | | |  | | |  | |  | | |  | |  |  |
| Q08380 | LG3BP | | |  | | |  | | |  | | |  | |  | | |  | |  |  |
| P02652 | APOA2 | | |  | | |  | | |  | | |  | |  | | |  | |  |  |
| A0A0B4J2D9 | KVD13 | | |  | | |  | | |  | | |  | |  | | |  | |  |  |
| P02765 | FETUA | | |  | | |  | | |  | | |  | |  | | |  | |  |  |
| P28799 | GRN | | |  | | |  | | |  | | |  | |  | | |  | |  |  |
| P62979 | RS27A | | |  | | |  | | |  | | |  | |  | | |  | |  |  |
| P01024 | CO3 | | |  | | |  | | |  | | |  | |  | | |  | |  |  |
| P01033 | TIMP1 | | |  | | |  | | |  | | |  | |  | | |  | |  |  |
| P05090 | APOD | | |  | | |  | | |  | | |  | |  | | |  | |  |  |
| Q5QNW6 | H2B2F | | |  | | |  | | |  | | |  | |  | | |  | |  |  |
| Q99574 | NEUS | | |  | | |  | | |  | | |  | |  | | |  | |  |  |
| P61626 | LYSC | | |  | | |  | | |  | | |  | |  | | |  | |  |  |
| O43852 | CALU | | |  | | |  | | |  | | |  | |  | | |  | |  |  |
| Q6ZMY3 | SPOC1 | | |  | | |  | | |  | | |  | |  | | |  | |  |  |
| P40925 | MDHC | | |  | | |  | | |  | | |  | |  | | |  | |  |  |
| P0DP08 | HVD82 | | |  | | |  | | |  | | |  | |  | | |  | |  |  |
| P30740 | ILEU | | |  | | |  | | |  | | |  | |  | | |  | |  |  |
| Q9UBG3 | CRNN | | |  | | |  | | |  | | |  | |  | | |  | |  |  |
| Q08431 | MFGM | | |  | | |  | | |  | | |  | |  | | |  | |  |  |
| Q5VSP4 | LC1L1 | | |  | | |  | | |  | | |  | |  | | |  | |  |  |
| P13645 | K1C10 | | |  | | |  | | |  | | |  | |  | | |  | |  |  |
| P60660 | MYL6 | | |  | | |  | | |  | | |  | |  | | |  | |  |  |
| P98160 | PGBM | | |  | | |  | | |  | | |  | |  | | |  | |  |  |
| P01701 | LV151 | | |  | | |  | | |  | | |  | |  | | |  | |  |  |
| P0DMV9 | HS71B | | |  | | |  | | |  | | |  | |  | | |  | |  |  |
| P30838 | AL3A1 | | |  | | |  | | |  | | |  | |  | | |  | |  |  |
| Q9UL51 | HCN2 | | |  | | |  | | |  | | |  | |  | | |  | |  |  |
| P31946 | 1433B | | |  | | |  | | |  | | |  | |  | | |  | |  |  |
| P61769 | B2MG | | |  | | |  | | |  | | |  | |  | | |  | |  |  |
| Q9UBC9 | SPRR3 | | |  | | |  | | |  | | |  | |  | | |  | |  |  |
| Q9BRK5 | CAB45 | | |  | | |  | | |  | | |  | |  | | |  | |  |  |
| P08670 | VIME | | |  | | |  | | |  | | |  | |  | | |  | |  |  |
| P0DP09 | KV113 | | |  | | |  | | |  | | |  | |  | | |  | |  |  |
| A0A0C4DH38 | HV551 | | |  | | |  | | |  | | |  | |  | | |  | |  |  |
| Q8NCB2 | CAMKV | | |  | | |  | | |  | | |  | |  | | |  | |  |  |
| P49788 | TIG1 | | |  | | |  | | |  | | |  | |  | | |  | |  |  |
| P0DOX3 | *IGD | | |  | | |  | | |  | | |  | |  | | |  | |  |  |
| P46459 | NSF | | |  | | |  | | |  | | |  | |  | | |  | |  |  |
| P15814 | *IGLL1 | | |  | | |  | | |  | | |  | |  | | |  | |  |  |
| P68133 | ACTS | | |  | | |  | | |  | | |  | |  | | |  | |  |  |
| P60900 | PSA6 | | |  | | |  | | |  | | |  | |  | | |  | |  |  |
| P04217 | A1BG | | |  | | |  | | |  | | |  | |  | | |  | |  |  |
| Q13162 | PRDX4 | | |  | | |  | | |  | | |  | |  | | |  | |  |  |
| Q9Y5Z4 | HEBP2 | | |  | | |  | | |  | | |  | |  | | |  | |  |  |
| Q14697 | GANAB | | |  | | |  | | |  | | |  | |  | | |  | |  |  |
| P01717 | LV325 | | |  | | |  | | |  | | |  | |  | | |  | |  |  |
| P0DOX7 | IGK | | |  | | |  | | |  | | |  | |  | | |  | |  |  |
| P04004 | VTNC | | |  | | |  | | |  | | |  | |  | | |  | |  |  |
| Q99880 | H2B1L | | |  | | |  | | |  | | |  | |  | | |  | |  |  |
| P0DP06 | HVD34 | | |  | | |  | | |  | | |  | |  | | |  | |  |  |
| P81605 | DCD | | |  | | |  | | |  | | |  | |  | | |  | |  |  |
| P02538 | K2C6A | | |  | | |  | | |  | | |  | |  | | |  | |  |  |
| B9A064 | *IGLL5 | | |  | | |  | | |  | | |  | |  | | |  | |  |  |
| Q9UBZ9 | REV1 | | |  | | |  | | |  | | |  | |  | | |  | |  |  |
| P01611 | KVD12 | | |  | | |  | | |  | | |  | |  | | |  | |  |  |
| P13646 | K1C13 | | |  | | |  | | |  | | |  | |  | | |  | |  |  |
| P0DOY2 | *IGLC2 | | |  | | |  | | |  | | |  | |  | | |  | |  |  |
| O00462 | MANBA | | |  | | |  | | |  | | |  | |  | | |  | |  |  |
| A0A0C4DH72 | KV106 | | |  | | |  | | |  | | |  | |  | | |  | |  |  |
| P30044 | PRDX5 | | |  | | |  | | |  | | |  | |  | | |  | |  |  |
| P31151 | S10A7 | | |  | | |  | | |  | | |  | |  | | |  | |  |  |
| P01619 | KV320 | | |  | | |  | | |  | | |  | |  | | |  | |  |  |
| P18510 | IL1RA | | |  | | |  | | |  | | |  | |  | | |  | |  |  |
| P06312 | KV401 | | |  | | |  | | |  | | |  | |  | | |  | |  |  |
| P08729 | K2C7 | | |  | | |  | | |  | | |  | |  | | |  | |  |  |
| A0A075B6J9 | LV218 | | |  | | |  | | |  | | |  | |  | | |  | |  |  |
| P04211 | LV743 | | |  | | |  | | |  | | |  | |  | | |  | |  |  |
| Q6S8J3 | POTEE | | |  | | |  | | |  | | |  | |  | | |  | |  |  |
| P68871 | HBB | | |  | | |  | | |  | | |  | |  | | |  | |  |  |
| P16070 | CD44 | | |  | | |  | | |  | | |  | |  | | |  | |  |  |
| A0A075B6Q5 | HV364 | | |  | | |  | | |  | | |  | |  | | |  | |  |  |
| A6NKD9 | CC85C | | |  | | |  | | |  | | |  | |  | | |  | |  |  |
| O75874 | IDHC | | |  | | |  | | |  | | |  | |  | | |  | |  |  |
| Q04828 | AK1C1 | | |  | | |  | | |  | | |  | |  | | |  | |  |  |
| P01037 | CYTN | | |  | | |  | | |  | | |  | |  | | |  | |  |  |
| P0DTE7 | AMY1B | | |  | | |  | | |  | | |  | |  | | |  | |  |  |
| P04075 | ALDOA | | |  | | |  | | |  | | |  | |  | | |  | |  |  |
| A0A0C4DH24 | KV621 | | |  | | |  | | |  | | |  | |  | | |  | |  |  |
| P08779 | K1C16 | | |  | | |  | | |  | | |  | |  | | |  | |  |  |
| P02790 | HEMO | | |  | | |  | | |  | | |  | |  | | |  | |  |  |
| P60174 | TPIS | | |  | | |  | | |  | | |  | |  | | |  | |  |  |
| P0DP25 | CALM3 | | |  | | |  | | |  | | |  | |  | | |  | |  |  |
| P31947 | 1433S | | |  | | |  | | |  | | |  | |  | | |  | |  |  |
| P19105 | ML12A | | |  | | |  | | |  | | |  | |  | | |  | |  |  |
| P02545 | LMNA | | |  | | |  | | |  | | |  | |  | | |  | |  |  |
| A0A0C4DH42 | HV366 | | |  | | |  | | |  | | |  | |  | | |  | |  |  |
| P0DTE8 | AMY1C | | |  | | |  | | |  | | |  | |  | | |  | |  |  |
| P02533 | K1C14 | | |  | | |  | | |  | | |  | |  | | |  | |  |  |
| P01825 | HV459 | | |  | | |  | | |  | | |  | |  | | |  | |  |  |
| Q96KK5 | H2A1H | | |  | | |  | | |  | | |  | |  | | |  | |  |  |
| P25705 | ATPA | | |  | | |  | | |  | | |  | |  | | |  | |  |  |
| P01700 | LV147 | | |  | | |  | | |  | | |  | |  | | |  | |  |  |
| Q99954 | SMR3A | | |  | | |  | | |  | | |  | |  | | |  | |  |  |
| P37802 | TAGL2 | | |  | | |  | | |  | | |  | |  | | |  | |  |  |
| P68363 | TBA1B | | |  | | |  | | |  | | |  | |  | | |  | |  |  |
| P23528 | COF1 | | |  | | |  | | |  | | |  | |  | | |  | |  |  |
| P24844 | MYL9 | | |  | | |  | | |  | | |  | |  | | |  | |  |  |
| P01036 | CYTS | | |  | | |  | | |  | | |  | |  | | |  | |  |  |
| P00738 | HPT | | |  | | |  | | |  | | |  | |  | | |  | |  |  |
| Q13315 | ATM | | |  | | |  | | |  | | |  | |  | | |  | |  |  |
| O75083 | WDR1 | | |  | | |  | | |  | | |  | |  | | |  | |  |  |
| P06733 | ENOA | | |  | | |  | | |  | | |  | |  | | |  | |  |  |
| P20671 | H2A1D | | |  | | |  | | |  | | |  | |  | | |  | |  |  |
| P62277 | RS13 | | |  | | |  | | |  | | |  | |  | | |  | |  |  |
| P01591 | *IGJ | | |  | | |  | | |  | | |  | |  | | |  | |  |  |
| P11021 | BIP | | |  | | |  | | |  | | |  | |  | | |  | |  |  |
| P62987 | RL40 | | |  | | |  | | |  | | |  | |  | | |  | |  |  |
| P01833 | PIGR | | |  | | |  | | |  | | |  | |  | | |  | |  |  |
| P00338 | LDHA | | |  | | |  | | |  | | |  | |  | | |  | |  |  |
| Q15366 | PCBP2 | | |  | | |  | | |  | | |  | |  | | |  | |  |  |
| Q15181 | IPYR | | |  | | |  | | |  | | |  | |  | | |  | |  |  |
| P33778 | H2B1B | | |  | | |  | | |  | | |  | |  | | |  | |  |  |
| P07384 | CAN1 | | |  | | |  | | |  | | |  | |  | | |  | |  |  |
| P08582 | TRFM | | |  | | |  | | |  | | |  | |  | | |  | |  |  |
| P80303 | NUCB2 | | |  | | |  | | |  | | |  | |  | | |  | |  |  |
| Q9HBH5 | RDH14 | | |  | | |  | | |  | | |  | |  | | |  | |  |  |
| P25311 | ZA2G | | |  | | |  | | |  | | |  | |  | | |  | |  |  |
| P06396 | GELS | | |  | | |  | | |  | | |  | |  | | |  | |  |  |
| P16401 | H15 | | |  | | |  | | |  | | |  | |  | | |  | |  |  |
| Q6FI13 | H2A2A | | |  | | |  | | |  | | |  | |  | | |  | |  |  |
| P0DSN7 | KVD37 | | |  | | |  | | |  | | |  | |  | | |  | |  |  |
| Q13421 | MSLN | | |  | | |  | | |  | | |  | |  | | |  | |  |  |
| P50995 | ANX11 | | |  | | |  | | |  | | |  | |  | | |  | |  |  |
| P04908 | H2A1B | | |  | | |  | | |  | | |  | |  | | |  | |  |  |
| Q9NZJ7 | MTCH1 | | |  | | |  | | |  | | |  | |  | | |  | |  |  |
| O43548 | TGM5 | | |  | | |  | | |  | | |  | |  | | |  | |  |  |
| P02787 | TRFE | | |  | | |  | | |  | | |  | |  | | |  | |  |  |
| Q8TD19 | NEK9 | | |  | | |  | | |  | | |  | |  | | |  | |  |  |
| P06702 | S10A9 | | |  | | |  | | |  | | |  | |  | | |  | |  |  |
| P10909 | CLUS | | |  | | |  | | |  | | |  | |  | | |  | |  |  |
| P62805 | H4 | | |  | | |  | | |  | | |  | |  | | |  | |  |  |
| P07355 | ANXA2 | | |  | | |  | | |  | | |  | |  | | |  | |  |  |
| P07602 | SAP | | |  | | |  | | |  | | |  | |  | | |  | |  |  |
| P68032 | ACTC | | |  | | |  | | |  | | |  | |  | | |  | |  |  |
| P23284 | PPIB | | |  | | |  | | |  | | |  | |  | | |  | |  |  |
| Q7Z5P9 | MUC19 | | |  | | |  | | |  | | |  | |  | | |  | |  |  |
| P25815 | S100P | | |  | | |  | | |  | | |  | |  | | |  | |  |  |
| P52907 | CAZA1 | | |  | | |  | | |  | | |  | |  | | |  | |  |  |
| P01721 | LV657 | | |  | | |  | | |  | | |  | |  | | |  | |  |  |
| P13693 | TCTP | | |  | | |  | | |  | | |  | |  | | |  | |  |  |
| P22079 | PERL | | |  | | |  | | |  | | |  | |  | | |  | |  |  |
| P36952 | SPB5 | | |  | | |  | | |  | | |  | |  | | |  | |  |  |
| P04080 | CYTB | | |  | | |  | | |  | | |  | |  | | |  | |  |  |
| P04433 | KV311 | | |  | | |  | | |  | | |  | |  | | |  | |  |  |
| P30086 | PEBP1 | | |  | | |  | | |  | | |  | |  | | |  | |  |  |
| P55058 | PLTP | | |  | | |  | | |  | | |  | |  | | |  | |  |  |
| Q16378 | PROL4 | | |  | | |  | | |  | | |  | |  | | |  | |  |  |
| A0A075B6I9 | LV746 | | |  | | |  | | |  | | |  | |  | | |  | |  |  |
| P07858 | CATB | | |  | | |  | | |  | | |  | |  | | |  | |  |  |
| P52209 | 6PGD | | |  | | |  | | |  | | |  | |  | | |  | |  |  |
| P52565 | GDIR1 | | |  | | |  | | |  | | |  | |  | | |  | |  |  |
| P0CG48 | UBC | | |  | | |  | | |  | | |  | |  | | |  | |  |  |
| P08727 | K1C19 | | |  | | |  | | |  | | |  | |  | | |  | |  |  |
| P02768 | ALBU | | |  | | |  | | |  | | |  | |  | | |  | |  |  |
| Q6MZM9 | PRR27 | | |  | | |  | | |  | | |  | |  | | |  | |  |  |
| P28325 | CYTD | | |  | | |  | | |  | | |  | |  | | |  | |  |  |
| Q93079 | H2B1H | | |  | | |  | | |  | | |  | |  | | |  | |  |  |
| P26447 | S10A4 | | |  | | |  | | |  | | |  | |  | | |  | |  |  |
| P07237 | PDIA1 | | |  | | |  | | |  | | |  | |  | | |  | |  |  |
| Q99935 | PROL1 | | |  | | |  | | |  | | |  | |  | | |  | |  |  |
| P55072 | TERA | | |  | | |  | | |  | | |  | |  | | |  | |  |  |
| A0A0C4DH68 | KV224 | | |  | | |  | | |  | | |  | |  | | |  | |  |  |
| Q92743 | HTRA1 | | |  | | |  | | |  | | |  | |  | | |  | |  |  |
| A0A0B4J1Y9 | HV372 | | |  | | |  | | |  | | |  | |  | | |  | |  |  |
| P0DP23 | CALM1 | | |  | | |  | | |  | | |  | |  | | |  | |  |  |
| P01009 | A1AT | | |  | | |  | | |  | | |  | |  | | |  | |  |  |
| P80188 | NGAL | | |  | | |  | | |  | | |  | |  | | |  | |  |  |
| P02679 | FIBG | | |  | | |  | | |  | | |  | |  | | |  | |  |  |
| P31949 | S10AB | | |  | | |  | | |  | | |  | |  | | |  | |  |  |
| P02814 | SMR3B | | |  | | |  | | |  | | |  | |  | | |  | |  |  |
| P02788 | TRFL | | |  | | |  | | |  | | |  | |  | | |  | |  |  |
| Q02818 | NUCB1 | | |  | | |  | | |  | | |  | |  | | |  | |  |  |
| P09228 | CYTT | | |  | | |  | | |  | | |  | |  | | |  | |  |  |
| P12830 | CADH1 | | |  | | |  | | |  | | |  | |  | | |  | |  |  |
| P07195 | LDHB | | |  | | |  | | |  | | |  | |  | | |  | |  |  |
| P19013 | K2C4 | | |  | | |  | | |  | | |  | |  | | |  | |  |  |
| O00716 | E2F3 | | |  | | |  | | |  | | |  | |  | | |  | |  |  |
| Q9BUP0 | EFHD1 | | |  | | |  | | |  | | |  | |  | | |  | |  |  |
| P0DOY3 | *IGLC3 | | |  | | |  | | |  | | |  | |  | | |  | |  |  |
| P00390 | GSHR | | |  | | |  | | |  | | |  | |  | | |  | |  |  |
| P49189 | AL9A1 | | |  | | |  | | |  | | |  | |  | | |  | |  |  |
| Q16777 | H2A2C | | |  | | |  | | |  | | |  | |  | | |  | |  |  |
| P60709 | ACTB | | |  | | |  | | |  | | |  | |  | | |  | |  |  |
| P21926 | CD9 | | |  | | |  | | |  | | |  | |  | | |  | |  |  |
| A8MYP8 | ODF3B | | |  | | |  | | |  | | |  | |  | | |  | |  |  |
| P02763 | A1AG1 | | |  | | |  | | |  | | |  | |  | | |  | |  |  |
| Q99497 | PARK7 | | |  | | |  | | |  | | |  | |  | | |  | |  |  |
| P16152 | CBR1 | | |  | | |  | | |  | | |  | |  | | |  | |  |  |
| P20061 | TCO1 | | |  | | |  | | |  | | |  | |  | | |  | |  |  |
| P23526 | SAHH | | |  | | |  | | |  | | |  | |  | | |  | |  |  |
| P0DP24 | CALM2 | | |  | | |  | | |  | | |  | |  | | |  | |  |  |
| P00352 | AL1A1 | | |  | | |  | | |  | | |  | |  | | |  | |  |  |
| Q96QV6 | H2A1A | | |  | | |  | | |  | | |  | |  | | |  | |  |  |
| P04406 | G3P | | |  | | |  | | |  | | |  | |  | | |  | |  |  |
| P16403 | H12 | | |  | | |  | | |  | | |  | |  | | |  | |  |  |
| A0A075B6I4 | LVX54 | | |  | | |  | | |  | | |  | |  | | |  | |  |  |
| P19971 | TYPH | | |  | | |  | | |  | | |  | |  | | |  | |  |  |
| Q13608 | PEX6 | | |  | | |  | | |  | | |  | |  | | |  | |  |  |
| P04745 | AMY1A | | |  | | |  | | |  | | |  | |  | | |  | |  |  |
| Q99879 | H2B1M | | |  | | |  | | |  | | |  | |  | | |  | |  |  |
| P18669 | PGAM1 | | |  | | |  | | |  | | |  | |  | | |  | |  |  |
| P01624 | KV315 | | |  | | |  | | |  | | |  | |  | | |  | |  |  |
| P07108 | ACBP | | |  | | |  | | |  | | |  | |  | | |  | |  |  |
| A0A075B6S9 | KV137 | | |  | | |  | | |  | | |  | |  | | |  | |  |  |
| P08571 | CD14 | | |  | | |  | | |  | | |  | |  | | |  | |  |  |
| A0A0C4DH41 | HV461 | | |  | | |  | | |  | | |  | |  | | |  | |  |  |
| Q9NRU3 | CNNM1 | | |  | | |  | | |  | | |  | |  | | |  | |  |  |
| A0A075B6I0 | LV861 | | |  | | |  | | |  | | |  | |  | | |  | |  |  |
| P04792 | HSPB1 | | |  | | |  | | |  | | |  | |  | | |  | |  |  |
| P43490 | NAMPT | | |  | | |  | | |  | | |  | |  | | |  | |  |  |
| P0DOX2 | *IGA2 | | |  | | |  | | |  | | |  | |  | | |  | |  |  |
| Q16778 | H2B2E | | |  | | |  | | |  | | |  | |  | | |  | |  |  |
| P25774 | CATS | | |  | | |  | | |  | | |  | |  | | |  | |  |  |
| O95968 | SG1D1 | | |  | | |  | | |  | | |  | |  | | |  | |  |  |
| P58876 | H2B1D | | |  | | |  | | |  | | |  | |  | | |  | |  |  |
| P68371 | TBB4B | | |  | | |  | | |  | | |  | |  | | |  | |  |  |
| Q9GZZ8 | LACRT | | |  | | |  | | |  | | |  | |  | | |  | |  |  |
| P01824 | HV439 | | |  | | |  | | |  | | |  | |  | | |  | |  |  |
| P14555 | PA2GA | | |  | | |  | | |  | | |  | |  | | |  | |  |  |
| Q8N474 | SFRP1 | | |  | | |  | | |  | | |  | |  | | |  | |  |  |
| P07900 | HS90A | | |  | | |  | | |  | | |  | |  | | |  | |  |  |
| O75556 | SG2A1 | | |  | | |  | | |  | | |  | |  | | |  | |  |  |
| Q6ZS81 | WDFY4 | | |  | | |  | | |  | | |  | |  | | |  | |  |  |
| Q9UBQ7 | GRHPR | | |  | | |  | | |  | | |  | |  | | |  | |  |  |
| P35527 | K1C9 | | |  | | |  | | |  | | |  | |  | | |  | |  |  |
| P63241 | IF5A1 | | |  | | |  | | |  | | |  | |  | | |  | |  |  |
| O60664 | PLIN3 | | |  | | |  | | |  | | |  | |  | | |  | |  |  |
| P05109 | S10A8 | | |  | | |  | | |  | | |  | |  | | |  | |  |  |
| Q15293 | RCN1 | | |  | | |  | | |  | | |  | |  | | |  | |  |  |
| P01743 | HV146 | | |  | | |  | | |  | | |  | |  | | |  | |  |  |
| P57723 | PCBP4 | | |  | | |  | | |  | | |  | |  | | |  | |  |  |
| A0A0A0MT36 | KVD21 | | |  | | |  | | |  | | |  | |  | | |  | |  |  |
| P22392 | NDKB | | |  | | |  | | |  | | |  | |  | | |  | |  |  |
|  |  | | |  | | |  | | |  | | |  | |  | | |  | |  |  |
| * = Immunoglobulins | | | |  | | |  | | |  | | |  | |  | | |  | |  |  |

**Table S2.** Quantitative Changes in Selected Reflex Tear Proteins Between Sampling Methods

| **Method Comparison** | **10-Plex ^1^** | **Uniprot\|Protein ID** | **Reflex Tear Change [14]** | **Fold Change (Log2)** | **Adj. p-value ^2^** |
| --- | --- | --- | --- | --- | --- |
| SS/MCT | 1 | P02768\|ALBU_HUMAN | Higher | 3.86 | NS |
|  | 1 | Q16378\|PROL4_HUMAN | Higher | 0.90 | NS |
|  | 1 | Q96DA0\|ZG16B_HUMAN | Higher | 0.46 | NS |
|  | 1 | P10909\|CLUS_HUMAN | Lower | 0.65 | NS |
|  | 1 | P01037\|CYTN_HUMAN | Lower | 0.22 | NS |
|  | 1 | P01876\|IGHA1_HUMAN | Lower | 1.31 | 0.028 |
|  | 1 | P01833\|PIGR_HUMAN | Lower | 1.16 | NS |
|  | 1 | O75556\|SG2A1_HUMAN | Lower | 0.73 | NS |
|  | 1 | P31025\|LCN1_HUMAN | No change | 2.17 | NS |
|  | 1 | P61626\|LYSC_HUMAN | No change | 1.71 | NS |
|  | 1 | P02788\|TRFL_HUMAN | No change | 1.89 | NS |
|  | 2 | P02768\|ALBU_HUMAN | Higher | 2.90 | NS |
|  | 2 | Q16378\|PROL4_HUMAN | Higher | 0.14 | NS |
|  | 2 | Q96DA0\|ZG16B_HUMAN | Higher | 0.59 | NS |
|  | 2 | P10909\|CLUS_HUMAN | Lower | 0.21 | NS |
|  | 2 | P01037\|CYTN_HUMAN | Lower | 0.46 | NS |
|  | 2 | P01876\|IGHA1_HUMAN | Lower | 0.15 | NS |
|  | 2 | P01833\|PIGR_HUMAN | Lower | -0.18 | NS |
|  | 2 | O75556\|SG2A1_HUMAN | Lower | -0.03 | NS |
|  | 2 | P31025\|LCN1_HUMAN | No change | 0.76 | NS |
|  | 2 | P61626\|LYSC_HUMAN | No change | 0.36 | NS |
|  | 2 | P02788\|TRFL_HUMAN | No change | 0.14 | NS |
|  |  |  |  |  |  |
| **Method Comparison** | **10-Plex ^1^** | **Uniprot\|Protein ID** | **Reflex Tear Change [14]** | **Fold Change (Log2)** | **Adj. p-value ^2^** |
| SS/SCL | 1 | P02768\|ALBU_HUMAN | Higher | 5.20 | NS |
|  | 1 | Q16378\|PROL4_HUMAN | Higher | 1.71 | 0.049 |
|  | 1 | Q96DA0\|ZG16B_HUMAN | Higher | 3.45 | NS |
|  | 1 | P10909\|CLUS_HUMAN | Lower | 3.19 | NS |
|  | 1 | P01037\|CYTN_HUMAN | Lower | 2.99 | NS |
|  | 1 | P01876\|IGHA1_HUMAN | Lower | 4.20 | NS |
|  | 1 | P01833\|PIGR_HUMAN | Lower | 3.64 | NS |
|  | 1 | O75556\|SG2A1_HUMAN | Lower | 4.20 | NS |
|  | 1 | P31025\|LCN1_HUMAN | No change | 4.70 | NS |
|  | 1 | P61626\|LYSC_HUMAN | No change | -0.96 | NS |
|  | 1 | P02788\|TRFL_HUMAN | No change | 4.10 | 0.047 |
|  | 2 | P02768\|ALBU_HUMAN | Higher | 4.65 | NS |
|  | 2 | Q16378\|PROL4_HUMAN | Higher | 1.52 | NS |
|  | 2 | Q96DA0\|ZG16B_HUMAN | Higher | 3.65 | NS |
|  | 2 | P10909\|CLUS_HUMAN | Lower | 1.96 | NS |
|  | 2 | P01037\|CYTN_HUMAN | Lower | 2.52 | NS |
|  | 2 | P01876\|IGHA1_HUMAN | Lower | 2.67 | NS |
|  | 2 | P01833\|PIGR_HUMAN | Lower | 2.52 | NS |
|  | 2 | O75556\|SG2A1_HUMAN | Lower | 2.94 | NS |
|  | 2 | P31025\|LCN1_HUMAN | No change | 3.63 | NS |
|  | 2 | P61626\|LYSC_HUMAN | No change | -2.59 | NS |
|  | 2 | P02788\|TRFL_HUMAN | No change | 2.78 | NS |
|  |  |  |  |  |  |
| **Method Comparison** | **10-Plex ^1^** | **Uniprot\|Protein ID** | **Reflex Tear Change [14]** | **Fold Change (Log2)** | **Adj. p-value ^2^** |
| MCT/SCL | 1 | P02768\|ALBU_HUMAN | Higher | 1.33 | NS |
|  | 1 | Q16378\|PROL4_HUMAN | Higher | 0.81 | NS |
|  | 1 | Q96DA0\|ZG16B_HUMAN | Higher | 2.98 | 0.034 |
|  | 1 | P10909\|CLUS_HUMAN | Lower | 2.54 | NS |
|  | 1 | P01037\|CYTN_HUMAN | Lower | 2.77 | NS |
|  | 1 | P01876\|IGHA1_HUMAN | Lower | 2.89 | NS |
|  | 1 | P01833\|PIGR_HUMAN | Lower | 2.47 | NS |
|  | 1 | O75556\|SG2A1_HUMAN | Lower | 3.47 | 0.044 |
|  | 1 | P31025\|LCN1_HUMAN | No change | 2.53 | NS |
|  | 1 | P61626\|LYSC_HUMAN | No change | -2.67 | 0.016 |
|  | 1 | P02788\|TRFL_HUMAN | No change | 2.21 | 0.045 |
|  | 2 | P02768\|ALBU_HUMAN | Higher | 1.74 | NS |
|  | 2 | Q16378\|PROL4_HUMAN | Higher | 1.38 | NS |
|  | 2 | Q96DA0\|ZG16B_HUMAN | Higher | 3.06 | 0.024 |
|  | 2 | P10909\|CLUS_HUMAN | Lower | 1.75 | 0.030 |
|  | 2 | P01037\|CYTN_HUMAN | Lower | 2.06 | NS |
|  | 2 | P01876\|IGHA1_HUMAN | Lower | 2.52 | NS |
|  | 2 | P01833\|PIGR_HUMAN | Lower | 2.70 | NS |
|  | 2 | O75556\|SG2A1_HUMAN | Lower | 2.97 | NS |
|  | 2 | P31025\|LCN1_HUMAN | No change | 2.88 | NS |
|  | 2 | P61626\|LYSC_HUMAN | No change | -2.95 | 0.012 |
|  | 2 | P02788\|TRFL_HUMAN | No change | 2.63 | 0.035 |
| ^1^ 1 = TF5, 2 = TF6 |  |  |  |  |  |
| ^2^ Statistical significance was tested using the Benjamini-Hochberg equation. | | | |  |  |
| NS = Not significant | |  |  |  |  |

| **Table S3.** Schirmer Strip Wet Length by Subject | | | | |
| --- | --- | --- | --- | --- |
|  |  |  |  |  |
|  | Subject | OD (mm) | OS (mm) |  |
|  | 1 | >35 | >35 |  |
|  | 2 | >35 | >35 |  |
|  | 3 | >35 | >35 |  |
|  | 4 | >35 | 31 |  |
|  | 5 | >35 | >35 |  |
|  | 6 | 8 | 13 |  |
|  |  |  |  |  |
|  | Wet lengths >35mm represent saturated Schirmer strips | | |  |
|  |  |  |  |  |
|  |  |  |  |  |

| **Table S4.** Supplemental Mass Spectrometry Data | | | |
| --- | --- | --- | --- |
| **Data** | **TMT 10-plex 1 (TF5)** | **TMT 10-plex 2 (TF6)** | **TF5 & TF6** |
| MS1 scans (Spider) | 9288 | 9153 | 18441 |
| MS2/MS3 scans (Spider) | 123015 | 121496 | 244511 |
| Peptide IDs (Spider) | 3129 | 3628 | 4733 |
| Peptide IDs (Quant) | 3043 | 3551 | 4616 |
| Protein IDs (Spider) | 432 | 445 | 525 |
| Protein IDs (Quant) | 341 | 350 | 432 |
| Peptide Spectrum Matches at 1% FDR (Spider) | 8053 | 8448 | 16501 |
| Peptide Spectrum Matches at 1% FDR (Quant) | - | - | 15912 |

| **Table S5.** Mass Spectrometry Settings & Details | |
| --- | --- |
|  |  |
| Database: Uniprot_SwissProt_Oct2020, Homo sapiens (human) | |
| Searched entries: 20384 | |
| Parent Mass Error Tolerance: 20ppm | |
| Fragment Mass Effort Tolerance: 0.5 Da | |
| Fixed Modifications: Carbamidomethylation, TMT 10plex | |
| Variable Modifications: Pyro-glu from Q, Oxidation (M) | |
| The target/decoy method used for PSM FDR determination |  |
| Only unique proteins were used in protein summation | |
| Reporter ion peak heights were directly correlated to TMT labeled peptides | |
| Reporter ion quantitative intensity values were used as protein abundance proxies | |

**Table S6.** TMT10-plex Label Assignments


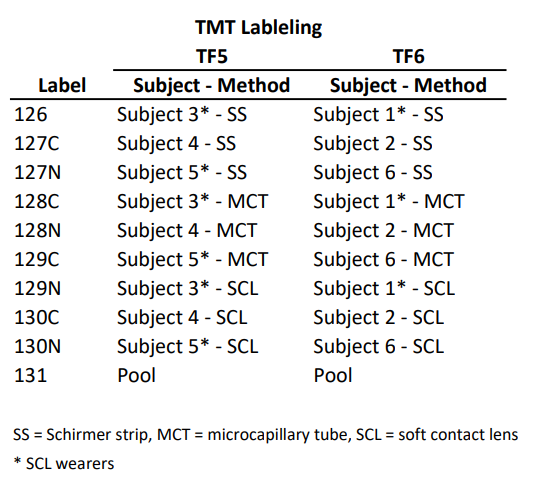


| **Table S7.** Contaminants Identified In Subject Tears Using the common Repository of Adventitious Proteins (cRAP) database | | |
| --- | --- | --- |
|  |  |  |
| **Uniprot Accession** | **Protein ID** | **Protein Name** |
| P61626 | LYSC_HUMAN | Lysozyme C |
| P06396 | GELS_HUMAN | Gelsolin |
| P35527 | K1C9_HUMAN | Keratin, type I cytoskeletal 9 |
| P35908 | K22E_HUMAN | Keratin, type II cytoskeletal 2 epidermal |
| P04264 | K2C1_HUMAN | Keratin, type II cytoskeletal 1 |
| P62937 | PPIA_HUMAN | Peptidyl-prolyl cis-trans isomerase A |
| Q06830 | PRDX1_HUMAN | Peroxiredoxin-1 |
| P61769 | B2MG_HUMAN | Beta-2-microglobulin |
| P09211 | GSTP1_HUMAN | Glutathione S-transferase P |
| P13645 | K1C10_HUMAN | Keratin, type I cytoskeletal 10 |
| P02788 | TRFL_HUMAN | Lactotransferrin |
| P02768 | ALBU_HUMAN | Albumin |
| P62979 | RS27A_HUMAN | Ubiquitin-ribosomal protein eS31 fusion protein |
| P68871 | HBB_HUMAN | Hemoglobin subunit beta |
| P08758 | ANXA5_HUMAN | Annexin A5 |
| P02787 | TRFE_HUMAN | Serotransferrin |
| P07339 | CATD_HUMAN | Cathepsin D |
| P01008 | ANT3_HUMAN | Antithrombin-III |
| The cRAP database was accessed 8/21/23 | | |

| **Table S8.** Median Protein Values for Individual Proteins for Individual 10-plexes | | | | | | | | | | | | |
| --- | --- | --- | --- | --- | --- | --- | --- | --- | --- | --- | --- | --- |
|  |  |  |  |  |  |  |  |  |  |  |  |  |
| **TMT10-plex 1 (TF5)** | |  |  |  |  |  |  |  |  |  |  |  |
| **Accession** | **3S** | **4S** | **5S** | **Median** | **3M** | **4M** | **5M** | **Median** | **3C** | **4C** | **5C** | **Median** |
| P2788\|TRFL_HUMAN | 7.38195 | 8.20476 | 8.70205 | 8.20476 | 6.49222 | 5.76784 | 6.35463 | 6.35463 | 3.77546 | 3.60326 | 4.61609 | 3.77546 |
| P2768\|ALBU_HUMAN | 3.1078 | 8.59191 | 5.0708 | 5.0708 | 2.74761 | 1.83179 | 0.59902 | 1.83179 | 0.03709 | 0.29799 | 0.84062 | 0.29799 |
| P3125\|LCN1_HUMAN | 5.87985 | 7.54185 | 7.3809 | 7.3809 | 4.87167 | 5.03606 | 4.38067 | 4.87167 | 2.20413 | 1.88042 | 2.61919 | 2.20413 |
| P9816\|PGBM_HUMAN | 0.93152 | 0.92858 | 1.72892 | 0.93152 | 0.18235 | 0.10382 | 0.15 | 0.15 | -1.31067 | -0.97134 | -1.40654 | -1.31067 |
| P1833\|PIGR_HUMAN | 3.10972 | 4.78021 | 2.91628 | 3.10972 | 2.41763 | 2.99404 | 1.90871 | 2.41763 | 0.42131 | -0.38516 | -0.14039 | -0.14039 |
| P124\|CO3_HUMAN | 0.04355 | 1.18143 | -0.04427 | 0.04355 | -0.82178 | -1.76536 | -1.40477 | -1.40477 | -3.39014 | -3.77244 | -2.76378 | -3.39014 |
| P1876\|IGHA1_HUMAN | 3.16947 | 5.02388 | 2.39032 | 3.16947 | 2.10552 | 3.25087 | 1.29736 | 2.10552 | -0.10142 | -1.47138 | -0.43979 | -0.43979 |
| P61626\|LYSC_HUMAN | 5.88584 | 6.71991 | 6.76244 | 6.71991 | 4.98303 | 4.66719 | 4.58292 | 4.66719 | 7.58801 | 7.08743 | 7.56093 | 7.56093 |
| P25311\|ZA2G_HUMAN | 3.06755 | 3.68635 | 3.41395 | 3.41395 | 2.49991 | 2.42764 | 2.02303 | 2.42764 | -0.54407 | -0.66529 | -0.4778 | -0.54407 |
| PDOX7\|IGK_HUMAN | 3.19272 | 5.30374 | 2.78901 | 3.19272 | 2.51797 | 3.22013 | 1.82869 | 2.51797 | 0.30474 | -0.10728 | -0.06719 | -0.06719 |
| P4264\|K2C1_HUMAN | 0.28763 | -0.45826 | -0.44582 | -0.44582 | -0.48631 | -1.00829 | -0.22559 | -0.48631 | -0.71876 | -0.91459 | -1.22809 | -0.91459 |
| Q5VSP4\|LC1L1_HUMAN | -6.00302 | -7.92303 | -6.29528 | -6.29528 | -6.63557 | -6.22994 | -6.64855 | -6.63557 | 0 | 0 | 0 | 0 |
| P2787\|TRFE_HUMAN | 1.61901 | 3.72083 | 1.74898 | 1.74898 | 0.58774 | -0.0805 | 0.11176 | 0.11176 | -1.94884 | -1.68013 | -2.0144 | -1.94884 |
| P14618\|KPYM_HUMAN | -0.61459 | 1.56003 | 0.25004 | 0.25004 | -2.28724 | -2.60753 | -3.90691 | -2.60753 | -4.00578 | -3.79375 | -4.01526 | -4.00578 |
| PDOX2\|IGA2_HUMAN | -8.56721 | -10.456 | -8.19193 | -8.56721 | -7.99537 | -7.30613 | -6.90438 | -7.30613 | 0 | 0 | 0 | 0 |
| P6733\|ENOA_HUMAN | -0.25562 | 2.10025 | 0.78293 | 0.78293 | -1.70787 | -2.15866 | -3.11629 | -2.15866 | -4.54015 | -3.54037 | -4.04011 | -4.04011 |
| P1913\|K2C4_HUMAN | 0.31447 | 1.49684 | 1.38957 | 1.38957 | -1.85428 | -1.07993 | -1.83337 | -1.83337 | -5.64848 | -4.66314 | -2.7791 | -4.66314 |
| P136\|CYTS_HUMAN | 2.47042 | 3.39465 | 0.60827 | 2.47042 | 2.39048 | 2.45739 | 0.21821 | 2.39048 | -1.22907 | -2.50351 | -1.16679 | -1.22907 |
| P2198\|TGM2_HUMAN | -0.00963 | 2.887 | 0.69508 | 0.69508 | -1.16014 | -1.23814 | -2.72495 | -1.23814 | -3.30282 | -3.18315 | -3.42587 | -3.30282 |
| Q9UGM3\|DMBT1_HUMAN | 0.18847 | -1.0158 | -0.60254 | -0.60254 | 0.00294 | -0.96887 | -0.80313 | -0.80313 | -3.65922 | -4.64734 | -3.07584 | -3.65922 |
| P137\|CYTN_HUMAN | 0.27793 | 0.6012 | -0.97617 | 0.27793 | 0.29776 | 0.26061 | -1.3151 | 0.26061 | -3.34696 | -3.2752 | -2.44599 | -3.2752 |
| P6396\|GELS_HUMAN | -1.21827 | 0.32756 | -0.5831 | -0.5831 | -2.13597 | -2.59519 | -2.93362 | -2.59519 | -6.0388 | -5.33806 | -5.40911 | -5.40911 |
| P13647\|K2C5_HUMAN | -0.03901 | 0.92037 | 1.33592 | 0.92037 | -1.70714 | -0.77884 | -1.3662 | -1.3662 | -2.83211 | -2.35956 | -1.78827 | -2.35956 |
| P199\|CLUS_HUMAN | 1.70861 | 2.86101 | 1.11826 | 1.70861 | 1.65775 | 1.50146 | 0.56896 | 1.50146 | -1.21703 | -1.51648 | -1.15143 | -1.21703 |
| P13645\|K1C1_HUMAN | -1.47582 | -3.17907 | -1.81749 | -1.81749 | -2.2178 | -2.28632 | -1.57999 | -2.2178 | -3.23834 | -3.08143 | -3.57676 | -3.23834 |
| P9888\|MUC5A_HUMAN | -1.2581 | -1.48144 | 0.27193 | -1.2581 | -3.43429 | -2.56093 | -3.97051 | -3.43429 | -4.56292 | -5.23208 | -4.99942 | -4.99942 |
| Q13421\|MSLN_HUMAN | -0.09731 | 0.05665 | -1.4865 | -0.09731 | -0.58475 | -0.4196 | -2.25542 | -0.58475 | -3.27562 | -3.66073 | -3.71952 | -3.66073 |
| P19\|A1AT_HUMAN | -1.4568 | 2.50378 | -0.9042 | -0.9042 | -1.37843 | -2.1471 | -2.80648 | -2.1471 | -4.55525 | -4.25357 | -4.26447 | -4.26447 |
| P679\|ACTB_HUMAN | -0.9672 | 1.86139 | 0.0675 | 0.0675 | -2.0777 | -2.56479 | -4.33219 | -2.56479 | -5.57081 | -5.5138 | -4.8842 | -5.5138 |
| P483\|ANXA1_HUMAN | 0.02001 | 2.29441 | 1.1382 | 1.1382 | -1.17919 | -1.4707 | -2.34632 | -1.4707 | -2.81041 | -2.77573 | -3.07525 | -2.81041 |
| P2647\|APOA1_HUMAN | -2.61862 | 1.01351 | -1.39212 | -1.39212 | -2.31823 | -3.06846 | -3.54773 | -3.06846 | -4.17571 | -3.5609 | -4.23688 | -4.17571 |
| P2538\|K2C6A_HUMAN | -5.82049 | -7.98071 | -5.40259 | -5.82049 | -6.12774 | -4.22489 | -4.72725 | -4.72725 | -7.67592 | -5.10304 | -6.71071 | -6.71071 |
| P35527\|K1C9_HUMAN | -6.08418 | -5.64223 | -5.31213 | -5.64223 | -5.14294 | -5.44061 | -4.8989 | -5.14294 | -5.839 | -6.33457 | -6.18589 | -6.18589 |
| P8727\|K1C19_HUMAN | 0.21376 | 1.65599 | 1.57027 | 1.57027 | -1.30827 | -1.30371 | -1.72441 | -1.30827 | -5.14437 | -5.87136 | -3.04039 | -5.14437 |
| P738\|HPT_HUMAN | 0.12915 | 2.71998 | -0.44907 | 0.12915 | -0.34947 | -0.35536 | -2.07673 | -0.35536 | -3.55957 | -3.96354 | -3.77501 | -3.77501 |
| P7355\|ANXA2_HUMAN | -1.28261 | 0.9601 | -0.0218 | -0.0218 | -2.76845 | -2.63197 | -3.75471 | -2.76845 | -5.53096 | -5.56485 | -4.70332 | -5.53096 |
| P352\|AL1A1_HUMAN | -0.26418 | 2.22332 | 0.39614 | 0.39614 | -1.42162 | -1.88985 | -3.0119 | -1.88985 | -4.38809 | -4.43856 | -3.95357 | -4.38809 |
| Q16378\|PROL4_HUMAN | 3.69321 | 4.02811 | 3.21402 | 3.69321 | 3.31806 | 2.99273 | 1.91162 | 2.99273 | 2.067 | 2.48625 | 1.25263 | 2.067 |
| P3598\|K22E_HUMAN | -1.46789 | -3.50672 | -2.53801 | -2.53801 | -2.34885 | -3.19363 | -1.70057 | -2.34885 | -2.56172 | -2.62898 | -2.86546 | -2.62898 |
| PDOX5\|IGG1_HUMAN | -2.30661 | 1.37109 | -1.19812 | -1.19812 | -2.33005 | -2.91223 | -3.82733 | -2.91223 | -5.16933 | -4.02119 | -4.75819 | -4.75819 |
| P1871\|IGHM_HUMAN | -1.56926 | 1.04178 | -1.22565 | -1.22565 | -1.70199 | -0.22012 | -1.89321 | -1.70199 | -3.62597 | -3.77915 | -3.6058 | -3.62597 |
| P45\|CERU_HUMAN | -0.46417 | 1.00233 | -0.40746 | -0.40746 | -1.30802 | -1.31703 | -2.33356 | -1.31703 | -4.67871 | -4.85717 | -4.78105 | -4.78105 |
| Q9GZZ8\|LACRT_HUMAN | 5.58158 | 7.07587 | 3.97628 | 5.58158 | 5.25299 | 5.19016 | 3.0827 | 5.19016 | 2.33283 | 2.26094 | 1.7155 | 2.26094 |
| PDMV8\|HS71A_HUMAN | -1.65825 | -0.09226 | -0.71005 | -0.71005 | -3.39022 | -3.27177 | -4.93086 | -3.39022 | -5.85234 | -6.20991 | -5.22169 | -5.85234 |
| PDMV9\|HS71B_HUMAN | -1.65825 | -0.09226 | -0.71005 | -0.71005 | -3.39022 | -3.27177 | -4.93086 | -3.39022 | -5.85234 | -6.20991 | -5.22169 | -5.85234 |
| P13646\|K1C13_HUMAN | -2.95823 | -2.71775 | -1.77874 | -2.71775 | -4.27803 | -3.32634 | -4.03721 | -4.03721 | -6.21269 | -5.45853 | -5.16235 | -5.45853 |
| P9228\|CYTT_HUMAN | -5.75588 | -7.12231 | -7.76803 | -7.12231 | -5.95017 | -6.21796 | -7.00598 | -6.21796 | 0 | 0 | -8.22589 | 0 |
| P672\|S1A9_HUMAN | 3.46174 | 6.9284 | 4.19673 | 4.19673 | 1.51763 | 1.09944 | -0.45519 | 1.09944 | -1.35381 | -0.31757 | -0.26838 | -0.31757 |
| P374\|ILEU_HUMAN | -2.08833 | 0.53568 | -0.33805 | -0.33805 | -2.36047 | -2.56678 | -3.25035 | -2.56678 | -5.99696 | -5.68419 | -6.16699 | -5.99696 |
| P6174\|TPIS_HUMAN | -1.29078 | 0.91994 | -0.25936 | -0.25936 | -2.75857 | -3.16616 | -4.69032 | -3.16616 | -7.62605 | -5.43259 | -5.53877 | -5.53877 |
| P8729\|K2C7_HUMAN | -2.18121 | -0.428 | -1.172 | -1.172 | -2.63141 | -2.40089 | -3.46594 | -2.63141 | -4.77389 | -4.65185 | -4.98035 | -4.77389 |
| P12273\|PIP_HUMAN | 4.38797 | 5.0462 | 4.41839 | 4.41839 | 2.95738 | 2.75371 | 1.47835 | 2.75371 | -0.42696 | -0.62759 | -0.59794 | -0.59794 |
| P4259\|K2C6B_HUMAN | -5.52145 | -8.00775 | -5.48379 | -5.52145 | -6.03439 | -4.52733 | -5.10676 | -5.10676 | -7.45965 | -5.40794 | -7.52562 | -7.45965 |
| P1861\|IGHG4_HUMAN | -6.85526 | -5.26422 | -9.58008 | -6.85526 | -6.89936 | -8.43025 | -8.43212 | -8.43025 | 0 | 0 | 0 | 0 |
| P1859\|IGHG2_HUMAN | -6.77616 | -3.67788 | -6.33857 | -6.33857 | -6.5862 | -6.74283 | -8.83676 | -6.74283 | -10.9347 | -9.74573 | 0 | -9.74573 |
| P6814\|EF1A1_HUMAN | -0.8001 | 1.36827 | 0.0292 | 0.0292 | -2.27888 | -2.68249 | -3.8699 | -2.68249 | -5.74907 | -4.9865 | -4.87049 | -4.9865 |
| P833\|NUCB2_HUMAN | 0.25521 | -0.5867 | 0.41391 | 0.25521 | 0.43748 | -0.45283 | 0.04104 | 0.04104 | -3.5811 | -3.32609 | -3.10479 | -3.32609 |
| P446\|G3P_HUMAN | -1.06298 | 0.9163 | -0.07633 | -0.07633 | -2.10895 | -2.22896 | -3.18569 | -2.22896 | -6.08296 | -5.32851 | -4.77374 | -5.32851 |
| P558\|PGK1_HUMAN | -1.77716 | 0.0629 | -1.07966 | -1.07966 | -3.05771 | -3.34545 | -4.11173 | -3.34545 | -5.91311 | -5.6365 | -5.56423 | -5.6365 |
| B9A64\|IGLL5_HUMAN | -2.88079 | -1.90068 | -3.44913 | -2.88079 | -2.50481 | -1.38129 | -2.45425 | -2.45425 | -5.12783 | -5.35347 | -6.61254 | -5.35347 |
| O75556\|SG2A1_HUMAN | 1.8537 | 2.9399 | 1.21649 | 1.8537 | 1.58751 | 1.93355 | 0.3017 | 1.58751 | -1.65088 | -2.39276 | -2.54158 | -2.39276 |
| P4792\|HSPB1_HUMAN | -1.11764 | 0.61572 | -0.51235 | -0.51235 | -2.50018 | -2.70934 | -3.44538 | -2.70934 | -6.65268 | -5.62167 | -5.85907 | -5.85907 |
| P123\|A2MG_HUMAN | -2.51015 | 0.98592 | -1.16619 | -1.16619 | -2.39637 | -2.83322 | -3.81927 | -2.83322 | -5.88861 | -5.22874 | -5.58501 | -5.58501 |
| P2533\|K1C14_HUMAN | -4.25705 | -5.51405 | -3.11133 | -4.25705 | -3.06365 | -3.93041 | -2.53436 | -3.06365 | -8.6203 | -5.86596 | -7.94605 | -7.94605 |
| P9211\|GSTP1_HUMAN | -1.81678 | 0.20127 | -1.34763 | -1.34763 | -2.83289 | -3.04126 | -3.86327 | -3.04126 | -6.5921 | -5.77691 | -6.80093 | -6.5921 |
| P344\|PRDX5_HUMAN | -1.53969 | 0.47467 | -1.06099 | -1.06099 | -2.87831 | -3.52816 | -4.47337 | -3.52816 | -3.77098 | -3.60764 | -4.75983 | -3.77098 |
| P386\|PEBP1_HUMAN | -2.66762 | -1.22803 | -2.32935 | -2.32935 | -4.22505 | -4.47405 | -6.11792 | -4.47405 | -8.45685 | -7.14303 | -5.42585 | -7.14303 |
| P186\|IGHG3_HUMAN | -8.42 | -6.21079 | -9.36558 | -8.42 | 0 | -9.04332 | 0 | 0 | -11.0548 | 0 | 0 | 0 |
| P475\|ALDOA_HUMAN | -2.44419 | -0.76593 | -1.95078 | -1.95078 | -3.60727 | -4.04142 | -5.21552 | -4.04142 | -8.08343 | -6.49547 | -6.86444 | -6.86444 |
| P5787\|K2C8_HUMAN | -1.44683 | -0.27473 | -0.07806 | -0.27473 | -2.26059 | -2.35732 | -2.7834 | -2.35732 | -5.5309 | -5.72406 | -5.23705 | -5.5309 |
| P2675\|FIBB_HUMAN | -3.23431 | 0.5158 | -1.99442 | -1.99442 | -2.7891 | -3.21548 | -5.18928 | -3.21548 | -6.59681 | -5.3995 | -6.27652 | -6.27652 |
| P279\|HEMO_HUMAN | -4.16613 | -1.54389 | -3.36086 | -3.36086 | -4.11674 | -5.16776 | -4.91682 | -4.91682 | -8.2431 | -6.78491 | -6.80206 | -6.80206 |
| P2679\|FIBG_HUMAN | -4.07533 | -0.57032 | -2.85047 | -2.85047 | -3.81872 | -4.44933 | -6.04727 | -4.44933 | -4.54424 | -3.9503 | -4.76872 | -4.54424 |
| Q838\|LG3BP_HUMAN | 0.34105 | -0.57302 | 1.2996 | 0.34105 | -0.47934 | -0.50058 | -0.80461 | -0.50058 | -3.64076 | -3.26238 | -3.0479 | -3.26238 |
| P11142\|HSP7C_HUMAN | -2.70505 | -1.61129 | -2.17415 | -2.17415 | -3.32167 | -3.99705 | -3.9123 | -3.9123 | -5.73319 | -5.38549 | -5.59082 | -5.59082 |
| P2774\|VTDB_HUMAN | -2.78462 | -0.14452 | -1.94831 | -1.94831 | -2.6067 | -3.25917 | -3.47997 | -3.25917 | -6.39096 | -5.84288 | -6.14219 | -6.14219 |
| O95968\|SG1D1_HUMAN | 0.71995 | 1.52663 | 0.10992 | 0.71995 | 0.45284 | 1.00789 | -0.4023 | 0.45284 | -2.32903 | -2.18531 | -2.35038 | -2.32903 |
| P79\|HS9A_HUMAN | -2.3623 | -0.17108 | -1.58889 | -1.58889 | -3.09314 | -3.43627 | -4.04934 | -3.43627 | -6.47035 | -5.63394 | -5.15438 | -5.63394 |
| P7858\|CATB_HUMAN | -0.44837 | -0.90184 | 0.11022 | -0.44837 | -1.49404 | -1.87743 | -1.89759 | -1.87743 | -2.83191 | -2.94357 | -2.62935 | -2.83191 |
| Q683\|PRDX1_HUMAN | -1.73929 | 0.30443 | -0.682 | -0.682 | -3.03575 | -3.40193 | -4.46286 | -3.40193 | -5.78031 | -5.52817 | -5.23715 | -5.52817 |
| Q8N474\|SFRP1_HUMAN | 1.11197 | 0.09703 | 2.21631 | 1.11197 | -2.14619 | -0.66338 | -2.41725 | -2.14619 | -3.37071 | -3.12879 | -1.60643 | -3.12879 |
| P762\|SAP_HUMAN | -1.3494 | -0.35436 | -1.21581 | -1.21581 | -1.06667 | -1.77567 | -1.5242 | -1.5242 | -4.68537 | -4.33728 | -3.86459 | -4.33728 |
| P519\|S1A8_HUMAN | 0.36357 | 3.21281 | 1.0675 | 1.0675 | -0.73509 | -1.67982 | -3.56368 | -1.67982 | -3.24014 | -2.36613 | -2.38927 | -2.38927 |
| Q13228\|SBP1_HUMAN | -2.73356 | -0.42316 | -1.89151 | -1.89151 | -4.29242 | -4.42204 | -5.52787 | -4.42204 | -8.08999 | -7.59348 | -6.6543 | -7.59348 |
| Q7Z46\|MYH14_HUMAN | -3.21318 | -1.83065 | -2.5457 | -2.5457 | -3.90521 | -4.37287 | -4.80699 | -4.37287 | -8.40752 | -7.99991 | -7.58924 | -7.99991 |
| P31946\|1433B_HUMAN | -4.02356 | -1.92296 | -2.95498 | -2.95498 | -4.2488 | -4.72063 | -5.76392 | -4.72063 | -8.60049 | -8.54718 | -7.83974 | -8.54718 |
| P15311\|EZRI_HUMAN | -3.07777 | -1.97054 | -2.45552 | -2.45552 | -3.78689 | -4.25933 | -4.24523 | -4.24523 | -6.27126 | -6.23078 | -5.74043 | -6.23078 |
| P8188\|NGAL_HUMAN | -1.47691 | -0.89749 | -1.51115 | -1.47691 | -1.87203 | -2.40832 | -2.15601 | -2.15601 | -4.89607 | -4.23834 | -3.58134 | -4.23834 |
| P134\|CYTC_HUMAN | -2.38191 | -2.72298 | -2.2791 | -2.38191 | -2.54135 | -2.95936 | -2.68368 | -2.68368 | -5.25492 | -4.89438 | -4.83886 | -4.89438 |
| P6314\|1433Z_HUMAN | -3.11018 | -1.45682 | -2.44344 | -2.44344 | -3.91731 | -3.99805 | -4.35659 | -3.99805 | -8.76895 | -7.70815 | -7.83725 | -7.83725 |
| Q9BQE3\|TBA1C_HUMAN | -2.81427 | -0.97559 | -2.40421 | -2.40421 | -3.60069 | -4.18743 | -4.86005 | -4.18743 | -6.58923 | -6.14347 | -6.17707 | -6.17707 |
| Q71U36\|TBA1A_HUMAN | -2.81427 | -0.97559 | -2.40421 | -2.40421 | -3.60069 | -4.18743 | -4.86005 | -4.18743 | -6.58923 | -6.14347 | -6.17707 | -6.17707 |
| P68363\|TBA1B_HUMAN | -2.81427 | -0.97559 | -2.40421 | -2.40421 | -3.60069 | -4.18743 | -4.86005 | -4.18743 | -6.58923 | -6.14347 | -6.17707 | -6.17707 |
| P1643\|H12_HUMAN | -2.74754 | -0.1788 | -1.75256 | -1.75256 | -3.31628 | -3.3946 | -4.42794 | -3.3946 | -6.4621 | -6.45344 | -5.92934 | -6.45344 |
| Q14515\|SPRL1_HUMAN | -5.37264 | -6.76658 | -6.29423 | -6.29423 | -5.27119 | -5.54191 | -6.30496 | -5.54191 | -8.27158 | -7.03327 | -7.78853 | -7.78853 |
| P966\|ASSY_HUMAN | -4.45701 | -2.92786 | -4.20907 | -4.20907 | -5.62871 | -6.04219 | -7.76742 | -6.04219 | -9.24696 | -9.20362 | -9.63242 | -9.24696 |
| Q99935\|PROL1_HUMAN | -0.89698 | -1.49947 | -0.62387 | -0.89698 | -1.32509 | -2.11931 | -1.93114 | -1.93114 | -4.13648 | -3.9987 | -3.06807 | -3.9987 |
| P8238\|HS9B_HUMAN | -8.06532 | -7.27523 | -8.57558 | -8.06532 | 0 | 0 | 0 | 0 | 0 | -9.36877 | 0 | 0 |
| P3782\|TAGL2_HUMAN | -3.22239 | -1.86971 | -2.94666 | -2.94666 | -4.51341 | -4.74189 | -5.24847 | -4.74189 | -6.63958 | -5.89961 | -6.00117 | -6.00117 |
| Q96DA\|ZG16B_HUMAN | -1.49086 | -2.4093 | -1.26702 | -1.49086 | -2.19999 | -1.94866 | -2.41314 | -2.19999 | -5.51025 | -5.19295 | -4.80711 | -5.19295 |
| P341\|PRDX6_HUMAN | -5.44872 | -3.97946 | -4.77793 | -4.77793 | -5.76687 | -5.75587 | -6.55182 | -5.76687 | -7.48162 | -6.77072 | -8.33302 | -7.48162 |
| P1121\|BIP_HUMAN | -4.05408 | -3.27686 | -3.34595 | -3.34595 | -4.21108 | -3.89797 | -4.42798 | -4.21108 | -8.02923 | -7.05577 | -7.74448 | -7.74448 |
| P23528\|COF1_HUMAN | -2.59736 | -0.72083 | -1.98036 | -1.98036 | -4.04365 | -4.39635 | -5.48583 | -4.39635 | -11.0921 | -9.89448 | -7.40577 | -9.89448 |
| P2279\|PERL_HUMAN | -1.30892 | -1.99985 | -1.67903 | -1.67903 | -1.7401 | -1.85885 | -2.36793 | -1.85885 | -3.28698 | -3.42057 | -3.45817 | -3.42057 |
| P111\|AACT_HUMAN | -5.33933 | -3.30491 | -5.32982 | -5.32982 | -4.81867 | -5.39823 | -5.84742 | -5.39823 | -9.32801 | -7.69145 | -9.23959 | -9.23959 |
| P6727\|APOA4_HUMAN | -2.39731 | 1.28576 | -0.96425 | -0.96425 | -2.4461 | -3.09538 | -4.22966 | -3.09538 | -6.25597 | -5.7784 | -5.57872 | -5.7784 |
| P62937\|PPIA_HUMAN | -3.08064 | -1.64192 | -2.54835 | -2.54835 | -4.26355 | -4.30266 | -5.03594 | -4.30266 | -8.08355 | -8.06218 | -7.50296 | -8.06218 |
| P2763\|A1AG1_HUMAN | -4.20638 | -0.58957 | -3.76226 | -3.76226 | -4.56686 | -4.94302 | -5.86231 | -4.94302 | -9.20742 | -6.5241 | -7.51305 | -7.51305 |
| O43852\|CALU_HUMAN | -2.90787 | -4.16455 | -3.23201 | -3.23201 | -3.06343 | -3.38097 | -3.33512 | -3.33512 | -6.29331 | -5.99337 | -6.25251 | -6.25251 |
| Q6MZM9\|PRR27_HUMAN | -4.84046 | -3.81319 | -4.35132 | -4.35132 | -5.30544 | -3.62927 | -4.53485 | -4.53485 | -8.26074 | -6.62679 | -7.84612 | -7.84612 |
| P2652\|APOA2_HUMAN | -4.09229 | 0.03725 | -2.69829 | -2.69829 | -4.08286 | -5.26561 | -6.18385 | -5.26561 | -6.8943 | -6.47742 | -6.72036 | -6.72036 |
| P6832\|ACTC_HUMAN | -4.01057 | -1.77493 | -3.39717 | -3.39717 | -5.17519 | -5.50575 | -9.2169 | -5.50575 | 0 | -9.03176 | -8.04811 | -8.04811 |
| P62736\|ACTA_HUMAN | -4.01057 | -1.77493 | -3.39717 | -3.39717 | -5.17519 | -5.50575 | -9.2169 | -5.50575 | 0 | -9.03176 | -8.04811 | -8.04811 |
| P63267\|ACTH_HUMAN | -4.01057 | -1.77493 | -3.39717 | -3.39717 | -5.17519 | -5.50575 | -9.2169 | -5.50575 | 0 | -9.03176 | -8.04811 | -8.04811 |
| P68133\|ACTS_HUMAN | -4.01057 | -1.77493 | -3.39717 | -3.39717 | -5.17519 | -5.50575 | -9.2169 | -5.50575 | 0 | -9.03176 | -8.04811 | -8.04811 |
| Q96KP4\|CNDP2_HUMAN | -3.19587 | -1.88806 | -2.86055 | -2.86055 | -4.61306 | -4.769 | -6.33472 | -4.769 | -9.15956 | -9.50114 | -8.02263 | -9.15956 |
| P3838\|AL3A1_HUMAN | -2.83505 | 0.22735 | -1.00714 | -1.00714 | -3.04859 | -3.28481 | -4.6667 | -3.28481 | -6.46073 | -5.59554 | -5.43866 | -5.59554 |
| P2671\|FIBA_HUMAN | -2.61609 | -0.11997 | -1.89384 | -1.89384 | -3.11388 | -3.51117 | -4.28847 | -3.51117 | -4.95341 | -3.99525 | -5.49766 | -4.95341 |
| P14555\|PA2GA_HUMAN | -3.39172 | -3.82073 | -4.97659 | -3.82073 | -3.94154 | -4.13327 | -5.32752 | -4.13327 | -2.8048 | -2.74416 | -5.20453 | -2.8048 |
| P32119\|PRDX2_HUMAN | -3.68223 | -2.19836 | -2.97332 | -2.97332 | -5.2257 | -5.98678 | -5.72793 | -5.72793 | -8.51903 | -6.14843 | -7.51376 | -7.51376 |
| P7737\|PROF1_HUMAN | -0.84879 | 1.61283 | -0.09888 | -0.09888 | -2.22868 | -2.90706 | -4.01772 | -2.90706 | -6.71003 | -5.79926 | -4.85415 | -5.79926 |
| P261\|TCO1_HUMAN | -0.84863 | -2.25648 | -0.48789 | -0.84863 | -1.54054 | -1.79753 | -1.26097 | -1.54054 | -5.84513 | -5.70197 | -5.36912 | -5.70197 |
| Q1518\|CAP1_HUMAN | -4.10699 | -2.63854 | -3.75258 | -3.75258 | -4.52674 | -4.874 | -7.85487 | -4.874 | -7.6339 | -6.49053 | -9.74509 | -7.6339 |
| P7437\|TBB5_HUMAN | -5.4478 | -4.34676 | -5.35236 | -5.35236 | -6.23557 | -6.31218 | -7.19595 | -6.31218 | -9.41934 | -8.74406 | 0 | -8.74406 |
| P178\|HV37_HUMAN | -2.99882 | -2.41586 | -3.96953 | -2.99882 | -3.63467 | -2.54883 | -4.01034 | -3.63467 | -8.20769 | -6.54984 | -5.79368 | -6.54984 |
| P1591\|IGJ_HUMAN | 0.28833 | 1.65133 | -0.50703 | 0.28833 | -0.20078 | 0.37604 | -1.03299 | -0.20078 | -3.01705 | -4.16588 | -3.70753 | -3.70753 |
| P3973\|SLPI_HUMAN | -4.18801 | -5.42479 | -4.25742 | -4.25742 | -4.75122 | -5.21102 | -4.57843 | -4.75122 | -4.44234 | -4.29277 | -4.74659 | -4.44234 |
| P35579\|MYH9_HUMAN | -7.33223 | -6.58852 | -7.3747 | -7.33223 | -8.69789 | -8.59018 | -8.97603 | -8.69789 | -9.32787 | 0 | 0 | 0 |
| P1619\|KV32_HUMAN | -4.36963 | -5.02172 | -4.68459 | -4.68459 | -5.02023 | -4.55608 | -5.11864 | -5.02023 | -8.98094 | -8.06981 | -7.76394 | -8.06981 |
| PDP24\|CALM2_HUMAN | -3.85184 | -2.25443 | -3.09267 | -3.09267 | -5.35554 | -5.64147 | -6.48767 | -5.64147 | 0 | -10.1604 | 0 | 0 |
| PDP23\|CALM1_HUMAN | -3.85184 | -2.25443 | -3.09267 | -3.09267 | -5.35554 | -5.64147 | -6.48767 | -5.64147 | 0 | -10.1604 | 0 | 0 |
| PDP25\|CALM3_HUMAN | -3.85184 | -2.25443 | -3.09267 | -3.09267 | -5.35554 | -5.64147 | -6.48767 | -5.64147 | 0 | -10.1604 | 0 | 0 |
| P385\|KCY_HUMAN | -3.23042 | -1.6057 | -2.92362 | -2.92362 | -4.54734 | -5.35251 | -7.22828 | -5.35251 | -8.65494 | -9.60849 | -8.50438 | -8.65494 |
| P61769\|B2MG_HUMAN | -0.20011 | -0.42761 | 0.79865 | -0.20011 | 0.30411 | -0.00106 | 0.4796 | 0.30411 | -3.47045 | -3.20129 | -3.0044 | -3.20129 |
| P666\|MYL6_HUMAN | -2.45197 | -0.2543 | -1.12245 | -1.12245 | -3.95192 | -3.79247 | -4.4349 | -3.95192 | -7.6674 | -6.27547 | -5.99892 | -6.27547 |
| Q9988\|H2B1L_HUMAN | -3.15251 | -0.74695 | -2.13708 | -2.13708 | -4.95899 | -4.40508 | -4.82747 | -4.82747 | -8.40952 | -7.60531 | -6.67949 | -7.60531 |
| Q99879\|H2B1M_HUMAN | -3.15251 | -0.74695 | -2.13708 | -2.13708 | -4.95899 | -4.40508 | -4.82747 | -4.82747 | -8.40952 | -7.60531 | -6.67949 | -7.60531 |
| Q9379\|H2B1H_HUMAN | -3.15251 | -0.74695 | -2.13708 | -2.13708 | -4.95899 | -4.40508 | -4.82747 | -4.82747 | -8.40952 | -7.60531 | -6.67949 | -7.60531 |
| Q5QNW6\|H2B2F_HUMAN | -3.15251 | -0.74695 | -2.13708 | -2.13708 | -4.95899 | -4.40508 | -4.82747 | -4.82747 | -8.40952 | -7.60531 | -6.67949 | -7.60531 |
| Q99877\|H2B1N_HUMAN | -3.15251 | -0.74695 | -2.13708 | -2.13708 | -4.95899 | -4.40508 | -4.82747 | -4.82747 | -8.40952 | -7.60531 | -6.67949 | -7.60531 |
| O6814\|H2B1K_HUMAN | -3.15251 | -0.74695 | -2.13708 | -2.13708 | -4.95899 | -4.40508 | -4.82747 | -4.82747 | -8.40952 | -7.60531 | -6.67949 | -7.60531 |
| P6287\|H2B1C_HUMAN | -3.15251 | -0.74695 | -2.13708 | -2.13708 | -4.95899 | -4.40508 | -4.82747 | -4.82747 | -8.40952 | -7.60531 | -6.67949 | -7.60531 |
| P58876\|H2B1D_HUMAN | -3.15251 | -0.74695 | -2.13708 | -2.13708 | -4.95899 | -4.40508 | -4.82747 | -4.82747 | -8.40952 | -7.60531 | -6.67949 | -7.60531 |
| P5753\|H2BFS_HUMAN | -3.15251 | -0.74695 | -2.13708 | -2.13708 | -4.95899 | -4.40508 | -4.82747 | -4.82747 | -8.40952 | -7.60531 | -6.67949 | -7.60531 |
| P63241\|IF5A1_HUMAN | -2.52895 | -0.96249 | -2.05679 | -2.05679 | -3.34214 | -3.19035 | -4.17709 | -3.34214 | -5.53792 | -5.11474 | -5.5545 | -5.53792 |
| Q4695\|K1C17_HUMAN | -8.8724 | -10.2555 | -8.94768 | -8.94768 | -7.70442 | -8.24043 | -7.53536 | -7.70442 | 0 | 0 | 0 | 0 |
| P18669\|PGAM1_HUMAN | -5.17302 | -3.93744 | -4.53253 | -4.53253 | -6.114 | -6.45402 | -8.10019 | -6.45402 | -9.41788 | -8.73993 | -8.52907 | -8.73993 |
| Q9BRK5\|CAB45_HUMAN | -3.54528 | -4.89461 | -3.85828 | -3.85828 | -3.35336 | -3.63047 | -3.66233 | -3.63047 | -7.20749 | -7.28309 | -7.4399 | -7.28309 |
| P31949\|S1AB_HUMAN | -3.20538 | -2.78096 | -2.9351 | -2.9351 | -3.40984 | -3.76338 | -3.39824 | -3.40984 | -6.55667 | -5.7102 | -7.04839 | -6.55667 |
| O75874\|IDHC_HUMAN | -3.23528 | -2.15519 | -2.76629 | -2.76629 | -4.12679 | -4.36187 | -4.59196 | -4.36187 | -7.61944 | -6.92702 | -6.98011 | -6.98011 |
| P68871\|HBB_HUMAN | -2.83589 | -0.19739 | -3.82131 | -2.83589 | -2.4326 | -2.77108 | -4.19635 | -2.77108 | -6.29246 | -5.54156 | -5.77775 | -5.77775 |
| P33778\|H2B1B_HUMAN | -3.3559 | -1.53778 | -2.70157 | -2.70157 | -4.01782 | -3.79017 | -5.09059 | -4.01782 | -7.58044 | -8.43505 | -7.99983 | -7.99983 |
| P6899\|H2B1J_HUMAN | -3.3559 | -1.53778 | -2.70157 | -2.70157 | -4.01782 | -3.79017 | -5.09059 | -4.01782 | -7.58044 | -8.43505 | -7.99983 | -7.99983 |
| P23527\|H2B1O_HUMAN | -3.3559 | -1.53778 | -2.70157 | -2.70157 | -4.01782 | -3.79017 | -5.09059 | -4.01782 | -7.58044 | -8.43505 | -7.99983 | -7.99983 |
| Q16778\|H2B2E_HUMAN | -3.3559 | -1.53778 | -2.70157 | -2.70157 | -4.01782 | -3.79017 | -5.09059 | -4.01782 | -7.58044 | -8.43505 | -7.99983 | -7.99983 |
| Q8N257\|H2B3B_HUMAN | -3.3559 | -1.53778 | -2.70157 | -2.70157 | -4.01782 | -3.79017 | -5.09059 | -4.01782 | -7.58044 | -8.43505 | -7.99983 | -7.99983 |
| P7384\|CAN1_HUMAN | -4.30868 | -3.73533 | -4.38107 | -4.30868 | -4.3869 | -4.80117 | -4.73458 | -4.73458 | -10.0809 | -8.74266 | -8.06737 | -8.74266 |
| Q2818\|NUCB1_HUMAN | -5.02707 | -5.65628 | -5.24026 | -5.24026 | -4.94935 | -5.25173 | -5.0924 | -5.0924 | -10.8253 | -10.0796 | -10.7864 | -10.7864 |
| Q99497\|PARK7_HUMAN | -2.30945 | -0.59815 | -1.67524 | -1.67524 | -4.62008 | -5.40007 | -6.0085 | -5.40007 | -8.79195 | -8.55843 | -6.81247 | -8.55843 |
| Q8NBJ4\|GOLM1_HUMAN | -2.53048 | -3.54568 | -3.17272 | -3.17272 | -2.56898 | -3.00054 | -3.65915 | -3.00054 | -6.02795 | -5.73846 | -5.70137 | -5.73846 |
| P5229\|6PGD_HUMAN | -8.34008 | -7.64692 | -9.50636 | -8.34008 | -8.51321 | -9.1632 | 0 | -8.51321 | 0 | 0 | 0 | 0 |
| Q13217\|DNJC3_HUMAN | -2.41302 | -3.86637 | -2.10947 | -2.41302 | -2.51736 | -2.93 | -2.78718 | -2.78718 | -6.12793 | -5.84593 | -6.19004 | -6.12793 |
| P48\|CYTB_HUMAN | -3.43118 | -1.68988 | -3.11317 | -3.11317 | -4.31998 | -4.91951 | -6.26905 | -4.91951 | -8.14521 | -8.12877 | -8.04371 | -8.12877 |
| P4121\|CAPG_HUMAN | 0 | -10.4616 | -10.1196 | -10.1196 | 0 | 0 | 0 | 0 | -10.7926 | 0 | 0 | 0 |
| P7237\|PDIA1_HUMAN | -1.97246 | -0.37294 | -0.96393 | -0.96393 | -2.37632 | -2.29504 | -3.09123 | -2.37632 | -5.84259 | -5.72326 | -4.9468 | -5.72326 |
| P28799\|GRN_HUMAN | -1.98481 | -3.20725 | -2.16323 | -2.16323 | -1.63247 | -2.51593 | -1.72023 | -1.72023 | -5.09418 | -5.30004 | -5.14434 | -5.14434 |
| P17931\|LEG3_HUMAN | -2.27366 | -0.18422 | -1.42492 | -1.42492 | -3.84119 | -4.25184 | -5.46279 | -4.25184 | -9.6995 | -8.04187 | -6.02254 | -8.04187 |
| P6285\|H4_HUMAN | -2.19082 | 0.36052 | -0.92317 | -0.92317 | -2.81121 | -2.79083 | -4.09524 | -2.81121 | -6.15973 | -5.57031 | -4.78648 | -5.57031 |
| P36952\|SPB5_HUMAN | -4.9265 | -5.20608 | -4.49428 | -4.9265 | -6.17538 | -6.40484 | -6.86945 | -6.40484 | -7.8595 | -6.31807 | -7.93975 | -7.8595 |
| P13489\|RINI_HUMAN | -5.65686 | -4.25174 | -4.72275 | -4.72275 | -6.41933 | -6.56551 | -7.26404 | -6.56551 | -10.2522 | 0 | -10.2913 | -10.2522 |
| P62258\|1433E_HUMAN | -5.76807 | -4.80162 | -4.93554 | -4.93554 | -6.36577 | -7.06988 | -8.67799 | -7.06988 | 0 | 0 | 0 | 0 |
| P59\|APOD_HUMAN | -4.64694 | -3.08251 | -1.58854 | -3.08251 | -4.6565 | -4.3431 | -4.71309 | -4.6565 | -7.92888 | -6.35312 | -5.77889 | -6.35312 |
| P18\|ANT3_HUMAN | -5.34164 | -2.82877 | -4.59283 | -4.59283 | -5.60432 | -6.52527 | -6.45363 | -6.45363 | 0 | 0 | -8.69566 | 0 |
| P44\|VTNC_HUMAN | -5.96269 | -3.80583 | -5.04828 | -5.04828 | -5.95164 | -6.07303 | -6.23902 | -6.07303 | -10.4414 | -7.949 | -7.62652 | -7.949 |
| P5395\|GDIB_HUMAN | -5.53598 | -3.59295 | -4.71589 | -4.71589 | -6.17788 | -7.03392 | 0 | -6.17788 | -9.59544 | 0 | 0 | 0 |
| P35241\|RADI_HUMAN | 0 | -10.4312 | -9.8533 | -9.8533 | 0 | 0 | 0 | 0 | -9.90904 | 0 | 0 | 0 |
| P2766\|TTHY_HUMAN | -2.47409 | 1.41093 | -0.92846 | -0.92846 | -2.29764 | -2.65273 | -3.47722 | -2.65273 | -5.7623 | -5.14209 | -6.41639 | -5.7623 |
| Q14764\|MVP_HUMAN | -4.0118 | -3.66413 | -3.67641 | -3.67641 | -4.14668 | -4.14252 | -4.22831 | -4.14668 | -7.57926 | -7.16099 | -8.0586 | -7.57926 |
| P8748\|LV321_HUMAN | -8.19506 | -10.0019 | 0 | -8.19506 | -7.64612 | -7.47972 | -9.21778 | -7.64612 | 0 | 0 | 0 | 0 |
| P1782\|HV39_HUMAN | -4.21789 | -1.85246 | -4.64407 | -4.21789 | -4.2384 | -2.40439 | -4.69918 | -4.2384 | -7.08545 | -7.34863 | -7.78425 | -7.34863 |
| P8571\|CD14_HUMAN | -3.42853 | -4.19838 | -3.42322 | -3.42853 | -3.51421 | -3.43203 | -4.0329 | -3.51421 | -7.90784 | -7.41474 | -7.35163 | -7.41474 |
| P4217\|A1BG_HUMAN | -4.39968 | -2.60967 | -4.23168 | -4.23168 | -4.6171 | -4.86943 | -4.75353 | -4.75353 | -7.39103 | -7.24644 | -6.8322 | -7.24644 |
| P338\|LDHA_HUMAN | -0.89231 | 1.38225 | 0.0579 | 0.0579 | -2.39761 | -2.75141 | -4.01673 | -2.75141 | -5.35891 | -5.44794 | -4.40241 | -5.35891 |
| P2765\|FETUA_HUMAN | -4.75269 | -1.65445 | -3.85891 | -3.85891 | -4.36051 | -5.31169 | -7.67312 | -5.31169 | 0 | -10.1519 | -9.46681 | -9.46681 |
| P1283\|CADH1_HUMAN | -3.09243 | -4.24869 | -3.06103 | -3.09243 | -3.30676 | -3.50134 | -3.61797 | -3.50134 | -3.23283 | -3.22408 | -3.75753 | -3.23283 |
| P5995\|ANX11_HUMAN | -3.37763 | -3.13532 | -3.63823 | -3.37763 | -4.31274 | -4.1641 | -5.47435 | -4.31274 | -7.1473 | -7.06174 | -7.23685 | -7.1473 |
| P22392\|NDKB_HUMAN | -3.66641 | -1.99592 | -3.08654 | -3.08654 | -5.66761 | -6.11433 | 0 | -5.66761 | 0 | 0 | -8.9513 | 0 |
| Q4828\|AK1C1_HUMAN | -2.43439 | -0.42166 | -1.64677 | -1.64677 | -4.58329 | -4.86699 | -5.32735 | -4.86699 | -7.74784 | -6.48082 | -5.22982 | -6.48082 |
| P27348\|1433T_HUMAN | -6.68826 | -5.275 | -5.7885 | -5.7885 | -7.03462 | -7.39142 | -7.65225 | -7.39142 | 0 | 0 | 0 | 0 |
| P1851\|IL1RA_HUMAN | -7.36285 | -8.11776 | -7.71152 | -7.71152 | -7.99977 | -8.74071 | 0 | -7.99977 | 0 | -9.13888 | 0 | 0 |
| P2638\|MOES_HUMAN | 0 | -10.6271 | -9.52132 | -9.52132 | -8.54507 | 0 | 0 | 0 | 0 | 0 | 0 | 0 |
| P631\|KV23_HUMAN | -8.75366 | -8.18573 | -10.7289 | -8.75366 | -8.96313 | -7.49204 | 0 | -7.49204 | 0 | -9.18177 | 0 | 0 |
| AA75B6S6\|KVD3_HUMAN | -8.75366 | -8.18573 | -10.7289 | -8.75366 | -8.96313 | -7.49204 | 0 | -7.49204 | 0 | -9.18177 | 0 | 0 |
| P2941\|TKT_HUMAN | -5.22644 | -4.94048 | -4.76246 | -4.94048 | -6.13978 | -5.784 | -6.73155 | -6.13978 | -8.47538 | -7.6216 | -7.38677 | -7.6216 |
| O299\|CLIC1_HUMAN | -3.58086 | -1.57638 | -3.08394 | -3.08394 | -3.76348 | -4.16494 | -5.65048 | -4.16494 | -7.62077 | -7.73831 | -8.43225 | -7.73831 |
| P311\|PDIA3_HUMAN | -8.96171 | -7.54752 | -7.57318 | -7.57318 | -8.67064 | -8.9977 | 0 | -8.67064 | 0 | -10.1221 | 0 | 0 |
| P863\|CFAH_HUMAN | -1.99068 | -0.23762 | -1.6289 | -1.6289 | -2.31514 | -3.16631 | -2.90372 | -2.90372 | -4.83726 | -4.67735 | -4.06903 | -4.67735 |
| AAC4DH38\|HV551_HUMAN | -1.35152 | -1.215 | -2.32203 | -1.35152 | -1.85016 | -1.44418 | -2.14196 | -1.85016 | -5.78606 | -6.29159 | -4.71453 | -5.78606 |
| PCG47\|UBB_HUMAN | -4.06635 | -2.75309 | -3.15333 | -3.15333 | -5.2207 | -5.21479 | -5.67346 | -5.2207 | -8.70096 | -6.93986 | -7.42749 | -7.42749 |
| PCG48\|UBC_HUMAN | -4.06635 | -2.75309 | -3.15333 | -3.15333 | -5.2207 | -5.21479 | -5.67346 | -5.2207 | -8.70096 | -6.93986 | -7.42749 | -7.42749 |
| P62987\|RL4_HUMAN | -4.06635 | -2.75309 | -3.15333 | -3.15333 | -5.2207 | -5.21479 | -5.67346 | -5.2207 | -8.70096 | -6.93986 | -7.42749 | -7.42749 |
| P62979\|RS27A_HUMAN | -4.06635 | -2.75309 | -3.15333 | -3.15333 | -5.2207 | -5.21479 | -5.67346 | -5.2207 | -8.70096 | -6.93986 | -7.42749 | -7.42749 |
| P19971\|TYPH_HUMAN | -4.61354 | -3.64781 | -5.00049 | -4.61354 | -4.0336 | -3.94647 | -4.82055 | -4.0336 | -6.24311 | -6.9679 | -7.50844 | -6.9679 |
| P718\|ACBP_HUMAN | -5.37814 | -4.84887 | -5.43412 | -5.37814 | -7.37993 | -7.59092 | -8.91661 | -7.59092 | 0 | -9.00275 | 0 | 0 |
| P275\|A2GL_HUMAN | -4.65151 | -6.23256 | -5.61744 | -5.61744 | -4.85851 | -5.44927 | -4.82213 | -4.85851 | -7.8271 | -7.81283 | -8.51126 | -7.8271 |
| P1615\|KVD28_HUMAN | -8.86013 | -9.54651 | -8.80904 | -8.86013 | -7.97824 | -7.58515 | -7.48925 | -7.58515 | 0 | -9.20159 | 0 | 0 |
| AA75B6P5\|KV228_HUMAN | -8.86013 | -9.54651 | -8.80904 | -8.86013 | -7.97824 | -7.58515 | -7.48925 | -7.58515 | 0 | -9.20159 | 0 | 0 |
| P1593\|KVD33_HUMAN | -5.33295 | -5.75361 | -6.2016 | -5.75361 | -5.21691 | -4.66399 | -5.10591 | -5.10591 | -7.75098 | -7.80888 | -7.36127 | -7.75098 |
| P1594\|KV133_HUMAN | -5.33295 | -5.75361 | -6.2016 | -5.75361 | -5.21691 | -4.66399 | -5.10591 | -5.10591 | -7.75098 | -7.80888 | -7.36127 | -7.75098 |
| Q9Y49\|TLN1_HUMAN | -9.22965 | -8.09689 | -8.89615 | -8.89615 | -9.36041 | 0 | -9.28253 | -9.28253 | 0 | 0 | 0 | 0 |
| P395\|ANGI_HUMAN | -6.74375 | -8.59114 | -7.26217 | -7.26217 | -6.80767 | -6.84803 | -6.99526 | -6.84803 | -7.22485 | -6.50944 | -7.61887 | -7.22485 |
| Q96S96\|PEBP4_HUMAN | -4.82387 | -6.38436 | -6.70208 | -6.38436 | -4.69406 | -4.44151 | -6.08514 | -4.69406 | -7.13125 | -7.26156 | -7.75731 | -7.26156 |
| P47895\|AL1A3_HUMAN | -3.3806 | -2.75961 | -2.92791 | -2.92791 | -4.87499 | -4.96882 | -5.72229 | -4.96882 | -7.4534 | -6.33715 | -6.66931 | -6.66931 |
| AA75B6K4\|LV31_HUMAN | -3.88331 | -4.34496 | -5.18356 | -4.34496 | -3.70924 | -3.36667 | -4.65285 | -3.70924 | -7.01194 | -6.85671 | -8.75383 | -7.01194 |
| P1717\|LV325_HUMAN | -3.88331 | -4.34496 | -5.18356 | -4.34496 | -3.70924 | -3.36667 | -4.65285 | -3.70924 | -7.01194 | -6.85671 | -8.75383 | -7.01194 |
| P9467\|F16P1_HUMAN | -3.09735 | -1.94171 | -2.41755 | -2.41755 | -4.58169 | -4.92943 | -6.51481 | -4.92943 | 0 | 0 | -7.65588 | 0 |
| P1641\|H15_HUMAN | -5.62238 | -4.34832 | -5.05947 | -5.05947 | -5.75501 | -5.23423 | -6.10043 | -5.75501 | -7.0525 | -6.65492 | -6.70977 | -6.70977 |
| P751\|CFAB_HUMAN | -5.59517 | -4.62106 | -6.18928 | -5.59517 | -5.6392 | -6.28273 | -6.63632 | -6.28273 | -9.52612 | -9.09417 | -8.97567 | -9.09417 |
| P17\|LV147_HUMAN | -5.07218 | -4.2329 | -6.6171 | -5.07218 | -4.46493 | -2.98878 | -5.25776 | -4.46493 | -7.57164 | -7.92118 | -8.6054 | -7.92118 |
| P39\|GSHR_HUMAN | -4.05245 | -2.89412 | -3.36072 | -3.36072 | -5.66013 | -6.14894 | -6.28329 | -6.14894 | -8.69623 | -8.43548 | -7.94105 | -8.43548 |
| P14625\|ENPL_HUMAN | -6.57894 | -5.85906 | -5.96837 | -5.96837 | -7.52225 | -7.28057 | -7.53265 | -7.52225 | 0 | 0 | -10.7374 | 0 |
| P5572\|TERA_HUMAN | -2.90224 | -1.3207 | -2.23943 | -2.23943 | -3.65234 | -3.47523 | -4.69318 | -3.65234 | -7.55148 | -7.26843 | -6.06658 | -7.26843 |
| Q13162\|PRDX4_HUMAN | -3.42681 | -3.75767 | -3.84679 | -3.75767 | -3.65949 | -3.87287 | -5.06924 | -3.87287 | -6.15904 | -7.1915 | -6.83816 | -6.83816 |
| P5558\|PLTP_HUMAN | -4.95708 | -6.74178 | -5.34565 | -5.34565 | -4.95186 | -5.01391 | -4.99522 | -4.99522 | 0 | 0 | 0 | 0 |
| P5155\|IC1_HUMAN | -5.86624 | -5.01878 | -5.96915 | -5.86624 | -5.83815 | -6.49426 | -6.74507 | -6.49426 | -6.98393 | -5.98325 | -7.04558 | -6.98393 |
| P4694\|IQGA1_HUMAN | -9.41791 | -8.77582 | -8.72327 | -8.77582 | 0 | 0 | 0 | 0 | 0 | 0 | -10.6391 | 0 |
| P1825\|HV459_HUMAN | -3.6832 | -3.94934 | -5.68431 | -3.94934 | -4.30803 | -3.50743 | -5.24216 | -4.30803 | -10.1132 | -9.27414 | -8.65324 | -9.27414 |
| PDP8\|HVD82_HUMAN | -3.6832 | -3.94934 | -5.68431 | -3.94934 | -4.30803 | -3.50743 | -5.24216 | -4.30803 | -10.1132 | -9.27414 | -8.65324 | -9.27414 |
| PDP7\|HV431_HUMAN | -3.6832 | -3.94934 | -5.68431 | -3.94934 | -4.30803 | -3.50743 | -5.24216 | -4.30803 | -10.1132 | -9.27414 | -8.65324 | -9.27414 |
| AAC4DH41\|HV461_HUMAN | -3.6832 | -3.94934 | -5.68431 | -3.94934 | -4.30803 | -3.50743 | -5.24216 | -4.30803 | -10.1132 | -9.27414 | -8.65324 | -9.27414 |
| PDP6\|HVD34_HUMAN | -3.6832 | -3.94934 | -5.68431 | -3.94934 | -4.30803 | -3.50743 | -5.24216 | -4.30803 | -10.1132 | -9.27414 | -8.65324 | -9.27414 |
| P1824\|HV439_HUMAN | -3.6832 | -3.94934 | -5.68431 | -3.94934 | -4.30803 | -3.50743 | -5.24216 | -4.30803 | -10.1132 | -9.27414 | -8.65324 | -9.27414 |
| P52566\|GDIR2_HUMAN | -6.7348 | -6.94431 | -7.72739 | -6.94431 | -8.13807 | 0 | 0 | 0 | 0 | 0 | 0 | 0 |
| AAAMS15\|HV349_HUMAN | -6.97263 | -7.74095 | -8.82466 | -7.74095 | -6.52915 | -6.06111 | -7.21564 | -6.52915 | -10.0901 | -9.95817 | -8.60696 | -9.95817 |
| Q9HC38\|GLOD4_HUMAN | -5.81317 | -5.22684 | -5.64317 | -5.64317 | -6.26842 | -6.86364 | -7.25381 | -6.86364 | -8.61499 | -8.16336 | -8.99349 | -8.61499 |
| AAB4J2D9\|KVD13_HUMAN | -2.75051 | -4.75787 | -5.67367 | -4.75787 | -2.66877 | -4.52281 | -4.49548 | -4.49548 | -6.95192 | -7.51541 | -7.424 | -7.424 |
| PDP9\|KV113_HUMAN | -2.75051 | -4.75787 | -5.67367 | -4.75787 | -2.66877 | -4.52281 | -4.49548 | -4.49548 | -6.95192 | -7.51541 | -7.424 | -7.424 |
| O4377\|ACTN4_HUMAN | -5.05472 | -4.93146 | -5.03031 | -5.03031 | -5.49553 | -5.2274 | -5.72069 | -5.49553 | 0 | -10.0747 | 0 | 0 |
| Q99538\|LGMN_HUMAN | -7.37858 | -9.71911 | -7.36642 | -7.37858 | -7.60016 | -7.5799 | -7.77561 | -7.60016 | 0 | 0 | 0 | 0 |
| P16152\|CBR1_HUMAN | -5.45722 | -5.15239 | -5.70244 | -5.45722 | -6.45654 | -6.57982 | -7.58191 | -6.57982 | -9.9485 | -9.76377 | -10.3501 | -9.9485 |
| P31151\|S1A7_HUMAN | -4.75402 | -5.86915 | -3.99324 | -4.75402 | -4.91342 | -4.38144 | -5.17718 | -4.91342 | -6.40737 | -2.83021 | -5.9105 | -5.9105 |
| P26447\|S1A4_HUMAN | 0.31436 | 2.87165 | 1.54195 | 1.54195 | -1.80363 | -2.32455 | -4.85476 | -2.32455 | -8.07589 | -6.50638 | -4.67425 | -6.50638 |
| P6753\|TPM3_HUMAN | -3.28027 | -1.34859 | -2.67432 | -2.67432 | -4.14132 | -4.30778 | -5.13221 | -4.30778 | -5.10932 | -4.25015 | -4.62408 | -4.62408 |
| AAC4DH32\|HV32_HUMAN | -4.6839 | -4.54099 | -4.7912 | -4.6839 | -4.25327 | -3.5008 | -4.34853 | -4.25327 | -6.26059 | -8.15323 | -6.75067 | -6.75067 |
| P133\|TIMP1_HUMAN | -3.47912 | -4.42124 | -3.61874 | -3.61874 | -3.50613 | -3.7909 | -3.50687 | -3.50687 | -6.90695 | -5.9535 | -6.11968 | -6.11968 |
| P4925\|MDHC_HUMAN | -4.26546 | -3.06983 | -3.7455 | -3.7455 | -5.30207 | -4.77944 | -6.40026 | -5.30207 | 0 | -8.58444 | -9.70804 | -8.58444 |
| P2575\|ATPA_HUMAN | -4.69076 | -3.21151 | -3.88575 | -3.88575 | -5.39858 | -5.54752 | -6.21981 | -5.54752 | -7.12383 | -6.80329 | -6.67218 | -6.80329 |
| P22626\|ROA2_HUMAN | -3.90834 | -3.92561 | -4.22645 | -3.92561 | -4.48611 | -4.16303 | -5.5091 | -4.48611 | -8.92695 | -7.73828 | -7.10462 | -7.73828 |
| P1921\|AMD_HUMAN | -5.19667 | -7.11273 | -5.53719 | -5.53719 | -5.72303 | -5.89679 | -6.16868 | -5.89679 | -10.9329 | -8.71572 | 0 | -8.71572 |
| P2545\|LMNA_HUMAN | -5.08445 | -4.49885 | -4.30695 | -4.49885 | -6.14446 | -5.63153 | -6.19092 | -6.14446 | -8.47499 | -7.64873 | -7.55596 | -7.64873 |
| P8165\|DCD_HUMAN | -4.98221 | -6.03708 | -5.17462 | -5.17462 | -4.47614 | -4.40205 | -4.90928 | -4.47614 | -9.57982 | -8.43924 | -8.87504 | -8.87504 |
| P22314\|UBA1_HUMAN | -7.82492 | -8.24475 | -8.09551 | -8.09551 | -8.4527 | -8.30886 | 0 | -8.30886 | -10.9343 | -10.0967 | 0 | -10.0967 |
| P48643\|TCPE_HUMAN | -9.08557 | -9.76297 | -9.99316 | -9.76297 | 0 | 0 | 0 | 0 | -10.9872 | 0 | -10.7224 | -10.7224 |
| P1624\|KV315_HUMAN | -4.08699 | -5.08172 | -4.56175 | -4.56175 | -4.45065 | -3.96695 | -4.5509 | -4.45065 | -8.23568 | -7.86535 | -8.85666 | -8.23568 |
| Q8TCD5\|NT5C_HUMAN | -9.92181 | -8.57374 | -9.11985 | -9.11985 | 0 | 0 | 0 | 0 | 0 | 0 | 0 | 0 |
| P3496\|RNAS4_HUMAN | -5.38221 | -6.11669 | -6.20879 | -6.11669 | -5.62222 | -5.42678 | -6.83783 | -5.62222 | -6.00837 | -5.46339 | -5.16083 | -5.46339 |
| P6312\|KV41_HUMAN | -3.42999 | -3.86621 | -3.69873 | -3.69873 | -3.27352 | -2.78186 | -3.23435 | -3.23435 | -7.23921 | -7.38047 | -6.54187 | -7.23921 |
| Q14118\|DAG1_HUMAN | -5.74637 | -7.87516 | -5.83473 | -5.83473 | -6.22895 | -6.47152 | -6.30094 | -6.30094 | 0 | -9.8522 | 0 | 0 |
| O151\|PDLI1_HUMAN | -5.66693 | -3.93727 | -4.60966 | -4.60966 | -7.0885 | -6.78633 | -8.71046 | -7.0885 | 0 | 0 | 0 | 0 |
| P5297\|CAZA1_HUMAN | -4.48396 | -3.41746 | -3.86215 | -3.86215 | -5.29417 | -5.47224 | -6.00987 | -5.47224 | -8.85355 | -7.27774 | -7.94864 | -7.94864 |
| AAC4DH69\|KV19_HUMAN | -7.11981 | -7.82031 | -7.79626 | -7.79626 | -6.90678 | -5.8554 | -6.77223 | -6.77223 | 0 | 0 | 0 | 0 |
| P9651\|ROA1_HUMAN | -6.77981 | -5.92031 | -6.28429 | -6.28429 | -7.70484 | -7.89945 | -8.24837 | -7.89945 | -9.23244 | -8.15681 | -10.2841 | -9.23244 |
| Q32P51\|RA1L2_HUMAN | -6.77981 | -5.92031 | -6.28429 | -6.28429 | -7.70484 | -7.89945 | -8.24837 | -7.89945 | -9.23244 | -8.15681 | -10.2841 | -9.23244 |
| Q99878\|H2A1J_HUMAN | -7.72394 | -6.86261 | -6.89785 | -6.89785 | 0 | 0 | 0 | 0 | -10.9184 | 0 | 0 | 0 |
| Q96KK5\|H2A1H_HUMAN | -7.72394 | -6.86261 | -6.89785 | -6.89785 | 0 | 0 | 0 | 0 | -10.9184 | 0 | 0 | 0 |
| Q16777\|H2A2C_HUMAN | -7.72394 | -6.86261 | -6.89785 | -6.89785 | 0 | 0 | 0 | 0 | -10.9184 | 0 | 0 | 0 |
| Q9BTM1\|H2AJ_HUMAN | -7.72394 | -6.86261 | -6.89785 | -6.89785 | 0 | 0 | 0 | 0 | -10.9184 | 0 | 0 | 0 |
| P498\|H2A1B_HUMAN | -7.72394 | -6.86261 | -6.89785 | -6.89785 | 0 | 0 | 0 | 0 | -10.9184 | 0 | 0 | 0 |
| Q6FI13\|H2A2A_HUMAN | -7.72394 | -6.86261 | -6.89785 | -6.89785 | 0 | 0 | 0 | 0 | -10.9184 | 0 | 0 | 0 |
| Q7L7L\|H2A3_HUMAN | -7.72394 | -6.86261 | -6.89785 | -6.89785 | 0 | 0 | 0 | 0 | -10.9184 | 0 | 0 | 0 |
| Q9377\|H2A1C_HUMAN | -7.72394 | -6.86261 | -6.89785 | -6.89785 | 0 | 0 | 0 | 0 | -10.9184 | 0 | 0 | 0 |
| PCS8\|H2A1_HUMAN | -7.72394 | -6.86261 | -6.89785 | -6.89785 | 0 | 0 | 0 | 0 | -10.9184 | 0 | 0 | 0 |
| P2671\|H2A1D_HUMAN | -7.72394 | -6.86261 | -6.89785 | -6.89785 | 0 | 0 | 0 | 0 | -10.9184 | 0 | 0 | 0 |
| Q8IUE6\|H2A2B_HUMAN | -7.72394 | -6.86261 | -6.89785 | -6.89785 | 0 | 0 | 0 | 0 | -10.9184 | 0 | 0 | 0 |
| P1614\|H2AX_HUMAN | -7.72394 | -6.86261 | -6.89785 | -6.89785 | 0 | 0 | 0 | 0 | -10.9184 | 0 | 0 | 0 |
| Q96QV6\|H2A1A_HUMAN | -7.72394 | -6.86261 | -6.89785 | -6.89785 | 0 | 0 | 0 | 0 | -10.9184 | 0 | 0 | 0 |
| Q92743\|HTRA1_HUMAN | -3.77108 | -5.88187 | -4.13034 | -4.13034 | -4.17554 | -4.6776 | -4.61375 | -4.61375 | -7.47395 | -7.06354 | -7.78995 | -7.47395 |
| AA75B6R9\|KVD24_HUMAN | -4.91741 | -5.63193 | -5.76279 | -5.63193 | -4.94029 | -5.48718 | -5.62983 | -5.48718 | 0 | 0 | -9.17481 | 0 |
| AAC4DH68\|KV224_HUMAN | -4.91741 | -5.63193 | -5.76279 | -5.63193 | -4.94029 | -5.48718 | -5.62983 | -5.48718 | 0 | 0 | -9.17481 | 0 |
| P52565\|GDIR1_HUMAN | -3.8631 | -2.56317 | -3.61807 | -3.61807 | -5.38132 | -5.50049 | -6.39981 | -5.50049 | -7.848 | -7.20373 | -7.2355 | -7.2355 |
| P49189\|AL9A1_HUMAN | -3.69227 | -3.33553 | -3.13838 | -3.33553 | -5.02724 | -4.81432 | -4.56948 | -4.81432 | -6.18885 | -6.14519 | -5.38829 | -6.14519 |
| P1714\|LV319_HUMAN | -8.35906 | -9.62096 | 0 | -8.35906 | -7.68741 | -7.28748 | -8.56932 | -7.68741 | -10.8659 | 0 | -10.6175 | -10.6175 |
| P7339\|CATD_HUMAN | -5.29699 | -6.29542 | -5.21966 | -5.29699 | -5.49874 | -6.33458 | -6.79515 | -6.33458 | -8.68265 | -7.92496 | -7.85172 | -7.92496 |
| Q15181\|IPYR_HUMAN | -8.00692 | -6.78319 | -7.01075 | -7.01075 | -8.70512 | -8.10311 | 0 | -8.10311 | 0 | 0 | 0 | 0 |
| Q8WVQ1\|CANT1_HUMAN | -2.93491 | -4.36399 | -2.88189 | -2.93491 | -3.61538 | -3.4752 | -4.01132 | -3.61538 | -7.05429 | -6.73206 | -5.92792 | -6.73206 |
| P142\|KNG1_HUMAN | -8.76679 | -7.13304 | -9.08894 | -8.76679 | -7.80418 | 0 | -9.20347 | -7.80418 | 0 | 0 | 0 | 0 |
| P6576\|ATPB_HUMAN | -8.42603 | -7.90154 | -8.03947 | -8.03947 | 0 | 0 | 0 | 0 | 0 | 0 | -10.5015 | 0 |
| O7583\|WDR1_HUMAN | -4.68636 | -3.97575 | -4.73557 | -4.68636 | -5.95385 | -6.04039 | -6.83239 | -6.04039 | -7.24204 | -6.12437 | -6.60343 | -6.60343 |
| P1455\|AK1A1_HUMAN | -3.76879 | -2.9595 | -3.8705 | -3.76879 | -4.12054 | -4.07281 | -4.85261 | -4.12054 | -7.90017 | -8.77822 | -8.62298 | -8.62298 |
| P17987\|TCPA_HUMAN | -7.21589 | -8.52037 | -8.15038 | -8.15038 | -7.6737 | -9.25614 | -7.91841 | -7.91841 | 0 | 0 | 0 | 0 |
| P17858\|PFKAL_HUMAN | -6.78633 | -6.42536 | -6.52449 | -6.52449 | -7.5168 | -7.65569 | -7.34926 | -7.5168 | -10.978 | -10.1261 | 0 | -10.1261 |
| P9429\|HMGB1_HUMAN | -2.72906 | -0.6883 | -1.83982 | -1.83982 | -4.00089 | -4.05922 | -4.30905 | -4.05922 | -7.33272 | -6.45679 | -5.31499 | -6.45679 |
| P4939\|ECHA_HUMAN | -7.64552 | -6.76666 | -7.26512 | -7.26512 | -7.84897 | 0 | -8.52182 | -7.84897 | 0 | 0 | 0 | 0 |
| P867\|VIME_HUMAN | -4.08957 | -2.4132 | -3.21212 | -3.21212 | -4.62955 | -4.74563 | -5.27243 | -4.74563 | -7.609 | -5.97339 | -7.09018 | -7.09018 |
| P25815\|S1P_HUMAN | -5.09405 | -3.66201 | -3.51026 | -3.66201 | -6.67909 | -6.06447 | -8.10822 | -6.67909 | 0 | 0 | -9.89928 | 0 |
| P167\|CD44_HUMAN | -4.03786 | -4.23064 | -4.27339 | -4.23064 | -4.03449 | -4.4218 | -4.89602 | -4.4218 | -7.73742 | -7.08727 | -7.32804 | -7.32804 |
| PCL4\|CO4A_HUMAN | -4.39691 | -2.52959 | -4.72385 | -4.39691 | -4.59281 | -3.93723 | -5.50852 | -4.59281 | -7.8286 | -8.274 | -8.10295 | -8.10295 |
| PCL5\|CO4B_HUMAN | -4.39691 | -2.52959 | -4.72385 | -4.39691 | -4.59281 | -3.93723 | -5.50852 | -4.59281 | -7.8286 | -8.274 | -8.10295 | -8.10295 |
| Q621\|GFPT1_HUMAN | 0 | -9.23594 | -10.6764 | -9.23594 | 0 | 0 | 0 | 0 | -11.089 | 0 | 0 | 0 |
| O9488\|GFPT2_HUMAN | 0 | -9.23594 | -10.6764 | -9.23594 | 0 | 0 | 0 | 0 | -11.089 | 0 | 0 | 0 |
| Q9UQ8\|PA2G4_HUMAN | -9.39452 | -8.87125 | -9.09304 | -9.09304 | 0 | 0 | 0 | 0 | 0 | 0 | 0 | 0 |
| Q99954\|SMR3A_HUMAN | -2.99023 | -3.98673 | -2.71475 | -2.99023 | -3.52524 | -3.31939 | -4.12936 | -3.52524 | -6.19451 | -6.23112 | -4.99032 | -6.19451 |
| P13797\|PLST_HUMAN | -8.43976 | -8.24807 | -8.19685 | -8.24807 | 0 | 0 | 0 | 0 | 0 | 0 | 0 | 0 |
| P4926\|MDHM_HUMAN | -6.59889 | -5.031 | -5.15292 | -5.15292 | -6.95143 | -7.3202 | -8.91902 | -7.3202 | -9.59119 | 0 | -10.3584 | -9.59119 |
| P19652\|A1AG2_HUMAN | -6.60166 | -4.00204 | -6.24084 | -6.24084 | -5.39072 | -6.03488 | -7.74502 | -6.03488 | -10.1609 | -9.10651 | 0 | -9.10651 |
| P1611\|KVD12_HUMAN | -5.46278 | -5.82796 | -7.41058 | -5.82796 | -5.54835 | -4.83312 | -6.87871 | -5.54835 | -9.0506 | -9.02947 | -9.05214 | -9.0506 |
| AAC4DH73\|KV112_HUMAN | -5.46278 | -5.82796 | -7.41058 | -5.82796 | -5.54835 | -4.83312 | -6.87871 | -5.54835 | -9.0506 | -9.02947 | -9.05214 | -9.0506 |
| P62277\|RS13_HUMAN | -7.11051 | -6.2842 | -6.70833 | -6.70833 | -7.72825 | -7.95427 | -8.6759 | -7.95427 | 0 | -9.82299 | -10.474 | -9.82299 |
| Q14697\|GANAB_HUMAN | -4.73844 | -5.23325 | -5.23397 | -5.23325 | -4.89388 | -5.01378 | -5.48222 | -5.01378 | -7.75449 | -7.21363 | -7.83419 | -7.75449 |
| P13693\|TCTP_HUMAN | -6.15378 | -5.44717 | -6.20573 | -6.15378 | -7.34315 | -6.96185 | 0 | -6.96185 | -9.42767 | -9.21518 | -9.64669 | -9.42767 |
| O462\|MANBA_HUMAN | -6.23228 | -7.80017 | -7.0506 | -7.0506 | -6.64628 | -6.53836 | -6.59649 | -6.59649 | -8.84596 | -9.22571 | -10.3895 | -9.22571 |
| P27797\|CALR_HUMAN | -8.00218 | -7.98184 | -7.54986 | -7.98184 | -8.84188 | -8.49593 | -9.22367 | -8.84188 | 0 | 0 | 0 | 0 |
| P8582\|TRFM_HUMAN | -2.85315 | -4.61702 | -4.16012 | -4.16012 | -4.12426 | -4.40465 | -3.69273 | -4.12426 | -7.80336 | -6.69718 | -7.78324 | -7.78324 |
| Q7Z5P9\|MUC19_HUMAN | -8.21722 | -9.37711 | -9.0456 | -9.0456 | -7.52225 | -8.35004 | -7.99828 | -7.99828 | -8.73927 | -8.48183 | -8.00394 | -8.48183 |
| P2511\|CRYAB_HUMAN | -4.67734 | -3.4128 | -3.74069 | -3.74069 | -6.58317 | -6.33675 | -7.11941 | -6.58317 | -9.68339 | -7.88328 | -8.37849 | -8.37849 |
| P49788\|TIG1_HUMAN | -6.77233 | -8.09757 | -5.98825 | -6.77233 | -7.07204 | -7.07724 | -6.03686 | -7.07204 | 0 | 0 | -9.65202 | 0 |
| P19827\|ITIH1_HUMAN | -7.82889 | -5.64111 | -7.30424 | -7.30424 | -7.65521 | -7.72798 | -8.93348 | -7.72798 | 0 | -9.2288 | -10.1475 | -9.2288 |
| P8758\|ANXA5_HUMAN | -4.72473 | -6.32035 | -4.90803 | -4.90803 | -4.68491 | -4.78235 | -4.44442 | -4.68491 | -6.30165 | -8.77229 | -9.42357 | -8.77229 |
| Q9BS4\|LXN_HUMAN | -5.92954 | -6.14383 | -6.06199 | -6.06199 | -7.30733 | -7.03468 | -9.16423 | -7.30733 | 0 | 0 | 0 | 0 |
| Q9H8\|LHPP_HUMAN | -8.93021 | -7.6112 | -7.9758 | -7.9758 | -9.21508 | 0 | 0 | 0 | 0 | -10.1896 | 0 | 0 |
| AA87WSZ\|KVD8_HUMAN | -9.29994 | -10.2026 | -8.06744 | -9.29994 | -7.95491 | -8.13112 | -7.71148 | -7.95491 | 0 | 0 | -9.5508 | 0 |
| Q1556\|IF4H_HUMAN | -7.45278 | -8.58722 | -8.64793 | -8.58722 | -8.02575 | -9.15209 | 0 | -8.02575 | -10.9482 | 0 | 0 | 0 |
| O95834\|EMAL2_HUMAN | -7.48369 | -6.80949 | -7.48634 | -7.48369 | -8.88861 | 0 | 0 | 0 | 0 | 0 | 0 | 0 |
| P21964\|COMT_HUMAN | -6.35611 | -5.77027 | -5.44046 | -5.77027 | -6.16674 | -6.21539 | -7.14129 | -6.21539 | -7.98101 | -6.82044 | -8.0907 | -7.98101 |
| P9525\|ANXA4_HUMAN | -7.12144 | -6.89456 | -7.15964 | -7.12144 | -7.54662 | -7.51202 | -7.8965 | -7.54662 | -10.3807 | -9.27056 | 0 | -9.27056 |
| P436\|PSB1_HUMAN | -8.02037 | -8.27344 | -8.94953 | -8.27344 | -7.88466 | -7.68758 | -8.50941 | -7.88466 | -11.0214 | 0 | -9.5382 | -9.5382 |
| P25789\|PSA4_HUMAN | -5.78112 | -4.57193 | -5.24869 | -5.24869 | -5.94809 | -5.90676 | -6.95559 | -5.94809 | -7.20421 | -6.69787 | -6.84589 | -6.84589 |
| Q182\|SPTB2_HUMAN | -9.56688 | -8.71909 | -9.26353 | -9.26353 | 0 | 0 | 0 | 0 | 0 | 0 | 0 | 0 |
| P2814\|SMR3B_HUMAN | -4.84403 | -8.3415 | -8.25734 | -8.25734 | -7.3178 | -6.38675 | -7.02749 | -7.02749 | -6.39996 | -6.06965 | -5.07071 | -6.06965 |
| P17812\|PYRG1_HUMAN | -5.01961 | -6.61698 | -5.38612 | -5.38612 | -4.72002 | -5.6796 | -4.81654 | -4.81654 | 0 | 0 | -9.90018 | 0 |
| P19823\|ITIH2_HUMAN | -7.45235 | -4.07184 | -6.23645 | -6.23645 | -6.30327 | -7.18856 | -8.16385 | -7.18856 | 0 | -8.86608 | 0 | 0 |
| O1495\|ML12B_HUMAN | -3.05746 | -1.86832 | -1.96642 | -1.96642 | -4.61001 | -4.62926 | -5.87489 | -4.62926 | -6.10804 | -5.72182 | -6.74478 | -6.10804 |
| P24844\|MYL9_HUMAN | -3.05746 | -1.86832 | -1.96642 | -1.96642 | -4.61001 | -4.62926 | -5.87489 | -4.62926 | -6.10804 | -5.72182 | -6.74478 | -6.10804 |
| P1915\|ML12A_HUMAN | -3.05746 | -1.86832 | -1.96642 | -1.96642 | -4.61001 | -4.62926 | -5.87489 | -4.62926 | -6.10804 | -5.72182 | -6.74478 | -6.10804 |
| Q16881\|TRXR1_HUMAN | 0 | -10.9493 | 0 | 0 | 0 | 0 | 0 | 0 | 0 | 0 | 0 | 0 |
| P4349\|NAMPT_HUMAN | -5.51221 | -4.50692 | -5.93448 | -5.51221 | -5.27595 | -6.15054 | -6.48729 | -6.15054 | -7.92611 | -7.13357 | -7.40116 | -7.40116 |
| P17655\|CAN2_HUMAN | -9.25728 | -8.74247 | -9.51205 | -9.25728 | 0 | 0 | 0 | 0 | 0 | 0 | 0 | 0 |
| Q61\|CLH1_HUMAN | -8.16434 | -8.48425 | -9.00269 | -8.48425 | -8.19973 | -8.59329 | -8.00081 | -8.19973 | 0 | 0 | 0 | 0 |
| P1718\|LV327_HUMAN | -6.70403 | -7.2274 | -7.29854 | -7.2274 | -6.19512 | -5.21828 | -6.30891 | -6.19512 | -9.25552 | 0 | -9.23482 | -9.23482 |
| Q9H299\|SH3L3_HUMAN | 0 | -9.38769 | -9.36021 | -9.36021 | 0 | 0 | 0 | 0 | 0 | 0 | 0 | 0 |
| Q72\|RL18_HUMAN | -7.60675 | -7.09284 | -7.83289 | -7.60675 | -7.97208 | 0 | 0 | 0 | 0 | -9.79105 | 0 | 0 |
| Q7Z4S6\|KI21A_HUMAN | -9.08624 | -10.7854 | -9.17258 | -9.17258 | 0 | 0 | 0 | 0 | 0 | 0 | 0 | 0 |
| O534\|VMA5A_HUMAN | -9.14063 | -9.43468 | -9.33981 | -9.33981 | 0 | -9.36521 | 0 | 0 | 0 | 0 | 0 | 0 |
| Q9666\|AHNK_HUMAN | -3.2151 | -1.80349 | -2.27808 | -2.27808 | -4.58707 | -4.19326 | -4.90474 | -4.58707 | -7.29073 | -6.71698 | -6.37502 | -6.71698 |
| Q8TD19\|NEK9_HUMAN | -4.52804 | -6.05329 | -4.47849 | -4.52804 | -4.22327 | -4.47713 | -4.03022 | -4.22327 | -8.03423 | -7.98919 | -7.24196 | -7.98919 |
| O15144\|ARPC2_HUMAN | -7.8342 | -6.89317 | -7.3732 | -7.3732 | -8.33126 | -8.37571 | 0 | -8.33126 | -10.0437 | 0 | -10.8061 | -10.0437 |
| Q8IZP2\|ST134_HUMAN | -7.85474 | -8.03868 | -8.17601 | -8.03868 | -9.34361 | 0 | 0 | 0 | 0 | 0 | 0 | 0 |
| P552\|F1A1_HUMAN | -7.85474 | -8.03868 | -8.17601 | -8.03868 | -9.34361 | 0 | 0 | 0 | 0 | 0 | 0 | 0 |
| Q8NFI4\|F1A5_HUMAN | -7.85474 | -8.03868 | -8.17601 | -8.03868 | -9.34361 | 0 | 0 | 0 | 0 | 0 | 0 | 0 |
| P55795\|HNRH2_HUMAN | -7.82857 | -7.21828 | -7.61934 | -7.61934 | -7.94485 | -7.56089 | -8.48022 | -7.94485 | 0 | -9.96437 | 0 | 0 |
| P31943\|HNRH1_HUMAN | -7.82857 | -7.21828 | -7.61934 | -7.61934 | -7.94485 | -7.56089 | -8.48022 | -7.94485 | 0 | -9.96437 | 0 | 0 |
| Q99933\|BAG1_HUMAN | -9.1472 | -8.43307 | -8.98506 | -8.98506 | -9.2348 | 0 | -8.73522 | -8.73522 | 0 | 0 | 0 | 0 |
| P21926\|CD9_HUMAN | -3.5809 | -2.68701 | -3.07806 | -3.07806 | -3.92461 | -3.76438 | -3.96877 | -3.92461 | -7.04465 | -6.96526 | -6.09777 | -6.96526 |
| P7195\|LDHB_HUMAN | -6.33834 | -5.83572 | -6.64737 | -6.33834 | -6.00691 | -6.12911 | -7.22926 | -6.12911 | 0 | 0 | 0 | 0 |
| P3566\|COPB2_HUMAN | -10.1502 | -9.90956 | -9.55147 | -9.90956 | 0 | 0 | 0 | 0 | 0 | 0 | 0 | 0 |
| P49354\|FNTA_HUMAN | -9.138 | -10.346 | 0 | -9.138 | 0 | 0 | 0 | 0 | 0 | 0 | 0 | 0 |
| Q99832\|TCPH_HUMAN | -10.3296 | -11.2777 | -10.8468 | -10.8468 | 0 | 0 | 0 | 0 | 0 | 0 | -10.6286 | 0 |
| P2866\|PSA5_HUMAN | -7.48498 | -6.449 | -7.31479 | -7.31479 | -7.91142 | -8.14335 | 0 | -7.91142 | -10.2423 | -10.0101 | 0 | -10.0101 |
| P11216\|PYGB_HUMAN | -8.83868 | -9.27503 | -9.6182 | -9.27503 | -8.4302 | -8.9452 | 0 | -8.4302 | 0 | 0 | 0 | 0 |
| Q9Y5Z4\|HEBP2_HUMAN | -5.8749 | -5.64322 | -5.48975 | -5.64322 | -5.33476 | -5.17922 | -5.34702 | -5.33476 | -6.82292 | -6.5192 | -6.62007 | -6.62007 |
| Q9H6S3\|ES8L2_HUMAN | -10.2778 | -10.0112 | -10.1152 | -10.1152 | 0 | 0 | 0 | 0 | 0 | 0 | 0 | 0 |
| P52788\|SPSY_HUMAN | -8.27299 | -7.92318 | -8.90503 | -8.27299 | 0 | 0 | 0 | 0 | 0 | 0 | 0 | 0 |
| Q721\|C1QBP_HUMAN | 0 | -9.88897 | -9.29221 | -9.29221 | 0 | 0 | 0 | 0 | 0 | 0 | 0 | 0 |
| Q96FW1\|OTUB1_HUMAN | -7.86318 | -8.27435 | -7.7344 | -7.86318 | -8.11962 | -7.64652 | -7.89561 | -7.89561 | 0 | 0 | 0 | 0 |
| O75882\|ATRN_HUMAN | -7.60258 | -8.71338 | -8.10541 | -8.10541 | -7.30199 | -7.45241 | -7.3885 | -7.3885 | -10.6984 | 0 | -10.2036 | -10.2036 |
| Q6PID8\|KLD1_HUMAN | 0 | 0 | -11.179 | 0 | -9.29516 | 0 | 0 | 0 | 0 | 0 | 0 | 0 |
| P326\|ADH1G_HUMAN | -7.5862 | -6.55747 | -6.51954 | -6.55747 | -6.81887 | -7.54267 | -8.47038 | -7.54267 | 0 | -8.30651 | -10.066 | -8.30651 |
| P7327\|ADH1A_HUMAN | -7.5862 | -6.55747 | -6.51954 | -6.55747 | -6.81887 | -7.54267 | -8.47038 | -7.54267 | 0 | -8.30651 | -10.066 | -8.30651 |
| Q9H2U2\|IPYR2_HUMAN | 0 | -9.34025 | -8.71921 | -8.71921 | 0 | 0 | 0 | 0 | 0 | -10.0845 | 0 | 0 |
| Q9NSB4\|KRT82_HUMAN | -2.84666 | -2.32532 | -1.93629 | -2.32532 | -4.74989 | -4.14644 | -3.71919 | -4.14644 | -7.09552 | -6.49781 | -4.80239 | -6.49781 |
| P6744\|G6PI_HUMAN | -6.29523 | -6.30097 | -5.53892 | -6.29523 | -8.86062 | -7.07822 | -7.85701 | -7.85701 | 0 | -10.0382 | 0 | 0 |
| Q6NT89\|TRNP1_HUMAN | -6.92222 | -7.57876 | -8.11673 | -7.57876 | -5.58083 | -4.35312 | -5.32206 | -5.32206 | -8.85774 | -8.93754 | -9.71577 | -8.93754 |
| Q1584\|PDIA6_HUMAN | 0 | -10.9075 | 0 | 0 | -9.50149 | 0 | 0 | 0 | 0 | 0 | 0 | 0 |
| Q8TD6\|AGR3_HUMAN | -8.41064 | -7.72697 | -7.08177 | -7.72697 | -8.93388 | -8.83464 | -9.01467 | -8.93388 | 0 | -9.11471 | 0 | 0 |
| Q9H7S9\|ZN73_HUMAN | 0 | -10.1954 | -9.51736 | -9.51736 | 0 | -9.05665 | -9.00203 | -9.00203 | 0 | 0 | 0 | 0 |
| Q53LP3\|SWAHC_HUMAN | -8.50092 | -11.6553 | 0 | -8.50092 | -7.13332 | 0 | 0 | 0 | 0 | 0 | -9.63845 | 0 |
| P25398\|RS12_HUMAN | -7.07011 | -6.09523 | -6.68999 | -6.68999 | -8.41054 | -8.50328 | 0 | -8.41054 | 0 | 0 | 0 | 0 |
| P48634\|PRC2A_HUMAN | -5.87039 | -8.80665 | -7.58223 | -7.58223 | -5.0684 | -5.32956 | -5.00316 | -5.0684 | -3.02619 | -2.55665 | -3.42022 | -3.02619 |
| Q9UL51\|HCN2_HUMAN | -6.16894 | -7.86866 | -6.43733 | -6.43733 | -6.76033 | -6.80701 | -7.4626 | -6.80701 | -8.77578 | -7.60261 | -9.55169 | -8.77578 |
| Q9HAP2\|MLXIP_HUMAN | -7.52349 | -9.75693 | -8.60504 | -8.60504 | -7.50093 | -8.08457 | -8.51442 | -8.08457 | 0 | -8.84287 | 0 | 0 |
| Q86UE8\|TLK2_HUMAN | 0 | -11.8627 | -10.5949 | -10.5949 | -9.27107 | -9.3949 | 0 | -9.27107 | 0 | 0 | 0 | 0 |
| Q9NY33\|DPP3_HUMAN | -9.43049 | -10.5638 | -9.8599 | -9.8599 | -8.90019 | 0 | 0 | 0 | 0 | 0 | 0 | 0 |
| Q96JH8\|RADIL_HUMAN | -8.00415 | -10.1566 | -8.29252 | -8.29252 | -6.88572 | -7.16942 | -7.75436 | -7.16942 | -10.5604 | 0 | 0 | 0 |
| P36955\|PEDF_HUMAN | -8.38993 | -8.98309 | -10.4109 | -8.98309 | 0 | -8.99724 | -8.97914 | -8.97914 | 0 | 0 | 0 | 0 |
| Q86UT5\|NHRF4_HUMAN | -6.18426 | -6.79452 | -6.65175 | -6.65175 | -7.39209 | -6.94934 | -7.26043 | -7.26043 | -9.04055 | -7.35717 | -7.92506 | -7.92506 |
| Q8NCB2\|CAMKV_HUMAN | -8.04579 | -9.11932 | -8.31641 | -8.31641 | -7.49716 | -7.84663 | -8.59462 | -7.84663 | -7.44889 | -7.04042 | -8.88391 | -7.44889 |
| Q6SPF\|SAMD1_HUMAN | -1.84577 | -5.22796 | -3.66738 | -3.66738 | -1.3496 | -2.20797 | -1.73376 | -1.73376 | -4.11382 | -3.45742 | -2.96477 | -3.45742 |
| Q86YR7\|MF2L2_HUMAN | -8.68825 | -11.9056 | -9.99132 | -9.99132 | 0 | -8.82329 | -8.64963 | -8.64963 | 0 | 0 | -10.7376 | 0 |
| Q9NRI5\|DISC1_HUMAN | -6.37876 | -5.78006 | -6.5248 | -6.37876 | -5.91887 | -6.24048 | -5.90516 | -5.91887 | -7.19988 | -6.29685 | -7.68885 | -7.19988 |
| P25774\|CATS_HUMAN | -7.10171 | -8.70754 | -7.83854 | -7.83854 | -6.39597 | -6.02152 | -5.38159 | -6.02152 | -10.0161 | -8.58724 | -10.2543 | -10.0161 |
| O6437\|PEPL_HUMAN | -7.36837 | -6.24087 | -7.20898 | -7.20898 | -6.1612 | -6.87971 | -7.39654 | -6.87971 | -9.57263 | 0 | 0 | 0 |
| A8MYP8\|ODF3B_HUMAN | -3.44462 | -5.14242 | -5.90944 | -5.14242 | -3.19635 | -2.57342 | -3.58409 | -3.19635 | -6.14903 | -5.91903 | -5.08884 | -5.91903 |
| Q6UB35\|C1TM_HUMAN | 0 | 0 | 0 | 0 | -9.08308 | -8.90068 | 0 | -8.90068 | 0 | 0 | 0 | 0 |
| AA286YF58\|TM271_HUMAN | -8.66401 | -12.1528 | -11.2439 | -11.2439 | 0 | 0 | 0 | 0 | -11.1014 | 0 | -9.80836 | -9.80836 |
| Q9BXJ3\|C1QT4_HUMAN | -8.17755 | -9.77683 | -8.76378 | -8.76378 | -8.29052 | -8.9845 | -7.24352 | -8.29052 | 0 | 0 | 0 | 0 |
| O716\|E2F3_HUMAN | -6.0106 | -8.42262 | -5.87539 | -6.0106 | -6.1956 | -6.72947 | -6.89643 | -6.72947 | -9.00105 | -9.48792 | -9.96089 | -9.48792 |
| Q6ZMY3\|SPOC1_HUMAN | -5.54326 | -6.55884 | -7.1965 | -6.55884 | -4.73456 | -4.77318 | -5.9757 | -4.77318 | -9.38916 | -8.53066 | -9.40092 | -9.38916 |
| PDOY3\|IGLC3_HUMAN | 0 | 0 | 0 | 0 | 0 | 0 | 0 | 0 | 0 | 0 | 0 | 0 |
| PDOY2\|IGLC2_HUMAN | 0 | 0 | 0 | 0 | 0 | 0 | 0 | 0 | 0 | 0 | 0 | 0 |
| P68371\|TBB4B_HUMAN | 0 | 0 | 0 | 0 | 0 | 0 | 0 | 0 | 0 | 0 | 0 | 0 |
| AM8Q6\|IGLC7_HUMAN | 0 | 0 | 0 | 0 | 0 | 0 | 0 | 0 | 0 | 0 | 0 | 0 |
| P8779\|K1C16_HUMAN | 0 | 0 | 0 | 0 | 0 | 0 | 0 | 0 | 0 | 0 | 0 | 0 |
| AAB4J1V\|HV315_HUMAN | 0 | 0 | 0 | 0 | 0 | 0 | 0 | 0 | 0 | 0 | 0 | 0 |
| Q1469\|FABP5_HUMAN | 0 | 0 | 0 | 0 | 0 | 0 | 0 | 0 | 0 | 0 | 0 | 0 |
| AAC4DH25\|KVD2_HUMAN | 0 | 0 | 0 | 0 | 0 | 0 | 0 | 0 | 0 | 0 | 0 | 0 |
| Q6S8J3\|POTEE_HUMAN | 0 | 0 | 0 | 0 | 0 | 0 | 0 | 0 | 0 | 0 | 0 | 0 |
| P747\|PLMN_HUMAN | 0 | 0 | 0 | 0 | 0 | 0 | 0 | 0 | 0 | 0 | 0 | 0 |
| P31947\|1433S_HUMAN | 0 | 0 | 0 | 0 | 0 | 0 | 0 | 0 | 0 | 0 | 0 | 0 |
| Q9UBG3\|CRNN_HUMAN | 0 | 0 | 0 | 0 | 0 | 0 | 0 | 0 | 0 | 0 | 0 | 0 |
| AAB4J1Y9\|HV372_HUMAN | 0 | 0 | 0 | 0 | 0 | 0 | 0 | 0 | 0 | 0 | 0 | 0 |
| P28325\|CYTD_HUMAN | 0 | 0 | 0 | 0 | 0 | 0 | 0 | 0 | 0 | 0 | 0 | 0 |
| Q9UBC9\|SPRR3_HUMAN | 0 | 0 | 0 | 0 | 0 | 0 | 0 | 0 | 0 | 0 | 0 | 0 |
| P61981\|1433G_HUMAN | 0 | 0 | 0 | 0 | 0 | 0 | 0 | 0 | 0 | 0 | 0 | 0 |
| AAAMRZ8\|KVD11_HUMAN | 0 | 0 | 0 | 0 | 0 | 0 | 0 | 0 | 0 | 0 | 0 | 0 |
| P4433\|KV311_HUMAN | 0 | 0 | 0 | 0 | 0 | 0 | 0 | 0 | 0 | 0 | 0 | 0 |
| P1743\|HV146_HUMAN | 0 | 0 | 0 | 0 | 0 | 0 | 0 | 0 | 0 | 0 | 0 | 0 |
| A2NJV5\|KV229_HUMAN | 0 | 0 | 0 | 0 | 0 | 0 | 0 | 0 | 0 | 0 | 0 | 0 |
| AA75B6S2\|KVD29_HUMAN | 0 | 0 | 0 | 0 | 0 | 0 | 0 | 0 | 0 | 0 | 0 | 0 |
| AAC4DH72\|KV16_HUMAN | 0 | 0 | 0 | 0 | 0 | 0 | 0 | 0 | 0 | 0 | 0 | 0 |
| Q6323\|PSME1_HUMAN | 0 | 0 | 0 | 0 | 0 | 0 | 0 | 0 | 0 | 0 | 0 | 0 |
| O15143\|ARC1B_HUMAN | 0 | 0 | 0 | 0 | 0 | 0 | 0 | 0 | 0 | 0 | 0 | 0 |
| AA75B6S9\|KV137_HUMAN | 0 | 0 | 0 | 0 | 0 | 0 | 0 | 0 | 0 | 0 | 0 | 0 |
| PDSN7\|KVD37_HUMAN | 0 | 0 | 0 | 0 | 0 | 0 | 0 | 0 | 0 | 0 | 0 | 0 |
| AAC4DH42\|HV366_HUMAN | 0 | 0 | 0 | 0 | 0 | 0 | 0 | 0 | 0 | 0 | 0 | 0 |
| AAC4DH34\|HV428_HUMAN | 0 | 0 | 0 | 0 | 0 | 0 | 0 | 0 | 0 | 0 | 0 | 0 |
| Q8431\|MFGM_HUMAN | 0 | 0 | 0 | 0 | 0 | 0 | 0 | 0 | 0 | 0 | 0 | 0 |
| O764\|PDXK_HUMAN | 0 | 0 | 0 | 0 | 0 | 0 | 0 | 0 | 0 | 0 | 0 | 0 |
| P441\|SODC_HUMAN | 0 | 0 | 0 | 0 | 0 | 0 | 0 | 0 | 0 | 0 | 0 | 0 |
| AAC4DH29\|HV13_HUMAN | 0 | 0 | 0 | 0 | 0 | 0 | 0 | 0 | 0 | 0 | 0 | 0 |
| Q15293\|RCN1_HUMAN | 0 | 0 | 0 | 0 | 0 | 0 | 0 | 0 | 0 | 0 | 0 | 0 |
| P5564\|AQP5_HUMAN | 0 | 0 | 0 | 0 | 0 | 0 | 0 | 0 | 0 | 0 | 0 | 0 |
| AA75B6I\|LV861_HUMAN | 0 | 0 | 0 | 0 | 0 | 0 | 0 | 0 | 0 | 0 | 0 | 0 |
| P14\|CYTA_HUMAN | 0 | 0 | 0 | 0 | 0 | 0 | 0 | 0 | 0 | 0 | 0 | 0 |
| P4179\|SODM_HUMAN | 0 | 0 | 0 | 0 | 0 | 0 | 0 | 0 | 0 | 0 | 0 | 0 |
| P15814\|IGLL1_HUMAN | 0 | 0 | 0 | 0 | 0 | 0 | 0 | 0 | 0 | 0 | 0 | 0 |
| P19961\|AMY2B_HUMAN | 0 | 0 | 0 | 0 | 0 | 0 | 0 | 0 | 0 | 0 | 0 | 0 |
| PDTE7\|AMY1B_HUMAN | 0 | 0 | 0 | 0 | 0 | 0 | 0 | 0 | 0 | 0 | 0 | 0 |
| P4746\|AMYP_HUMAN | 0 | 0 | 0 | 0 | 0 | 0 | 0 | 0 | 0 | 0 | 0 | 0 |
| P4745\|AMY1A_HUMAN | 0 | 0 | 0 | 0 | 0 | 0 | 0 | 0 | 0 | 0 | 0 | 0 |
| PDTE8\|AMY1C_HUMAN | 0 | 0 | 0 | 0 | 0 | 0 | 0 | 0 | 0 | 0 | 0 | 0 |
| Q796\|DHSO_HUMAN | 0 | 0 | 0 | 0 | 0 | 0 | 0 | 0 | 0 | 0 | 0 | 0 |
| O6664\|PLIN3_HUMAN | 0 | 0 | 0 | 0 | 0 | 0 | 0 | 0 | 0 | 0 | 0 | 0 |
| P1742\|HV169_HUMAN | 0 | 0 | 0 | 0 | 0 | 0 | 0 | 0 | 0 | 0 | 0 | 0 |
| AAB4J2H\|HV69D_HUMAN | 0 | 0 | 0 | 0 | 0 | 0 | 0 | 0 | 0 | 0 | 0 | 0 |
| PDOX3\|IGD_HUMAN | 0 | 0 | 0 | 0 | 0 | 0 | 0 | 0 | 0 | 0 | 0 | 0 |
| AA75B6I9\|LV746_HUMAN | 0 | 0 | 0 | 0 | 0 | 0 | 0 | 0 | 0 | 0 | 0 | 0 |
| P4211\|LV743_HUMAN | 0 | 0 | 0 | 0 | 0 | 0 | 0 | 0 | 0 | 0 | 0 | 0 |
| AA75B6H8\|KVD42_HUMAN | 0 | 0 | 0 | 0 | 0 | 0 | 0 | 0 | 0 | 0 | 0 | 0 |
| AA75B6J9\|LV218_HUMAN | 0 | 0 | 0 | 0 | 0 | 0 | 0 | 0 | 0 | 0 | 0 | 0 |
| AAB4J1U7\|HV61_HUMAN | 0 | 0 | 0 | 0 | 0 | 0 | 0 | 0 | 0 | 0 | 0 | 0 |
| P37837\|TALDO_HUMAN | 0 | 0 | 0 | 0 | 0 | 0 | 0 | 0 | 0 | 0 | 0 | 0 |
| AAAMT36\|KVD21_HUMAN | 0 | 0 | 0 | 0 | 0 | 0 | 0 | 0 | 0 | 0 | 0 | 0 |
| AAC4DH24\|KV621_HUMAN | 0 | 0 | 0 | 0 | 0 | 0 | 0 | 0 | 0 | 0 | 0 | 0 |
| P13639\|EF2_HUMAN | 0 | 0 | 0 | 0 | 0 | 0 | 0 | 0 | 0 | 0 | 0 | 0 |
| P171\|LV151_HUMAN | 0 | 0 | 0 | 0 | 0 | 0 | 0 | 0 | 0 | 0 | 0 | 0 |
| AA75B6I4\|LVX54_HUMAN | 0 | 0 | 0 | 0 | 0 | 0 | 0 | 0 | 0 | 0 | 0 | 0 |
| P1721\|LV657_HUMAN | 0 | 0 | 0 | 0 | 0 | 0 | 0 | 0 | 0 | 0 | 0 | 0 |
| Q9BUP\|EFHD1_HUMAN | 0 | 0 | 0 | 0 | 0 | 0 | 0 | 0 | 0 | 0 | 0 | 0 |
| Q96C19\|EFHD2_HUMAN | 0 | 0 | 0 | 0 | 0 | 0 | 0 | 0 | 0 | 0 | 0 | 0 |
| AA75B6Q5\|HV364_HUMAN | 0 | 0 | 0 | 0 | 0 | 0 | 0 | 0 | 0 | 0 | 0 | 0 |
| Q132\|PSMD2_HUMAN | 0 | 0 | 0 | 0 | 0 | 0 | 0 | 0 | 0 | 0 | 0 | 0 |
| Q9UKY7\|CDV3_HUMAN | 0 | 0 | 0 | 0 | 0 | 0 | 0 | 0 | 0 | 0 | 0 | 0 |
| Q99574\|NEUS_HUMAN | 0 | 0 | 0 | 0 | 0 | 0 | 0 | 0 | 0 | 0 | 0 | 0 |
| O391\|QSOX1_HUMAN | 0 | 0 | 0 | 0 | 0 | 0 | 0 | 0 | 0 | 0 | 0 | 0 |
| P173\|LV14_HUMAN | 0 | 0 | 0 | 0 | 0 | 0 | 0 | 0 | 0 | 0 | 0 | 0 |
| P23526\|SAHH_HUMAN | 0 | 0 | 0 | 0 | 0 | 0 | 0 | 0 | 0 | 0 | 0 | 0 |
| P25786\|PSA1_HUMAN | 0 | 0 | 0 | 0 | 0 | 0 | 0 | 0 | 0 | 0 | 0 | 0 |
| O43548\|TGM5_HUMAN | 0 | 0 | 0 | 0 | 0 | 0 | 0 | 0 | 0 | 0 | 0 | 0 |
| Q9Y6U3\|ADSV_HUMAN | 0 | 0 | 0 | 0 | 0 | 0 | 0 | 0 | 0 | 0 | 0 | 0 |
| Q9UBZ9\|REV1_HUMAN | 0 | 0 | 0 | 0 | 0 | 0 | 0 | 0 | 0 | 0 | 0 | 0 |
| Q8WXS5\|CCG8_HUMAN | 0 | 0 | 0 | 0 | 0 | 0 | 0 | 0 | 0 | 0 | 0 | 0 |
| O1499\|IPP2C_HUMAN | 0 | 0 | 0 | 0 | 0 | 0 | 0 | 0 | 0 | 0 | 0 | 0 |
| P3866\|VATA_HUMAN | 0 | 0 | 0 | 0 | 0 | 0 | 0 | 0 | 0 | 0 | 0 | 0 |
| Q15782\|CH3L2_HUMAN | 0 | 0 | 0 | 0 | 0 | 0 | 0 | 0 | 0 | 0 | 0 | 0 |
| P23284\|PPIB_HUMAN | 0 | 0 | 0 | 0 | 0 | 0 | 0 | 0 | 0 | 0 | 0 | 0 |
| Q7Z572\|SPT21_HUMAN | 0 | 0 | 0 | 0 | 0 | 0 | 0 | 0 | 0 | 0 | 0 | 0 |
| Q15365\|PCBP1_HUMAN | 0 | 0 | 0 | 0 | 0 | 0 | 0 | 0 | 0 | 0 | 0 | 0 |
| Q15366\|PCBP2_HUMAN | 0 | 0 | 0 | 0 | 0 | 0 | 0 | 0 | 0 | 0 | 0 | 0 |
| P57721\|PCBP3_HUMAN | 0 | 0 | 0 | 0 | 0 | 0 | 0 | 0 | 0 | 0 | 0 | 0 |
| P57723\|PCBP4_HUMAN | 0 | 0 | 0 | 0 | 0 | 0 | 0 | 0 | 0 | 0 | 0 | 0 |
| Q7Z614\|SNX2_HUMAN | 0 | 0 | 0 | 0 | 0 | 0 | 0 | 0 | 0 | 0 | 0 | 0 |
| A6NKD9\|CC85C_HUMAN | 0 | 0 | 0 | 0 | 0 | 0 | 0 | 0 | 0 | 0 | 0 | 0 |
| Q13315\|ATM_HUMAN | 0 | 0 | 0 | 0 | 0 | 0 | 0 | 0 | 0 | 0 | 0 | 0 |
| Q14624\|ITIH4_HUMAN | 0 | 0 | 0 | 0 | 0 | 0 | 0 | 0 | 0 | 0 | 0 | 0 |
| P46459\|NSF_HUMAN | 0 | 0 | 0 | 0 | 0 | 0 | 0 | 0 | 0 | 0 | 0 | 0 |
| Q9UBQ7\|GRHPR_HUMAN | 0 | 0 | 0 | 0 | 0 | 0 | 0 | 0 | 0 | 0 | 0 | 0 |
| P1514\|GLNA_HUMAN | 0 | 0 | 0 | 0 | 0 | 0 | 0 | 0 | 0 | 0 | 0 | 0 |
| Q9Y263\|PLAP_HUMAN | 0 | 0 | 0 | 0 | 0 | 0 | 0 | 0 | 0 | 0 | 0 | 0 |
| Q9NZJ7\|MTCH1_HUMAN | 0 | 0 | 0 | 0 | 0 | 0 | 0 | 0 | 0 | 0 | 0 | 0 |
| P23588\|IF4B_HUMAN | 0 | 0 | 0 | 0 | 0 | 0 | 0 | 0 | 0 | 0 | 0 | 0 |
| Q86UL8\|MAGI2_HUMAN | 0 | 0 | 0 | 0 | 0 | 0 | 0 | 0 | 0 | 0 | 0 | 0 |
| Q9Y6X\|SETBP_HUMAN | 0 | 0 | 0 | 0 | 0 | 0 | 0 | 0 | 0 | 0 | 0 | 0 |
| Q8WUM4\|PDC6I_HUMAN | 0 | 0 | 0 | 0 | 0 | 0 | 0 | 0 | 0 | 0 | 0 | 0 |
| P69\|PSA6_HUMAN | 0 | 0 | 0 | 0 | 0 | 0 | 0 | 0 | 0 | 0 | 0 | 0 |
| Q6ZS81\|WDFY4_HUMAN | 0 | 0 | 0 | 0 | 0 | 0 | 0 | 0 | 0 | 0 | 0 | 0 |
| Q96T58\|MINT_HUMAN | 0 | 0 | 0 | 0 | 0 | 0 | 0 | 0 | 0 | 0 | 0 | 0 |
| Q1368\|PEX6_HUMAN | 0 | 0 | 0 | 0 | 0 | 0 | 0 | 0 | 0 | 0 | 0 | 0 |
| Q9HBH5\|RDH14_HUMAN | 0 | 0 | 0 | 0 | 0 | 0 | 0 | 0 | 0 | 0 | 0 | 0 |
| O95969\|SG1D2_HUMAN | 0 | 0 | 0 | 0 | 0 | 0 | 0 | 0 | 0 | 0 | 0 | 0 |
| Q9NRU3\|CNNM1_HUMAN | 0 | 0 | 0 | 0 | 0 | 0 | 0 | 0 | 0 | 0 | 0 | 0 |
| Q96G74\|OTUD5_HUMAN | 0 | 0 | 0 | 0 | 0 | 0 | 0 | 0 | 0 | 0 | 0 | 0 |
| Q6ZN18\|AEBP2_HUMAN | 0 | 0 | 0 | 0 | 0 | 0 | 0 | 0 | 0 | 0 | 0 | 0 |
| P2654\|APOC1_HUMAN | 0 | 0 | 0 | 0 | 0 | 0 | 0 | 0 | 0 | 0 | 0 | 0 |
|  |  |  |  |  |  |  |  |  |  |  |  |  |
| **TMT10-plex 2 (TF6)** | |  |  |  |  |  |  |  |  |  |  |  |
| **Accession** | **1S** | **2S** | **6S** | **Median** | **1M** | **2M** | **6M** | **Median** | **1C** | **2C** | **6C** | **Median** |
| P2788\|TRFL_HUMAN | 6.24466 | 8.14396 | 7.46272 | 7.46272 | 7.17 | 7.42678 | 6.81994 | 7.17 | 4.1294 | 4.8205 | 4.57321 | 4.57321 |
| P2768\|ALBU_HUMAN | 2.90793 | 7.56477 | 7.43315 | 7.43315 | 2.83215 | 3.81787 | 2.54771 | 2.83215 | 0.49757 | 1.40152 | 2.07121 | 1.40152 |
| P3125\|LCN1_HUMAN | 7.47243 | 8.48939 | 8.26013 | 8.26013 | 7.95831 | 7.0291 | 6.96845 | 7.0291 | 4.28543 | 4.33222 | 4.70846 | 4.33222 |
| P9816\|PGBM_HUMAN | 0.58168 | 1.14945 | 0.38463 | 0.58168 | -0.17357 | 0.0626 | -0.71979 | -0.17357 | -2.50653 | -2.30683 | -2.87608 | -2.50653 |
| P1833\|PIGR_HUMAN | 1.80361 | 4.41414 | 4.14135 | 4.14135 | 2.30484 | 4.75032 | 3.8431 | 3.8431 | 0.81658 | 1.22627 | 0.74651 | 0.81658 |
| P124\|CO3_HUMAN | -0.61888 | 2.63066 | 1.09743 | 1.09743 | -0.90696 | 0.96695 | -0.51693 | -0.51693 | -1.1297 | -1.15827 | -1.0616 | -1.1297 |
| P1876\|IGHA1_HUMAN | 1.12668 | 4.05015 | 3.83789 | 3.83789 | 1.39773 | 4.13801 | 3.01712 | 3.01712 | 0.2759 | -0.00762 | 0.7341 | 0.2759 |
| P61626\|LYSC_HUMAN | 4.89589 | 5.70015 | 5.49998 | 5.49998 | 5.29631 | 4.76701 | 4.95398 | 4.95398 | 8.02403 | 7.83338 | 8.00868 | 8.00868 |
| P25311\|ZA2G_HUMAN | 1.94436 | 2.67214 | 2.68242 | 2.67214 | 2.22082 | 2.05958 | 2.23487 | 2.22082 | -0.69014 | 0.20721 | 0.06588 | 0.06588 |
| PDOX7\|IGK_HUMAN | 1.7052 | 4.62352 | 4.38178 | 4.38178 | 2.3638 | 4.93035 | 4.07742 | 4.07742 | 0.70116 | 0.91778 | 0.69237 | 0.70116 |
| P4264\|K2C1_HUMAN | -0.73945 | -0.44876 | -0.90433 | -0.73945 | -1.02342 | 0.97346 | -0.39767 | -0.39767 | -0.4499 | -1.07134 | 2.42581 | -0.4499 |
| Q5VSP4\|LC1L1_HUMAN | -4.62679 | -6.33369 | -6.87094 | -6.33369 | -6.24707 | -7.32288 | -7.55841 | -7.32288 | -9.63641 | -9.13944 | -9.38046 | -9.38046 |
| P2787\|TRFE_HUMAN | -0.77406 | 2.79568 | 1.96329 | 1.96329 | -1.14743 | -0.46486 | -1.41664 | -1.14743 | -2.32285 | -2.48373 | -1.66485 | -2.32285 |
| P14618\|KPYM_HUMAN | -1.59693 | 0.15605 | 1.26086 | 0.15605 | -4.06144 | -2.68222 | -3.24493 | -3.24493 | -6.12643 | -5.66748 | -5.35627 | -5.66748 |
| PDOX2\|IGA2_HUMAN | -2.60229 | -0.02455 | -0.31509 | -0.31509 | -2.75457 | 0.41489 | -0.53234 | -0.53234 | -4.09932 | -3.71334 | -3.72081 | -3.72081 |
| P6733\|ENOA_HUMAN | 0.10252 | 1.62748 | 3.48155 | 1.62748 | -1.4755 | 0.06325 | 0.33002 | 0.06325 | -3.19468 | -2.31074 | -2.41742 | -2.41742 |
| P1913\|K2C4_HUMAN | -0.54103 | 1.57449 | 2.14723 | 1.57449 | -1.93647 | -1.52745 | 1.18632 | -1.52745 | -2.6081 | -2.62704 | -0.4988 | -2.6081 |
| P136\|CYTS_HUMAN | 2.3741 | 2.96236 | 2.29193 | 2.3741 | 2.96557 | 2.30618 | 1.89691 | 2.30618 | -1.52927 | -0.92198 | -0.09149 | -0.92198 |
| P2198\|TGM2_HUMAN | -0.96473 | 1.70381 | 2.68242 | 1.70381 | -2.60481 | -1.71103 | -3.12824 | -2.60481 | -6.12761 | -6.256 | -4.50047 | -6.12761 |
| Q9UGM3\|DMBT1_HUMAN | -0.41584 | 0.78317 | -0.16476 | -0.16476 | -0.63072 | 0.18993 | -0.62757 | -0.62757 | -4.47208 | -3.3678 | -4.01894 | -4.01894 |
| P137\|CYTN_HUMAN | 0.30952 | 2.04862 | 0.68841 | 0.68841 | 0.31199 | 1.31344 | 0.02622 | 0.31199 | -2.19022 | -0.88006 | -1.45539 | -1.45539 |
| P6396\|GELS_HUMAN | -0.86911 | 0.75701 | 1.50608 | 0.75701 | -2.44904 | -1.08332 | -1.77673 | -1.77673 | -4.91483 | -3.69051 | -3.85234 | -3.85234 |
| P13647\|K2C5_HUMAN | -1.36547 | 0.13078 | 0.10879 | 0.10879 | -2.12975 | -1.67185 | 1.37786 | -1.67185 | -3.04148 | -2.09909 | -0.42976 | -2.09909 |
| P199\|CLUS_HUMAN | 1.00035 | 2.63039 | 2.68625 | 2.63039 | 1.20916 | 1.9006 | 2.57425 | 1.9006 | -0.39465 | 0.26107 | 0.55629 | 0.26107 |
| P13645\|K1C1_HUMAN | -1.57995 | -1.29032 | -1.69128 | -1.57995 | -3.459 | -1.91182 | -1.63491 | -1.91182 | -3.01462 | -3.02645 | 0.57517 | -3.01462 |
| P9888\|MUC5A_HUMAN | -0.97292 | -0.67748 | -0.46349 | -0.67748 | -3.69081 | -3.25401 | -4.05043 | -3.69081 | -4.87885 | -4.98413 | -5.20297 | -4.98413 |
| Q13421\|MSLN_HUMAN | 0.12433 | 1.5253 | 1.2252 | 1.2252 | 0.09627 | 1.44598 | 0.51285 | 0.51285 | -1.55747 | -1.20945 | -2.07624 | -1.55747 |
| P19\|A1AT_HUMAN | -1.94975 | 2.61207 | 1.16871 | 1.16871 | -1.78164 | -1.06041 | -2.93193 | -1.78164 | -4.4178 | -3.65548 | -3.35934 | -3.65548 |
| P679\|ACTB_HUMAN | -4.3761 | -2.27548 | -1.30255 | -2.27548 | -6.1282 | -4.71679 | -5.94916 | -5.94916 | -8.73346 | -9.82952 | -8.39617 | -8.73346 |
| P483\|ANXA1_HUMAN | -1.56902 | -0.0393 | 1.67065 | -0.0393 | -3.19776 | -2.19399 | -2.40775 | -2.40775 | -6.29789 | -5.39142 | -4.66893 | -5.39142 |
| P2647\|APOA1_HUMAN | -2.12468 | 1.25166 | 0.02378 | 0.02378 | -2.79357 | -2.66474 | -3.65909 | -2.79357 | -4.63022 | -4.50044 | -4.80013 | -4.63022 |
| P2538\|K2C6A_HUMAN | -5.35059 | -5.21094 | -6.58128 | -5.35059 | -6.71649 | -5.21098 | -2.79915 | -5.21098 | -6.64162 | -5.24073 | -4.11959 | -5.24073 |
| P35527\|K1C9_HUMAN | -2.75116 | -1.91829 | -3.51602 | -2.75116 | -2.97421 | 0.36704 | -3.22646 | -2.97421 | -1.96077 | -2.88478 | 0.51574 | -1.96077 |
| P8727\|K1C19_HUMAN | -1.51609 | 0.63841 | 1.15091 | 0.63841 | -2.93622 | -2.34287 | 0.05749 | -2.34287 | -4.94303 | -4.3416 | -2.15728 | -4.3416 |
| P738\|HPT_HUMAN | -1.91143 | 1.96409 | 0.96282 | 0.96282 | -2.12563 | -0.54637 | -0.68536 | -0.68536 | -4.08694 | -2.92423 | -3.09782 | -3.09782 |
| P7355\|ANXA2_HUMAN | -1.22235 | 0.28206 | 1.30562 | 0.28206 | -2.41498 | -2.25028 | -2.23893 | -2.25028 | -4.47809 | -3.86648 | -3.2202 | -3.86648 |
| P352\|AL1A1_HUMAN | -1.90599 | -0.14928 | 1.56506 | -0.14928 | -3.94557 | -2.71492 | -4.46962 | -3.94557 | -3.55091 | -4.90417 | -3.28598 | -3.55091 |
| Q16378\|PROL4_HUMAN | 4.21747 | 2.87359 | 2.53031 | 2.87359 | 4.87524 | 2.06268 | 2.26599 | 2.26599 | 3.02046 | 0.79716 | 1.25836 | 1.25836 |
| P3598\|K22E_HUMAN | -2.13909 | -2.21185 | -1.94996 | -2.13909 | -2.90905 | -1.33816 | -2.54562 | -2.54562 | -1.79776 | -2.77774 | 2.76545 | -1.79776 |
| PDOX5\|IGG1_HUMAN | -3.36569 | -0.36233 | -0.63457 | -0.63457 | -4.2282 | -3.1531 | -3.85769 | -3.85769 | -6.02066 | -6.31088 | -6.11862 | -6.11862 |
| P1871\|IGHM_HUMAN | -1.57743 | 1.46529 | -0.6684 | -0.6684 | -1.58645 | 1.72323 | -1.22854 | -1.22854 | -2.17971 | -2.25067 | -2.8071 | -2.25067 |
| P45\|CERU_HUMAN | -0.49129 | 1.41275 | 0.02605 | 0.02605 | -1.01488 | -0.38137 | -1.27625 | -1.01488 | -3.67662 | -3.64099 | -3.71147 | -3.67662 |
| Q9GZZ8\|LACRT_HUMAN | 5.3104 | 6.1787 | 6.43028 | 6.1787 | 6.86364 | 5.66562 | 6.37152 | 6.37152 | 3.58063 | 4.22919 | 4.04698 | 4.04698 |
| PDMV8\|HS71A_HUMAN | -2.45015 | -1.38045 | 0.18306 | -1.38045 | -4.54508 | -3.60784 | -3.59737 | -3.60784 | -4.26533 | -5.65329 | -3.74437 | -4.26533 |
| PDMV9\|HS71B_HUMAN | -2.45015 | -1.38045 | 0.18306 | -1.38045 | -4.54508 | -3.60784 | -3.59737 | -3.60784 | -4.26533 | -5.65329 | -3.74437 | -4.26533 |
| P13646\|K1C13_HUMAN | -2.44023 | -1.37351 | -0.99411 | -1.37351 | -4.76188 | -3.77357 | -0.49694 | -3.77357 | -4.48786 | -4.13523 | -2.2943 | -4.13523 |
| P9228\|CYTT_HUMAN | -3.22874 | -1.27789 | -4.46088 | -3.22874 | -4.81891 | -2.68062 | -5.60739 | -4.81891 | -7.87922 | -5.28464 | -5.72842 | -5.72842 |
| P672\|S1A9_HUMAN | 0.67048 | 2.00541 | 4.34475 | 2.00541 | -1.35404 | 0.50403 | 1.49161 | 0.50403 | -3.14633 | -1.80643 | -1.14046 | -1.80643 |
| P374\|ILEU_HUMAN | -1.04184 | 0.15483 | 1.67456 | 0.15483 | -3.33276 | -2.21634 | -2.7529 | -2.7529 | -6.72439 | -4.38615 | -4.51927 | -4.51927 |
| P6174\|TPIS_HUMAN | -1.95355 | -0.40811 | 1.10328 | -0.40811 | -4.09844 | -2.58898 | -3.20739 | -3.20739 | -6.66933 | -5.78333 | -5.14776 | -5.78333 |
| P8729\|K2C7_HUMAN | -3.03418 | -0.72894 | -0.72411 | -0.72894 | -4.11235 | -3.21245 | -2.47531 | -3.21245 | -5.80092 | -5.66575 | -4.83227 | -5.66575 |
| P12273\|PIP_HUMAN | 4.0675 | 5.7656 | 4.83594 | 4.83594 | 3.09244 | 3.64858 | 2.64418 | 3.09244 | -0.20201 | 0.88928 | 0.17632 | 0.17632 |
| P4259\|K2C6B_HUMAN | -5.36043 | -4.19975 | -3.13528 | -4.19975 | -7.6481 | -6.09079 | -6.67701 | -6.67701 | -8.03869 | -8.18454 | -8.39421 | -8.18454 |
| P1861\|IGHG4_HUMAN | -7.7577 | -5.5459 | -5.30366 | -5.5459 | -10.7668 | -8.58879 | -10.915 | -10.7668 | 0 | 0 | -10.6169 | 0 |
| P1859\|IGHG2_HUMAN | -3.84169 | -1.20647 | -1.84264 | -1.84264 | -6.24728 | -5.15539 | -6.19754 | -6.19754 | -7.22978 | -7.15535 | -6.81626 | -7.15535 |
| P6814\|EF1A1_HUMAN | -1.1216 | 0.86013 | 1.77754 | 0.86013 | -2.40846 | -1.58395 | -1.65524 | -1.65524 | -3.72612 | -3.60362 | -3.4598 | -3.60362 |
| P833\|NUCB2_HUMAN | -1.47955 | -2.24404 | -2.19248 | -2.19248 | -2.07465 | -3.11188 | -2.11414 | -2.11414 | -6.41938 | -5.61724 | -6.11881 | -6.11881 |
| P446\|G3P_HUMAN | -1.16589 | 0.86813 | 2.04529 | 0.86813 | -2.79536 | -1.37636 | -1.72902 | -1.72902 | -4.36304 | -2.91735 | -2.55448 | -2.91735 |
| P558\|PGK1_HUMAN | -3.59942 | -1.96942 | -0.81155 | -1.96942 | -4.90859 | -4.10232 | -4.42934 | -4.42934 | -5.19662 | -4.06785 | -4.79634 | -4.79634 |
| B9A64\|IGLL5_HUMAN | -2.69973 | -1.17541 | -1.28451 | -1.28451 | -2.73511 | -0.64383 | -1.31757 | -1.31757 | -4.5183 | -4.41431 | -5.41084 | -4.5183 |
| O75556\|SG2A1_HUMAN | 4.19349 | 4.40335 | 4.92708 | 4.40335 | 5.26892 | 3.61222 | 4.74079 | 4.74079 | 1.46763 | 1.69267 | 1.55156 | 1.55156 |
| P4792\|HSPB1_HUMAN | -1.84832 | -0.95113 | 0.77235 | -0.95113 | -4.23003 | -3.07865 | -2.16883 | -3.07865 | -6.09233 | -4.98168 | -4.49245 | -4.98168 |
| P123\|A2MG_HUMAN | -2.10746 | 0.81157 | -0.6125 | -0.6125 | -3.05329 | -2.60521 | -3.55218 | -3.05329 | -4.68654 | -4.34474 | -4.50089 | -4.50089 |
| P2533\|K1C14_HUMAN | -3.39417 | -3.04041 | -3.85582 | -3.39417 | -4.54618 | -2.87485 | -1.02634 | -2.87485 | -4.53424 | -3.70698 | -2.30475 | -3.70698 |
| P9211\|GSTP1_HUMAN | -1.62525 | 0.41295 | 1.72794 | 0.41295 | -3.27111 | -2.42275 | -3.50831 | -3.27111 | -5.65418 | -4.38309 | -4.62343 | -4.62343 |
| P344\|PRDX5_HUMAN | -3.6412 | -2.30882 | -0.40992 | -2.30882 | -6.17174 | -4.54442 | -5.65679 | -5.65679 | -10.2175 | -7.27349 | -8.53708 | -8.53708 |
| P386\|PEBP1_HUMAN | -3.57069 | -2.01508 | -0.35624 | -2.01508 | -5.5879 | -3.86144 | -4.22965 | -4.22965 | -6.07275 | -5.94201 | -4.864 | -5.94201 |
| P186\|IGHG3_HUMAN | 0 | 0 | 0 | 0 | 0 | 0 | 0 | 0 | 0 | 0 | 0 | 0 |
| P475\|ALDOA_HUMAN | -1.49669 | -0.15838 | 1.12945 | -0.15838 | -3.15332 | -2.44371 | -2.28086 | -2.44371 | -5.16963 | -4.83155 | -4.26913 | -4.83155 |
| P5787\|K2C8_HUMAN | -2.99204 | -1.48731 | -0.62393 | -1.48731 | -3.91206 | -3.30463 | -2.44123 | -3.30463 | -6.15168 | -5.6758 | -4.19813 | -5.6758 |
| P2675\|FIBB_HUMAN | -3.79081 | -0.68263 | -2.26714 | -2.26714 | -4.74573 | -4.40343 | -6.18122 | -4.74573 | -8.28701 | -7.27427 | -7.67821 | -7.67821 |
| P279\|HEMO_HUMAN | -3.34655 | -0.01569 | -0.79745 | -0.79745 | -4.40392 | -3.87682 | -5.29265 | -4.40392 | -7.73359 | -6.06518 | -6.67312 | -6.67312 |
| P2679\|FIBG_HUMAN | -2.22592 | 0.95529 | -0.56467 | -0.56467 | -2.37192 | -2.59863 | -3.29795 | -2.59863 | -3.05865 | -3.92131 | -2.80919 | -3.05865 |
| Q838\|LG3BP_HUMAN | -0.91874 | -0.21887 | -0.69243 | -0.69243 | -2.01751 | -1.16 | -1.41304 | -1.41304 | -4.2287 | -3.4874 | -3.47968 | -3.4874 |
| P11142\|HSP7C_HUMAN | -2.82935 | -1.78475 | -0.78098 | -1.78475 | -4.71454 | -3.80575 | -4.46182 | -4.46182 | -7.04328 | -6.31583 | -5.7886 | -6.31583 |
| P2774\|VTDB_HUMAN | -3.3578 | -0.12724 | -1.58965 | -1.58965 | -3.95961 | -2.64916 | -4.01273 | -3.95961 | -6.44017 | -5.66634 | -5.59897 | -5.66634 |
| O95968\|SG1D1_HUMAN | 2.19443 | 2.13075 | 2.51979 | 2.19443 | 2.22804 | 0.82902 | 1.81249 | 1.81249 | 0.09256 | -0.23199 | 0.43225 | 0.09256 |
| P79\|HS9A_HUMAN | -2.30744 | -1.18403 | -0.86122 | -1.18403 | -2.91175 | -1.30129 | -2.02489 | -2.02489 | -6.02532 | -4.67513 | -4.85702 | -4.85702 |
| P7858\|CATB_HUMAN | -1.88055 | -0.75478 | -1.47521 | -1.47521 | -3.08174 | -2.17361 | -2.45087 | -2.45087 | -5.70902 | -4.75335 | -4.77617 | -4.77617 |
| Q683\|PRDX1_HUMAN | -1.55149 | 0.31481 | 1.52313 | 0.31481 | -3.63208 | -2.40597 | -3.54402 | -3.54402 | -5.87591 | -5.18264 | -5.56485 | -5.56485 |
| Q8N474\|SFRP1_HUMAN | -0.8085 | 0.72893 | -0.72259 | -0.72259 | -3.10678 | -2.28365 | -2.62411 | -2.62411 | -3.90869 | -2.51595 | -2.87005 | -2.87005 |
| P762\|SAP_HUMAN | -1.16123 | -0.34128 | -1.05057 | -1.05057 | -1.31764 | -1.24066 | -0.97183 | -1.24066 | -2.97131 | -2.231 | -2.38893 | -2.38893 |
| P519\|S1A8_HUMAN | -1.6075 | 0.35029 | 1.90071 | 0.35029 | -3.19183 | -1.43116 | -1.96153 | -1.96153 | -4.72763 | -3.99811 | -3.07429 | -3.99811 |
| Q13228\|SBP1_HUMAN | -3.88387 | -1.86734 | -0.82492 | -1.86734 | -5.05052 | -3.86271 | -4.50578 | -4.50578 | -7.4393 | -5.90015 | -6.22347 | -6.22347 |
| Q7Z46\|MYH14_HUMAN | -3.45647 | -2.90245 | -1.99926 | -2.90245 | -4.91998 | -4.32426 | -4.61233 | -4.61233 | -6.31715 | -6.25903 | -5.60777 | -6.25903 |
| P31946\|1433B_HUMAN | -3.66742 | -2.14038 | -1.07041 | -2.14038 | -5.05007 | -3.76043 | -4.56435 | -4.56435 | -4.63081 | -4.99488 | -4.55001 | -4.63081 |
| P15311\|EZRI_HUMAN | -3.4196 | -2.5926 | -1.49236 | -2.5926 | -6.44198 | -4.9597 | -5.35598 | -5.35598 | -7.10007 | -7.05514 | -6.39112 | -7.05514 |
| P8188\|NGAL_HUMAN | -2.60889 | -0.87213 | -1.43472 | -1.43472 | -3.61237 | -0.96101 | -1.39581 | -1.39581 | -4.42751 | -4.25159 | -3.10235 | -4.25159 |
| P134\|CYTC_HUMAN | -1.26595 | 0.08733 | -0.64889 | -0.64889 | -1.99719 | -0.83227 | -1.47034 | -1.47034 | -3.0597 | -2.00808 | -1.98498 | -2.00808 |
| P6314\|1433Z_HUMAN | -3.29475 | -1.4916 | -0.21578 | -1.4916 | -5.23534 | -3.90453 | -4.44237 | -4.44237 | -7.63989 | -6.71492 | -6.14124 | -6.71492 |
| Q9BQE3\|TBA1C_HUMAN | -3.5909 | -1.6977 | -0.7053 | -1.6977 | -5.06163 | -4.05793 | -5.01743 | -5.01743 | -6.83834 | -6.31335 | -5.5954 | -6.31335 |
| Q71U36\|TBA1A_HUMAN | -3.5909 | -1.6977 | -0.7053 | -1.6977 | -5.06163 | -4.05793 | -5.01743 | -5.01743 | -6.83834 | -6.31335 | -5.5954 | -6.31335 |
| P68363\|TBA1B_HUMAN | -3.5909 | -1.6977 | -0.7053 | -1.6977 | -5.06163 | -4.05793 | -5.01743 | -5.01743 | -6.83834 | -6.31335 | -5.5954 | -6.31335 |
| P1643\|H12_HUMAN | -3.18589 | -1.40098 | -1.98876 | -1.98876 | -4.52035 | -4.86141 | -3.17596 | -4.52035 | -4.19674 | -3.92307 | -3.472 | -3.92307 |
| Q14515\|SPRL1_HUMAN | -3.04303 | -4.27117 | -3.03815 | -3.04303 | -3.80002 | -4.83073 | -2.72999 | -3.80002 | -8.25018 | -6.21912 | -6.97089 | -6.97089 |
| P966\|ASSY_HUMAN | -2.45793 | -1.24608 | -0.88453 | -1.24608 | -5.45179 | -4.94016 | -6.23221 | -5.45179 | -8.41465 | -7.24583 | -4.86317 | -7.24583 |
| Q99935\|PROL1_HUMAN | -0.94652 | -0.17249 | -1.03528 | -0.94652 | -1.61562 | -1.32757 | -1.87974 | -1.61562 | -2.99141 | -2.03419 | -1.36434 | -2.03419 |
| P8238\|HS9B_HUMAN | -9.56868 | -8.74178 | -9.15096 | -9.15096 | 0 | 0 | 0 | 0 | 0 | -11.6145 | 0 | 0 |
| P3782\|TAGL2_HUMAN | -3.16296 | -2.08908 | -0.6412 | -2.08908 | -5.17877 | -4.80932 | -4.21722 | -4.80932 | -8.90374 | -6.94723 | -6.6107 | -6.94723 |
| Q96DA\|ZG16B_HUMAN | -1.28702 | -1.38254 | -2.55917 | -1.38254 | -2.20263 | -2.02455 | -2.76903 | -2.20263 | -5.59027 | -5.0967 | -5.48715 | -5.48715 |
| P341\|PRDX6_HUMAN | -4.296 | -3.15814 | -1.95907 | -3.15814 | -5.67042 | -4.63371 | -5.47546 | -5.47546 | -7.43148 | -7.7616 | -6.43531 | -7.43148 |
| P1121\|BIP_HUMAN | -4.45383 | -4.03083 | -3.99093 | -4.03083 | -6.13042 | -6.03333 | -5.71856 | -6.03333 | -7.92781 | -7.32474 | -7.73196 | -7.73196 |
| P23528\|COF1_HUMAN | -2.55566 | -1.10879 | 0.1595 | -1.10879 | -5.41874 | -3.51051 | -3.57248 | -3.57248 | -4.77629 | -4.25461 | -4.24625 | -4.25461 |
| P2279\|PERL_HUMAN | -2.15523 | -3.59286 | -2.85507 | -2.85507 | -3.39148 | -4.53906 | -3.95264 | -3.95264 | -5.3473 | -5.34361 | -5.19317 | -5.34361 |
| P111\|AACT_HUMAN | -2.37523 | -2.21383 | -3.08591 | -2.37523 | -5.79095 | -4.75982 | -5.77415 | -5.77415 | -5.66386 | -5.55293 | -5.71769 | -5.66386 |
| P6727\|APOA4_HUMAN | -3.96536 | -2.09069 | -2.91968 | -2.91968 | -5.67047 | -4.97578 | -6.02766 | -5.67047 | -7.91452 | -8.05292 | -6.60844 | -7.91452 |
| P62937\|PPIA_HUMAN | -3.0663 | -2.49085 | -0.94376 | -2.49085 | -5.11551 | -4.05516 | -4.20182 | -4.20182 | -8.41523 | -6.64606 | -6.46905 | -6.64606 |
| P2763\|A1AG1_HUMAN | -4.06566 | -1.32967 | -2.35577 | -2.35577 | -6.60566 | -4.85307 | -5.69851 | -5.69851 | -7.91673 | -6.46649 | -6.3854 | -6.46649 |
| O43852\|CALU_HUMAN | -2.34051 | -2.34201 | -3.10408 | -2.34201 | -3.11895 | -3.10267 | -3.49735 | -3.11895 | -7.15174 | -5.92532 | -7.27905 | -7.15174 |
| Q6MZM9\|PRR27_HUMAN | -3.165 | -3.64707 | -3.18286 | -3.18286 | -4.4032 | -4.18414 | -4.00567 | -4.18414 | -6.3047 | -5.13233 | -4.70009 | -5.13233 |
| P2652\|APOA2_HUMAN | -4.76037 | -1.04729 | -2.2082 | -2.2082 | -5.63419 | -5.11609 | -6.71214 | -5.63419 | -7.74675 | -7.26422 | -7.27905 | -7.27905 |
| P6832\|ACTC_HUMAN | -5.0175 | -2.68544 | -1.52758 | -2.68544 | -7.72084 | -5.76176 | -6.56918 | -6.56918 | -11.6618 | -9.47786 | -8.27513 | -9.47786 |
| P62736\|ACTA_HUMAN | -5.0175 | -2.68544 | -1.52758 | -2.68544 | -7.72084 | -5.76176 | -6.56918 | -6.56918 | -11.6618 | -9.47786 | -8.27513 | -9.47786 |
| P63267\|ACTH_HUMAN | -5.0175 | -2.68544 | -1.52758 | -2.68544 | -7.72084 | -5.76176 | -6.56918 | -6.56918 | -11.6618 | -9.47786 | -8.27513 | -9.47786 |
| P68133\|ACTS_HUMAN | -5.0175 | -2.68544 | -1.52758 | -2.68544 | -7.72084 | -5.76176 | -6.56918 | -6.56918 | -11.6618 | -9.47786 | -8.27513 | -9.47786 |
| Q96KP4\|CNDP2_HUMAN | -5.93564 | -4.39054 | -2.68466 | -4.39054 | -8.47904 | -6.91615 | -8.60567 | -8.47904 | -11.6027 | -10.1349 | -11.1183 | -11.1183 |
| P3838\|AL3A1_HUMAN | -2.36058 | -1.62647 | 1.28866 | -1.62647 | -4.19654 | -2.53727 | -3.86129 | -3.86129 | -6.38951 | -5.77187 | -4.76145 | -5.77187 |
| P2671\|FIBA_HUMAN | -1.43463 | 0.94902 | -0.33201 | -0.33201 | -2.12684 | -2.18539 | -2.77987 | -2.18539 | -4.56853 | -3.79953 | -4.43747 | -4.43747 |
| P14555\|PA2GA_HUMAN | -2.55354 | -2.88328 | -2.99131 | -2.88328 | -4.91008 | -4.77904 | -4.69014 | -4.77904 | -0.1891 | -1.09899 | -1.37165 | -1.09899 |
| P32119\|PRDX2_HUMAN | -4.29008 | -2.968 | -1.63369 | -2.968 | -6.90305 | -5.60102 | -5.7878 | -5.7878 | -8.86247 | -7.33409 | -6.92957 | -7.33409 |
| P7737\|PROF1_HUMAN | -2.24512 | -0.33818 | 0.80468 | -0.33818 | -4.73966 | -3.33462 | -3.87113 | -3.87113 | -6.31525 | -5.68958 | -4.6973 | -5.68958 |
| P261\|TCO1_HUMAN | -0.8169 | 0.92615 | 0.00293 | 0.00293 | -1.80963 | 0.04143 | -0.8655 | -0.8655 | -4.29076 | -3.23485 | -3.53673 | -3.53673 |
| Q1518\|CAP1_HUMAN | -3.42106 | -2.30292 | -1.16402 | -2.30292 | -5.26385 | -4.89674 | -5.61281 | -5.26385 | -7.17666 | -7.92701 | -7.03474 | -7.17666 |
| P7437\|TBB5_HUMAN | 0 | 0 | 0 | 0 | 0 | 0 | 0 | 0 | 0 | 0 | 0 | 0 |
| P178\|HV37_HUMAN | -5.83922 | -3.97102 | -4.5089 | -4.5089 | -6.81498 | -3.72628 | -4.79948 | -4.79948 | -8.22305 | -7.22012 | -8.01919 | -8.01919 |
| P1591\|IGJ_HUMAN | -1.43932 | 0.83939 | 0.41222 | 0.41222 | -1.47341 | 1.57444 | 0.62129 | 0.62129 | -3.10019 | -2.43817 | -1.90312 | -2.43817 |
| P3973\|SLPI_HUMAN | -2.92941 | -2.608 | -3.95062 | -2.92941 | -4.75638 | -3.88418 | -4.40177 | -4.40177 | -2.04032 | -1.63974 | -1.83317 | -1.83317 |
| P35579\|MYH9_HUMAN | -9.42625 | -8.53075 | -7.63172 | -8.53075 | 0 | -11.8975 | 0 | 0 | 0 | 0 | 0 | 0 |
| P1619\|KV32_HUMAN | -3.81089 | -2.36504 | -2.59424 | -2.59424 | -4.23893 | -2.06945 | -2.75844 | -2.75844 | -5.03289 | -5.47688 | -5.50329 | -5.47688 |
| PDP24\|CALM2_HUMAN | -6.62177 | -5.60995 | -4.23581 | -5.60995 | -10.0001 | -8.16602 | -7.94812 | -8.16602 | 0 | -12.0602 | -11.9499 | -11.9499 |
| PDP23\|CALM1_HUMAN | -6.62177 | -5.60995 | -4.23581 | -5.60995 | -10.0001 | -8.16602 | -7.94812 | -8.16602 | 0 | -12.0602 | -11.9499 | -11.9499 |
| PDP25\|CALM3_HUMAN | -6.62177 | -5.60995 | -4.23581 | -5.60995 | -10.0001 | -8.16602 | -7.94812 | -8.16602 | 0 | -12.0602 | -11.9499 | -11.9499 |
| P385\|KCY_HUMAN | -4.04861 | -3.10289 | -1.69492 | -3.10289 | -6.62635 | -4.89816 | -6.07971 | -6.07971 | -8.12146 | -7.81467 | -7.99118 | -7.99118 |
| P61769\|B2MG_HUMAN | 0.31336 | 0.70755 | 0.83516 | 0.70755 | 0.13779 | 0.16275 | 0.17478 | 0.16275 | -2.54353 | -1.84693 | -1.21851 | -1.84693 |
| P666\|MYL6_HUMAN | -3.76724 | -2.68834 | -1.71619 | -2.68834 | -5.9159 | -5.33531 | -4.98363 | -5.33531 | -7.49066 | -6.37032 | -6.3258 | -6.37032 |
| Q9988\|H2B1L_HUMAN | -5.0287 | -3.3769 | -3.80395 | -3.80395 | -7.79164 | -6.42998 | -5.4413 | -6.42998 | -8.41939 | -8.58484 | -6.37629 | -8.41939 |
| Q99879\|H2B1M_HUMAN | -5.0287 | -3.3769 | -3.80395 | -3.80395 | -7.79164 | -6.42998 | -5.4413 | -6.42998 | -8.41939 | -8.58484 | -6.37629 | -8.41939 |
| Q9379\|H2B1H_HUMAN | -5.0287 | -3.3769 | -3.80395 | -3.80395 | -7.79164 | -6.42998 | -5.4413 | -6.42998 | -8.41939 | -8.58484 | -6.37629 | -8.41939 |
| Q5QNW6\|H2B2F_HUMAN | -5.0287 | -3.3769 | -3.80395 | -3.80395 | -7.79164 | -6.42998 | -5.4413 | -6.42998 | -8.41939 | -8.58484 | -6.37629 | -8.41939 |
| Q99877\|H2B1N_HUMAN | -5.0287 | -3.3769 | -3.80395 | -3.80395 | -7.79164 | -6.42998 | -5.4413 | -6.42998 | -8.41939 | -8.58484 | -6.37629 | -8.41939 |
| O6814\|H2B1K_HUMAN | -5.0287 | -3.3769 | -3.80395 | -3.80395 | -7.79164 | -6.42998 | -5.4413 | -6.42998 | -8.41939 | -8.58484 | -6.37629 | -8.41939 |
| P6287\|H2B1C_HUMAN | -5.0287 | -3.3769 | -3.80395 | -3.80395 | -7.79164 | -6.42998 | -5.4413 | -6.42998 | -8.41939 | -8.58484 | -6.37629 | -8.41939 |
| P58876\|H2B1D_HUMAN | -5.0287 | -3.3769 | -3.80395 | -3.80395 | -7.79164 | -6.42998 | -5.4413 | -6.42998 | -8.41939 | -8.58484 | -6.37629 | -8.41939 |
| P5753\|H2BFS_HUMAN | -5.0287 | -3.3769 | -3.80395 | -3.80395 | -7.79164 | -6.42998 | -5.4413 | -6.42998 | -8.41939 | -8.58484 | -6.37629 | -8.41939 |
| P63241\|IF5A1_HUMAN | -5.07546 | -4.23343 | -3.59853 | -4.23343 | -6.97359 | -6.31335 | -7.05571 | -6.97359 | -8.93928 | -8.73802 | -9.15672 | -8.93928 |
| Q4695\|K1C17_HUMAN | 0 | 0 | 0 | 0 | 0 | 0 | 0 | 0 | 0 | 0 | 0 | 0 |
| P18669\|PGAM1_HUMAN | -5.72722 | -4.58325 | -3.25283 | -4.58325 | -8.24378 | -6.38814 | -7.00918 | -7.00918 | -9.89338 | -9.53884 | -9.84107 | -9.84107 |
| Q9BRK5\|CAB45_HUMAN | -2.66605 | -2.78265 | -3.1004 | -2.78265 | -3.25413 | -3.23252 | -3.14013 | -3.23252 | -6.50038 | -5.91504 | -6.104 | -6.104 |
| P31949\|S1AB_HUMAN | -3.42995 | -2.20418 | -0.61481 | -2.20418 | -5.97079 | -4.9302 | -4.56476 | -4.9302 | -8.17653 | -8.06694 | -7.31162 | -8.06694 |
| O75874\|IDHC_HUMAN | -3.4457 | -2.31603 | -0.27509 | -2.31603 | -5.79561 | -4.77573 | -5.18988 | -5.18988 | -7.41736 | -7.12069 | -6.042 | -7.12069 |
| P68871\|HBB_HUMAN | -3.67679 | 1.04712 | -9.88334 | -3.67679 | -3.2067 | -5.94905 | -5.97819 | -5.94905 | -6.90658 | -7.97746 | -6.20279 | -6.90658 |
| P33778\|H2B1B_HUMAN | -7.17821 | -5.76788 | -6.19652 | -6.19652 | -9.88213 | -10.4749 | -8.00712 | -9.88213 | -11.7777 | -10.2937 | -9.02775 | -10.2937 |
| P6899\|H2B1J_HUMAN | -7.17821 | -5.76788 | -6.19652 | -6.19652 | -9.88213 | -10.4749 | -8.00712 | -9.88213 | -11.7777 | -10.2937 | -9.02775 | -10.2937 |
| P23527\|H2B1O_HUMAN | -7.17821 | -5.76788 | -6.19652 | -6.19652 | -9.88213 | -10.4749 | -8.00712 | -9.88213 | -11.7777 | -10.2937 | -9.02775 | -10.2937 |
| Q16778\|H2B2E_HUMAN | -7.17821 | -5.76788 | -6.19652 | -6.19652 | -9.88213 | -10.4749 | -8.00712 | -9.88213 | -11.7777 | -10.2937 | -9.02775 | -10.2937 |
| Q8N257\|H2B3B_HUMAN | -7.17821 | -5.76788 | -6.19652 | -6.19652 | -9.88213 | -10.4749 | -8.00712 | -9.88213 | -11.7777 | -10.2937 | -9.02775 | -10.2937 |
| P7384\|CAN1_HUMAN | -4.46641 | -3.54254 | -2.18495 | -3.54254 | -6.32947 | -5.78418 | -6.0997 | -6.0997 | -7.90899 | -7.22213 | -7.40628 | -7.40628 |
| Q2818\|NUCB1_HUMAN | -3.71784 | -4.04313 | -4.38032 | -4.04313 | -4.07764 | -4.97921 | -4.47633 | -4.47633 | -7.6871 | -7.09774 | -7.57934 | -7.57934 |
| Q99497\|PARK7_HUMAN | -3.50072 | -2.0597 | -0.87538 | -2.0597 | -6.65078 | -5.12692 | -5.2425 | -5.2425 | -10.0597 | -7.7134 | -7.07981 | -7.7134 |
| Q8NBJ4\|GOLM1_HUMAN | -1.98361 | -2.71707 | -2.56724 | -2.56724 | -2.08317 | -3.2036 | -2.06315 | -2.08317 | -5.51259 | -5.08922 | -5.19816 | -5.19816 |
| P5229\|6PGD_HUMAN | -4.03583 | -3.35432 | -2.07583 | -3.35432 | -4.62743 | -4.40079 | -4.56347 | -4.56347 | -7.7517 | -5.14077 | -5.98178 | -5.98178 |
| Q13217\|DNJC3_HUMAN | -2.10877 | -2.36433 | -2.97842 | -2.36433 | -2.93446 | -3.3635 | -3.21777 | -3.21777 | -6.12931 | -5.42613 | -6.05965 | -6.05965 |
| P48\|CYTB_HUMAN | -3.13833 | -1.17612 | -0.24378 | -1.17612 | -5.874 | -3.95831 | -2.80502 | -3.95831 | -7.67608 | -5.88058 | -5.22566 | -5.88058 |
| P4121\|CAPG_HUMAN | -3.25845 | -2.22896 | -0.86593 | -2.22896 | -6.06153 | -4.69721 | -4.86708 | -4.86708 | -7.44644 | -7.04362 | -6.47658 | -7.04362 |
| P7237\|PDIA1_HUMAN | -5.98782 | -6.29539 | -6.38883 | -6.29539 | -8.52938 | -8.53503 | -7.83889 | -8.52938 | -12.2926 | -10.1325 | -10.703 | -10.703 |
| P28799\|GRN_HUMAN | -3.82153 | -3.90707 | -4.47364 | -3.90707 | -4.98668 | -4.62252 | -4.91223 | -4.91223 | -7.24634 | -6.8287 | -6.26137 | -6.8287 |
| P17931\|LEG3_HUMAN | -2.77977 | -1.31876 | 0.22759 | -1.31876 | -5.26714 | -2.90992 | -3.30512 | -3.30512 | -6.22672 | -5.3226 | -4.63987 | -5.3226 |
| P6285\|H4_HUMAN | -3.58454 | -1.47196 | -2.006 | -2.006 | -5.75259 | -4.75229 | -3.51616 | -4.75229 | -7.39112 | -6.21623 | -4.98612 | -6.21623 |
| P36952\|SPB5_HUMAN | -4.38028 | -3.48675 | -2.68323 | -3.48675 | -7.77487 | -4.42111 | -5.05772 | -5.05772 | -7.90647 | -6.10703 | -6.47033 | -6.47033 |
| P13489\|RINI_HUMAN | 0 | -10.7596 | -8.7965 | -8.7965 | 0 | 0 | 0 | 0 | -11.9741 | 0 | 0 | 0 |
| P62258\|1433E_HUMAN | -3.91825 | -2.45254 | -1.14706 | -2.45254 | -6.40963 | -4.87002 | -6.29984 | -6.29984 | -6.02748 | -7.28619 | -5.44525 | -6.02748 |
| P59\|APOD_HUMAN | -4.14488 | -2.60979 | -4.55013 | -4.14488 | -4.08284 | -3.90256 | -4.87494 | -4.08284 | -6.36459 | -5.69674 | -2.95967 | -5.69674 |
| P18\|ANT3_HUMAN | -4.32122 | -2.33904 | -2.54105 | -2.54105 | -5.90026 | -5.42686 | -6.6041 | -5.90026 | -8.80274 | -7.69621 | -7.17472 | -7.69621 |
| P44\|VTNC_HUMAN | -4.86267 | -2.64065 | -4.19983 | -4.19983 | -6.5679 | -5.86013 | -6.86152 | -6.5679 | -9.59666 | -8.34131 | -8.11936 | -8.34131 |
| P5395\|GDIB_HUMAN | -6.57901 | -5.31142 | -4.05338 | -5.31142 | -10.1331 | -7.84797 | 0 | -7.84797 | 0 | 0 | 0 | 0 |
| P35241\|RADI_HUMAN | 0 | 0 | 0 | 0 | 0 | 0 | 0 | 0 | 0 | 0 | 0 | 0 |
| P2766\|TTHY_HUMAN | -2.23755 | -0.58038 | -1.26749 | -1.26749 | -3.06328 | -2.41836 | -3.18842 | -3.06328 | -6.48157 | -5.17192 | -5.26964 | -5.26964 |
| Q14764\|MVP_HUMAN | -5.08883 | -4.84986 | -4.48831 | -4.84986 | -6.95768 | -5.51472 | -5.97044 | -5.97044 | -8.89969 | -8.87621 | -8.48466 | -8.87621 |
| P8748\|LV321_HUMAN | -4.83813 | -3.25935 | -3.56138 | -3.56138 | -5.53856 | -2.85758 | -3.84141 | -3.84141 | -6.17859 | -5.64916 | -5.47617 | -5.64916 |
| P1782\|HV39_HUMAN | -2.98248 | -0.89685 | -1.21012 | -1.21012 | -4.05016 | -1.40248 | -2.07413 | -2.07413 | -5.22898 | -5.16881 | -5.3813 | -5.22898 |
| P8571\|CD14_HUMAN | -6.29081 | -5.76261 | -6.97803 | -6.29081 | -7.32115 | -7.00807 | -7.51076 | -7.32115 | -9.24927 | -8.95027 | -8.8296 | -8.95027 |
| P4217\|A1BG_HUMAN | -4.68517 | -2.25695 | -3.31489 | -3.31489 | -6.16953 | -5.31626 | -6.79583 | -6.16953 | -8.09255 | -8.46042 | -6.78151 | -8.09255 |
| P338\|LDHA_HUMAN | -1.87789 | -1.25189 | 2.20885 | -1.25189 | -3.74074 | -2.13636 | -3.18138 | -3.18138 | -5.01225 | -4.31929 | -3.77374 | -4.31929 |
| P2765\|FETUA_HUMAN | -5.32653 | -2.40962 | -3.79741 | -3.79741 | -6.84123 | -6.00863 | -8.55938 | -6.84123 | -11.5896 | -11.4648 | -8.91337 | -11.4648 |
| P1283\|CADH1_HUMAN | -4.99885 | -5.94926 | -6.45884 | -5.94926 | -7.12152 | -7.09693 | -6.71168 | -7.09693 | -5.25983 | -5.12254 | -5.13932 | -5.13932 |
| P5995\|ANX11_HUMAN | -3.84492 | -2.95901 | -1.37621 | -2.95901 | -5.82045 | -5.09585 | -5.06156 | -5.09585 | -6.47113 | -5.16229 | -5.53884 | -5.53884 |
| P22392\|NDKB_HUMAN | -2.47266 | -1.04477 | -0.2096 | -1.04477 | -3.61691 | -2.5605 | -3.64337 | -3.61691 | -5.83399 | -5.12275 | -5.11727 | -5.12275 |
| Q4828\|AK1C1_HUMAN | -4.8091 | -2.50547 | -2.27934 | -2.50547 | -6.79651 | -5.7416 | -6.91521 | -6.79651 | -7.83161 | -8.85342 | -7.07671 | -7.83161 |
| P27348\|1433T_HUMAN | 0 | 0 | 0 | 0 | 0 | 0 | 0 | 0 | 0 | 0 | 0 | 0 |
| P1851\|IL1RA_HUMAN | -3.8206 | -3.90205 | -2.35785 | -3.8206 | -5.96907 | -5.2395 | -5.53365 | -5.53365 | -7.44573 | -6.46346 | -6.92484 | -6.92484 |
| P2638\|MOES_HUMAN | 0 | 0 | 0 | 0 | 0 | 0 | 0 | 0 | 0 | 0 | 0 | 0 |
| P631\|KV23_HUMAN | 0 | -6.79628 | -7.59521 | -6.79628 | -11.8108 | -6.8679 | -8.73719 | -8.73719 | 0 | -11.2136 | -11.4626 | -11.2136 |
| AA75B6S6\|KVD3_HUMAN | 0 | -6.79628 | -7.59521 | -6.79628 | -11.8108 | -6.8679 | -8.73719 | -8.73719 | 0 | -11.2136 | -11.4626 | -11.2136 |
| P2941\|TKT_HUMAN | -3.97946 | -3.72688 | -2.60513 | -3.72688 | -6.63631 | -4.89082 | -6.69183 | -6.63631 | -7.6306 | -7.76825 | -6.89582 | -7.6306 |
| O299\|CLIC1_HUMAN | -4.3407 | -3.39848 | -2.60842 | -3.39848 | -6.03111 | -5.75997 | -6.70035 | -6.03111 | -9.31933 | -8.47469 | -7.67247 | -8.47469 |
| P311\|PDIA3_HUMAN | -5.96201 | -5.09736 | -4.94943 | -5.09736 | -8.51373 | -8.26376 | -6.64233 | -8.26376 | -11.5443 | -10.1007 | -9.77467 | -10.1007 |
| P863\|CFAH_HUMAN | -6.35763 | -4.47845 | -6.04529 | -6.04529 | -9.33202 | -7.92314 | -9.46066 | -9.33202 | -12.0295 | 0 | -10.7991 | -10.7991 |
| AAC4DH38\|HV551_HUMAN | -6.83595 | -5.34899 | -5.89741 | -5.89741 | -7.97625 | -5.52414 | -6.4802 | -6.4802 | -9.19382 | -10.1597 | -9.19653 | -9.19653 |
| PCG47\|UBB_HUMAN | -5.66341 | -5.18717 | -4.29998 | -5.18717 | -9.01086 | -7.38725 | -6.59631 | -7.38725 | -10.5831 | -8.44317 | -7.60368 | -8.44317 |
| PCG48\|UBC_HUMAN | -5.66341 | -5.18717 | -4.29998 | -5.18717 | -9.01086 | -7.38725 | -6.59631 | -7.38725 | -10.5831 | -8.44317 | -7.60368 | -8.44317 |
| P62987\|RL4_HUMAN | -5.66341 | -5.18717 | -4.29998 | -5.18717 | -9.01086 | -7.38725 | -6.59631 | -7.38725 | -10.5831 | -8.44317 | -7.60368 | -8.44317 |
| P62979\|RS27A_HUMAN | -5.66341 | -5.18717 | -4.29998 | -5.18717 | -9.01086 | -7.38725 | -6.59631 | -7.38725 | -10.5831 | -8.44317 | -7.60368 | -8.44317 |
| P19971\|TYPH_HUMAN | 0 | 0 | 0 | 0 | 0 | 0 | 0 | 0 | 0 | 0 | 0 | 0 |
| P718\|ACBP_HUMAN | -3.17527 | -2.26546 | -0.6402 | -2.26546 | -5.70495 | -3.83689 | -3.41109 | -3.83689 | -7.91337 | -6.04324 | -5.782 | -6.04324 |
| P275\|A2GL_HUMAN | -5.35067 | -5.14956 | -6.03698 | -5.35067 | -7.39043 | -6.56265 | -5.67435 | -6.56265 | -8.3042 | -9.10087 | -9.24022 | -9.10087 |
| P1615\|KVD28_HUMAN | -9.01113 | -8.17112 | -8.72404 | -8.72404 | -10.6331 | -8.53151 | -9.50525 | -9.50525 | 0 | -12.0122 | 0 | 0 |
| AA75B6P5\|KV228_HUMAN | -9.01113 | -8.17112 | -8.72404 | -8.72404 | -10.6331 | -8.53151 | -9.50525 | -9.50525 | 0 | -12.0122 | 0 | 0 |
| P1593\|KVD33_HUMAN | 0 | -11.594 | -10.535 | -10.535 | 0 | 0 | 0 | 0 | -12.0596 | -11.7916 | 0 | -11.7916 |
| P1594\|KV133_HUMAN | 0 | -11.594 | -10.535 | -10.535 | 0 | 0 | 0 | 0 | -12.0596 | -11.7916 | 0 | -11.7916 |
| Q9Y49\|TLN1_HUMAN | -8.00803 | -10.0917 | -10.2681 | -10.0917 | 0 | -10.5912 | 0 | 0 | 0 | 0 | 0 | 0 |
| P395\|ANGI_HUMAN | -2.33636 | -2.64272 | -3.17528 | -2.64272 | -3.4614 | -3.54163 | -3.68744 | -3.54163 | -1.38851 | -1.08128 | -1.04215 | -1.08128 |
| Q96S96\|PEBP4_HUMAN | -7.30571 | -9.58889 | -9.67077 | -9.58889 | -9.13494 | -9.90965 | -9.18853 | -9.18853 | 0 | 0 | 0 | 0 |
| P47895\|AL1A3_HUMAN | -3.78072 | -3.13163 | -2.84753 | -3.13163 | -5.22906 | -4.50683 | -5.25144 | -5.22906 | -7.96705 | -7.38256 | -7.86362 | -7.86362 |
| AA75B6K4\|LV31_HUMAN | -6.29153 | -5.30529 | -5.20837 | -5.30529 | -7.63492 | -4.9837 | -5.67615 | -5.67615 | -10.1069 | -9.45923 | -8.87672 | -9.45923 |
| P1717\|LV325_HUMAN | -6.29153 | -5.30529 | -5.20837 | -5.30529 | -7.63492 | -4.9837 | -5.67615 | -5.67615 | -10.1069 | -9.45923 | -8.87672 | -9.45923 |
| P9467\|F16P1_HUMAN | -4.86121 | -4.84707 | -3.19513 | -4.84707 | -7.53411 | -7.19111 | -8.84936 | -7.53411 | -10.2411 | -10.1796 | -8.62538 | -10.1796 |
| P1641\|H15_HUMAN | -6.69706 | -5.21141 | -6.85144 | -6.69706 | -10.4666 | -9.08369 | -6.8287 | -9.08369 | -10.2972 | -8.92181 | -8.1 | -8.92181 |
| P751\|CFAB_HUMAN | -6.85688 | -5.45917 | -7.00092 | -6.85688 | -9.68502 | -8.31124 | -9.31511 | -9.31511 | 0 | -10.7282 | -11.4502 | -10.7282 |
| P17\|LV147_HUMAN | -3.88999 | -2.96709 | -3.43619 | -3.43619 | -4.4132 | -2.46006 | -3.47052 | -3.47052 | -5.97574 | -6.04523 | -5.98507 | -5.98507 |
| P39\|GSHR_HUMAN | -4.53507 | -3.6285 | -2.88516 | -3.6285 | -6.66088 | -5.80663 | -6.89561 | -6.66088 | -8.75448 | -6.35859 | -7.9462 | -7.9462 |
| P14625\|ENPL_HUMAN | -8.41214 | -7.94427 | -9.00506 | -8.41214 | -11.8587 | -11.0038 | -10.2511 | -11.0038 | 0 | 0 | 0 | 0 |
| P5572\|TERA_HUMAN | -4.05227 | -2.30827 | -1.70792 | -2.30827 | -5.55665 | -4.0108 | -5.1018 | -5.1018 | -7.12234 | -6.97857 | -6.27087 | -6.97857 |
| Q13162\|PRDX4_HUMAN | -7.30227 | -8.09031 | -7.87672 | -7.87672 | -10.3987 | -9.54427 | -9.67454 | -9.67454 | 0 | -11.0903 | 0 | 0 |
| P5558\|PLTP_HUMAN | -4.96636 | -5.46886 | -6.60713 | -5.46886 | -6.5353 | -7.07717 | -7.1349 | -7.07717 | -9.06984 | -9.19605 | -10.3561 | -9.19605 |
| P5155\|IC1_HUMAN | -5.5701 | -3.51886 | -4.19467 | -4.19467 | -6.71288 | -6.20539 | -3.91103 | -6.20539 | -9.94698 | -8.27123 | -7.54091 | -8.27123 |
| P4694\|IQGA1_HUMAN | -5.16755 | -6.76191 | -7.29271 | -6.76191 | -6.56952 | -7.68166 | -8.09198 | -7.68166 | -10.1465 | -11.3511 | -11.8801 | -11.3511 |
| P1825\|HV459_HUMAN | -5.24195 | -3.35407 | -4.14678 | -4.14678 | -6.19523 | -3.35696 | -4.42201 | -4.42201 | -5.58133 | -6.81862 | -5.59897 | -5.59897 |
| PDP8\|HVD82_HUMAN | -5.24195 | -3.35407 | -4.14678 | -4.14678 | -6.19523 | -3.35696 | -4.42201 | -4.42201 | -5.58133 | -6.81862 | -5.59897 | -5.59897 |
| PDP7\|HV431_HUMAN | -5.24195 | -3.35407 | -4.14678 | -4.14678 | -6.19523 | -3.35696 | -4.42201 | -4.42201 | -5.58133 | -6.81862 | -5.59897 | -5.59897 |
| AAC4DH41\|HV461_HUMAN | -5.24195 | -3.35407 | -4.14678 | -4.14678 | -6.19523 | -3.35696 | -4.42201 | -4.42201 | -5.58133 | -6.81862 | -5.59897 | -5.59897 |
| PDP6\|HVD34_HUMAN | -5.24195 | -3.35407 | -4.14678 | -4.14678 | -6.19523 | -3.35696 | -4.42201 | -4.42201 | -5.58133 | -6.81862 | -5.59897 | -5.59897 |
| P1824\|HV439_HUMAN | -5.24195 | -3.35407 | -4.14678 | -4.14678 | -6.19523 | -3.35696 | -4.42201 | -4.42201 | -5.58133 | -6.81862 | -5.59897 | -5.59897 |
| P52566\|GDIR2_HUMAN | -6.85139 | -7.13639 | -5.77362 | -6.85139 | -11.5208 | -9.53711 | -10.3978 | -10.3978 | 0 | 0 | 0 | 0 |
| AAAMS15\|HV349_HUMAN | -7.53105 | -7.0454 | -8.0392 | -7.53105 | -9.65627 | -6.46921 | -8.12184 | -8.12184 | -9.94989 | -9.89491 | -8.82164 | -9.89491 |
| Q9HC38\|GLOD4_HUMAN | -4.36222 | -3.59183 | -2.37759 | -3.59183 | -6.43413 | -5.06156 | -6.0025 | -6.0025 | -7.93038 | -7.36748 | -7.75033 | -7.75033 |
| AAB4J2D9\|KVD13_HUMAN | -5.51044 | -5.15319 | -4.76754 | -5.15319 | -6.55851 | -4.86856 | -4.88772 | -4.88772 | -8.66659 | -8.38308 | -8.52353 | -8.52353 |
| PDP9\|KV113_HUMAN | -5.51044 | -5.15319 | -4.76754 | -5.15319 | -6.55851 | -4.86856 | -4.88772 | -4.88772 | -8.66659 | -8.38308 | -8.52353 | -8.52353 |
| O4377\|ACTN4_HUMAN | -5.56159 | -4.29067 | -3.0617 | -4.29067 | -7.29036 | -6.23596 | -7.3482 | -7.29036 | -9.35676 | -9.12567 | -8.39257 | -9.12567 |
| Q99538\|LGMN_HUMAN | -7.69725 | -9.09299 | -9.07786 | -9.07786 | 0 | -11.5221 | -10.5784 | -10.5784 | 0 | 0 | -11.7724 | 0 |
| P16152\|CBR1_HUMAN | -5.17064 | -4.5842 | -3.99049 | -4.5842 | -6.86293 | -6.21403 | -6.82227 | -6.82227 | -9.64745 | -8.36287 | -8.72621 | -8.72621 |
| P31151\|S1A7_HUMAN | 0 | 0 | 0 | 0 | 0 | 0 | 0 | 0 | 0 | 0 | 0 | 0 |
| P26447\|S1A4_HUMAN | -1.90204 | 0.09207 | 1.79733 | 0.09207 | -4.78311 | -2.85171 | -2.87476 | -2.87476 | -7.77567 | -5.4683 | -5.52491 | -5.52491 |
| P6753\|TPM3_HUMAN | -5.17871 | -4.09543 | -3.31649 | -4.09543 | -7.46081 | -6.3803 | -6.00863 | -6.3803 | -8.47754 | -8.0514 | -8.04051 | -8.0514 |
| AAC4DH32\|HV32_HUMAN | 0 | 0 | 0 | 0 | 0 | 0 | 0 | 0 | 0 | 0 | 0 | 0 |
| P133\|TIMP1_HUMAN | -4.78765 | -3.76262 | -5.09031 | -4.78765 | -6.07313 | -4.51729 | -5.50383 | -5.50383 | -8.79903 | -7.11622 | -7.99052 | -7.99052 |
| P4925\|MDHC_HUMAN | -5.06781 | -4.0538 | -2.99739 | -4.0538 | -7.63332 | -6.4445 | -7.71055 | -7.63332 | 0 | -10.2775 | -10.2481 | -10.2481 |
| P2575\|ATPA_HUMAN | 0 | 0 | 0 | 0 | 0 | 0 | 0 | 0 | 0 | 0 | 0 | 0 |
| P22626\|ROA2_HUMAN | -5.22517 | -4.4126 | -3.79953 | -4.4126 | -7.43404 | -6.46769 | -5.85235 | -6.46769 | -9.58416 | -8.76076 | -7.46298 | -8.76076 |
| P1921\|AMD_HUMAN | -5.97703 | -6.72894 | -7.71323 | -6.72894 | -8.42935 | -8.1728 | -8.46569 | -8.42935 | 0 | -11.7745 | -10.6168 | -10.6168 |
| P2545\|LMNA_HUMAN | -3.2079 | -2.75536 | -3.13275 | -3.13275 | -3.94748 | -3.64665 | -3.3122 | -3.64665 | -6.05389 | -5.4552 | -5.82988 | -5.82988 |
| P8165\|DCD_HUMAN | -5.4007 | -7.8249 | -7.49861 | -7.49861 | -8.04275 | -8.16809 | -8.20721 | -8.16809 | -8.69525 | -10.4563 | -3.76683 | -8.69525 |
| P22314\|UBA1_HUMAN | -5.99564 | -5.04598 | -4.07689 | -5.04598 | -8.59164 | -7.93447 | -8.45241 | -8.45241 | -10.2488 | -9.80682 | -9.33211 | -9.80682 |
| P48643\|TCPE_HUMAN | 0 | -10.6458 | -9.17843 | -9.17843 | 0 | -12.11 | -11.9167 | -11.9167 | 0 | 0 | 0 | 0 |
| P1624\|KV315_HUMAN | -4.08893 | -2.44951 | -2.58906 | -2.58906 | -4.01644 | -1.8353 | -2.47985 | -2.47985 | -7.05957 | -5.59397 | -5.96355 | -5.96355 |
| Q8TCD5\|NT5C_HUMAN | 0 | -9.76802 | -8.2216 | -8.2216 | 0 | 0 | 0 | 0 | -12.0691 | 0 | 0 | 0 |
| P3496\|RNAS4_HUMAN | -7.00098 | -8.38366 | -8.96134 | -8.38366 | -9.74044 | -9.40304 | -9.54218 | -9.54218 | -6.32999 | -5.95617 | -5.60782 | -5.95617 |
| P6312\|KV41_HUMAN | -4.5089 | -2.82002 | -3.31208 | -3.31208 | -4.68034 | -2.0359 | -2.97892 | -2.97892 | -6.5166 | -6.63764 | -6.68545 | -6.63764 |
| Q14118\|DAG1_HUMAN | 0 | 0 | 0 | 0 | 0 | 0 | 0 | 0 | 0 | 0 | 0 | 0 |
| O151\|PDLI1_HUMAN | -8.33553 | -8.41395 | -7.02325 | -8.33553 | 0 | -11.5298 | -11.6249 | -11.5298 | -10.7351 | -12.0496 | 0 | -10.7351 |
| P5297\|CAZA1_HUMAN | 0 | 0 | 0 | 0 | 0 | 0 | 0 | 0 | 0 | 0 | 0 | 0 |
| AAC4DH69\|KV19_HUMAN | -7.94777 | -5.87066 | -8.40475 | -7.94777 | -8.56569 | -5.55497 | -9.10896 | -8.56569 | -8.86117 | -9.50588 | -11.7326 | -9.50588 |
| P9651\|ROA1_HUMAN | -6.9596 | -5.86682 | -5.68757 | -5.86682 | -9.01355 | -8.58851 | -8.04406 | -8.58851 | -10.4436 | -10.2079 | -9.64819 | -10.2079 |
| Q32P51\|RA1L2_HUMAN | -6.9596 | -5.86682 | -5.68757 | -5.86682 | -9.01355 | -8.58851 | -8.04406 | -8.58851 | -10.4436 | -10.2079 | -9.64819 | -10.2079 |
| Q99878\|H2A1J_HUMAN | -4.82399 | -2.66837 | -3.35081 | -3.35081 | -7.82473 | -5.82819 | -4.59052 | -5.82819 | -7.08557 | -7.38201 | -5.55312 | -7.08557 |
| Q96KK5\|H2A1H_HUMAN | -4.82399 | -2.66837 | -3.35081 | -3.35081 | -7.82473 | -5.82819 | -4.59052 | -5.82819 | -7.08557 | -7.38201 | -5.55312 | -7.08557 |
| Q16777\|H2A2C_HUMAN | -4.82399 | -2.66837 | -3.35081 | -3.35081 | -7.82473 | -5.82819 | -4.59052 | -5.82819 | -7.08557 | -7.38201 | -5.55312 | -7.08557 |
| Q9BTM1\|H2AJ_HUMAN | -4.82399 | -2.66837 | -3.35081 | -3.35081 | -7.82473 | -5.82819 | -4.59052 | -5.82819 | -7.08557 | -7.38201 | -5.55312 | -7.08557 |
| P498\|H2A1B_HUMAN | -4.82399 | -2.66837 | -3.35081 | -3.35081 | -7.82473 | -5.82819 | -4.59052 | -5.82819 | -7.08557 | -7.38201 | -5.55312 | -7.08557 |
| Q6FI13\|H2A2A_HUMAN | -4.82399 | -2.66837 | -3.35081 | -3.35081 | -7.82473 | -5.82819 | -4.59052 | -5.82819 | -7.08557 | -7.38201 | -5.55312 | -7.08557 |
| Q7L7L\|H2A3_HUMAN | -4.82399 | -2.66837 | -3.35081 | -3.35081 | -7.82473 | -5.82819 | -4.59052 | -5.82819 | -7.08557 | -7.38201 | -5.55312 | -7.08557 |
| Q9377\|H2A1C_HUMAN | -4.82399 | -2.66837 | -3.35081 | -3.35081 | -7.82473 | -5.82819 | -4.59052 | -5.82819 | -7.08557 | -7.38201 | -5.55312 | -7.08557 |
| PCS8\|H2A1_HUMAN | -4.82399 | -2.66837 | -3.35081 | -3.35081 | -7.82473 | -5.82819 | -4.59052 | -5.82819 | -7.08557 | -7.38201 | -5.55312 | -7.08557 |
| P2671\|H2A1D_HUMAN | -4.82399 | -2.66837 | -3.35081 | -3.35081 | -7.82473 | -5.82819 | -4.59052 | -5.82819 | -7.08557 | -7.38201 | -5.55312 | -7.08557 |
| Q8IUE6\|H2A2B_HUMAN | -4.82399 | -2.66837 | -3.35081 | -3.35081 | -7.82473 | -5.82819 | -4.59052 | -5.82819 | -7.08557 | -7.38201 | -5.55312 | -7.08557 |
| P1614\|H2AX_HUMAN | -4.82399 | -2.66837 | -3.35081 | -3.35081 | -7.82473 | -5.82819 | -4.59052 | -5.82819 | -7.08557 | -7.38201 | -5.55312 | -7.08557 |
| Q96QV6\|H2A1A_HUMAN | -4.82399 | -2.66837 | -3.35081 | -3.35081 | -7.82473 | -5.82819 | -4.59052 | -5.82819 | -7.08557 | -7.38201 | -5.55312 | -7.08557 |
| Q92743\|HTRA1_HUMAN | -5.48934 | -5.18055 | -6.54448 | -5.48934 | -7.22714 | -6.00809 | -6.8953 | -6.8953 | -9.77073 | -8.69095 | -9.75795 | -9.75795 |
| AA75B6R9\|KVD24_HUMAN | -9.51089 | -7.07055 | -9.02782 | -9.02782 | -10.029 | -6.56282 | -9.32628 | -9.32628 | -9.82716 | -11.4489 | 0 | -9.82716 |
| AAC4DH68\|KV224_HUMAN | -9.51089 | -7.07055 | -9.02782 | -9.02782 | -10.029 | -6.56282 | -9.32628 | -9.32628 | -9.82716 | -11.4489 | 0 | -9.82716 |
| P52565\|GDIR1_HUMAN | -4.38301 | -3.74595 | -2.17398 | -3.74595 | -7.09449 | -5.8429 | -5.63834 | -5.8429 | -5.98813 | -6.01925 | -6.67023 | -6.01925 |
| P49189\|AL9A1_HUMAN | 0 | 0 | 0 | 0 | 0 | 0 | 0 | 0 | 0 | 0 | 0 | 0 |
| P1714\|LV319_HUMAN | -5.92933 | -5.93224 | -6.03296 | -5.93224 | -7.66317 | -5.73451 | -5.7258 | -5.73451 | -7.96172 | -9.13111 | -9.34707 | -9.13111 |
| P7339\|CATD_HUMAN | -5.73314 | -6.08625 | -6.1483 | -6.08625 | -8.19863 | -7.08442 | -6.66906 | -7.08442 | -9.0294 | -7.01925 | -6.88841 | -7.01925 |
| Q15181\|IPYR_HUMAN | -4.11253 | -4.50742 | -4.09304 | -4.11253 | -4.95371 | -4.97066 | -5.41934 | -4.97066 | -9.47394 | -8.25951 | -8.89877 | -8.89877 |
| Q8WVQ1\|CANT1_HUMAN | 0 | 0 | 0 | 0 | 0 | 0 | 0 | 0 | 0 | 0 | 0 | 0 |
| P142\|KNG1_HUMAN | -6.9571 | -4.70138 | -6.03043 | -6.03043 | -8.50804 | -7.83055 | -8.69047 | -8.50804 | 0 | -11.7204 | 0 | 0 |
| P6576\|ATPB_HUMAN | 0 | -9.18231 | -10.1359 | -9.18231 | 0 | -11.355 | -11.8297 | -11.355 | 0 | 0 | 0 | 0 |
| O7583\|WDR1_HUMAN | -5.41955 | -4.73408 | -3.33028 | -4.73408 | -8.08908 | -6.94872 | -7.77698 | -7.77698 | -11.0632 | -9.44625 | -9.93927 | -9.93927 |
| P1455\|AK1A1_HUMAN | -2.70756 | -2.23778 | -2.15144 | -2.23778 | -4.86977 | -4.36452 | -5.31973 | -4.86977 | -7.39376 | -7.36243 | -7.29441 | -7.36243 |
| P17987\|TCPA_HUMAN | -9.5783 | -11.4889 | -10.2747 | -10.2747 | 0 | 0 | 0 | 0 | 0 | 0 | 0 | 0 |
| P17858\|PFKAL_HUMAN | -7.64213 | -9.03613 | -10.4966 | -9.03613 | -10.2375 | -11.4312 | -10.4003 | -10.4003 | 0 | 0 | -9.70349 | 0 |
| P9429\|HMGB1_HUMAN | -4.53916 | -2.63559 | -1.73624 | -2.63559 | -6.57377 | -5.43744 | -3.87587 | -5.43744 | -8.09654 | -7.01382 | -5.53728 | -7.01382 |
| P4939\|ECHA_HUMAN | -7.72246 | -6.7846 | -6.36467 | -6.7846 | 0 | -10.8001 | -10.7154 | -10.7154 | 0 | 0 | 0 | 0 |
| P867\|VIME_HUMAN | 0 | 0 | 0 | 0 | 0 | 0 | 0 | 0 | 0 | 0 | 0 | 0 |
| P25815\|S1P_HUMAN | -5.3129 | -5.19385 | -4.33628 | -5.19385 | -8.32118 | -8.23451 | -7.87861 | -8.23451 | -11.1106 | -9.806 | -9.41472 | -9.806 |
| P167\|CD44_HUMAN | 0 | 0 | 0 | 0 | 0 | 0 | 0 | 0 | 0 | 0 | 0 | 0 |
| PCL4\|CO4A_HUMAN | 0 | -9.77174 | -11.5765 | -9.77174 | 0 | 0 | 0 | 0 | 0 | 0 | 0 | 0 |
| PCL5\|CO4B_HUMAN | 0 | -9.77174 | -11.5765 | -9.77174 | 0 | 0 | 0 | 0 | 0 | 0 | 0 | 0 |
| Q621\|GFPT1_HUMAN | 0 | -10.0247 | -8.76721 | -8.76721 | 0 | 0 | 0 | 0 | 0 | 0 | 0 | 0 |
| O9488\|GFPT2_HUMAN | 0 | -10.0247 | -8.76721 | -8.76721 | 0 | 0 | 0 | 0 | 0 | 0 | 0 | 0 |
| Q9UQ8\|PA2G4_HUMAN | 0 | -9.1692 | -9.6518 | -9.1692 | 0 | 0 | -12.0578 | 0 | 0 | 0 | 0 | 0 |
| Q99954\|SMR3A_HUMAN | -4.21791 | -6.1998 | -4.92934 | -4.92934 | -6.38601 | -7.31254 | -6.80733 | -6.80733 | -9.3344 | -7.98586 | -8.87651 | -8.87651 |
| P13797\|PLST_HUMAN | -7.87686 | -6.75836 | -5.25179 | -6.75836 | -11.5289 | -9.18221 | -9.65728 | -9.65728 | -10.5158 | -11.035 | -9.92746 | -10.5158 |
| P4926\|MDHM_HUMAN | -6.9482 | -4.71652 | -5.60198 | -5.60198 | -9.14851 | -8.86633 | -6.99337 | -8.86633 | 0 | -10.2861 | -9.69322 | -9.69322 |
| P19652\|A1AG2_HUMAN | 0 | 0 | 0 | 0 | 0 | 0 | 0 | 0 | 0 | 0 | 0 | 0 |
| P1611\|KVD12_HUMAN | -5.7715 | -3.46962 | -4.17747 | -4.17747 | -6.54783 | -3.69112 | -4.71057 | -4.71057 | -6.7418 | -7.12553 | -6.43288 | -6.7418 |
| AAC4DH73\|KV112_HUMAN | -5.7715 | -3.46962 | -4.17747 | -4.17747 | -6.54783 | -3.69112 | -4.71057 | -4.71057 | -6.7418 | -7.12553 | -6.43288 | -6.7418 |
| P62277\|RS13_HUMAN | -6.57108 | -5.1147 | -4.68784 | -5.1147 | -10.0699 | -8.99871 | -8.2153 | -8.99871 | -11.5587 | -10.0181 | -9.09468 | -10.0181 |
| Q14697\|GANAB_HUMAN | 0 | 0 | 0 | 0 | 0 | 0 | 0 | 0 | 0 | 0 | 0 | 0 |
| P13693\|TCTP_HUMAN | -5.28712 | -4.89412 | -3.68164 | -4.89412 | -9.65515 | -7.42172 | -7.60085 | -7.60085 | 0 | -10.5429 | -10.144 | -10.144 |
| O462\|MANBA_HUMAN | 0 | 0 | 0 | 0 | 0 | 0 | 0 | 0 | 0 | 0 | 0 | 0 |
| P27797\|CALR_HUMAN | -8.89348 | -8.41456 | -8.22257 | -8.41456 | -11.9054 | 0 | -10.3808 | -10.3808 | 0 | -11.7671 | 0 | 0 |
| P8582\|TRFM_HUMAN | -4.35354 | -4.14883 | -3.86779 | -4.14883 | -5.19887 | -5.46887 | -5.60289 | -5.46887 | -7.60682 | -6.66079 | -7.67427 | -7.60682 |
| Q7Z5P9\|MUC19_HUMAN | -5.36203 | -6.31687 | -6.53422 | -6.31687 | -7.20035 | -7.54546 | -7.13311 | -7.20035 | -9.56271 | -8.76539 | -10.6106 | -9.56271 |
| P2511\|CRYAB_HUMAN | -4.57605 | -4.03073 | -1.95754 | -4.03073 | -8.21659 | -6.07686 | -4.26728 | -6.07686 | -8.71005 | -7.52225 | -7.0208 | -7.52225 |
| P49788\|TIG1_HUMAN | -7.94379 | -5.94239 | -7.27052 | -7.27052 | -9.4263 | -6.81498 | -7.82244 | -7.82244 | -9.96974 | -9.1997 | -9.57774 | -9.57774 |
| P19827\|ITIH1_HUMAN | 0 | 0 | 0 | 0 | 0 | 0 | 0 | 0 | 0 | 0 | 0 | 0 |
| P8758\|ANXA5_HUMAN | 0 | 0 | 0 | 0 | 0 | 0 | 0 | 0 | 0 | 0 | 0 | 0 |
| Q9BS4\|LXN_HUMAN | 0 | 0 | 0 | 0 | 0 | 0 | 0 | 0 | 0 | 0 | 0 | 0 |
| Q9H8\|LHPP_HUMAN | 0 | 0 | 0 | 0 | 0 | 0 | 0 | 0 | 0 | 0 | 0 | 0 |
| AA87WSZ\|KVD8_HUMAN | -7.88049 | -9.88559 | -11.3539 | -9.88559 | -9.81939 | -10.0299 | -10.948 | -10.0299 | 0 | 0 | 0 | 0 |
| Q1556\|IF4H_HUMAN | 0 | -11.1514 | -8.88417 | -8.88417 | 0 | 0 | 0 | 0 | 0 | 0 | 0 | 0 |
| O95834\|EMAL2_HUMAN | 0 | 0 | 0 | 0 | 0 | 0 | 0 | 0 | 0 | 0 | 0 | 0 |
| P21964\|COMT_HUMAN | -5.87835 | -5.77874 | -4.94687 | -5.77874 | -7.71407 | -7.00191 | -7.73482 | -7.71407 | -9.05089 | -8.98697 | -8.36855 | -8.98697 |
| P9525\|ANXA4_HUMAN | 0 | 0 | 0 | 0 | 0 | 0 | 0 | 0 | 0 | 0 | 0 | 0 |
| P436\|PSB1_HUMAN | 0 | 0 | 0 | 0 | 0 | 0 | 0 | 0 | 0 | 0 | 0 | 0 |
| P25789\|PSA4_HUMAN | -7.43351 | -8.6045 | -8.24456 | -8.24456 | -9.8889 | -9.38617 | -9.72716 | -9.72716 | 0 | 0 | 0 | 0 |
| Q182\|SPTB2_HUMAN | 0 | 0 | 0 | 0 | 0 | 0 | 0 | 0 | 0 | 0 | 0 | 0 |
| P2814\|SMR3B_HUMAN | -2.80177 | -7.45707 | -7.2419 | -7.2419 | -9.44861 | -8.40919 | -9.27281 | -9.27281 | -9.76124 | -5.679 | -7.12093 | -7.12093 |
| P17812\|PYRG1_HUMAN | 0 | 0 | 0 | 0 | 0 | 0 | 0 | 0 | 0 | 0 | 0 | 0 |
| P19823\|ITIH2_HUMAN | -6.22214 | -2.91722 | -3.93193 | -3.93193 | -7.88806 | -7.08269 | -9.49884 | -7.88806 | -10.5842 | -10.76 | -9.65654 | -10.5842 |
| O1495\|ML12B_HUMAN | -5.29955 | -3.92946 | -2.72343 | -3.92946 | -7.99074 | -6.45645 | -7.17801 | -7.17801 | -11.1002 | -9.76124 | -9.88316 | -9.88316 |
| P24844\|MYL9_HUMAN | -5.29955 | -3.92946 | -2.72343 | -3.92946 | -7.99074 | -6.45645 | -7.17801 | -7.17801 | -11.1002 | -9.76124 | -9.88316 | -9.88316 |
| P1915\|ML12A_HUMAN | -5.29955 | -3.92946 | -2.72343 | -3.92946 | -7.99074 | -6.45645 | -7.17801 | -7.17801 | -11.1002 | -9.76124 | -9.88316 | -9.88316 |
| Q16881\|TRXR1_HUMAN | 0 | 0 | 0 | 0 | 0 | 0 | 0 | 0 | 0 | 0 | 0 | 0 |
| P4349\|NAMPT_HUMAN | 0 | 0 | 0 | 0 | 0 | 0 | 0 | 0 | 0 | 0 | 0 | 0 |
| P17655\|CAN2_HUMAN | 0 | 0 | 0 | 0 | 0 | 0 | 0 | 0 | 0 | 0 | 0 | 0 |
| Q61\|CLH1_HUMAN | 0 | 0 | 0 | 0 | 0 | 0 | 0 | 0 | 0 | 0 | 0 | 0 |
| P1718\|LV327_HUMAN | 0 | 0 | 0 | 0 | 0 | 0 | 0 | 0 | 0 | 0 | 0 | 0 |
| Q9H299\|SH3L3_HUMAN | 0 | 0 | 0 | 0 | 0 | 0 | 0 | 0 | 0 | 0 | 0 | 0 |
| Q72\|RL18_HUMAN | 0 | 0 | 0 | 0 | 0 | 0 | 0 | 0 | 0 | 0 | 0 | 0 |
| Q7Z4S6\|KI21A_HUMAN | 0 | 0 | 0 | 0 | 0 | 0 | 0 | 0 | 0 | 0 | 0 | 0 |
| O534\|VMA5A_HUMAN | 0 | 0 | 0 | 0 | 0 | 0 | 0 | 0 | 0 | 0 | 0 | 0 |
| Q9666\|AHNK_HUMAN | -4.91457 | -4.46791 | -2.69116 | -4.46791 | -7.75136 | -6.21843 | -4.43193 | -6.21843 | -7.64955 | -7.36189 | -5.4732 | -7.36189 |
| Q8TD19\|NEK9_HUMAN | 0 | 0 | 0 | 0 | 0 | 0 | 0 | 0 | 0 | 0 | 0 | 0 |
| O15144\|ARPC2_HUMAN | 0 | 0 | 0 | 0 | 0 | 0 | 0 | 0 | 0 | 0 | 0 | 0 |
| Q8IZP2\|ST134_HUMAN | 0 | 0 | 0 | 0 | 0 | 0 | 0 | 0 | 0 | 0 | 0 | 0 |
| P552\|F1A1_HUMAN | 0 | 0 | 0 | 0 | 0 | 0 | 0 | 0 | 0 | 0 | 0 | 0 |
| Q8NFI4\|F1A5_HUMAN | 0 | 0 | 0 | 0 | 0 | 0 | 0 | 0 | 0 | 0 | 0 | 0 |
| P55795\|HNRH2_HUMAN | 0 | 0 | 0 | 0 | 0 | 0 | 0 | 0 | 0 | 0 | 0 | 0 |
| P31943\|HNRH1_HUMAN | 0 | 0 | 0 | 0 | 0 | 0 | 0 | 0 | 0 | 0 | 0 | 0 |
| Q99933\|BAG1_HUMAN | -9.65813 | -9.63795 | -7.9888 | -9.63795 | 0 | -11.5139 | -10.074 | -10.074 | 0 | 0 | 0 | 0 |
| P21926\|CD9_HUMAN | -6.13345 | -5.976 | -6.30869 | -6.13345 | -7.61835 | -6.35612 | -6.69393 | -6.69393 | -9.27272 | -8.79481 | -8.73537 | -8.79481 |
| P7195\|LDHB_HUMAN | -5.79752 | -5.56768 | -5.78924 | -5.78924 | -7.87134 | -7.31943 | -7.38174 | -7.38174 | -11.2265 | -10.9445 | -10.9403 | -10.9445 |
| P3566\|COPB2_HUMAN | 0 | 0 | 0 | 0 | 0 | 0 | 0 | 0 | 0 | 0 | 0 | 0 |
| P49354\|FNTA_HUMAN | 0 | 0 | 0 | 0 | 0 | 0 | 0 | 0 | 0 | 0 | 0 | 0 |
| Q99832\|TCPH_HUMAN | 0 | 0 | 0 | 0 | 0 | 0 | 0 | 0 | 0 | 0 | 0 | 0 |
| P2866\|PSA5_HUMAN | 0 | 0 | 0 | 0 | 0 | 0 | 0 | 0 | 0 | 0 | 0 | 0 |
| P11216\|PYGB_HUMAN | 0 | 0 | 0 | 0 | 0 | 0 | 0 | 0 | 0 | 0 | 0 | 0 |
| Q9Y5Z4\|HEBP2_HUMAN | 0 | 0 | 0 | 0 | 0 | 0 | 0 | 0 | 0 | 0 | 0 | 0 |
| Q9H6S3\|ES8L2_HUMAN | 0 | 0 | 0 | 0 | 0 | 0 | 0 | 0 | 0 | 0 | 0 | 0 |
| P52788\|SPSY_HUMAN | 0 | 0 | 0 | 0 | 0 | 0 | 0 | 0 | 0 | 0 | 0 | 0 |
| Q721\|C1QBP_HUMAN | 0 | 0 | 0 | 0 | 0 | 0 | 0 | 0 | 0 | 0 | 0 | 0 |
| Q96FW1\|OTUB1_HUMAN | 0 | 0 | 0 | 0 | 0 | 0 | 0 | 0 | 0 | 0 | 0 | 0 |
| O75882\|ATRN_HUMAN | 0 | 0 | 0 | 0 | 0 | 0 | 0 | 0 | 0 | 0 | 0 | 0 |
| Q6PID8\|KLD1_HUMAN | 0 | 0 | 0 | 0 | 0 | 0 | 0 | 0 | 0 | 0 | 0 | 0 |
| P326\|ADH1G_HUMAN | 0 | 0 | 0 | 0 | 0 | 0 | 0 | 0 | 0 | 0 | 0 | 0 |
| P7327\|ADH1A_HUMAN | 0 | 0 | 0 | 0 | 0 | 0 | 0 | 0 | 0 | 0 | 0 | 0 |
| Q9H2U2\|IPYR2_HUMAN | 0 | 0 | 0 | 0 | 0 | 0 | 0 | 0 | 0 | 0 | 0 | 0 |
| Q9NSB4\|KRT82_HUMAN | 0 | 0 | 0 | 0 | 0 | 0 | 0 | 0 | 0 | 0 | 0 | 0 |
| P6744\|G6PI_HUMAN | 0 | 0 | 0 | 0 | 0 | 0 | 0 | 0 | 0 | 0 | 0 | 0 |
| Q6NT89\|TRNP1_HUMAN | 0 | 0 | 0 | 0 | 0 | 0 | 0 | 0 | 0 | 0 | 0 | 0 |
| Q1584\|PDIA6_HUMAN | 0 | 0 | 0 | 0 | 0 | 0 | 0 | 0 | 0 | 0 | 0 | 0 |
| Q8TD6\|AGR3_HUMAN | -7.6932 | -7.13334 | -7.38684 | -7.38684 | -11.8207 | -10.8515 | -8.21011 | -10.8515 | 0 | -10.8029 | 0 | 0 |
| Q9H7S9\|ZN73_HUMAN | 0 | 0 | 0 | 0 | 0 | 0 | 0 | 0 | 0 | 0 | 0 | 0 |
| Q53LP3\|SWAHC_HUMAN | 0 | 0 | 0 | 0 | 0 | 0 | 0 | 0 | 0 | 0 | 0 | 0 |
| P25398\|RS12_HUMAN | 0 | 0 | 0 | 0 | 0 | 0 | 0 | 0 | 0 | 0 | 0 | 0 |
| P48634\|PRC2A_HUMAN | 0 | 0 | 0 | 0 | 0 | 0 | 0 | 0 | 0 | 0 | 0 | 0 |
| Q9UL51\|HCN2_HUMAN | 0 | 0 | 0 | 0 | 0 | 0 | 0 | 0 | 0 | 0 | 0 | 0 |
| Q9HAP2\|MLXIP_HUMAN | 0 | 0 | 0 | 0 | 0 | 0 | 0 | 0 | 0 | 0 | 0 | 0 |
| Q86UE8\|TLK2_HUMAN | 0 | 0 | 0 | 0 | 0 | 0 | 0 | 0 | 0 | 0 | 0 | 0 |
| Q9NY33\|DPP3_HUMAN | 0 | 0 | 0 | 0 | 0 | 0 | 0 | 0 | 0 | 0 | 0 | 0 |
| Q96JH8\|RADIL_HUMAN | 0 | 0 | 0 | 0 | 0 | 0 | 0 | 0 | 0 | 0 | 0 | 0 |
| P36955\|PEDF_HUMAN | 0 | 0 | 0 | 0 | 0 | 0 | 0 | 0 | 0 | 0 | 0 | 0 |
| Q86UT5\|NHRF4_HUMAN | 0 | 0 | 0 | 0 | 0 | 0 | 0 | 0 | 0 | 0 | 0 | 0 |
| Q8NCB2\|CAMKV_HUMAN | 0 | 0 | 0 | 0 | 0 | 0 | 0 | 0 | 0 | 0 | 0 | 0 |
| Q6SPF\|SAMD1_HUMAN | 0 | 0 | 0 | 0 | 0 | 0 | 0 | 0 | 0 | 0 | 0 | 0 |
| Q86YR7\|MF2L2_HUMAN | 0 | 0 | 0 | 0 | 0 | 0 | 0 | 0 | 0 | 0 | 0 | 0 |
| Q9NRI5\|DISC1_HUMAN | 0 | 0 | 0 | 0 | 0 | 0 | 0 | 0 | 0 | 0 | 0 | 0 |
| P25774\|CATS_HUMAN | 0 | 0 | 0 | 0 | 0 | 0 | 0 | 0 | 0 | 0 | 0 | 0 |
| O6437\|PEPL_HUMAN | 0 | 0 | 0 | 0 | 0 | 0 | 0 | 0 | 0 | 0 | 0 | 0 |
| A8MYP8\|ODF3B_HUMAN | 0 | 0 | 0 | 0 | 0 | 0 | 0 | 0 | 0 | 0 | 0 | 0 |
| Q6UB35\|C1TM_HUMAN | 0 | 0 | 0 | 0 | 0 | 0 | 0 | 0 | 0 | 0 | 0 | 0 |
| AA286YF58\|TM271_HUMAN | 0 | 0 | 0 | 0 | 0 | 0 | 0 | 0 | 0 | 0 | 0 | 0 |
| Q9BXJ3\|C1QT4_HUMAN | 0 | 0 | 0 | 0 | 0 | 0 | 0 | 0 | 0 | 0 | 0 | 0 |
| O716\|E2F3_HUMAN | 0 | 0 | 0 | 0 | 0 | 0 | 0 | 0 | 0 | 0 | 0 | 0 |
| Q6ZMY3\|SPOC1_HUMAN | 0 | 0 | 0 | 0 | 0 | 0 | 0 | 0 | 0 | 0 | 0 | 0 |
| PDOY3\|IGLC3_HUMAN | -3.96072 | -3.6532 | -3.66934 | -3.66934 | -4.75887 | -3.27937 | -3.56763 | -3.56763 | -7.03295 | -7.04294 | -6.39066 | -7.03295 |
| PDOY2\|IGLC2_HUMAN | -3.96072 | -3.6532 | -3.66934 | -3.66934 | -4.75887 | -3.27937 | -3.56763 | -3.56763 | -7.03295 | -7.04294 | -6.39066 | -7.03295 |
| P68371\|TBB4B_HUMAN | -4.25789 | -4.32142 | -3.30963 | -4.25789 | -6.40031 | -6.18122 | -6.26498 | -6.26498 | -9.00948 | -8.22203 | -7.22325 | -8.22203 |
| AM8Q6\|IGLC7_HUMAN | -6.78895 | -8.01549 | -7.569 | -7.569 | -8.05612 | -6.20689 | -6.36565 | -6.36565 | 0 | 0 | 0 | 0 |
| P8779\|K1C16_HUMAN | -7.7978 | -9.88403 | -9.67966 | -9.67966 | -7.49345 | -7.42511 | -8.73081 | -7.49345 | -10.3731 | -9.8281 | -8.27328 | -9.8281 |
| AAB4J1V\|HV315_HUMAN | -5.04377 | -2.72177 | -4.36388 | -4.36388 | -5.66823 | -2.07448 | -4.32634 | -4.32634 | -7.40307 | -6.3047 | -7.32435 | -7.32435 |
| Q1469\|FABP5_HUMAN | -3.48429 | -2.58841 | -0.56938 | -2.58841 | -6.70974 | -4.57404 | -3.12605 | -4.57404 | -5.78876 | -6.20813 | -5.21652 | -5.78876 |
| AAC4DH25\|KVD2_HUMAN | -6.95511 | -5.13533 | -5.60806 | -5.60806 | -7.43304 | -5.17811 | -6.74124 | -6.74124 | -8.44812 | -9.04429 | -9.30332 | -9.04429 |
| Q6S8J3\|POTEE_HUMAN | -9.27279 | -10.2232 | -10.6284 | -10.2232 | -11.7487 | -10.7879 | -10.4532 | -10.7879 | -8.96617 | -9.88377 | -8.46689 | -8.96617 |
| P747\|PLMN_HUMAN | -5.92549 | -3.49469 | -4.97289 | -4.97289 | -8.40331 | -7.24304 | -8.27237 | -8.27237 | 0 | -9.88322 | 0 | 0 |
| P31947\|1433S_HUMAN | -4.50195 | -4.14112 | -2.28113 | -4.14112 | -8.00836 | -5.45783 | -4.77208 | -5.45783 | -9.89013 | -6.89272 | -6.99501 | -6.99501 |
| Q9UBG3\|CRNN_HUMAN | -7.04974 | -7.29538 | -8.01838 | -7.29538 | -7.84409 | -6.34977 | -2.95284 | -6.34977 | -8.65916 | -7.46862 | -7.62502 | -7.62502 |
| AAB4J1Y9\|HV372_HUMAN | -6.7753 | -3.71951 | -5.25497 | -5.25497 | -6.92526 | -2.92793 | -5.09391 | -5.09391 | -7.58975 | -7.41034 | -8.79366 | -7.58975 |
| P28325\|CYTD_HUMAN | -3.02548 | -1.03484 | -2.02381 | -2.02381 | -4.20534 | -2.06141 | -2.96195 | -2.96195 | -6.16485 | -3.59144 | -3.90313 | -3.90313 |
| Q9UBC9\|SPRR3_HUMAN | -4.73677 | -5.23852 | -4.25225 | -4.73677 | -6.91584 | -4.84654 | -2.106 | -4.84654 | -7.20295 | -5.83317 | -4.47291 | -5.83317 |
| P61981\|1433G_HUMAN | 0 | -11.7835 | 0 | 0 | 0 | 0 | 0 | 0 | 0 | -12.0825 | 0 | 0 |
| AAAMRZ8\|KVD11_HUMAN | -6.20251 | -6.02018 | -6.81941 | -6.20251 | -7.30424 | -5.7221 | -6.87776 | -6.87776 | -9.54641 | -9.54063 | -9.73178 | -9.54641 |
| P4433\|KV311_HUMAN | -6.20251 | -6.02018 | -6.81941 | -6.20251 | -7.30424 | -5.7221 | -6.87776 | -6.87776 | -9.54641 | -9.54063 | -9.73178 | -9.54641 |
| P1743\|HV146_HUMAN | -7.45646 | -5.92636 | -7.36175 | -7.36175 | -8.8185 | -5.54299 | -7.02503 | -7.02503 | -9.05651 | -9.42588 | -9.77662 | -9.42588 |
| A2NJV5\|KV229_HUMAN | -9.58488 | -9.27562 | -8.82612 | -9.27562 | 0 | -9.95478 | -10.1532 | -9.95478 | 0 | 0 | 0 | 0 |
| AA75B6S2\|KVD29_HUMAN | -9.58488 | -9.27562 | -8.82612 | -9.27562 | 0 | -9.95478 | -10.1532 | -9.95478 | 0 | 0 | 0 | 0 |
| AAC4DH72\|KV16_HUMAN | -3.61632 | -3.64248 | -4.24921 | -3.64248 | -5.19966 | -3.52405 | -4.82811 | -4.82811 | -3.32534 | -5.28654 | -3.1965 | -3.32534 |
| Q6323\|PSME1_HUMAN | -4.31511 | -3.74839 | -3.14511 | -3.74839 | -5.59155 | -5.66272 | -5.83218 | -5.66272 | -3.74406 | -3.98484 | -3.94839 | -3.94839 |
| O15143\|ARC1B_HUMAN | -6.4888 | -4.84904 | -4.16509 | -4.84904 | -9.12551 | -8.48042 | -9.8307 | -9.12551 | 0 | -10.4366 | -10.1038 | -10.1038 |
| AA75B6S9\|KV137_HUMAN | -6.06684 | -5.56076 | -6.08009 | -6.06684 | -7.26294 | -5.19568 | -6.01705 | -6.01705 | -9.13244 | -8.80685 | -9.14042 | -9.13244 |
| PDSN7\|KVD37_HUMAN | -6.06684 | -5.56076 | -6.08009 | -6.06684 | -7.26294 | -5.19568 | -6.01705 | -6.01705 | -9.13244 | -8.80685 | -9.14042 | -9.13244 |
| AAC4DH42\|HV366_HUMAN | -5.11334 | -3.94081 | -3.95894 | -3.95894 | -6.28924 | -3.4938 | -4.11825 | -4.11825 | -7.51746 | -6.65479 | -7.44101 | -7.44101 |
| AAC4DH34\|HV428_HUMAN | -4.81852 | -3.19134 | -3.09771 | -3.19134 | -6.23547 | -3.04065 | -3.37489 | -3.37489 | 0 | -7.67706 | -6.70826 | -6.70826 |
| Q8431\|MFGM_HUMAN | -5.91381 | -6.51646 | -8.14373 | -6.51646 | -8.10512 | -8.20011 | -8.93224 | -8.20011 | -11.0096 | -10.1628 | -11.3434 | -11.0096 |
| O764\|PDXK_HUMAN | -8.43694 | -8.20306 | -7.11164 | -8.20306 | 0 | -11.3039 | -11.9744 | -11.3039 | 0 | 0 | -11.8586 | 0 |
| P441\|SODC_HUMAN | -6.13028 | -6.35076 | -5.20765 | -6.13028 | -7.941 | -7.87689 | -8.06561 | -7.941 | -6.75645 | -10.4119 | -7.0296 | -7.0296 |
| AAC4DH29\|HV13_HUMAN | -9.01314 | -10.6777 | -8.79622 | -9.01314 | -9.84685 | -11.1586 | -8.42284 | -9.84685 | 0 | 0 | -9.814 | 0 |
| Q15293\|RCN1_HUMAN | -4.07375 | -4.63033 | -4.92323 | -4.63033 | -5.16811 | -5.37967 | -5.42501 | -5.37967 | -7.69953 | -5.80345 | -7.64407 | -7.64407 |
| P5564\|AQP5_HUMAN | -6.32313 | -6.19073 | -6.27864 | -6.27864 | -7.45375 | -6.2503 | -7.10193 | -7.10193 | 0 | -11.0664 | -8.03359 | -8.03359 |
| AA75B6I\|LV861_HUMAN | -5.3676 | -3.48953 | -6.76521 | -5.3676 | -6.04958 | -2.98133 | -6.77929 | -6.04958 | -7.09856 | -7.14707 | -9.55427 | -7.14707 |
| P14\|CYTA_HUMAN | -8.16861 | -10.3834 | -8.95097 | -8.95097 | 0 | -9.36368 | -8.03122 | -8.03122 | 0 | 0 | -11.7281 | 0 |
| P4179\|SODM_HUMAN | -6.63829 | -3.65982 | -4.43773 | -4.43773 | -7.93897 | -7.87856 | -5.71457 | -7.87856 | -10.6633 | -8.71005 | -7.89141 | -8.71005 |
| P15814\|IGLL1_HUMAN | -3.83145 | -1.95386 | -1.73815 | -1.95386 | -2.7162 | -0.09647 | -0.52207 | -0.52207 | -4.52422 | -4.39179 | -4.18652 | -4.39179 |
| P19961\|AMY2B_HUMAN | -7.45646 | -7.02276 | -6.95811 | -7.02276 | -9.87683 | -8.357 | -8.9082 | -8.9082 | -12.0663 | -9.76743 | -10.5511 | -10.5511 |
| PDTE7\|AMY1B_HUMAN | -7.45646 | -7.02276 | -6.95811 | -7.02276 | -9.87683 | -8.357 | -8.9082 | -8.9082 | -12.0663 | -9.76743 | -10.5511 | -10.5511 |
| P4746\|AMYP_HUMAN | -7.45646 | -7.02276 | -6.95811 | -7.02276 | -9.87683 | -8.357 | -8.9082 | -8.9082 | -12.0663 | -9.76743 | -10.5511 | -10.5511 |
| P4745\|AMY1A_HUMAN | -7.45646 | -7.02276 | -6.95811 | -7.02276 | -9.87683 | -8.357 | -8.9082 | -8.9082 | -12.0663 | -9.76743 | -10.5511 | -10.5511 |
| PDTE8\|AMY1C_HUMAN | -7.45646 | -7.02276 | -6.95811 | -7.02276 | -9.87683 | -8.357 | -8.9082 | -8.9082 | -12.0663 | -9.76743 | -10.5511 | -10.5511 |
| Q796\|DHSO_HUMAN | -5.50933 | -4.11838 | -2.20998 | -4.11838 | -7.02458 | -6.36911 | -7.11023 | -7.02458 | -8.62783 | -8.12236 | -8.41383 | -8.41383 |
| O6664\|PLIN3_HUMAN | -4.14129 | -3.27784 | -3.05591 | -3.27784 | -5.63975 | -3.92269 | -4.37154 | -4.37154 | -7.65634 | -6.47153 | -6.68418 | -6.68418 |
| P1742\|HV169_HUMAN | 0 | -9.13151 | -8.90415 | -8.90415 | -11.8893 | -9.03226 | -10.1149 | -10.1149 | 0 | 0 | 0 | 0 |
| AAB4J2H\|HV69D_HUMAN | 0 | -9.13151 | -8.90415 | -8.90415 | -11.8893 | -9.03226 | -10.1149 | -10.1149 | 0 | 0 | 0 | 0 |
| PDOX3\|IGD_HUMAN | -7.30689 | -6.27072 | -6.99687 | -6.99687 | -8.17706 | -5.94687 | -7.58337 | -7.58337 | -10.8146 | -9.63005 | -10.1529 | -10.1529 |
| AA75B6I9\|LV746_HUMAN | -6.44701 | -4.90809 | -5.38109 | -5.38109 | -7.46096 | -5.20252 | -5.84991 | -5.84991 | -8.70681 | -9.09578 | -8.21813 | -8.70681 |
| P4211\|LV743_HUMAN | -6.44701 | -4.90809 | -5.38109 | -5.38109 | -7.46096 | -5.20252 | -5.84991 | -5.84991 | -8.70681 | -9.09578 | -8.21813 | -8.70681 |
| AA75B6H8\|KVD42_HUMAN | -7.35869 | -6.94936 | -7.13918 | -7.13918 | -8.60633 | -6.11091 | -7.57517 | -7.57517 | -11.384 | -10.2829 | -10.8411 | -10.8411 |
| AA75B6J9\|LV218_HUMAN | -7.16518 | -5.90137 | -6.34204 | -6.34204 | -8.25145 | -5.48911 | -7.55002 | -7.55002 | -9.56872 | -8.87749 | -9.63736 | -9.56872 |
| AAB4J1U7\|HV61_HUMAN | -7.35882 | -5.26158 | -6.87899 | -6.87899 | -9.17079 | -4.87577 | -7.07178 | -7.07178 | -9.66471 | -8.75009 | -11.4922 | -9.66471 |
| P37837\|TALDO_HUMAN | -5.44894 | -5.77991 | -4.79324 | -5.44894 | -8.07702 | -7.71289 | -7.46254 | -7.71289 | -8.99385 | -8.26179 | -9.04244 | -8.99385 |
| AAAMT36\|KVD21_HUMAN | -7.21907 | -6.05001 | -6.62583 | -6.62583 | -8.04773 | -5.44395 | -6.4131 | -6.4131 | -9.85265 | -9.13201 | -9.62602 | -9.62602 |
| AAC4DH24\|KV621_HUMAN | -7.21907 | -6.05001 | -6.62583 | -6.62583 | -8.04773 | -5.44395 | -6.4131 | -6.4131 | -9.85265 | -9.13201 | -9.62602 | -9.62602 |
| P13639\|EF2_HUMAN | -6.73469 | -7.0359 | -6.5208 | -6.73469 | -10.4776 | -8.22239 | -10.5841 | -10.4776 | -11.7495 | 0 | -11.2862 | -11.2862 |
| P171\|LV151_HUMAN | -6.87588 | -5.84964 | -7.46659 | -6.87588 | -8.04749 | -5.16749 | -7.24596 | -7.24596 | -8.88457 | -7.84574 | -9.38372 | -8.88457 |
| AA75B6I4\|LVX54_HUMAN | -6.82434 | -4.1534 | -5.62851 | -5.62851 | -7.75264 | -3.35633 | -5.22818 | -5.22818 | -8.67106 | -7.16186 | -7.95444 | -7.95444 |
| P1721\|LV657_HUMAN | -7.6062 | -6.89409 | -6.57173 | -6.89409 | -8.78841 | -6.17818 | -6.35769 | -6.35769 | -10.4889 | -9.79289 | -10.1564 | -10.1564 |
| Q9BUP\|EFHD1_HUMAN | -5.22031 | -4.15213 | -3.23392 | -4.15213 | -7.77264 | -6.7873 | -7.62439 | -7.62439 | -9.52686 | -8.7859 | -8.53752 | -8.7859 |
| Q96C19\|EFHD2_HUMAN | -5.22031 | -4.15213 | -3.23392 | -4.15213 | -7.77264 | -6.7873 | -7.62439 | -7.62439 | -9.52686 | -8.7859 | -8.53752 | -8.7859 |
| AA75B6Q5\|HV364_HUMAN | -8.21479 | -7.99218 | -5.58138 | -7.99218 | -10.2378 | -7.81478 | -6.23777 | -7.81478 | -11.1924 | -11.7416 | -8.83796 | -11.1924 |
| Q132\|PSMD2_HUMAN | -9.34048 | 0 | -11.466 | -9.34048 | 0 | 0 | 0 | 0 | 0 | 0 | 0 | 0 |
| Q9UKY7\|CDV3_HUMAN | -8.34661 | -9.46856 | -8.81197 | -8.81197 | 0 | -10.866 | -10.6863 | -10.6863 | 0 | 0 | 0 | 0 |
| Q99574\|NEUS_HUMAN | -5.43465 | -5.94951 | -6.42265 | -5.94951 | -7.74176 | -7.10532 | -7.36611 | -7.36611 | -9.95568 | -9.43983 | -10.5984 | -9.95568 |
| O391\|QSOX1_HUMAN | -4.12256 | -3.88424 | -4.379 | -4.12256 | -5.49619 | -5.44216 | -6.11881 | -5.49619 | -8.12754 | -6.65551 | -7.22187 | -7.22187 |
| P173\|LV14_HUMAN | -6.23019 | -6.62594 | -6.94521 | -6.62594 | -7.67263 | -6.67755 | -7.64922 | -7.64922 | -8.23589 | -7.79847 | -8.95789 | -8.23589 |
| P23526\|SAHH_HUMAN | -4.09285 | -2.53411 | -1.13874 | -2.53411 | -6.709 | -5.48256 | -6.36715 | -6.36715 | -7.78578 | -7.42426 | -6.59968 | -7.42426 |
| P25786\|PSA1_HUMAN | -7.09309 | -6.75886 | -5.48831 | -6.75886 | -10.4042 | -10.1425 | -9.49331 | -10.1425 | 0 | 0 | -9.9068 | 0 |
| O43548\|TGM5_HUMAN | -5.45717 | -2.37024 | -4.05334 | -4.05334 | -4.8413 | -3.73963 | -4.92713 | -4.8413 | -6.86222 | -7.6362 | -7.1168 | -7.1168 |
| Q9Y6U3\|ADSV_HUMAN | -7.38876 | -8.51913 | -5.91487 | -7.38876 | -10.8167 | -9.77593 | -10.8029 | -10.8029 | 0 | -11.0555 | -11.3678 | -11.0555 |
| Q9UBZ9\|REV1_HUMAN | -6.50786 | -5.67627 | -5.97163 | -5.97163 | -8.03219 | -5.19396 | -5.87002 | -5.87002 | -9.37667 | -8.83091 | -8.83293 | -8.83293 |
| Q8WXS5\|CCG8_HUMAN | -8.68729 | -9.47825 | -10.5762 | -9.47825 | -11.5747 | -10.3948 | -10.6298 | -10.6298 | -10.4506 | -11.9807 | 0 | -10.4506 |
| O1499\|IPP2C_HUMAN | -7.78616 | -9.57403 | -8.68135 | -8.68135 | -9.91604 | -9.10612 | -8.75333 | -9.10612 | 0 | -11.5983 | 0 | 0 |
| P3866\|VATA_HUMAN | 0 | -10.5278 | -10.2279 | -10.2279 | 0 | -11.7195 | 0 | 0 | 0 | 0 | 0 | 0 |
| Q15782\|CH3L2_HUMAN | -3.03088 | -4.11133 | -5.00466 | -4.11133 | -5.46689 | -5.6974 | -6.19781 | -5.6974 | -8.80914 | -7.53849 | -7.83487 | -7.83487 |
| P23284\|PPIB_HUMAN | -5.557 | -4.65852 | -4.9682 | -4.9682 | -7.34281 | -7.62184 | -7.26486 | -7.34281 | -10.2512 | -10.5484 | -9.63631 | -10.2512 |
| Q7Z572\|SPT21_HUMAN | -8.97691 | -9.2032 | -8.75635 | -8.97691 | -10.3338 | -8.87541 | -9.36471 | -9.36471 | 0 | 0 | 0 | 0 |
| Q15365\|PCBP1_HUMAN | -6.31477 | -5.72508 | -4.73005 | -5.72508 | -8.73839 | -8.07755 | -7.76536 | -8.07755 | -10.821 | -8.88838 | -9.07074 | -9.07074 |
| Q15366\|PCBP2_HUMAN | -6.31477 | -5.72508 | -4.73005 | -5.72508 | -8.73839 | -8.07755 | -7.76536 | -8.07755 | -10.821 | -8.88838 | -9.07074 | -9.07074 |
| P57721\|PCBP3_HUMAN | -6.31477 | -5.72508 | -4.73005 | -5.72508 | -8.73839 | -8.07755 | -7.76536 | -8.07755 | -10.821 | -8.88838 | -9.07074 | -9.07074 |
| P57723\|PCBP4_HUMAN | -6.31477 | -5.72508 | -4.73005 | -5.72508 | -8.73839 | -8.07755 | -7.76536 | -8.07755 | -10.821 | -8.88838 | -9.07074 | -9.07074 |
| Q7Z614\|SNX2_HUMAN | -9.31718 | -9.75652 | -8.64725 | -9.31718 | -9.21823 | -7.28268 | -7.96766 | -7.96766 | -10.0941 | -9.11027 | -10.5617 | -10.0941 |
| A6NKD9\|CC85C_HUMAN | -4.76711 | -5.45347 | -6.09707 | -5.45347 | -7.32608 | -6.87634 | -7.38615 | -7.32608 | -9.06788 | -8.5537 | -8.73223 | -8.73223 |
| Q13315\|ATM_HUMAN | -5.59488 | -6.05496 | -6.28716 | -6.05496 | -7.74877 | -7.92288 | -7.80859 | -7.80859 | -9.48319 | -8.22321 | -8.07174 | -8.22321 |
| Q14624\|ITIH4_HUMAN | -5.83 | -7.95714 | -8.0049 | -7.95714 | -7.93047 | -9.19649 | -8.92508 | -8.92508 | 0 | 0 | 0 | 0 |
| P46459\|NSF_HUMAN | -5.11233 | -4.95349 | -4.43279 | -4.95349 | -6.58017 | -5.85696 | -7.29114 | -6.58017 | -8.91995 | -8.42264 | -9.36574 | -8.91995 |
| Q9UBQ7\|GRHPR_HUMAN | -5.71754 | -5.2182 | -4.02781 | -5.2182 | -7.26448 | -7.05537 | -7.75335 | -7.26448 | -7.28048 | -7.69339 | -6.79816 | -7.28048 |
| P1514\|GLNA_HUMAN | -6.57431 | -7.73567 | -5.4922 | -6.57431 | -11.9443 | -11.2462 | -7.75211 | -11.2462 | 0 | -11.5531 | -10.9723 | -10.9723 |
| Q9Y263\|PLAP_HUMAN | -8.55258 | 0 | -11.9655 | -8.55258 | -10.7387 | 0 | -11.3916 | -10.7387 | 0 | 0 | 0 | 0 |
| Q9NZJ7\|MTCH1_HUMAN | -5.41013 | -5.99973 | -6.71807 | -5.99973 | -5.31538 | -6.0638 | -5.90976 | -5.90976 | -7.96432 | -5.76633 | -7.55124 | -7.55124 |
| P23588\|IF4B_HUMAN | -9.46672 | 0 | 0 | 0 | 0 | 0 | 0 | 0 | 0 | 0 | -11.8173 | 0 |
| Q86UL8\|MAGI2_HUMAN | -7.64988 | -8.77621 | -10.2588 | -8.77621 | -8.43808 | -7.23634 | -7.66773 | -7.66773 | 0 | -9.11548 | 0 | 0 |
| Q9Y6X\|SETBP_HUMAN | -3.4825 | -4.5692 | -4.67115 | -4.5692 | -7.00819 | -6.50528 | -7.24876 | -7.00819 | -9.86998 | -8.98744 | -8.75916 | -8.98744 |
| Q8WUM4\|PDC6I_HUMAN | -9.16083 | -8.00162 | -8.13802 | -8.13802 | -11.5054 | -9.6534 | -8.97791 | -9.6534 | 0 | -10.8151 | 0 | 0 |
| P69\|PSA6_HUMAN | 0.38969 | -0.21912 | -0.70421 | -0.21912 | -3.70065 | -3.487 | -3.0367 | -3.487 | -1.34354 | -1.86975 | -1.63049 | -1.63049 |
| Q6ZS81\|WDFY4_HUMAN | -6.30875 | -7.29918 | -8.27882 | -7.29918 | -8.23932 | -7.85443 | -8.54801 | -8.23932 | -9.86274 | -8.76916 | -9.87592 | -9.86274 |
| Q96T58\|MINT_HUMAN | -6.30165 | -8.95476 | -8.73953 | -8.73953 | -8.65202 | -9.10635 | -8.20514 | -8.65202 | 0 | 0 | 0 | 0 |
| Q1368\|PEX6_HUMAN | -8.50825 | -10.7353 | -11.0904 | -10.7353 | -9.77182 | -10.3424 | -9.65814 | -9.77182 | -9.80874 | -10.2218 | -10.2691 | -10.2218 |
| Q9HBH5\|RDH14_HUMAN | -3.07424 | -4.29757 | -4.37644 | -4.29757 | -3.77712 | -5.10279 | -3.98916 | -3.98916 | -8.09945 | -6.91209 | -7.98198 | -7.98198 |
| O95969\|SG1D2_HUMAN | -5.58549 | -8.01102 | -7.85172 | -7.85172 | -7.94081 | -8.94095 | -9.31485 | -8.94095 | 0 | 0 | -9.49075 | 0 |
| Q9NRU3\|CNNM1_HUMAN | -4.58006 | -5.24679 | -6.29032 | -5.24679 | -5.94336 | -6.19265 | -6.09559 | -6.09559 | -9.62199 | -8.48843 | -9.0097 | -9.0097 |
| Q96G74\|OTUD5_HUMAN | -8.84636 | 0 | 0 | 0 | 0 | 0 | -11.2654 | 0 | -10.6999 | -7.15174 | -10.064 | -10.064 |
| Q6ZN18\|AEBP2_HUMAN | -7.17478 | -9.07279 | -9.11309 | -9.07279 | -9.03241 | -11.0023 | -9.7334 | -9.7334 | 0 | 0 | -11.771 | 0 |
| P2654\|APOC1_HUMAN | -7.42677 | -6.56754 | -7.96908 | -7.42677 | -11.7395 | -9.83224 | -10.7539 | -10.7539 | 0 | -11.9459 | 0 | 0 |

**Supplementary Methods S1**

Subjects 1, 3, and 5 were SCL wearers. Subjects 2, 4, and 6 reported they were not current or previous SCL wearers. Two groups of 3 human subjects were used to create two TMT 10-plexes (TF5 and TF6). Each 10-plex contained 3 sets of tears (SS, MCT, SCL) from the same 3 human subjects and 1 pooled sample.

Samples were grouped and TMT labeled as described in Supplementary Table S6
